# Whole genome resequencing of four Italian sweet pepper landraces provides insights on sequence variation in genes of agronomic value

**DOI:** 10.1038/s41598-020-66053-2

**Published:** 2020-06-08

**Authors:** Alberto Acquadro, Lorenzo Barchi, Ezio Portis, Mohamed Nourdine, Cristiano Carli, Simone Monge, Danila Valentino, Sergio Lanteri

**Affiliations:** 1 DISAFA, Plant Genetics and Breeding, University of Torino, Grugliasco, Italy; 2 Agrion, Foundation for Research, Innovation and Technological Development of Piedmontese Agriculture, Manta, CN Italy; 3 Confagricoltura, Cuneo, CN Italy

**Keywords:** Genomics, Plant genetics

## Abstract

Sweet pepper (*Capsicum annuum* L.) is a high value crop and one of the most widely grown vegetables belonging to the Solanaceae family. In addition to commercial varieties and F_1_ hybrids, a multitude of landraces are grown, whose genetic combination is the result of hundreds of years of random, environmental, and farmer selection. High genetic diversity exists in the landrace gene pool which however has scarcely been studied, thus bounding their cultivation. We re-sequenced four pepper inbred lines, within as many Italian landraces, which representative of as many fruit types: big sized blocky with sunken apex (‘Quadrato’) and protruding apex or heart shaped (‘Cuneo’), elongated (‘Corno’) and smaller sized sub-spherical (‘Tumaticot’). Each genomic sequence was obtained through Illumina platform at coverage ranging from 39 to 44×, and reconstructed at a chromosome scale. About 35.5k genes were predicted in each inbred line, of which 22,017 were shared among them and the reference genome (accession ‘CM334’). Distinctive variations in miRNAs, resistance gene analogues (RGAs) and susceptibility genes (S-genes) were detected. A detailed survey of the SNP/Indels occurring in genes affecting fruit size, shape and quality identified the highest frequencies of variation in regulatory regions. Many structural variations were identified as presence/absence variations (PAVs), notably in resistance gene analogues (RGAs) and in the capsanthin/capsorubin synthase (CCS) gene. The large allelic diversity observed in the four inbred lines suggests their potential use as a pre-breeding resource and represents a one-stop resource for *C. annuum* genomics and a key tool for dissecting the path from sequence variation to phenotype.

## Introduction

The *Capsicum* genus (Solanaceae family) originated in the New World and includes 27 species of which five (*C. annuum, C. chinense, C. frutescens, C. baccatum*, and *C. pubescens*) have been domesticated^1^ and used worldwide as spices and vegetables. The most cultivated is *C. annuum* L. (2n = 2×= 24), which includes most of the Mexican chili peppers, hot peppers grown in Africa and Asia as well as numerous cultivars and landraces of sweet (non pungent) peppers cultivated in the temperate regions of Europe and North America^1^. China is at present the main worldwide pepper producer, with more than 17 M tons, followed by Mexico, Turkey and Indonesia. Italy, Spain and the Netherlands are the main European producers, and in several regions of the former, the heterogeneity of the land, climate and soil has favoured the cultivation of landraces. Each of them is the result of selection processes for adaptation to specific ecological niches, and differ in fruit shape, organoleptic properties and resistance against abiotic and biotic stresses^2^.

Molecular markers techniques and gene chip arrays have been applied in the last decades for pepper germplasm characterization^3–10^ with the goal to provide complementary descriptors to conventional morphological variation. However, triggered by advancements in sequencing technologies, the genome sequence of several crop is now available and the application of NGS technologies for resequencing represent the most powerful tool for exploring the DNA‐level diversity among members of a crop, with the ultimate goal to understand the molecular basis for phenotype–genotype relationships^11^. The first whole-genome sequences of *C. annuum* ('CM334') and *C. chinense* (PI159236) were released by Kim *et al*.^12^ in 2014. In the same year, Qin *et al*.^13^ published the genome sequences of *C. annuum* Zunla-1 and of Chiltepin (*C. annuum* var. *glabriusculum*). Both studies highlighted that the pepper genome size is ~3–3.5 Gb, it is characterized by a high percentage (over 80%) of repetitive elements, and includes 35 K genes. Afterwards, a resequencing effort^14^ made available the raw sequences of two *C. annuum* lines: ‘Dempsey’, a large bell-type genotype, and ‘Perennial’, a genotype with small elongated fruits. A few years later, an improved version of the reference genome of both ‘CM334’ and *C. chinense* ‘PI159236’ as well as the genome sequence of the domesticated *C. baccatum* were published and contributed to decipher the evolutionary relationships among the three species as well as to estimate the lineage-divergence times occurring in *Capsicum*^15^. Recently, Hulse-Kemp *et al*.^16^, by adopting the linked-read sequencing technology, obtained the genome sequence of an F_1_ hybrid from a cross between ‘CM334’ with a non-pungent blocky accession of *C. annuum*. In 2019, Du and co-worker^17^, to identifying a panel of 92 SNPs for population structure analysis, produced low-pass resequencing data from 35 different *C. annuum* lines.

Here we report on the resequencing of four sweet pepper inbred lines, which we selected within four Italian landraces representative of as many fruit types, ranging from blocky to elongated and sub-spherical. The inbred lines produce sweet fruits with peculiar organoleptic and sensorial characteristics and of potential use for both fresh consumption and industrial transformation. The four genomes were reconstructed at a chromosomal scale and annotated. MiRNA loci as well as the number, position and phylogenetic relationships of putative resistance gene analogues (RGAs) and susceptibility genes (S-genes)^18^ were identified. Lastly, functionally characterised SNP/Indels and PAV in genes affecting berry size, shape and pigmentation were spotted.

## Results and Discussion

### Fruit morphological characterization

Four pepper lines, previously selected from landraces through a breeding program developed by DISAFA (www.disafa.unito.it), in collaboration with AGRION (www.agrion.it), were under study: ‘Cuneo’, ‘Quadrato di Carmagnola’ (later called ‘Quadrato’), ‘Corno di Carmagnola’ (later called ‘Corno’) and ‘Tumaticot’. The landrace ‘Cuneo’ produces red or yellow hearth shaped berries, mostly tri –lobed and, owning a fleshy and crunchy pericarp whose thickness ranges from 8 to 10 mm, and with a protruding apex characterized by a typical brownish anthocyanic spot, commonly called ‘moustache’. The landrace ‘Quadrato’ produces tetra-lobated berries with a sunken apex, a diameter ranging from 12 to 18 cm and whose pericarp is fleshy and aromatic. The landrace ‘Corno’ produces elongated fruits characterized by thinner pericarp which maintain its red or yellow color also after cooking, making them particularly suitable for the processing industry. ‘Tumaticot’, a landrace grown in the Piedmont region (North-West Italy) since the early ‘30 s, is characterized by smaller fruits, with a flattened shape and a flat or slightly sunken apex. The fruits are tri or tetra-lobated, with a color nuance from red to yellow and a pericarp thickness of 7–11 mm.

The average fruit weight and related components (diameter, length, fruit shape, thickness of the pericarp, number of lodges) of the inbred lines in study are reported in Table 1. Data for ‘Perennial’ genotype and ‘CM334’ were added to the analysis as references. The two blocky types ‘Cuneo’ and ‘Quadrato’ produce berries with the greatest average weight (272.7 and 270.5 g respectively), followed by ‘Tumaticot’, which produces heavier fruits (238.1 g) than ‘Corno’ (130.2 g), due to its thicker pericarp (Table 1). The latter is characterized by the longest berries, up to 18 cm (ratio lenght/diameter: 2.82), while ‘Quadrato’ is the only one producing red berries (Table 1).

**Table 1:** Characteristics of the berries of Piedmontese inbred lines, ‘CM334’ and Perennial.

Genotype	Berry colour	Average berry weight (gr)	Fruit Length (cm)	Fruit Diameter (cm)	Ratio L/D	Pericarp thickness (mm)	N° locules
Cuneo*	Yellow	272.7 ± 11.28	8.7 ± 0.27	9.9 ± 0.15	0.80 ± 0.01	6.8 ± 0.20	3.3 ± 0.33
Corno*	Yellow	130.2 ± 11.55	18.2 ± 1.02	5.8 ± 0.31	2.82 ± 0.24	3.3 ± 0.11	3.0 ± 0.11
Quadrato*	Red	270.5 ± 222.72	9.4 ± 0.48	9.3 ± 0.50	1.01 ± 0.03	5.4 ± 0.42	3.7 ± 0.17
Tumaticot**	Yellow	238.1 ± 75.20	4.9 ± 0.10	10.3 ± 0.10	0.48 ± 0.28	5.3 ± 0.10	2.8 ± 0.12
CM334***	Red	6.2 ± 1.10	5.9 ± 0.50	1.9 ± 0.20	3.17 ± 0.27	2.5 ± 0.20	2.3 ± 0.46
Perennial****	Red	1.0 ± 0.00	3.0 ± 0.3	1.0 ± 0.01	3.00 ± 0.30	**—**	2.0 ± 0.00

*Data from Portis *et al*.^2^; **Data from Portis *et al*.^120^; ***Data from Barchi *et al*.^37^; ****Data from Han K. *et al*.^14^.

### Genome assembly and reconstruction

Genome sequencing of the four *C. annuum* genotypes yielded 3.315 million raw pair-end reads (in 1.658 million clusters, Table 2). For each genotype an average of 829 million sequences (150 bp) were produced for a total of ~497.4 Gb, reduced to 465.7 Gb after removal of low-quality bases. The sequencing coverage was 41.46X on average, ranging from 38.76X in ‘Corno’ to 43.54X in ‘Tumaticot’ (Table 2). The sequence data have been deposited into NCBI Short Read Archive with specific submission identifiers (from SAMN14253609 to SAMN14253612), under the Bioproject PRJNA609444. Analyzed data are available in the www.pepper-genomics.unito.it portal.

**Table 2:** Summary of results from sequencing data for each of the four genotypes (before and after the clean-up phase).

Genotype	duplicated sequences (%)		GC (%)		Length of sequences (bp)		Total sequences (M)		Coverage	Read Mapping (%)
	Pre-cleaning	final	Pre-cleaning	Final	Pre-cleaning	Final	Pre-cleaning	Final		
Corno	10.55%	9.97%	35.00%	35.67%	150	141.33	870.8	866.7	43.54 ×	99.10
Cuneo	8.28%	8.19%	35.00%	35.00%	150	141.3	775.2	763.8	38.76 ×	98.11
Quadrato	9.77%	9.67%	35.00%	35.67%	150	141.33	816	812.5	40.80 ×	99.07
Tumaticot	10.94%	10.37%	35.00%	35.67%	150	141.67	854.4	850.4	42.72 ×	99.08
Total	—	—	—	—	—	—	3316.4	3293.4	—	—
Average	9.88%	9.55%	35.00%	35.75%	150	141.41	829.1	823.35	41.46 ×	98.84

Genome reconstruction, hampered by both the large genome size and its richness in repeated elements (~ 80%), was based on a combination of a *de novo* assembly procedure to generate contigs/scaffolds and an iterative read mapping strategy against the pepper reference genome to integrate contigs/scaffolds into pseudomolecules. In respect to a *de novo* assembly, this approach required lower sequencing depth and avoided the construction of multiple libraries.

An extensive k-mer survey (Table S1) identified the k-mer length of 63 as the one granting optimal contiguity metrics, and was adopted for the final assembly of all the genotypes (Table 3). In order to close the gaps emerging during the scaffolding process by SOAPdenovo, we applied Gapcloser, and lowered, in average, the number of unidentified nucleotides (N_s_) in the assemblies from 5.83 to 0.04% (Table 3). The obtained assemblies were successfully reconstructed in 12 pseudomolecules, corresponding to the haploid chromosome number of the species. A variable number of scaffolds were anchored (range 4,062–4,688), while the unanchored fraction, of each genome, was attributed to chr. 0 (range 110–115 Mb).

**Table 3:** Statistics of the four assembled genomes (k = 63), relative to the different steps of the assembly procedure (de novo, Gapclosing, filtering and reference-guided).

Genotype	Metrics	*De novo*	Gap closed	Filtered > 500 bp	Reference guided
Corno	**Number of scaffolds**	1,994,391	1,994,391	313,692	4,688
	**Total size of scaffolds (bp)**	3,238,887,426	3,156,174,802	2,888,201,005	2,952,523,284
	**Longest scaffold**	977,366	967,969	967,969	304,682,413
	**Shortest scaffold**	100	100	500	800
	**Average size (scaffolds, bp)**	1,624	1,583	9,207	629,804
	N_**50**_	47,527	47,339	55,304	244,870,051
	L_**50**_	15,812	15,493	12,873	6
	**% of Ns in scaffolds**	6.63	0.04	0.03	2.97
	**Number of genes**	35,484	—	—	29,204
Cuneo	**Number of scaffolds**	1,209,809	1,209,809	183,951	4,062
	**Total size of scaffolds (bp)**	3,015,917,026	3,003,730,531	2,856,195,490	2,888,888,923
	**Longest scaffold**	785,116	783,752	783,752	298,303,901
	**Shortest scaffold**	100	100	500	800
	**Average size (scaffolds, bp)**	2,493	2,483	15,527	711,199
	N_**50**_	53,466	53,354	57,217	239,928,576
	L_**50**_	14,568	14,539	13,205	6
	**% of Ns in scaffolds**	3.92	0.04	0.03	1.76
	**Number of genes**	35,518	—	—	29,300
Quadrato	**Number of scaffolds**	1,773,028	1,773,028	289,008	4,688
	**Total size of scaffolds (bp)**	3,196,719,955	3,111,823,022	2,880,333,039	2,938,129,169
	**Longest scaffold**	684,241	678,098	678,098	303,210,577
	**Shortest scaffold**	100	100	500	800
	**Average size (scaffolds, bp)**	1,803	1,755	9,966	626,734
	N_**50**_	46,314	46,168	52,573	243,527,465
	L_**50**_	16,181	15,845	13,497	6
	**% of Ns in scaffolds**	6.68	0.04	0.03	2.74
	**Number of genes**	35,538	—	—	29,288
Tumaticot	**Number of scaffolds**	1,191,858	1,191,858	173,746	4,322
	**Total size of scaffolds (bp)**	3,088,650,784	3,016,051,866	2,871,230,234	2,901,582,212
	**Longest scaffold**	788,112	780,295	780,295	300,041,623
	**Shortest scaffold**	100	100	500	800
	**Average size (scaffolds, bp)**	2,591	2,531	16,525	671,352
	N_**50**_	58,072	57,584	61,963	240,199,676
	L_**50**_	13,578	13,360	12,147	6
	**% of Ns in scaffolds**	6.09	0.03	0.02	1.64
	**Number of genes**	35,723	—	—	29,465

^1^The reference guided assembly contained also chromosome zero.

### Genome annotation and OrthoMCL analysis

**Figure 1:**
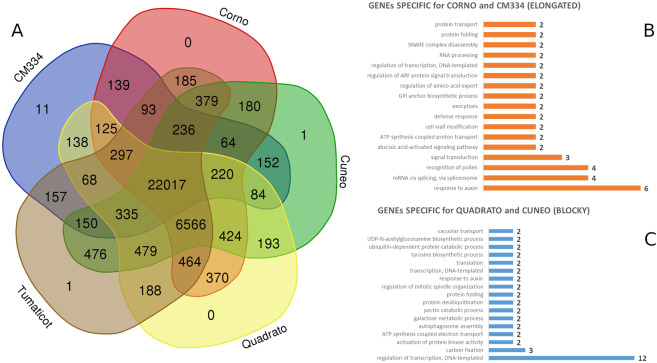
(**A**) Venn diagram showing the grouping of the four proteomes and the one of the reference ‘CM334’ based on Orthofinder. (**B**) GO distribution related to the genes present in the 139 orthogroups specific for the elongated types; (**C**) GO distribution related to the genes present in the 193 orthogroups specific for the blocky types.

Globally, ~80% of the resequenced genomes was masked, in line with recent findings^15,19^. The four assembled genomes were then structurally annotated with the Maker-P suite and the total number of genes identified was on average 35.5k (Fig. 1, AED ~ 0.35). The lowest number of genes (Table 3) was detected in ‘Corno’ (35.484) while the highest in ‘Tumaticot’ (35.723). No correlation between size of the assembled genomes and the number of predicted genes was highlighted; this suggests that the different genome sizes are attributable to non-coding regions or structural variants.

The functional annotation produced about 35.5k proteins, in line with what previously detected within the Solanaceae family (34.9k in ‘CM334’, 35.34k in ‘Zunla-1’, 34.5k in ‘Chiltepin’, 33.7k in tomato, 35,0k in eggplant and 38.5k in potato). Proteomes were validated using BUSCO; overall, more than 91% of 1,614 expected embryophyta genes were identified in our genome annotations as the complete and partial BUSCO profiles (Table S2**)**.

The function attributed to each predicted protein was based on the results of BlastP (SwissProt) and the InterProScan domain inspection. InterProScan analyses highlighted about 77% of the predicted proteins with at least one IPR domain. Among the top 20 SUPERFAMILY domains, listed in Table S3, the most abundant in all the genomes was SSF52540 (P-loop containing nucleoside triphosphate hydrolase), which is involved in several UniPathways, including chlorophyll or coenzyme A biosynthesis. The other most abundant Superfamilies were: SSF56112 (protein Kinase-like domain), which acts on signalling and regulatory processes in the eukaryotic cell, SSF52058 (Leucine-rich repeat domain, L domain-like), which is related to resistance to pathogens and SSF48371 (Armadillo-type fold), which is involved, inter alia, in defense response and translation factor activity.

Clustering the proteomes (169,465 sequences) of the four genomes and the reference ‘CM334’ with Orthofinder produced a set of 34,192 gene families (plus 8,682 unassigned genes, Fig. 1A), of which 22,017 (including 123,662 genes) were shared. The proteome of ‘CM334’ highlighted 11 genome-specific orthogroups (118 genes), while just one specific orthogroup was found in the ‘Cuneo’ and ‘Tumaticot’ genomes and none in ‘Corno’ and ‘Quadrato’. A total of 193 orthogroups, that include 391 genes among which the transcription factors MED28 and NAP2, were in common between the two blocky types ‘Quadrato’ and ‘Cuneo’ (Fig. 1B). MED proteins in plants perform different functions ranging from development regulation^20^ to biotic and abiotic stresses response^21^ while NAP2 is involved in delayed leaf senescence and enhanced fruit yield and sugar content^22^. A total of 139 orthogroups, that include 344 genes, were also shared between the long-shaped peppers ‘CM334’ and ‘Corno’. The common genes comprise SAUR (Small Auxin UP RNAs) genes, which are known to promote primary growth (lengthening) by inducing cell elongation, increasing the rate of cell division as well as cell wall expansion^23^. In any case, a more in-depth functional characterization of these genes is needed.

### Prediction and annotation of miRNA

A search against miRBase (release 21) led to the prediction of non-redundant miRNAs, ranging from 169 in ‘Tumaticot’ and ‘Corno’ (within 213 and 210 genomic regions respectively) to 175 in ‘Cuneo’ (in 219 genomic regions) and 176 in ‘Quadrato’ (in 222 genomic regions) (Table S4**)**, belonging to 62 (61 for ‘Tumaticot’) miRNA families. The same pipeline was applied to the recently improved ‘CM334’ genome, and resulted in the identification of 170 miRNAs, belonging to 59 families, in 215 genomic regions. It is well known that some miRNA families target known transcription factors related to plant development, morphology, flowering time, as well as response to stress^24–26^. Examples include miR164 and NAC-like proteins following drought or salinity stress^27^, miR160 and *ARF* (Auxin Response Factor), which control auxin-regulated transcription^24^, miR156 and *SQUAMOSA* promoter binding-like proteins which regulates the juvenile-to-adult vegetative transition and the vegetative-to-reproductive transition^24^, miR172 and AP2-like proteins^24^, which regulate plant development and response to stress. The search for genes targeted by the identified miRNAs (psRNATarget search) identified between 736 (‘Corno’) and 761 (‘Tumaticot’) putative miRNA:mRNA duplexes, involving 125–135 unique miRNAs and 275–584 unique transcripts. Almost 87% of genes encoding predicted target transcripts have functional InterPro annotations. The total number of miRNA families involved in miRNA:mRNA interactions varied according to the genotype, ranging from 53 in ‘Tumaticot’ to 58 in ‘Quadrato’ with miR2673, miR172 and miR395 being the top ranked family for all the four genotypes (Table S5). The same search in ‘CM334’ identified 635 putative miRNAs:mRNA duplexes, involving 1119 unique miRNAs and 412 unique transcripts (92% of genes having an InterPro annotation). The putative miRNA-target gene enrichment analysis in each genotype revealed significant enrichment for some GO terms (Table S6). Some of them appeared shared among all genotypes (Table S6), including GO:0009808/GO:0046274 (lignin metabolic/catabolic process) and GO:0003677 (DNA binding).

### Resistance genes

Many plant-pathogen interactions are determined by the presence of resistance (R) genes/alleles, which enable plants to recognize pathogens effectors and subsequently activate effector-triggered immunity (ETI)^28^, followed by a defense response often leading to cell death or a hypersensitive response (HR)^29^. Most intracellular immune receptors in plants belong to the nucleotide-binding site and leucine-rich repeat (NLR, also known as NB-LRR) superfamily^30,31^. The NLR family proteins include two classes on the basis of the presence of a toll and interleukin-1 receptor domain in the N-terminus (TIR-NLR or TNL) or its absence (non-TIR-NLR or non-TNL). Some non-TNL proteins have a coiled-coil motif consisting of CC-NLR (CNL).

The RGAugury pipeline detected between 925 and 943 resistance gene analogues (RGAs), in the four genomes, representing about 2.5% of the total number of predicted genes, while up to 1,600 were found in ‘CM334’. The majority of RGAs were receptor like kinases (RLKs), followed by receptor like proteins (RLP) and nucleotide binding site leucine rich repeat (NBS-LRR), while only few RGAs contain at least one NB-ARC domain (Table 4). Few TNLs in the genomes of the four inbred lines were found, in line with results obtained by^32^, and Barchi *et al*.^19^ in pepper and other Solanaceae species such as eggplant, tomato and potato. Indeed, Kim *et al*.^33^ highlighted that some Asterids contain functional TNLs, whereas others do not, resulting in the identification of only 19 and 13 full length CNLs in sunflower and lettuce respectively, but no full length TNLs. Recently, Acquadro *et al*.^34^ reported that in the *C. cardunculus* genome, the RGAs belong almost exclusively to the RLK/RLP families, while no TNLs and few CNLs were identified. This species-specific RGAs distribution was also observed in *Brassica oleracea, B. rapa, Arabidopsis* and *Teobroma cacao*, where the number of TNL was higher than CNL, while an opposite situation was found for *Populus trichocarpa, Vitis vinifera* and *Medicago truncatula*^35^.

**Table 4:** RGA proteins in the four pepper resequenced genotypes.

Genotype/Species	NBS	CNL	TNL	CN	TN	NL	TX	Others	RLP	RLK	TM-CC
Cuneo	37	92	21	6	8	129	8	4	142	445	42
Tumaticot	30	98	20	7	9	122	6	5	150	450	46
Quadrato	38	89	18	6	9	121	9	5	137	454	42
Cuneo	32	90	23	4	9	116	6	5	145	455	40
CM334	194	150	21	57	12	299	9	7	185	634	40
Heinz 1706 (Tomato)	60	66	22	12	9	81	7	1	72	481	47

Data on RGA in the reference ‘CM334’ genotype as well as in in tomato 'Heinz 1706' are also reported.

We found out clustering of RLKs, RLPs, NBS-encoding and TM-CC genes in some chromosome of the four inbred lines (Table S7**)**, in agreement with classical genetics and analysis from large scale sequencing data in plant genomes^36^. The chr. 3 was the richest in RGAs followed by 12, 1 and 2, while chr. 8 was the poorest. Otherwise, ‘CM334’ was rich in NBS genes mainly in chr. 9, 6 and 5. The majority of RLK genes were found on chr. 2, 3, 7 and 4 while the majority of NBS on chr. 1, 9 and 11.

The alignments of the amino acid sequences and subsequent IQ-TREE analyses generated phylogenetic trees for each RGA class (Figure S1). As expected, resistance genes and orthologs clusters together, although in some taxa one or more orthologs were absent. Interestingly, in ‘CM334’ genome, 105 NBS genes were found to cluster into two main groups while no orthologs were identified in the genomes of the four inbred lines. Besides ‘CM334’ contained two clusters of 124 NL and 74 RLK specific gene. To explore the evolutionary relationships among pepper NLRs, a phylogenetic tree was constructed using the CNL and TNL proteins identified together with 39 known plant resistance (R) proteins. As expected, the TNL and CNL clades branched out, although five predicted CNL genes were nested within the TNL clade. The CNL genes were splitted in 13 subgroups, being G4 and G10 the largest. Subgroups G1 and G3 contain only genes of ‘CM334’ while all the others included RGAs identified in the four inbred lines as well. We could hypothesize that the specific CM334 clusters could be somehow related to their high levels of resistance to diverse pathogens, including *Phytophthora capsici*, pepper mottle virus and root-knot nematodes^12,37,38^, confirming an unequal gene duplication event among subgroups not only at the species level^32^, but also at genotypic level.

CNG-G13 was reported to be particular expanded in potato. Our findings seem to support this hypothesis, as just one gene from ‘CM334’ was found in this CNL subgroup. Interestingly, several putative RGAs were identified and showed missing domains, in line with what was reported by several authors^39–48^. Besides genes belonging to types TN, TX and CN, which might serve as reservoirs for diversity or serve to guard other NLRs from genetic aberration^32^, several NL genes (NBS-LRR lacking coiled-coil or Toll/Interleukin-1 receptor) were found, as observed in *P. trichocarpa, V. vinifera* and *M*. *truncatula*^35^ or *Arachis* spp.^49^. It was also reported that maintaining many NBS/resistance genes has potential fitness costs^50,51^ and it has been suggested that microRNAs are exploited by plants to regulate NBS gene expression^52–56^. Indeed, we found that 19 (in ‘Cuneo’) to 34 (in ‘CM334’) identified RGAs of the CNL, TNL, RLP and RLK classes (Table S7) were putatively targeted by a miRNA, suggesting that this mechanism could also be present in pepper.

### Susceptibility genes

Typically, phytopathogens exploit plants’ susceptibility genes (S-genes) to facilitate their proliferation^18^, and their disruption may interfere with the compatibility between the host and the pathogens and consequently provide broad-spectrum and durable disease resistance^57^.

**Figure 2:**
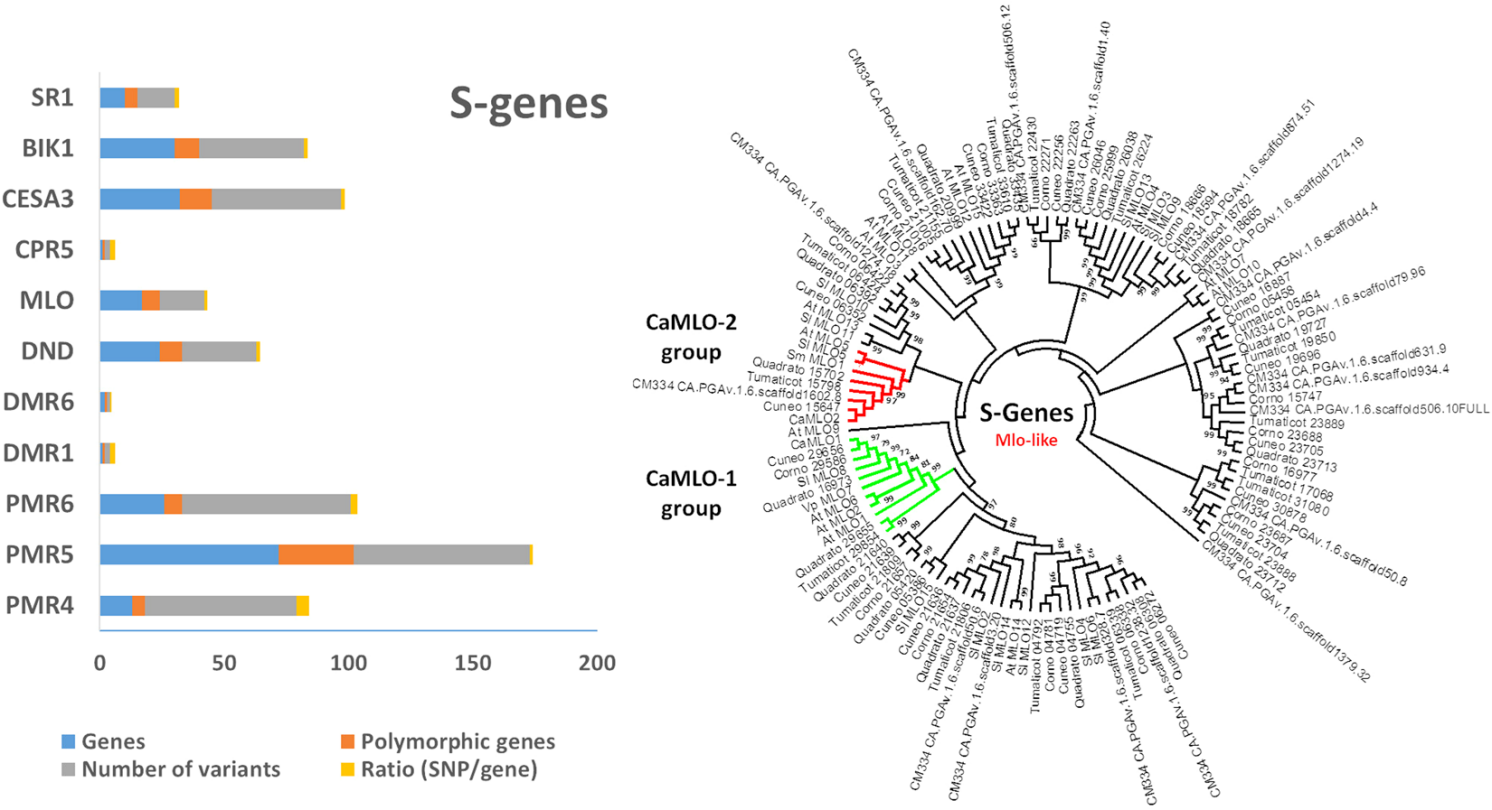
Pepper statistics on S-Genes. Left side: Number of genes, polymorphic genes, variants in S-genes. Right side: Phylogenetic tree of proteins belonging to MLO-like genes from the four genotypes analyzed and considering ‘CM334’ different plant orthologous proteins; *CaMLO*-1 and 2 groups are highlighted in yellow and orange, respectively; bootstrap values were inferred from 100 replicates.

In the genome of the four inbred lines and the reference ‘CM334’, we explored the presence and variants of 11 S-genes (Table 5**)**, which are involved in: (i) basic compatibility, which assists in host recognition and penetration, e.g. *MLO*; (ii) sustained compatibility, which is required for pathogen proliferation and spread, e.g. *CESA3* and (iii) negative regulation of immune signals e.g. *DMR1*^58^. *PMR* genes are involved in cell wall biology where they mediate structure formation and pectin accumulation^59^. Among them, the *PMR5* family was the most represented, totalling from 67 to 69 gene members in the four inbred lines and 72 in CM334’, which were scattered along the genome. Less represented were the families of *PMR6*, whose number ranged from 25 to 27, and *PMR4*, with 13 genes in ‘CM334’, 12 in ‘Quadrato’ and ‘Tumaticot’, 11 in ‘Corno’ and 10 in ‘Cuneo’ **(**Table 5). *MLO* is one of the best-known S genes, which is required for powdery mildew penetration in epidermal cells. It represents a prominent example of robustness in durable pathogen-resistance programs and is conserved throughout monocots and dicots. We identified several *MLO* genes ranging from 16 in ‘Corno’ to 18 in ‘Cuneo’ (Table 5). *MLO*-like genes were found scattered along the genome with 4–5 loci on chr. 2, and 3 in chr. 8. The phylogenetic tree of the *MLO* gene family in the four pepper inbred lines and ‘CM334’ (Fig. 2) highlighted, as expected, the clustering of *MLO* orthologs.

**Table 5:** Number of the S-genes and their variant proportion in the four genotypes.

S-Gene	Cuneo	Quadrato	Tumaticot	Corno	CM334	Polymorphic genes	Number of variants	ratio (SNP/genes)
*PMR4*	10	12	12	11	13	5	61	5.26
*PMR5*	69	68	68	67	72	30	71	1.03
*PMR6*	26	27	27	25	26	7	68	2.60
*DMR1*	1	1	1	1	1	1	2	2.00
*DMR6*	2	2	2	2	2	1	1	0.50
*DND*	22	23	23	23	24	9	30	1.30
*MLO*	18	17	18	16	17	7	18	1.05
*CPR5*	1	1	1	1	1	1	2	2.00
*CESA3*	35	34	36	37	32	13	52	1.49
*BIK1*	25	22	26	25	30	10	42	1.64
*SR1*	9	8	7	8	10	5	15	1.79

*PMR4/5/6* = powdery mildew resistance 4/5/6; *DMR1* = downy mildew resistance 1 (homoserine kinase); *DND* = defence no death (cyclic nucleotide-gated ion channel); *MLO* = mildew resistance locus O; *CPR5* = constitutive expressor of pathogenesis-related gene 5; *CESA3* = cellulose synthase 3; *BIK1* = *Botrytis*–induced kinase 1; *SR1* = signal responsive 1 (calmodulin binding transcription factor).

*DND* are cyclic nucleotide–gated calcium channels genes, which seem to specifically suppress the HR. We identified many *DND* genes ranging from 22 in ‘Cuneo’ to 24 in ‘CM334’ (Table 5), scattered along all the chromosomes except chr. 4. *CESA3* is involved in the pathway of plant cellulose biosynthesis and the homozygous recessive mutant alleles can confer resistance to multiple pathogens, as a result of an increase in planta of abscissic acid, jasmonic acid and ethylene levels^57^. Its copies ranged from 34 to 37 in the four pepper inbred lines and were 32 in ‘CM334’. *BIK 1* encodes a receptor-like cytoplasmic kinase that mediates PTI signalling from multiple pathogen-associated molecular pattern receptors^60^. Its copies ranged from 22 in the ‘Quadrato’ to 30 in the reference ‘CM334’. *SR1* is a transcription factor which binds to the promoter of *EDS1*, a key regulator of plant defense responses, and represses its expression. Thus, its loss-of-function was found to display enhanced disease resistance^61^. Ten copies of the genes were found in ‘CM334’ and from 7 to 9 in the genome of the four inbred lines. The less represented S-genes were *DMR6*, which has been used to trigger broad-spectrum resistance against multiple pathogens and was present in two copies in all the genomes, and the *DMR1* and *CPR5* genes. The formers have been described to confer resistance to downy mildew in Arabidopsis and tomato by accumulating elevated levels of homoserine^59^, while the latter to be involved in cell proliferation and cell death control^62^.

S-genes were analyzed for the presence of functional SNPs (Fig. 2) and high impact variants in both homozygous and heterozygous state (Table S9**)**. The number of deleterious SNPs fixed in homozygosity was two in the *CESA3*, *PMR5* and *MLO* genes and one in the *DND1* and *SR1* genes. Their effect on plant pathogen resistance deserves to be tested in the inbred lines in study. Furthermore, we detected a number of deleterious SNPs in heterozygosity, i.e. two in the *PMR5* genes and one in *PMR4*, *PMR6* and *BIK1* genes. Their effect should be tested after plant selfing with the goal to assess their effect when recessive S-genes are fixed in homozygosity.

### SNP/Indel mining and annotation

**Figure 3:**
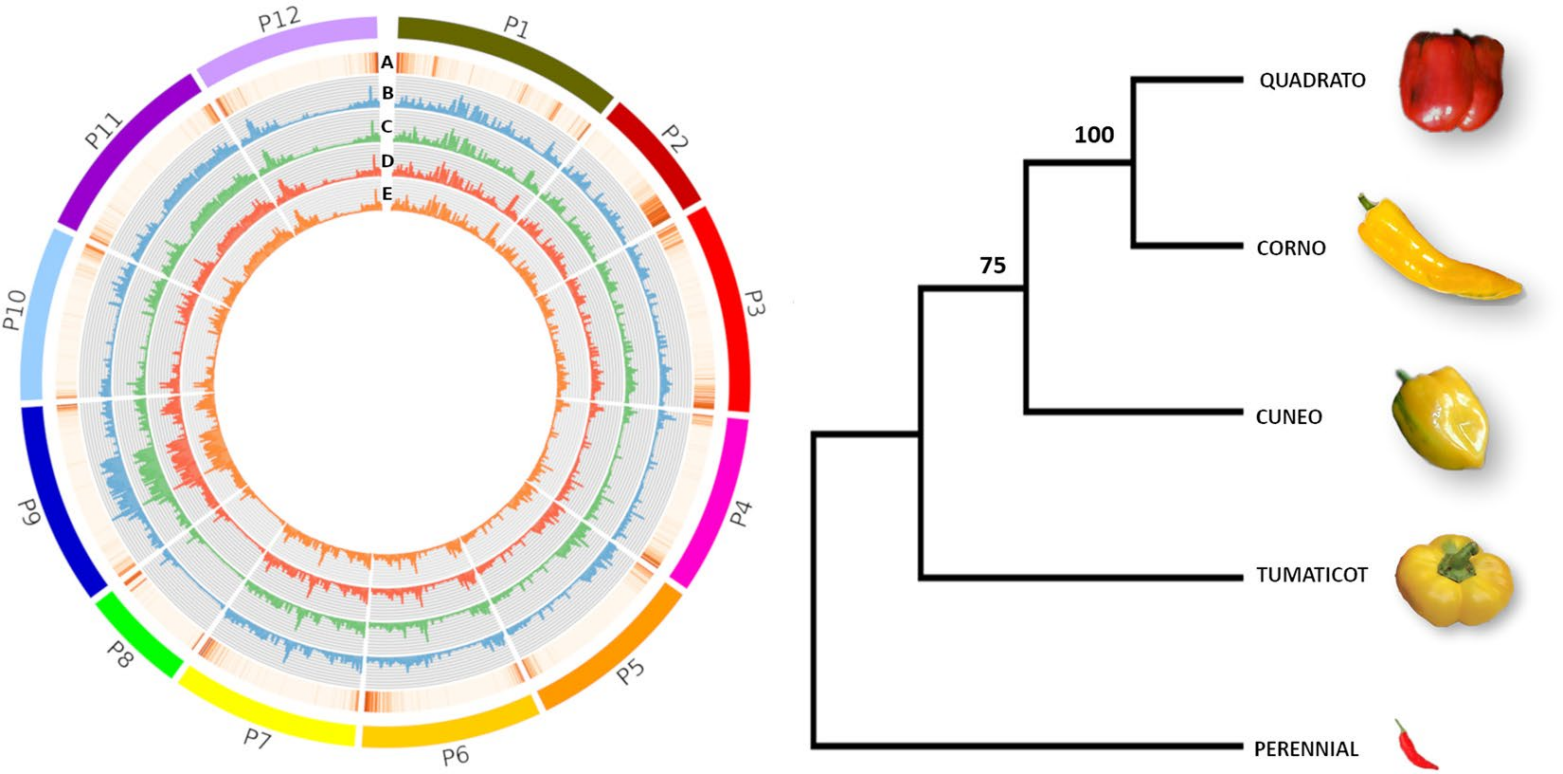
Left side: Diagram showing gene density and SNP distributions: (**A**) Heat map of gene density in the reference genome; 1Mbp histograms of SNP density for ‘Cuneo’ (trace **B**) ‘Tumaticot’ (trace **C**), ‘Quadrato’ (trace **D**), ‘Corno’ (trace **E**). Right side: UPGMA-based dendrogram of the four genotypes taking into account genomic SNPs; ‘Perennial’ was used as outgroup and bootstrap values were inferred from 100 replicates.

The sequence reads of the four inbred lines were aligned back to the reference genome (‘CM334’) and their mapping rate was on average 98,84% **(**Table 2**)**. The number of SNP/Indel variants detected ranged from about 16.33 to 18,08 M, for a total of ~19,63 M non-redundant SNPs. The majority of variants (13,07 M, 66,57%) were shared among the four genotypes, attributable to their high diversity in respect to ‘CM334’, which can be considered as a precursor of modern sweet peppers^17^. The SNP frequency observed at whole genome level was quite similar among the four inbred lines, ranging from 1 SNP/Indel every ~168,5 to 1 every 179,3 bp **(**~0.56–0.59%, Table 6**)**. Some biases were instead recorded in the 12 chromosomes (Table S10), as can be seen from the Circos (Fig. 3**)**. Chr. 9 resulted the most polymorphic in the four inbred lines (one polimorphism every 83.5 bp on average), while chr. 8 was the least polymorphic (one variation every 414 bp). Most of the SNP/Indels identified in the four inbred lines were highly homozygous, as the rate of heterozygosity ranged from 0,098% in ‘Tumaticot’ to 0,196% in ‘Quadrato’ and ‘Corno’ (Table 6**)**, coherently with their inbreeding history^5^. The majority of variants in homozygous state was observed in ‘Cuneo’ and ‘Tumaticot’: this, together with their low heterozygosis level (~0.098% and 0.112%; Table 6), suggest a higher genetic stability compared to the other two inbred lines, confirming a more effective selection carried out over the time by farmers^5,63^. The identified SNP/Indels were used to estimate the genetic relatedness among the four inbred lines (Fig. 3). They did not group according to the fruit shape, as ‘Quadrato’ and ‘Corno’ showed to be closer each other in respect to ‘Cuneo’ and ‘Tumaticot’. This might be explained because ‘Quadrato’ and ‘Corno’ are currently cultivated on larger areas more suited to horticulture and over time have been subject to a greater selection for traits of agronomic interest. Vice versa, ‘Cuneo’ and ‘Tumaticot’ are widespread in more limited and foothills areas and therefore adapted to more niche soil and climatic conditions.

**Table 6:** SNP/Indel (DP > 15) statistics identified in the four pepper genotypes with their relative frequency for each analysed genotype.

Genotype	unfiltered SNP/Indel	SNP/Indel	SNP	Indel	N°SNP/Indel (%)	SNP/Indel in 1000 bp	1 SNP/Indel every (bp)	N° SNP/Indel Homozygosis	SNP/Indel Heterozygosis	Heteroz. (%)
Cuneo	17,678,065	16,649,886	16,033,452	616,434	0.586%	5.86	170.6	13,228,374	3,421,512	0.112%
Quadrato	18,972,695	18,019,108	17,347,941	671,167	0.593%	5.93	168.5	12,019,779	5,999,329	0.196%
Corno	18,941,981	18,076,252	17,389,251	687,001	0.585%	5.85	171.0	12,082,809	5,993,443	0.196%
Tumaticot	17,224,938	16,330,222	15,701,405	628,817	0.558%	5.58	179.3	13,311,815	3,018,407	0.098%

The reference genome (‘CM334’) considered (without N) is of length equal to 3.065.158.452 bp.

About 80% of the SNP/Indel variants were found in intergenic region and just at most 1.8% in exons. The frequency of SNP/Indels in intronic region was 6%, while upstream and downstream gene variants were 12% and 9%, respectively (Table S11**)**. Following the SnpEff analysis focused on coding regions, the majority (63.8%) of variations were non-synonymous (missense), followed by synonymous (silent), while just 2.4% were nonsense. By considering homozygous or heterozygous variants (Table S12), no significant differences in number, among inbred lines, were observed for high effect SNPs in homozygous state (4.65 K on average), while the heterozygous ones widely varied, ranging from about 1.98 K in ‘Tumaticot’ to 3.47 K in ‘Quadrato’ (Table S13). An analogous trend was also observed for the missense mutations, whose total number varied from about 65k in ‘Tumaticot’ to about 84 K in ‘Quadrato’. (Table S13).

### Variants in fruit related genes

The size of the fruit is a key trait for breeding, which is controlled by several genes and multiple interacting biosynthetic pathways^64^. In pepper, a large number of QTLs affecting fruit size and shape have been identified so far in different genetic backgrounds^14,37^, and most of them locate on chr.s 2 and 4. Hill *et al*.^64^ detected in pepper a QTL (*Chaim2.2*), including both the regulatory genes *CaOVATE* and *CaWUS*, which explain up to 17% of the variation in the berry diameter, while in tomato, Frary *et al*.^65^ demonstrated that the *fw2.2* gene explains about 30% of the fruit weight variation.

We analysed variants in 55 genes belonging to categories/pathways controlling berry size and shape (Table S14**)**, and also included in the analysis orthologs of tomato genes involved in the domestication syndrome^66^, i.e.: *fw2.2/CNR* controlling fruit weight, *OVATE* and *SUN* controlling fruit shape, *fas/CLV3* and *lc/WUSCHEL* controlling fruit size and shape. On the whole, most of the genes affecting fruit shape and size (e.g.: *WUS*, *CLV3*, *SUN*,) were found to harbour more mutations than the ones influencing berry weight (e.g.: *fw2.2* and *fw3.2*).

By using ‘CM334’ as reference, the inbred lines in study contained 35 SNPs per gene on average, of which most resided in regulatory regions, but no deleterious variants were spotted (Figure S3**)**. Half of the genes (Figure S2) exhibited more than 20 SNPs in upstream regions (cis-regulatory regions), with a potential role in affecting agronomic traits^67^.

**Figure 4:**
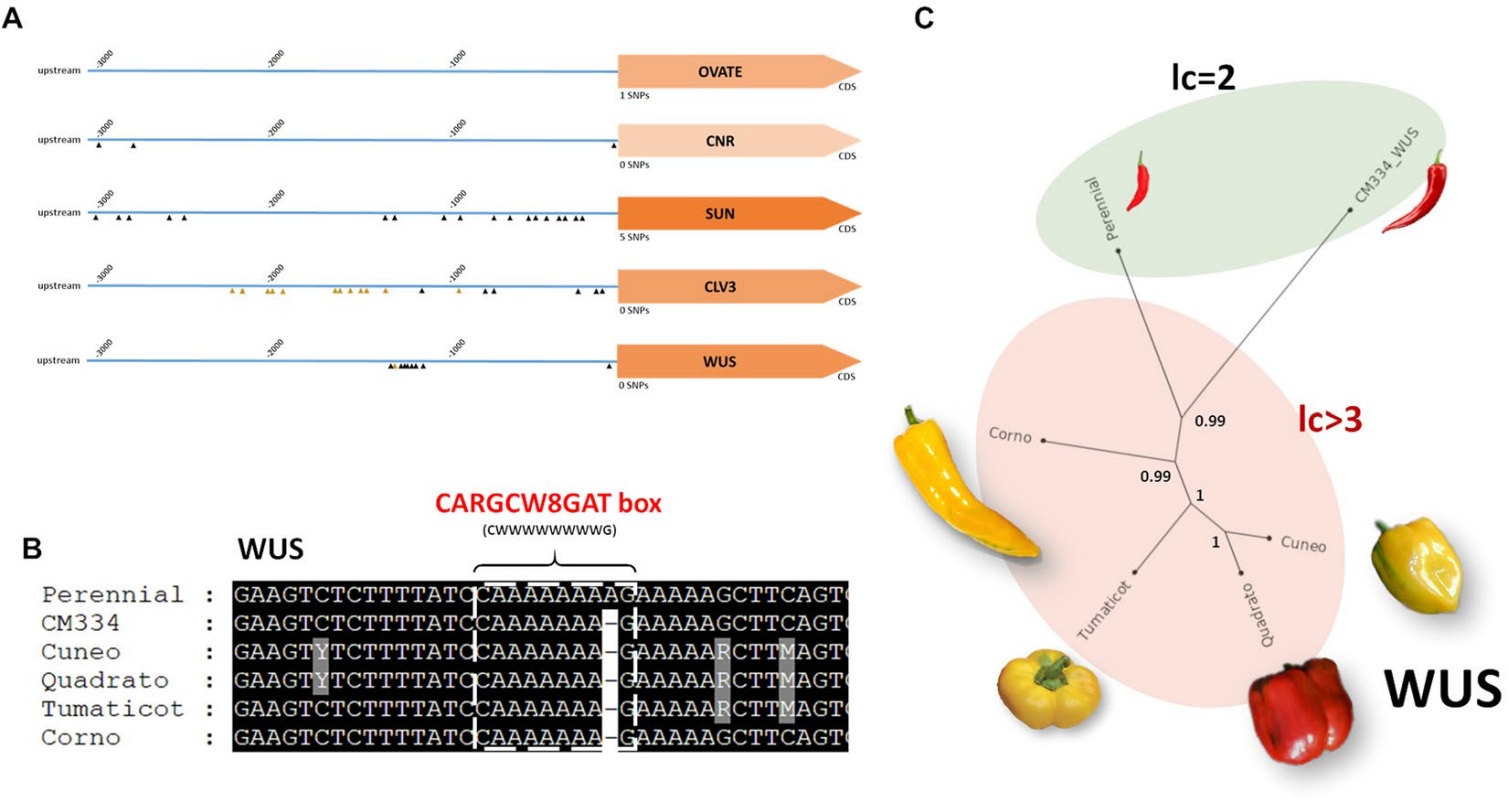
(**A**) SNP distribution in upstream regions of *OVATE*, *CNR*, *SUN*, *CLV3* and *WUS* genes. Triangles show detected SNPs. Orange triangles represent conserved SNPs in elongated vs blocky genotypes. (**B**) Example of one altered cis-element in the WUS gene. (**C**) Genetic tree constructed using information on SNPs present in the WUS promoter, with bootstrap values (100 iterations). “lc” notation means “locule average number”.

Promoters of the four inbred lines (Fig. 4**)** were compared with the ones of ‘CM334’ and ‘Perennial’, which produce very small berries (Table 1). In the 3 kb upstream (Figure S4) region of *SUN*, *WUS* and *CLV3*, consensus variants were spotted in the small-fruited ‘CM334’ and ‘Perennial’ as well as in our four inbred lines, as disclosed by the genetic trees (Fig. 4). In *SUN* and *CLV3* the variants were widespread in the 3 kb upstream the genes, while in *WUS* was highlighted a cluster of 18 SNP in a small region (130–140 bp). Due to the absence of some cis-regulatory elements (**File S1**), those mutations might impact the *WUS* and *CLV3* promoter with phenotipic consequences. As example, a mutated CARGCW8GAT box, a variant of CArG motif with an extended A/T-rich sequence^68^, was observed in the *WUS* promoter of the four inbred lines in study as well as in ‘CM334’ (Fig. 4). Many variants in *CLV3* were found conserved between ‘CM334’ and’Perennial’ in the 1500–2200 bp upstream the gene start codon (Figure S4**)** eliminating some cis-element (CCAAT box, MYb1 box, **File S1)**. Although not functionally validated, the mutations in the *WUS* and *CLV3* promoters of the four inbred lines (Fig. 4) might affect *CLV3* expression and its interaction with *WUS* in the classical *CLAVATA*-*WUSCHEL* stem cell circuit (CLV-WUS) controlling meristem size, as already observed in tomato^69,70^, in which they led to an increased fruit size during domestication. Mutations in *WUS* and *CLV* promoters might be related to the number of fruit lodges, which ranges from 3 to 4 in the four piedmontese inbred lines, while is two in ‘CM334’ (2.28) and ‘Perennial’. However, more complex allelic epistatic interactions as well as other genes might be involved in determining fruit shape, as SNPs in promoter region of many other genes involved in organ size/development were detected (Figure S2**)**. Currently, the effect of mutations in gene regulatory regions is based on the expensive and laborious selection of rare mutations occurring in these regions. However, hereafter, the application of the CRISPR/Cas9 technology on promoters of *WUS* and *CLV* may contribute to shedding light on the impact of the detected variants by modelling different cis-regulatory alleles and providing quantitative variations^67^.

### Detection of presence/absence variants (PAVs)

Gene presence–absence variations (PAVs) within a species can contribute to trait variation and recently Ou *et al*.^71^ published the PAN-genome of cultivated pepper, based on a wide set of cultivars belonging to the four *Capsicum* cultivated species, and applied a gene PAV approach for performing phylogenetic analysis and a genome-wide association study (GWAS)^71^. In our study, of the 875 PAV loci identified, most (654, i.e. about 75.0%) were present only in the ‘CM334’ genome. Among the other 221 PAV loci detected, 78 were absent in one, 43 in two and 100 in three inbred lines **(**Table S15**)**. Of the 100 PAV loci exclusive to a genotype, 39 were spotted in ‘Cuneo’, 42 in ‘Tumaticot’, 11 in ‘Corno’ and 8 in ‘Quadrato’ **(**Table S16**)**. Some of the PAVs related to RGAs seem justify some discrepancies detected in the RGA orthologous clusters (missing of some genes among genotypes, Figure S1), as well as some genotype-specific clusterization of NBS-LRRs, which likely occurred via segmental and/or tandem duplications (Table S6). Among the PAVs missing in a inbred line, two small clusters were detected, of which one chr. 11 (249,24 Mb − 249,25 Mb and 257,90 Mb 257,94 Mb) of ‘Cuneo’ and one in chr. 7 (0,77 Mb - 1,61 Mb) of ‘Tumaticot’ carrying three transcriptional factors (SAP4 Zinc finger A20 and AN1 domain-like) and four resistance RGAs. Among PAVs unique to a genotype, three small clusters were observed: two in ‘Cuneo’ located in chr. 2 (133,78 Mb - 133,81 Mb), and containing three RGAs in a 10k wide region and on chr. 6 (4,48 Mb-4,74 Mb); the third was located on chr. 9 (268,65 Mb-269,32 Mb) of ‘Tumaticot’ carrying four resistance gene analogues (RGAs, Figure S5).

The GO enrichment analysis for the five clusters showed just one over-representation (“nucleotide binding proteins”, GO:0000166) related to the PAVs exclusively present in ‘Tumaticot’.

**Figure 5:**
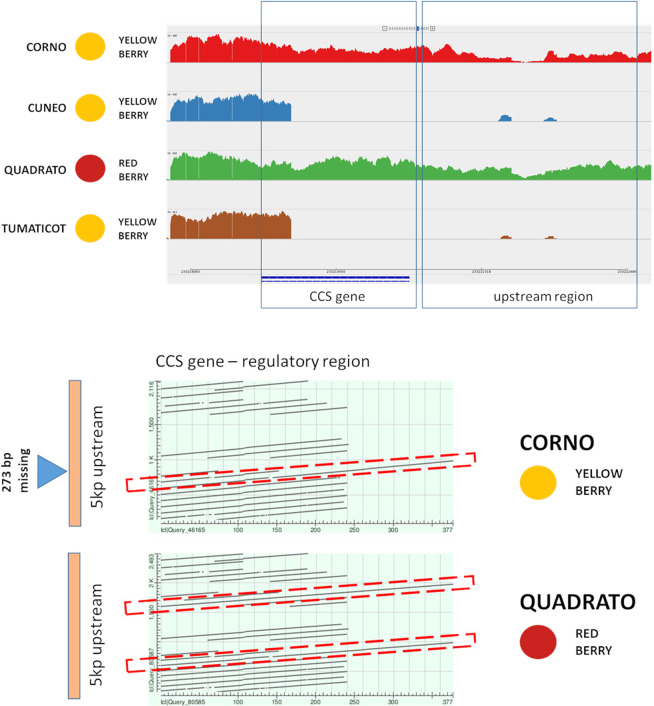
Top side: Presence absence variation in the Locus Y (*CCS*) is shown in the four resequenced pepper genotypes showing the *CCS* gene (in blue, bottom) and 5 kb upstream regions. Bottom side: 5Kb upstream region of the *CCS* gene. Pairwise blast of the 378 bp in the ‘Corno’ versus the 5 kb region of ‘Corno’ (top part) and ‘Quadrato’ (bottom part).

In pepper, capsanthin/capsorubin content is regulated by the capsanthin/capsorubin synthase (*CCS*) gene activity, and other three genes (*PSY*, *LCYB*, *CRTZ*) are responsible for red/orange/yellow pigmentation^72^. Three of the four pepper inbred lines in study (‘Cuneo’, ‘Corno’, and ‘Tumaticot’) produce yellow fruits while one (Quadrato) red fruits **(**Table 1). Since no deleterious SNPs were observed in the four genes involved in the berry pigmentation, we perfomed comparative analyses in order to detect possible PAVs. In two of the yellow-fruited inbred lines (‘Cuneo’ and ‘Tumaticot’, Fig. 5**)** missing reads in the *CCS* gene surrounding the coding sequence and its promoter, and leading to a trunked protein, were spotted (Fig. 5). This was not so in the red fruited ‘Quadrato’, but also in the yellow-fruited ‘Corno’, although the latter lacks of a 378 bp region located in the *CCS* promoter (1200–1300 bp upstream the start codon, Fig. 5). This region was scanned and many regulatory motifs were identified (e.g.: CAATBOX 1-like and myb-like cis-elements, Table S17). Moreover, some differences were also present in the common distal box, such as a triple CAAAT box in ‘Quadrato’, in respect of a double CAAAT box in ‘Corno’, the latter due to a 15 bp deletion. It is known that multiple repeats in a promoter segment can cause transcription factor autoregulation, and in red apples the number of such modules correlates with increased transactivation by a MYB10 protein^73^. A similar behaviour was reported in *C. annuum*, where the *CCS* promoter presents a 3-modular redundant structure^72^, in which a single repeat ensures *CCS* gene expression. By considering the 5 kb region up-stream the *CCS* gene in ‘Quadrato’ and ‘Corno’, the former (red) showed the presence of 2 modules (2 ×378 bp), while the latter (yellow) exhibited only one distal module, with a 15 bp deletion depleting one CCAT box (Fig. 5). As previously reported the transcriptional regulation of *CCS* expression is complex^74^, and we hypothesize that the *CCS* function in ‘Corno’ is thus compromised by its incomplete promoter structure.

## Conclusions

In spite of the high organoleptic and sensorial quality of their products, landraces of horticultural species have been displaced over time from market-driven production due to their lower yields, poorer pest and disease resistance, and often limited postharvest shelf-life in respect to bred commercial varieties and F_1_ hybrids. It is thus crucial to characterize the genetic variation within and between landraces of vegetables, with the goal to actualize their agronomic performances and make them fitting with current agriculture and consumption standards.

Recent advances in high-throughput sequencing technologies makes easily available big amounts of data, which integrated with phenotypic information, enable the identification of genes affecting key agronomic traits. We performed the genome resequencing of four pepper inbred lines selected within as many Italian landraces producing different berry types, ranging from blocky to elongated or sub-spherical. Our resequencing data revealed large genetic variations among them and demonstrate that resequencing provides an efficient way for gathering a large amounts of genomic information, although further analyses and functional studies will serve for practical applications in marker-assisted selection programs.

The identified RGAs provide tools for designing diagnostic markers and identifying quantitative trait loci (QTL) or markers associated with plant disease resistance. The identified susceptibility genes (S-genes), which favour the plant colonization by pathogens, represent ideal target for genomic editing, with the goal to disrupt their function and confer durable resistance to diseases. In addition, the spotted genes related to fruit quality traits may represent target for pepper breeding as well as for understanding the genomic features that distinguish modern from traditional varieties.

## Materials and Methods

### DNA extraction

Seeds were germinated and plantlets cultivated for four weeks in a growth chamber in conditions of darkness/light (8/16 hours) at 25 °C. Subsequently, total genomic DNA was extracted from fresh leaves of each genotype, using the DNA Mini Plant kit (Qiagen, Hilden, Germany). RNAse A was used to remove RNA contamination. DNA quality was checked by 1% (w/v) agarose gel electrophoresis, and its quantity was assessed by Qubit 2.0 based on Qubit dsDNA HS Assay (Thermo Fisher Scientific, Waltham, MA, USA).

### Sequencing, genome assembly and reference-guided reconstruction

A total amount of 1 μg of DNA was used for the construction of four short insert (length 350 bp) genomic libraries (Novogene, Hong Kong), which were sequenced using an Illumina sequencer (Illumina Inc., San Diego, CA, USA) with paired-end chemistry (2×150 bp). Raw reads were cleaned with Scythe (v0.994, https://github.com/vsbuffalo/scythe) for removing contaminant residual adapters and Sickle (v1.33, https://github.com/najoshi/sickle), which allows to remove reads with poor quality ends (Q  <  30). A two-step approach was adopted for assembly. The first one (*de novo*), which generated contigs/scaffolds, was performed with SoapDenovo v2.04^75^ using specific assembly parameters (avg_ins=300; max_rd_len=150; reverse_seq=0; asm_flags=3; rd_len_cutoff=100; rank=1; pair_num_cutoff=3; map_len=32). The second one (reference-guided), which integrates the contigs/scaffolds into large pseudomolecules corresponding to the chromosomes, was performed using the Chromosomer (v 0.1.4a) tool^76^, with default parameters. In the first one, a k-mer set (wide range: 51, 61, 71, 81 and 91) was preliminarily tested to identify the best k-mer range. Then, a second series of k-mers (e.g.: narrow range, 53–69), which revealed the best assembly performances, was scanned. Metrics for assessing the quality of a genome assembly (e.g.: N_50_, contig/scaffold number/size/length, genome length) were assessed using the perl script Assemblathon_stats.pl (https://github.com/ucdavis-bioinformatics/assemblathon2-analysis). GapCloser v1.12 (https://sourceforge.net/projects/soapdenovo2/files/GapCloser) was used to fill in the gap emerging in the assembly/scaffolding process. Finally, only the contigs/scaffolds with a length exceeding 500 bp were taken into consideration for genome reconstruction. The genome reconstruction of each genotype was performed using the Chromosomer^76^ pipeline taking into consideration the scaffolds/contig previously obtained and the sequence of the *Capsicum annuum* genome (‘CM334’)^15^ as a guide. Chromosomer uses two parameters influencing the assembly process. The first represents the alignment score threshold (r = 1), which is used to distinguish between anchored and unlocated fragments. If the ratio of the scores of the two fragment alignments with the highest score exceeds the threshold, the fragment is considered anchored, otherwise it will not be positionable and excluded from subsequent analyzes. The alignment score threshold must be a positive number greater than one. The second parameter is the average size of the sequenced fragments, which is used to insert gaps in regions not covered by fragments to be anchored. Raw data from ‘CM334’ and ‘Perennial’ genotypes were downloaded from NCBI (PRJNA223222 and PRJNA298503).

### Structural and functional annotation

Each genotype was masked using RepeatMasker^77^ v4.1.0 using a combination of homology-based and *de novo* approaches. A species specific repeats library was constructed following the Repeat Library Construction Advanced pipeline (http://weatherby.genetics.utah.edu/MAKER/wiki/index.php/Repeat_Library_Construction-Advanced) which requires the use of mite hunter^78^, LTRdigest^79^, LTR_harvest^80^ (available in genome tools^81^ v1.5.10) and Repeatmodeler^82^ v1.0.11. The new library was then combined with Repbase-viridiplantae^83^ to identify TEs. TEs were classified into two main classes (typical of plant genomes): Class I (retrotransposon elements) and Class II (DNA transposons). Gene prediction was performed using Maker-P^84^ v2.31.08. Augustus^85^ v3.3.2 Hidden Markov Models and SNAP^86^ gene prediction algorithms were combined with transcripts and proteins alignments as evidence to support prediction. All predicted gene models were filtered and only the ones with an AED ≤ 0.35 were maintained. AED measures the concordance of a gene predicted with aligned transcripts, mRNA-seq and protein homology data. AED scores range from 0 and 1, where 0 indicates perfect concordance between evidence and gene prediction, while 1 absence of concordance.

For each predicted gene, the gene function was assigned by a BlastP^87^ search against the Uniprot/Swissprot Viridiplantae database^88^, using the default parameters, with the exception of the e-value (<1e-5).

To measure the quality and completeness of the predicted proteomes, a quantitative assessment was carried out based on evolutionary informed expectations of gene content known as Benchmarking Universal Single-Copy Orthologs (BUSCO^89^ v3.0.2., *Embryophyta* odb 10).

The sequences of the predicted proteins were also noted using InterproScan5^90^ compared to all the available databases (ProSitePro les-20.119, PANTHER-10.0, Coils-2.2.1, PIRSF-3.01, Hamap-201511.02, Pfam-29.0, ProSitePatterns - 20.119, SUPERFAMILY-1.75, ProDom-2006.1, SMART-7.1, Gene3D-3.5.0 and TIGRFAM-15.0)^91–101^. Data obtained from the four proteomes were illustrated in a Venn diagram constructed with Interactivenn^102^. Then, GOfeat^103^ was used to identify the enrichment of GO terms for specific gene clusters. Some candidate genes were specifically analyzed in the four resequenced pepper inbred lines. To the scope, protein sequences of the orthologous genes (from pepper, tomato, *Arabidopsis*) and involved in specific traits/pathways (e.g.: fruit shape, colour, S-genes) were downloaded from NCBI, and used to retrieve, via BlastP, putative orthologous proteins in the proteomes of ‘Corno’, ‘Quadrato’, ‘Tumaticot’ and ‘Cuneo’.

### Prediction and annotation of miRNA

The MIReNA v2.0 software^104^ was used for the identification of high confidence miRNA-coding sequences (miRBase release 21^105^) in each pseudomolecule and chr. 0 of all the analyzed inbred lines. A homology search was conducted with known miRNAs from a group of plants and algae species, which included: *Solanum lycopersicum, Solanum tuberosum, Nicotiana tabacum, Vitis vinifera, Arabidopsis thaliana, Oryza sativa, Populus trichocarpa, Medicago trunculata, Zea mays, Picea abies, Triticum aestivum, Physcomitrella patens, Chlamydomonas reinhardtii*. MIReNA was run with default parameters and the maximum number of allowed mismatches between known miRNAs and putative miRNAs was set to 10. For each inbred line, miRNA identified were named based on the miRNA family with the addition of the name of the genotype (‘Corno’, ‘Cuneo’, ‘Quadrato’, ‘Tumaticot’). psRNATarget^106^ (2017 update) was applied to identify the targets of the identified pepper miRNA on the predicted CDSs. Results were parsed to keep only those targets having expectation <of 2.5. GO term enrichment of target sequences for each line was carried out with AGRIGOv2^107^ to find out a representative subset of the GO terms previously identified with the Interproscan pipeline. AGRIGOv2 cross comparison of SEA (SEACOMPARE) was used to identify common and different enrichment GO terms between the genotypes showing GO terms enrichment.

### Resistance genes analogs (RGA)

Candidate resistance genes were identified using RGAugury^108^ for all the four inbred lines in study as well as for the reference line 'CM334'. The pipeline first identifies RGA-related protein domains and motifs, namely nucleotide binding site (NB-ARC), leucine rich repeat (LRR), transmembrane (TM), serine/threonine and tyrosine kinase (STTK), lysine motif (LysM), coiled-coil (CC) and Toll/Interleukin-1 receptor (TIR). RGA candidates were identified and classified into four major families based on the presence of combinations of these RGA domains and motifs: NBS-encoding, TM-CC, and membrane associated RLP and RLK. The NBS-encoding gene family members are further divided into several subgroups according to their domain architecture, namely NBS (NBS domain), CNL (CC-NBS-LRR domains), TNL (TIR-NBS-LRR), TN (TIR-NBS), CN (CC-NBS), NL (NBS-LRR), TX (TIR-unknown domain) and other. To compare the RGAs within and among the four inbred lines in study and the reference line ‘CM334’, we divided the RGAs identified, following RGAugury analyses, in 6 separated groups: RLP (Receptor Like Proteins), RLK (Receptor like Kinases), NBS, CNL, NL and TM-CC. Furthermore, NLR proteins (CNL and TNL) in Solanaceae together with known R proteins from *Arabidopsis* were used. The multiple alignments were performed using MAFFT^109^ v7.450 with the following parameters: –ep 0 –reorder –maxiterate 1000 –genafpair. Genetic relationships were described by constructing a phylogenetic tree by maximum likelihood by using the IQ-TREE software^110^ v.1.6.12; branch supports were obtained with the ultrafast bootstrap^111^ with 1000 replicates. Trees were visualized using interactive Tree of Life (iTOL) v3^112^. To identify the number of RGAs per chromosome across all the 5 genotypes, as well as the presence of clusters, coordinates of the genes belonging to the classes LP (Receptor Like Proteins), RLK (Receptor like Kinases), NBS, CNL, NL and TM-CC were extracted and BEDTools^113^ intersected using genome windows of 1 Mb to count the number of genes falling into these regions.

### Susceptibility genes

A preliminary BLASTP analysis allowed to identify the possible orthologos for susceptibility genes, using information from several plant species (Table S8**)**, considering as a preferential choice criterion the e-value (range 0–1e^−10^), the percentage of similarity and the query coverage. Since many genes were present in multi-gene family, filtering criteria were varied each time and previous functional annotations were used to filter out wrong candidates. Multiple sequence alignments and phylogenetic analyses were carried out using Clustal Omega^114^. Phylogenetic trees were generated for candidate S-genes families using the neighbor-joining (NJ) algorithm. A confidence level was established for each node by performing a bootstrap analysis with 100 iterations. Trees were plotted using Figtree graphical viewer^115^.

### SNP calling and variant annotation

The sequences were mapped to the reference genome of the ‘CM334'^15^ line using the Burrows-Wheeler Aligner (BWA, v0.7.17) program and the ‘mem’ command with the default parameters^116^. BAM files were processed and use for the SNP calling using with Samtools (v1.6)^117^ mpileup with default parameters except for i) minimum mapping quality (Q = 20) and filtering out multimapping events (-q > 1). A vcf (variant call format) file was produced and was subsequently used to construct a tree diagram using Tassel v4.0^118^. SNP/Indels were counted and analyzed using custom bash scripts. The estimation of the heterozygous level of each genome was calculated by considering, per each inbred line, the ratio between the number of SNP/Indels (called in heterozygous state) and the size of the ‘CM334’ genome, deprived of Ns (3,065,158,452 bp). The identified genomic variants were analyzed with the SnpEff v4.3 program^119^, to infer their functional annotation and any potential deleterious effect on protein structure. The effect of each SNP/Indel was classified into four of classes of effects: 1) modifier effect, as variants located outside genes (non-transcribed regions or introns); 2) low effect, as synonymous variants in coding regions; 3) moderate effect, as variants altering the aminoacidic sequence and 4) high effect, as variants changing frameshift thereby introducing/eliminating stop codons or modifying splice sites. Upstream (3 kb) gene regions were searched for the presence/absence of cis-regulatory elements using PLACE (www.dna.affrc.go.jp/PLACE), a database which collect plant cis-acting regulatory DNA elements.

### Identification and characterization of PAV genes

Samtools^117^ was used to generate a file containing the number of reads mapping on the reference genome. The number of reads that mapped at each gene location for every pepper inbred line were extracted and normalized by the total number of reads mapping to the whole reference genome (‘CM334’) for each inbred line. Parameters were set up to spot unmapped regions, as follows: samtools view -c -F 4 -q 1 file.sort.bam. To identify putative PAV genes, all genes with less than six mapped reads from at least one inbred line and more than 29 mapped reads from at least another inbred line were selected. The list of candidate PAV genes obtained were then described using the available ‘CM334’ functional and structural annotation.

## Supplementary information

Supplementary File S1.

Supplementary Figures.

Supplementary Tables.
