# Supplementary material for: Whole genome resequencing of four Italian sweet pepper landraces provides insights on sequence variation in genes of agronomic value
Source: Sci Rep. 2020 Jun 8;10:9189. doi: 10.1038/s41598-020-66053-2 (PMC7280500; doi:10.1038/s41598-020-66053-2)
Supplement: Supplementary file 1 — Supplementary File S1. [file 41598_2020_66053_MOESM1_ESM.zip › cm334_WUS_PLACE.pdf]

New PLACE

A Database of Plant Cis-acting Regulatory DNA Elements

```
Tue Jul 23 20:45:50 JST 2019

TTAACATAAATTCTCATTTTTACCCTTGAAGTCTCTTTTATCCAAAAAAAGAAAAAGCTTCAGTCTCTTTTACTTGTCAAATCATAAGGGTAATTTTGGAAAAAAAATTCAACGATTCATT


RESULTS OF YOUR SIGNAL SCAN SEARCH REQUEST

This result is the output of the new signal scan program which was completely rewritten
from a scratch by Akio Miyao ($Id: 649.pl,v 1.11 2016/04/20 08:43:39 miyao Exp $).

The original program of signal scan was reported in
Prestridge, D.S. (1991) SIGNAL SCAN: A computer program that scans DNA sequences for
eukaryotic transcriptional elements. CABIOS 7, 203-206.



191 base pairs

 (+) = Current Strand
 (-) = Opposite Strand

1      TTAACATAAATTCTCATTTTTACCCTTGAAGTCTCTTTTATCCAAAAAAA
                   (+) INRNTPSADB S000395 13 YTCANTYY
                      (+) SEF4MOTIFGM7S S000103 16 RTTTTTR
                         (-) GT1CONSENSUS S000198 19 GRWAAW
                                     (-) SURECOREATSULTR11 S000499 31 GAGAC
                                       (+) NODCON2GM S000462 33 CTCTT
                                       (+) OSE2ROOTNODULE S000468 33 CTCTT
                                         (-) DOFCOREZM S000265 35 AAAG
                                           (-) GT1CONSENSUS S000198 37 GRWAAW
                                            (-) IBOXCORE S000199 38 GATAA
                                            (+) SREATMSD S000470 38 TTATCC
                                             (-) GATABOX S000039 39 GATA
                                             (-) MYBST1 S000180 39 GGATA
                                             (+) TATCCAOSAMY S000403 39 TATCCA
                                                      (+) DOFCOREZM S000265 48 AAAG
                                                        (+) POLLEN1LELAT52 S000245 50 AGAAA

51     GAAAAAGCTTCAGTCTCTTTTACTTGTCAAATCATAAGGGTAATTTTGGA
       (+) GT1CONSENSUS S000198 51 GRWAAW
       (+) GT1GMSCAM4 S000453 51 GAAAAA
          (+) DOFCOREZM S000265 54 AAAG
               (+) INRNTPSADB S000395 59 YTCANTYY
                   (-) SURECOREATSULTR11 S000499 63 GAGAC
                     (+) NODCON2GM S000462 65 CTCTT
                     (+) OSE2ROOTNODULE S000468 65 CTCTT
                       (-) DOFCOREZM S000265 67 AAAG
                           (+) CACTFTPPCA1 S000449 71 YACT
                               (+) BIHD1OS S000498 75 TGTCA
                                (-) WBOXATNPR1 S000390 76 TTGAC
                                (-) WRKY71OS S000447 76 TGAC
                                    (-) ARR1AT S000454 80 NGATT
                                             (+) GT1CONSENSUS S000198 89 GRWAAW
                                                      (-) PYRIMIDINEBOXHVEPB1 S000298 98 TTTTTTCC
                                                      (+) GT1CONSENSUS S000198 98 GRWAAW
                                                       (+) GT1CONSENSUS S000198 99 GRWAAW
                                                       (+) GT1GMSCAM4 S000453 99 GAAAAA

101    AAAAAATTCAACGATTCATTTATTTTGAAACACCAATAAATACTCCAACA
                  (+) ARR1AT S000454 112 NGATT
                         (-) POLASIG1 S000080 119 AATAAA
                          (+) TATABOX5 S000203 120 TTATTT
                                       (+) CCAATBOX1 S000030 133 CCAAT
                                        (+) CAATBOX1 S000028 134 CAAT
                                         (+) POLASIG1 S000080 135 AATAAA
                                               (+) CACTFTPPCA1 S000449 141 YACT
                                                    (+) RAV1AAT S000314 146 CAACA
                                                       (+) CAATBOX1 S000028 149 CAAT

151    ATTCATTTATTCCGAAATGGAGGGAGTATTTCTTTTAGCGC
            (-) POLASIG1 S000080 156 AATAAA
                  (+) LTRE1HVBLT49 S000250 162 CCGAAA
                               (-) CACTFTPPCA1 S000449 175 YACT
                                   (-) POLLEN1LELAT52 S000245 179 AGAAA
                                      (-) DOFCOREZM S000265 182 AAAG


    Factor or Site Name      Loc.(Str.)      Signal Sequence       SITE #
```

INRNTPSADB	13	(+)	YTCANTYY	S000395
SEF4MOTIFGM7S	16	(+)	RTTTTTR	S000103
GT1CONSENSUS	19	(-)	GRWAAW	S000198
SURECOREATSULTR11	31	(-)	GAGAC	S000499
NODCON2GM	33	(+)	CTCTT	S000462
OSE2ROOTNODULE	33	(+)	CTCTT	S000468
DOFCOREZM	35	(-)	AAAG	S000265
GT1CONSENSUS	37	(-)	GRWAAW	S000198
IBOXCORE	38	(-)	GATAA	S000199
SREATMSD	38	(+)	TTATCC	S000470
GATABOX	39	(-)	GATA	S000039
MYBST1	39	(-)	GGATA	S000180
TATCCAOSAMY	39	(+)	TATCCA	S000403
DOFCOREZM	48	(+)	AAAG	S000265
POLLEN1LELAT52	50	(+)	AGAAA	S000245
GT1CONSENSUS	51	(+)	GRWAAW	S000198
GT1GMSCAM4	51	(+)	GAAAAA	S000453
DOFCOREZM	54	(+)	AAAG	S000265
INRNTPSADB	59	(+)	YTCANTYY	S000395
SURECOREATSULTR11	63	(-)	GAGAC	S000499
NODCON2GM	65	(+)	CTCTT	S000462
OSE2ROOTNODULE	65	(+)	CTCTT	S000468
DOFCOREZM	67	(-)	AAAG	S000265
CACTFTPPCA1	71	(+)	YACT	S000449
BIHD1OS	75	(+)	TGTCA	S000498
WBOXATNPR1	76	(-)	TTGAC	S000390
WRKY71OS	76	(-)	TGAC	S000447
ARR1AT	80	(-)	NGATT	S000454
GT1CONSENSUS	89	(+)	GRWAAW	S000198
PYRIMIDINEBOXHVEPB1	98	(-)	TTTTTTCC	S000298
GT1CONSENSUS	98	(+)	GRWAAW	S000198
GT1CONSENSUS	99	(+)	GRWAAW	S000198
GT1GMSCAM4	99	(+)	GAAAAA	S000453
ARR1AT	112	(+)	NGATT	S000454
POLASIG1	119	(-)	AATAAA	S000080
TATABOX5	120	(+)	TTATTT	S000203
CCAATBOX1	133	(+)	CCAAT	S000030
CAATBOX1	134	(+)	CAAT	S000028
POLASIG1	135	(+)	AATAAA	S000080
CACTFTPPCA1	141	(+)	YACT	S000449
RAV1AAT	146	(+)	CAACA	S000314
CAATBOX1	149	(+)	CAAT	S000028
POLASIG1	156	(-)	AATAAA	S000080
LTRE1HVBLT49	162	(+)	CCGAAA	S000250
CACTFTPPCA1	175	(-)	YACT	S000449
POLLEN1LELAT52	179	(-)	AGAAA	S000245
DOFCOREZM	182	(-)	AAAG	S000265

//

©2011-2019 National Agriculture and Food Research Organization 1077551
