# Supplementary material for: Whole genome resequencing of four Italian sweet pepper landraces provides insights on sequence variation in genes of agronomic value
Source: Sci Rep. 2020 Jun 8;10:9189. doi: 10.1038/s41598-020-66053-2 (PMC7280500; doi:10.1038/s41598-020-66053-2)
Supplement: Supplementary file 1 — Supplementary File S1. [file 41598_2020_66053_MOESM1_ESM.zip › corno_CLV3_PLACE_plus_boxes.pdf]

New PLACE

A Database of Plant Cis-acting Regulatory DNA Elements

Fri Jul 26 18:23:55 JST 2019

```
CTCACTTTTGTCTAGAAAGTAAGTAAAAAATATACTTATATGATCCTTAAAGAATCAACA
GATTTTACTATTTGCAGTAAAAGAGCCAGTCACATTTGGCAAATTTAATTAAAGACAAAT
TCTGTGTAATTTTGACTAGGAGTTAGGGCCGCGCATTCTTATTAGGGGATTCTTTGTTGG
AATATAACTTACGATACCTGATGGTAGTTTAACCAAAAAATATGTATATATATATATATA
TATATATTAGGGGATTCAAAGAGGATCAASCTAGAATGTATGTGAAATATAATGAGATTC
AAATTTGCGATCTACTTCAAATATAAACACAACAATTTATTTATATCATATTTGACACAG
TATAATTTTCTGATAAAAGAGATCGATCCTGTTAAACATCCTTCATTATTGTAGATCCGC
CCCCATTTCCAATTGAGAAAGAGGGAACAAGCAAATGGGGGTGGATTTTACTAAATAATT
TAAGCCATGTACTCCATCTCAATTGTGGAAGTATTCCACAAACGAACTTTTCTTGAGTTG
ATCTCTCAGATAGTCAGTATTTCTAATAGTCGATATTTCAGAAAATCACTTGTTTTGTTG
AACAAAAAATTTGACTTAGAAAAAATGACTTTAGAGATAATAACTAAATCAAACCCTAAA
TTTTTCTCTAAGGACCCCAAATTACCTATGACATTGTGATTTGTGATGGAAACGTTGGTG
CGGAATTCTTGGATTCCTAGTATTTTGTTTTGTTAGGGTAACATTACAACAATAACAACA
TATTCAGTGTATGGTAAAGTGTACGCAGTCCATCCCGACCACTACCTCAGATGAAGTAGA
GAGGTTGTTTCCGATAGACCCCTCGGCTCATGTTWGTGTAACATTGACAATAGAAATTCC
TCTTTCCCTTCATGGTATTTTCTCCTCCTTTTCTTCTCTACTAGCATTATCACTAGTAAT
AAAAAGGCTATGAAGAGAGACTAGAATTCCTTTTCATGGGTAATAACTAACATAAGGAGT
AAAGTAGCCTCTTGGACTCATCTCTTTTATTTTATTTTCTTTTGCCTTTCGGTGCTCTAT
CTTCGACTTTTTCTGACCATTGTTCAGTCACACAGAAAAGTCAGTAAACTAAGAAAATGA
CATGACCACAACTAAAATCATGAAGTACATAATATGAGAGTAAGCAAATTCAAATATCTT
TTAAAAAAAAAAAAAATCAAGAGAAAAAACAAATTCAACGTGTGATATCCAAAGTAAATT
TTCTGATGTAATCACTCTAGCATCATCATTTTATTTTGTCTCTTCAAATCAAATAACGTA
ACTACATACATTATATTATATAAATGGAGGAAAAAGATTGATAAAAGAAAAATAATTTAT
TAGTACACACTAATAACAATGAAGAGGAAAAAATTAAAGCAAAATATATATGTTGTGACT
TGTGAGAGGATTATGTCAATTATCCAGTGGACAAATGCATACTCCTATAACCTACTTATT
TTATTTTCAGAAAAAAGAATGTGATGGTTTCAAGATTAGTACAATTATTTATGATCAGTG
CAGTGCAGTAGTAGAACCTGATGAGGATCGACCAATAAATTGGAAGAGGAAATAAAAGGA
AAATGATAACCAAATTATGAGGAATAATTAACGAACTTGTTAAATGGTGGACTAATAATT
ATTGGAATCCTTAAAAAAATATTATATTCACAAGAAACCCTAGTCCTATCCTATGTATAG
AGATCTAGTTCTATAAATATTAAATTAAATATTTAATTTAAAGCGTGAGTATTATTTAAT
TTCTGCCTAGGTACTTGTTGGATAGGGTTTGAACAAAATTAACTGGATCTGAACTAAAAT
CATATTTACTTCCATTAACCTTCAAAAATCAATCAAGGAAAAAAGAGAAAACAGTGAAAC
ACTTTGTTTTAAATTATTTGAAAATTAGCATAAAATATGACAATTTTCTAACACTTTGAA
GCAAAATTTCTAGAAACATCACATCTTTACGGTTGATTTATCAAATGGTCACTCAAGTAA
TATATAGTTATTATCTCAATTTGTTATCTTTCTTTTTTATTACAATCTTTTCCAACAAAG
AAAACAATTTTCCGAGAAAATAACTACTATTAATTGAATGACCATCTGAAAAATCATCTC
GGCATCTTTCTAACCCTATCTTATGGCTTCTACTAAAAAGAAAAAGAAGACTTCGGTTTA
ATTTCACAAGATTTGCTTTCAAAAAATCTAGAAAAAAGTGGAACAAAATTAAACTTTGAA
CTTGTATTCATCATCATAGACAAAATGAGAGCTCAGAAAAATGGCTCTTAATTTCTTCAA
ACGATTCGATCATGCATTTGAAAACCAGCTTAGCATATATAAAGGGTTTTAAAATTTTTG
AAGGAGATGGATGAAAAAATTAAAATAAAAGAAGTGTACCGCAAGAATTGTGTTGCAGAA
GTTGCAATGAACATAGCAAACACGTTCCAAGGAAGATGAATATGTCATGTCTAGTGACAT
TTTTCTTAAAGGTGTTTTTCTTTTCTTTTTCTTTTTTTAGCCCTGGAATCACGGATCGAA
GAAGAGACGGATCAACAAGGAGCTGTGAACTGA
```

RESULTS OF YOUR SIGNAL SCAN SEARCH REQUEST

This result is the output of the new signal scan program which was completely rewritten from a scratch by Akio Miyao ($Id: 649.pl,v 1.11 2016/04/20 08:43:39 miyao Exp $).

The original program of signal scan was reported in
Prestridge, D.S. (1991) SIGNAL SCAN: A computer program that scans DNA sequences for eukaryotic transcriptional elements. CABIOS 7, 203-206.

2656 base pairs

(+) = Current Strand
(-) = Opposite Strand

1 CTCACTTTTGTCTAGAAAGTAAGTAAAAAATATACTTATATGATCCTTAA
(+) INRNTPSADB S000395 1 YTCANTYY
(-) GTGANTG10 S000378 2 GTGA
(+) CACTFTPPCA1 S000449 3 YACT
(-) DOFCOREZM S000265 5 AAAG
(+) POLLEN1LELAT52 S000245 14 AGAAA
(+) DOFCOREZM S000265 16 AAAG
(-) CACTFTPPCA1 S000449 18 YACT
(-) CACTFTPPCA1 S000449 22 YACT
(-) ROOTMOTIFTAPOX1 S000098 29 ATATT
(+) CACTFTPPCA1 S000449 33 YACT
(+) TAAAGSTKST1 S000387 48 TAAAG
(+) DOFCOREZM S000265 49 AAAG

51 AGAATCAACANGATTTTACTATTTGCAGTAAAAGAGCCAGTCACATTTGG
(-) ARR1AT S000454 53 NGATT
(+) RAV1AAT S000314 56 CAACA
(+) SP8BFIBSP8BIB S000184 67 TACTATT
(+) CACTFTPPCA1 S000449 67 YACT
(-) CACTFTPPCA1 S000449 77 YACT
(+) DOFCOREZM S000265 81 AAAG
(-) NODCON2GM S000462 82 CTCTT
(-) OSE2ROOTNODULE S000468 82 CTCTT
(-) WBOXHVISO1 S000442 89 TGACT
(-) WBOXNTERF3 S000457 89 TGACY
(-) WRKY71OS S000447 90 TGAC
(-) GTGANTG10 S000378 91 GTGA
(-) EBOXBNNAPA S000144 94 CANNTG
(-) MYCCONSENSUSAT S000407 94 CANNTG
(+) EBOXBNNAPA S000144 94 CANNTG
(+) MYCCONSENSUSAT S000407 94 CANNTG

101 CAAATTTAATTAAAGACAAATNTCTGTGTAATTTTGACTAGGAGTTAGGG
(-) POLASIG2 S000081 105 AATTAAA
(+) POLASIG2 S000081 108 AATTAAA
(+) TAAAGSTKST1 S000387 111 TAAAG
(+) DOFCOREZM S000265 112 AAAG
(+) WBBOXPCWRKY1 S000310 133 TTTGACY
(+) WBOXATNPR1 S000390 134 TTGAC
(+) WBOXHVISO1 S000442 135 TGACT
(+) WRKY71OS S000447 135 TGAC
(+) WBOXNTERF3 S000457 135 TGACY
(+) SORLIP2AT S000483 148 GGGCC

151 CCGCGCATTCTTATTAGGGGATTCTTTGTTGGNAATATAACTTACGATAC
(-) CGCGBOXAT S000501 151 VCGCGB
(+) CGCGBOXAT S000501 151 VCGCGB
(+) CPBCSPOR S000491 162 TATTAG
(+) ARR1AT S000454 169 NGATT
(-) XYLAT S000510 172 ACAAAGAA
(-) DOFCOREZM S000265 174 AAAG
(-) RAV1AAT S000314 177 CAACA
(-) ROOTMOTIFTAPOX1 S000098 184 ATATT
(+) GATABOX S000039 196 GATA

201 CTGATGGTAGTTTAACCAAAAAATATGTATATATATATATATANTATATA
(+) S1FBOXSORPS1L21 S000223 204 ATGGTA
(-) GT1CORE S000125 212 GGTTAA
(+) MYB1AT S000408 213 WAACCA
(+) REALPHALGLHCB21 S000362 214 AACCAA
(-) ROOTMOTIFTAPOX1 S000098 222 ATATT
(+) SORLREP3AT S000488 226 TGTATATAT
(+) ROOTMOTIFTAPOX1 S000098 248 ATATT

```
                                                          (+) CPBCSPOR S000491 249 TATTAG

251    TTAGGGGATTCAAAGAGGATCAANCTAGAATGTATGTGAAATATAATGAG
            (+) ARR1AT S000454 256 NGATT
                  (+) DOFCOREZM S000265 262 AAAG
                   (-) NODCON2GM S000462 263 CTCTT
                   (-) OSE2ROOTNODULE S000468 263 CTCTT
                                          (+) GTGANTG10 S000378 286 GTGA
                                              (-) ROOTMOTIFTAPOX1 S000098 290 ATATT
                                                       (+) ARR1AT S000454 299 NGATT

301    ATTCNAAATTTGCGATCTACTTCAAATATAAACACAACAATTTATTTATA
                        (+) CACTFTPPCA1 S000449 318 YACT
                               (-) ROOTMOTIFTAPOX1 S000098 325 ATATT
                                         (+) RAV1AAT S000314 335 CAACA
                                            (+) CAATBOX1 S000028 338 CAAT
                                               (-) POLASIG1 S000080 341 AATAAA
                                                (+) TATABOX5 S000203 342 TTATTT
                                                  (-) TATABOX2 S000109 344 TATAAAT
                                                       (-) GATABOX S000039 349 GATA

351    TCATATTTGACACAGNTATAATTTTCTGATAAAAGAGATCGATCCTGTTA
         (+) ROOTMOTIFTAPOX1 S000098 353 ATATT
             (+) WBOXATNPR1 S000390 357 TTGAC
              (-) BIHD1OS S000498 358 TGTCA
              (+) WRKY71OS S000447 358 TGAC
                           (-) GT1CONSENSUS S000198 371 GRWAAW
                             (-) POLLEN1LELAT52 S000245 373 AGAAA
                                  (+) GATABOX S000039 378 GATA
                                  (+) GT1CONSENSUS S000198 378 GRWAAW
                                  (+) IBOXCORE S000199 378 GATAA
                                      (+) DOFCOREZM S000265 382 AAAG
                                       (-) NODCON2GM S000462 383 CTCTT
                                       (-) OSE2ROOTNODULE S000468 383 CTCTT
                                                   (+) MYBCORE S000176 395 CNGTTR

401    AACATCCTTCATTATTGTAGATCCGCNCCCCATTTCCAATTGAGAAAGAG
                 (-) POLASIG3 S000088 411 AATAAT
                    (-) CAATBOX1 S000028 414 CAAT
                                      (-) GT1CONSENSUS S000198 432 GRWAAW
                                          (+) CCAATBOX1 S000030 436 CCAAT
                                           (-) EBOXBNNAPA S000144 437 CANNTG
                                           (-) MYCCONSENSUSAT S000407 437 CANNTG
                                           (+) CAATBOX1 S000028 437 CAAT
                                           (+) EBOXBNNAPA S000144 437 CANNTG
                                           (+) MYCCONSENSUSAT S000407 437 CANNTG
                                             (-) CAATBOX1 S000028 439 CAAT
                                                 (+) POLLEN1LELAT52 S000245 443 AGAAA
                                                   (+) DOFCOREZM S000265 445 AAAG
                                                    (-) NODCON2GM S000462 446 CTCTT
                                                    (-) OSE2ROOTNODULE S000468 446 CTCTT

451    GGAACAAGCAAATGGGGGTGGATTTTACTAAATAATTNTAAGCCATGTAC
               (-) EBOXBNNAPA S000144 459 CANNTG
               (-) MYCCONSENSUSAT S000407 459 CANNTG
               (+) EBOXBNNAPA S000144 459 CANNTG
               (+) MYCCONSENSUSAT S000407 459 CANNTG
                          (+) ARR1AT S000454 470 NGATT
                                (+) CACTFTPPCA1 S000449 476 YACT
                                    (-) TATABOX5 S000203 480 TTATTT
                                     (+) POLASIG3 S000088 481 AATAAT
                                                     (-) CURECORECR S000493 497 GTAC
                                                     (+) CURECORECR S000493 497 GTAC
                                                      (+) CACTFTPPCA1 S000449 498 YACT

501    TCCATCTCAATTGTGGAAGTATTCCACAAACGAACTTTTCTTGAGTTGNA
              (-) EBOXBNNAPA S000144 508 CANNTG
              (-) MYCCONSENSUSAT S000407 508 CANNTG
```

(+) CAATBOX1 S000028 508 CAAT
(+) EBOXBNNAPA S000144 508 CANNTG
(+) MYCCONSENSUSAT S000407 508 CANNTG
(-) CAATBOX1 S000028 510 CAAT
(-) CACTFTPPCA1 S000449 518 YACT
(+) AMMORESIVDCRNIA1 S000375 531 CGAACTT
(-) DOFCOREZM S000265 535 AAAG
(-) POLLEN1LELAT52 S000245 537 AGAAA
(-) CAREOSREP1 S000421 543 CAACTC

551 TCTCTCAGATAGTCAGTATTTCTAATAGTCGATATTTCAGAAAATCACTT
(+) GATABOX S000039 558 GATA
(-) WBOXHVISO1 S000442 561 TGACT
(-) WBOXNTERF3 S000457 561 TGACY
(-) WBOXNTCHN48 S000508 561 CTGACY
(-) WRKY71OS S000447 562 TGAC
(-) CACTFTPPCA1 S000449 565 YACT
(-) POLLEN1LELAT52 S000245 569 AGAAA
(-) CPBCSPOR S000491 572 TATTAG
(-) CBFHV S000497 578 RYCGAC
(+) GATABOX S000039 581 GATA
(+) ROOTMOTIFTAPOX1 S000098 582 ATATT
(+) POLLEN1LELAT52 S000245 589 AGAAA
(-) EECCRCAH1 S000494 590 GANTTNC
(+) GT1CONSENSUS S000198 590 GRWAAW
(-) ARR1AT S000454 593 NGATT
(-) GTGANTG10 S000378 595 GTGA
(-) EBOXBNNAPA S000144 596 CANNTG
(-) MYCCONSENSUSAT S000407 596 CANNTG
(+) EBOXBNNAPA S000144 596 CANNTG
(+) MYCCONSENSUSAT S000407 596 CANNTG
(+) CACTFTPPCA1 S000449 596 YACT

601 GTTTTGTTGNAACAAAAAATTTGACTTAGAAAAAATGACTTTAGAGATAA
(-) RAV1AAT S000314 605 CAACA
(+) WBBOXPCWRKY1 S000310 620 TTTGACY
(+) WBOXATNPR1 S000390 621 TTGAC
(+) WBOXHVISO1 S000442 622 TGACT
(+) WRKY71OS S000447 622 TGAC
(+) WBOXNTERF3 S000457 622 TGACY
(+) POLLEN1LELAT52 S000245 628 AGAAA
(+) GT1CONSENSUS S000198 629 GRWAAW
(+) GT1GMSCAM4 S000453 629 GAAAAA
(+) WBOXHVISO1 S000442 636 TGACT
(+) WRKY71OS S000447 636 TGAC
(+) WBOXNTERF3 S000457 636 TGACY
(+) NTBBF1ARROLB S000273 638 ACTTTA
(-) DOFCOREZM S000265 639 AAAG
(-) TAAAGSTKST1 S000387 639 TAAAG
(+) GATABOX S000039 646 GATA
(+) GT1CONSENSUS S000198 646 GRWAAW
(+) IBOXCORE S000199 646 GATAA

651 TAACTAAATCAAACCCTAAANTTTTTCTCTAAGGACCCCAAATTACCTAT
(-) ARR1AT S000454 657 NGATT
(+) TELOBOXATEEF1AA1 S000308 661 AAACCCTAA
(+) UP2ATMSD S000472 661 AAACCCTA
(-) GT1CONSENSUS S000198 672 GRWAAW
(-) GT1GMSCAM4 S000453 672 GAAAAA
(-) POLLEN1LELAT52 S000245 674 AGAAA
(-) GT1CONSENSUS S000198 692 GRWAAW
(-) BIHD1OS S000498 700 TGTCA
(+) WRKY71OS S000447 700 TGAC

701 GACATTGTGATTTGTGATGGAAACGTTGGTGNCGGAATTCTTGGATTCCT
(-) CAATBOX1 S000028 704 CAAT
(+) GTGANTG10 S000378 707 GTGA
(+) ARR1AT S000454 708 NGATT

```
                  (+) GTGANTG10 S000378 714 GTGA
                          (-) ACGTTBOX S000132 722 AACGTT
                          (+) ACGTTBOX S000132 722 AACGTT
                           (-) ACGTATERD1 S000415 723 ACGT
                           (+) ACGTATERD1 S000415 723 ACGT
                                      (-) EECCRCAH1 S000494 734 GANTTNC
                                             (-) RBCSCONSENSUS S000127 741 AATCCAA
                                               (+) ARR1AT S000454 743 NGATT

751     AGTATTTTGTTTTGTTAGGGTAACATTACAACAATAACAACANTATTCAG
        (-) CACTFTPPCA1 S000449 751 YACT
             (-) ANAERO1CONSENSUS S000477 756 AAACAAA
                  (-) AMYBOX1 S000020 761 TAACARA
                  (-) MYBGAHV S000181 761 TAACAAA
                  (-) GAREAT S000439 761 TAACAAR
                                    (+) RAV1AAT S000314 779 CAACA
                                       (+) CAATBOX1 S000028 782 CAAT
                                             (+) RAV1AAT S000314 788 CAACA
                                                        (-) CACTFTPPCA1 S000449 799 YACT

801     TGTATGGTAAAGTGTACGCAGTCCATCCCGACCACTACCTCAGATGAAGT
           (+) S1FBOXSORPS1L21 S000223 804 ATGGTA
             (+) GT1CONSENSUS S000198 806 GRWAAW
               (-) NTBBF1ARROLB S000273 808 ACTTTA
               (+) TAAAGSTKST1 S000387 808 TAAAG
                (+) DOFCOREZM S000265 809 AAAG
                  (-) CACTFTPPCA1 S000449 811 YACT
                     (-) CURECORECR S000493 814 GTAC
                     (+) CURECORECR S000493 814 GTAC
                                   (+) LTRECOREATCOR15 S000153 828 CCGAC
                                   (+) PRECONSCRHSP70A S000506 828 SCGAYNRNNNNNNNNNNNNNNNHD
                                        (+) CACTFTPPCA1 S000449 833 YACT
                                                (-) EBOXBNNAPA S000144 841 CANNTG
                                                (-) MYCCONSENSUSAT S000407 841 CANNTG
                                                (+) EBOXBNNAPA S000144 841 CANNTG
                                                (+) MYCCONSENSUSAT S000407 841 CANNTG
                                                       (-) CACTFTPPCA1 S000449 848 YACT

851     AGANGAGGTTGTTTCCGATAGACCCCTCGGCTCATGTTNGTGTAACATTG
                      (+) PRECONSCRHSP70A S000506 865 SCGAYNRNNNNNNNNNNNNNNNHD
                        (+) GATABOX S000039 867 GATA
                                                      (-) CAATBOX1 S000028 897 CAAT
                                                       (+) WBOXATNPR1 S000390 898 TTGAC
                                                        (-) BIHD1OS S000498 899 TGTCA
                                                        (+) WRKY71OS S000447 899 TGAC

901     ACAATAGAAATTCCNTCTTTCCCTTCATGGTATTTTCTCCTCCTTTTCTT
         (+) CAATBOX1 S000028 902 CAAT
           (+) BOXIINTPATPB S000296 904 ATAGAA
             (+) POLLEN1LELAT52 S000245 906 AGAAA
              (-) EECCRCAH1 S000494 907 GANTTNC
                        (-) DOFCOREZM S000265 917 AAAG
                                  (+) S1FSORPL21 S000215 927 ATGGTATT
                                  (+) S1FBOXSORPS1L21 S000223 927 ATGGTA
                                       (-) GT1CONSENSUS S000198 932 GRWAAW
                                         (-) POLLEN1LELAT52 S000245 934 AGAAA
                                                 (+) PYRIMIDINEBOXOSRAMY1A S000259 942 CCTTTT
                                                  (-) DOFCOREZM S000265 943 AAAG
                                                    (-) POLLEN1LELAT52 S000245 945 AGAAA

951     CTCTACTAGCATTATCACTAGTAATNAAAAAGGCTATGAAGAGAGACTAG
           (+) CACTFTPPCA1 S000449 954 YACT
                  (-) GT1CONSENSUS S000198 961 GRWAAW
                   (-) IBOXCORE S000199 962 GATAA
                    (-) GATABOX S000039 963 GATA
                      (-) GTGANTG10 S000378 965 GTGA
                       (+) CACTFTPPCA1 S000449 966 YACT
                           (-) CACTFTPPCA1 S000449 970 YACT
```

(-) PYRIMIDINEBOXOSRAMY1A S000259 978 CCTTTT
(+) DOFCOREZM S000265 979 AAAG
(-) NODCON2GM S000462 989 CTCTT
(-) OSE2ROOTNODULE S000468 989 CTCTT
(+) SURECOREATSULTR11 S000499 993 GAGAC
(+) EECCRCAH1 S000494 1000 GANTTNC

1001 AATTCCTTTTCATGGGTAATAACTAACATAAGGAGTNAAAGTAGCCTCTT
(-) -300ELEMENT S000122 1005 TGHAAARK
(+) PYRIMIDINEBOXOSRAMY1A S000259 1005 CCTTTT
(-) DOFCOREZM S000265 1006 AAAG
(+) GT1CONSENSUS S000198 1015 GRWAAW
(-) MYB1LEPR S000443 1021 GTTAGTT
(+) DOFCOREZM S000265 1038 AAAG
(-) CACTFTPPCA1 S000449 1040 YACT
(+) NODCON2GM S000462 1046 CTCTT
(+) OSE2ROOTNODULE S000468 1046 CTCTT

1051 GGACTCATCTCTTTTATTTTATTTTCTTTTGCCTTTCGGTGCTCTATNCT
(+) PREATPRODH S000450 1053 ACTCAT
(+) NODCON2GM S000462 1059 CTCTT
(+) OSE2ROOTNODULE S000468 1059 CTCTT
(-) DOFCOREZM S000265 1061 AAAG
(-) POLASIG1 S000080 1063 AATAAA
(+) MARTBOX S000067 1064 TTWTWTTWTT
(+) TATABOX5 S000203 1064 TTATTT
(-) POLASIG1 S000080 1068 AATAAA
(+) TATABOX5 S000203 1069 TTATTT
(-) GT1CONSENSUS S000198 1071 GRWAAW
(-) POLLEN1LELAT52 S000245 1073 AGAAA
(-) DOFCOREZM S000265 1076 AAAG
(-) DOFCOREZM S000265 1083 AAAG
(-) LTRE1HVBLT49 S000250 1084 CCGAAA

1101 TCGACTTTTTCTGACCATTGTTCAGTCACACAGAAAAGTCAGTAAACTAA
(-) DOFCOREZM S000265 1105 AAAG
(-) GT1CONSENSUS S000198 1106 GRWAAW
(-) GT1GMSCAM4 S000453 1106 GAAAAA
(-) POLLEN1LELAT52 S000245 1108 AGAAA
(+) WBOXNTCHN48 S000508 1111 CTGACY
(+) WRKY71OS S000447 1112 TGAC
(+) WBOXNTERF3 S000457 1112 TGACY
(-) CAATBOX1 S000028 1117 CAAT
(-) WBOXHVISO1 S000442 1124 TGACT
(-) WBOXNTERF3 S000457 1124 TGACY
(-) WRKY71OS S000447 1125 TGAC
(-) GTGANTG10 S000378 1126 GTGA
(+) POLLEN1LELAT52 S000245 1132 AGAAA
(+) DOFCOREZM S000265 1135 AAAG
(-) WBOXHVISO1 S000442 1137 TGACT
(-) WBOXNTERF3 S000457 1137 TGACY
(-) WBOXNTCHN48 S000508 1137 CTGACY
(-) WRKY71OS S000447 1138 TGAC
(-) CACTFTPPCA1 S000449 1141 YACT
(+) POLLEN1LELAT52 S000245 1150 AGAAA

1151 GAAAATGANCATGACCACAACTAAAATCATGAAGTACATAATATGAGAGT
(+) GT1CONSENSUS S000198 1151 GRWAAW
(+) WRKY71OS S000447 1162 TGAC
(+) WBOXNTERF3 S000457 1162 TGACY
(-) ARR1AT S000454 1175 NGATT
(-) CACTFTPPCA1 S000449 1183 YACT
(-) CURECORECR S000493 1184 GTAC
(+) CURECORECR S000493 1184 GTAC
(-) ROOTMOTIFTAPOX1 S000098 1190 ATATT
(-) CACTFTPPCA1 S000449 1198 YACT

1201 AAGCAAATTCAAATATCTTNTTAAAAAAAAAAAAAATCAAGAGAAAAAAC

(+) ERELEE4 S000037 1206 AWTTCAAA
(-) ROOTMOTIFTAPOX1 S000098 1212 ATATT
(-) GATABOX S000039 1214 GATA
(-) MARTBOX S000067 1223 TTWTWTTWTT
(-) MARTBOX S000067 1224 TTWTWTTWTT
(-) MARTBOX S000067 1225 TTWTWTTWTT
(-) MARTBOX S000067 1226 TTWTWTTWTT
(-) MARTBOX S000067 1227 TTWTWTTWTT
(-) ARR1AT S000454 1235 NGATT
(-) NODCON2GM S000462 1239 CTCTT
(-) OSE2ROOTNODULE S000468 1239 CTCTT
(+) POLLEN1LELAT52 S000245 1242 AGAAA
(+) GT1CONSENSUS S000198 1243 GRWAAW
(+) GT1GMSCAM4 S000453 1243 GAAAAA
(+) ANAERO1CONSENSUS S000477 1247 AAACAAA

1251 AAATTCAACGTGTGATATCCAAAGTAAATTNTTCTGATGTAATCACTCTA
(+) BP5OSWX S000436 1256 CAACGTG
(+) QARBNEXTA S000244 1257 AACGTGT
(+) T/GBOXATPIN2 S000458 1257 AACGTG
(+) ABRERATCAL S000507 1257 MACGYGB
(-) ACGTATERD1 S000415 1258 ACGT
(+) ABRELATERD1 S000414 1258 ACGTG
(+) ACGTATERD1 S000415 1258 ACGT
(+) GTGANTG10 S000378 1262 GTGA
(+) GATABOX S000039 1264 GATA
(-) GATABOX S000039 1266 GATA
(-) MYBST1 S000180 1266 GGATA
(+) TATCCAOSAMY S000403 1266 TATCCA
(-) TBOXATGAPB S000383 1270 ACTTTG
(+) DOFCOREZM S000265 1271 AAAG
(-) CACTFTPPCA1 S000449 1273 YACT
(-) ARR1AT S000454 1291 NGATT
(-) GTGANTG10 S000378 1293 GTGA
(+) CACTFTPPCA1 S000449 1294 YACT

1301 GCATCATCATTTTATTTTGTCTCTTCAAATCAAATAACGTANACTACATA
(-) POLASIG1 S000080 1311 AATAAA
(+) TATABOX5 S000203 1312 TTATTT
(+) SEBFCONSSTPR10A S000391 1317 YTGTCWC
(+) ARFAT S000270 1318 TGTCTC
(-) SURECOREATSULTR11 S000499 1319 GAGAC
(+) NODCON2GM S000462 1321 CTCTT
(+) OSE2ROOTNODULE S000468 1321 CTCTT
(-) ARR1AT S000454 1328 NGATT
(-) TATABOX5 S000203 1332 TTATTT
(+) GARE2OSREP1 S000420 1335 TAACGTA
(-) ACGTATERD1 S000415 1337 ACGT
(+) ACGTATERD1 S000415 1337 ACGT

1351 CATTATATTATATAAATGGAGGAAAAAGATTGATAAAAGAAAAATAATTT
(+) ROOTMOTIFTAPOX1 S000098 1355 ATATT
(-) TATABOX4 S000111 1358 TATATAA
(-) TATAPVTRNALEU S000340 1359 TTTATATA
(+) TATABOX4 S000111 1359 TATATAA
(+) TATABOX2 S000109 1361 TATAAAT
(+) GT1CONSENSUS S000198 1371 GRWAAW
(+) GT1CONSENSUS S000198 1372 GRWAAW
(+) GT1GMSCAM4 S000453 1372 GAAAAA
(+) DOFCOREZM S000265 1375 AAAG
(+) NODCON1GM S000461 1375 AAAGAT
(+) OSE1ROOTNODULE S000467 1375 AAAGAT
(+) ARR1AT S000454 1377 NGATT
(-) CAATBOX1 S000028 1379 CAAT
(+) GATABOX S000039 1382 GATA
(+) GT1CONSENSUS S000198 1382 GRWAAW
(+) IBOXCORE S000199 1382 GATAA
(+) DOFCOREZM S000265 1386 AAAG

(+) POLLEN1LELAT52 S000245 1388 AGAAA
(+) GT1CONSENSUS S000198 1389 GRWAAW
(+) GT1GMSCAM4 S000453 1389 GAAAAA
(-) TATABOX5 S000203 1392 TTATTT
(+) POLASIG3 S000088 1393 AATAAT

1401 ATNTAGTACACACTAATAACAATGAAGAGGAAAAAATTAAAGCAAAATAT
(-) CACTFTPPCA1 S000449 1405 YACT
(-) CURECORECR S000493 1406 GTAC
(+) CURECORECR S000493 1406 GTAC
(+) CACTFTPPCA1 S000449 1411 YACT
(-) CPBCSPOR S000491 1413 TATTAG
(+) CAATBOX1 S000028 1420 CAAT
(-) NODCON2GM S000462 1425 CTCTT
(-) OSE2ROOTNODULE S000468 1425 CTCTT
(-) PYRIMIDINEBOXHVEPB1 S000298 1429 TTTTTTCC
(+) GT1CONSENSUS S000198 1429 GRWAAW
(+) GT1CONSENSUS S000198 1430 GRWAAW
(+) GT1GMSCAM4 S000453 1430 GAAAAA
(+) POLASIG2 S000081 1435 AATTAAA
(+) TAAAGSTKST1 S000387 1438 TAAAG
(+) DOFCOREZM S000265 1439 AAAG
(-) ROOTMOTIFTAPOX1 S000098 1446 ATATT

1451 ATATGTTGTGACTNTGTGAGAGGATTATGTCAATTATCCAGTGGACAAAT
(-) RAV1AAT S000314 1454 CAACA
(+) GTGANTG10 S000378 1458 GTGA
(+) WBOXHVISO1 S000442 1459 TGACT
(+) WRKY71OS S000447 1459 TGAC
(+) WBOXNTERF3 S000457 1459 TGACY
(+) GTGANTG10 S000378 1466 GTGA
(+) ARR1AT S000454 1472 NGATT
(+) BIHD1OS S000498 1478 TGTCA
(-) WBOXATNPR1 S000390 1479 TTGAC
(-) WRKY71OS S000447 1479 TGAC
(+) CAATBOX1 S000028 1481 CAAT
(-) GT1CONSENSUS S000198 1483 GRWAAW
(-) IBOXCORE S000199 1484 GATAA
(+) SREATMSD S000470 1484 TTATCC
(-) GATABOX S000039 1485 GATA
(-) MYBST1 S000180 1485 GGATA
(+) TATCCAOSAMY S000403 1485 TATCCA
(-) CACTFTPPCA1 S000449 1490 YACT
(-) EBOXBNNAPA S000144 1496 CANNTG
(-) MYCCONSENSUSAT S000407 1496 CANNTG
(+) EBOXBNNAPA S000144 1496 CANNTG
(+) MYCCONSENSUSAT S000407 1496 CANNTG

1501 GCATACTCCTATAACCTACTTATTNTTATTTTCAGAAAAAAGAATGTGAT
(+) CACTFTPPCA1 S000449 1504 YACT
(+) CACTFTPPCA1 S000449 1517 YACT
(+) TATABOX5 S000203 1526 TTATTT
(-) GT1CONSENSUS S000198 1528 GRWAAW
(+) POLLEN1LELAT52 S000245 1534 AGAAA
(+) GT1CONSENSUS S000198 1535 GRWAAW
(+) GT1GMSCAM4 S000453 1535 GAAAAA
(+) DOFCOREZM S000265 1539 AAAG
(+) GTGANTG10 S000378 1546 GTGA
(-) MYB1AT S000408 1550 WAACCA

1551 GGTTTCAAGATTAGTACAATTATTTATGATCAGTGNCAGTGCAGTAGTAG
(+) ARR1AT S000454 1558 NGATT
(-) CACTFTPPCA1 S000449 1563 YACT
(-) CURECORECR S000493 1564 GTAC
(+) CURECORECR S000493 1564 GTAC
(+) CAATBOX1 S000028 1567 CAAT
(-) POLASIG3 S000088 1569 AATAAT
(+) TATABOX5 S000203 1570 TTATTT

(-) CACTFTPPCA1 S000449 1582 YACT
(-) CACTFTPPCA1 S000449 1588 YACT
(-) CACTFTPPCA1 S000449 1593 YACT
(-) CACTFTPPCA1 S000449 1596 YACT

1601 AACCTGATGAGGATCGACCAATAAATTGGAAGAGGAAATAAAAGGANAAA
(+) CBFHV S000497 1613 RYCGAC
(-) CARGNCAT S000446 1618 CCWWWWWWWWGG
(+) CCAATBOX1 S000030 1618 CCAAT
(+) CARGNCAT S000446 1618 CCWWWWWWWWGG
(-) CARGCW8GAT S000431 1619 CWWWWWWWWG
(+) CAATBOX1 S000028 1619 CAAT
(+) CARGCW8GAT S000431 1619 CWWWWWWWWG
(+) POLASIG1 S000080 1620 AATAAA
(-) CAATBOX1 S000028 1625 CAAT
(-) CCAATBOX1 S000030 1625 CCAAT
(-) NODCON2GM S000462 1630 CTCTT
(-) OSE2ROOTNODULE S000468 1630 CTCTT
(+) GT1CONSENSUS S000198 1634 GRWAAW
(-) TATABOX5 S000203 1636 TTATTT
(+) POLASIG1 S000080 1637 AATAAA
(-) PYRIMIDINEBOXOSRAMY1A S000259 1640 CCTTTT
(+) DOFCOREZM S000265 1641 AAAG

1651 TGATAACCAAATTATGAGGAATAATTAACGAACTTGTTAAATGGTGGACT
(+) GATABOX S000039 1652 GATA
(+) IBOXCORE S000199 1652 GATAA
(+) MYB1AT S000408 1654 WAACCA
(+) REALPHALGLHCB21 S000362 1655 AACCAA
(+) POLASIG3 S000088 1670 AATAAT
(+) AMMORESIVDCRNIA1 S000375 1679 CGAACTT
(-) GAREAT S000439 1683 TAACAAR
(-) CPBCSPOR S000491 1699 TATTAG

1701 AATAATTNATTGGAATCCTTAAAAAAATATTATATTCACAAGAAACCCTA
(+) POLASIG3 S000088 1701 AATAAT
(-) CAATBOX1 S000028 1709 CAAT
(-) CCAATBOX1 S000030 1709 CCAAT
(-) ARR1AT S000454 1714 NGATT
(-) ROOTMOTIFTAPOX1 S000098 1726 ATATT
(+) ROOTMOTIFTAPOX1 S000098 1727 ATATT
(+) ROOTMOTIFTAPOX1 S000098 1732 ATATT
(-) GTGANTG10 S000378 1736 GTGA
(+) POLLEN1LELAT52 S000245 1741 AGAAA
(+) UP2ATMSD S000472 1743 AAACCCTA

1751 GTCCTATCCTATGTATAGNAGATCTAGTTCTATAAATATTAAATTAAATA
(-) GATABOX S000039 1755 GATA
(-) MYBST1 S000180 1755 GGATA
(-) BOXIINTPATPB S000296 1778 ATAGAA
(-) SEF1MOTIF S000006 1781 ATATTTAWW
(+) TATABOX2 S000109 1781 TATAAAT
(-) ROOTMOTIFTAPOX1 S000098 1785 ATATT
(+) ROOTMOTIFTAPOX1 S000098 1786 ATATT
(+) POLASIG2 S000081 1792 AATTAAA
(-) SEF1MOTIF S000006 1793 ATATTTAWW
(-) TATABOXOSPAL S000400 1794 TATTTAA
(-) ROOTMOTIFTAPOX1 S000098 1797 ATATT
(+) SEF1MOTIF S000006 1798 ATATTTAWW
(+) ROOTMOTIFTAPOX1 S000098 1798 ATATT
(+) TATABOXOSPAL S000400 1799 TATTTAA

1801 TTTAATTTAAAGCGTGAGTATTATTTAATNTTCTGCCTAGGTACTTGTTG
(-) POLASIG2 S000081 1801 AATTAAA
(+) TAAAGSTKST1 S000387 1808 TAAAG
(+) DOFCOREZM S000265 1809 AAAG
(+) GTGANTG10 S000378 1814 GTGA
(-) CACTFTPPCA1 S000449 1817 YACT

```
                                   (-) POLASIG3 S000088 1820 AATAAT
                                    (+) TATABOX5 S000203 1821 TTATTT
                                     (+) TATABOXOSPAL S000400 1822 TATTTAA
                                                        (-) CURECORECR S000493 1841 GTAC
                                                        (+) CURECORECR S000493 1841 GTAC
                                                         (+) CACTFTPPCA1 S000449 1842 YACT
                                                             (-) RAV1AAT S000314 1846 CAACA
                                                                (-) TATCCAOSAMY S000403 1849 TATCCA
                                                                 (+) MYBST1 S000180 1850 GGATA

1851    GATAGGGTTTGAACAAAATTAACTGGATCTGAACTAAAATNCATATTTAC
        (+) GATABOX S000039 1851 GATA
          (-) UP2ATMSD S000472 1853 AAACCCTA
                           (-) MYBCORE S000176 1870 CNGTTR
                           (+) MYB2AT S000177 1870 TAACTG
                           (+) MYB2CONSENSUSAT S000409 1870 YAACKG
                                                 (+) ROOTMOTIFTAPOX1 S000098 1893 ATATT
                                                      (+) CACTFTPPCA1 S000449 1898 YACT

1901    TTCCATTAACCTTCAAAAATCAATCAAGGAAAAAAGAGAAAACAGTGAAA
          (-) WUSATAg S000433 1903 TTAATGG
             (-) GT1CORE S000125 1906 GGTTAA
                     (-) SEF4MOTIFGM7S S000103 1914 RTTTTTR
                         (-) ARR1AT S000454 1918 NGATT
                            (+) CAATBOX1 S000028 1921 CAAT
                             (-) ARR1AT S000454 1922 NGATT
                                   (-) PYRIMIDINEBOXHVEPB1 S000298 1928 TTTTTTCC
                                   (+) GT1CONSENSUS S000198 1928 GRWAAW
                                    (+) GT1CONSENSUS S000198 1929 GRWAAW
                                    (+) GT1GMSCAM4 S000453 1929 GAAAAA
                                        (+) DOFCOREZM S000265 1933 AAAG
                                         (-) NODCON2GM S000462 1934 CTCTT
                                         (-) OSE2ROOTNODULE S000468 1934 CTCTT
                                            (+) POLLEN1LELAT52 S000245 1937 AGAAA
                                                   (-) CACTFTPPCA1 S000449 1944 YACT
                                                    (+) GTGANTG10 S000378 1945 GTGA

1951    CNACTTTGTTTTAAATTATTTGAAAATTAGCATAAAATATGACAATTTTC
          (+) TBOXATGAPB S000383 1953 ACTTTG
           (-) DOFCOREZM S000265 1954 AAAG
            (-) ANAERO1CONSENSUS S000477 1955 AAACAAA
                      (-) POLASIG3 S000088 1965 AATAAT
                       (+) TATABOX5 S000203 1966 TTATTT
                             (+) GT1CONSENSUS S000198 1972 GRWAAW
                                        (+) LECPLEACS2 S000465 1983 TAAAATAT
                                           (-) ROOTMOTIFTAPOX1 S000098 1986 ATATT
                                               (-) BIHD1OS S000498 1990 TGTCA
                                               (+) WRKY71OS S000447 1990 TGAC
                                                  (+) CAATBOX1 S000028 1993 CAAT
                                                    (-) GT1CONSENSUS S000198 1995 GRWAAW
                                                      (-) POLLEN1LELAT52 S000245 1997 AGAAA
                                                         (+) CANBNNAPA S000148 2000 CNAACAC

2001    TAACACTTTGAANGCAAAATTTCTAGAAACATCACATCTTTACGGTTGAT
           (+) CACTFTPPCA1 S000449 2004 YACT
            (+) TBOXATGAPB S000383 2005 ACTTTG
             (-) DOFCOREZM S000265 2006 AAAG
                           (-) POLLEN1LELAT52 S000245 2020 AGAAA
                                (+) POLLEN1LELAT52 S000245 2025 AGAAA
                                       (-) GTGANTG10 S000378 2032 GTGA
                                           (-) NODCON1GM S000461 2036 AAAGAT
                                           (-) OSE1ROOTNODULE S000467 2036 AAAGAT
                                             (-) DOFCOREZM S000265 2038 AAAG
                                             (-) TAAAGSTKST1 S000387 2038 TAAAG
                                                  (+) MYBCORE S000176 2043 CNGTTR
                                                      (+) ARR1AT S000454 2047 NGATT
                                                         (-) GT1CONSENSUS S000198 2050 GRWAAW
```

2051 TTATCAAATGGTCACTCAAGTAANTATATAGTTATTATCTCAATTTGTTA
(-) IBOXCORE S000199 2051 GATAA
(-) GATABOX S000039 2052 GATA
(-) EBOXBNNAPA S000144 2055 CANNTG
(-) MYCCONSENSUSAT S000407 2055 CANNTG
(+) EBOXBNNAPA S000144 2055 CANNTG
(+) MYCCONSENSUSAT S000407 2055 CANNTG
(-) WBOXNTERF3 S000457 2060 TGACY
(-) WRKY71OS S000447 2061 TGAC
(-) GTGANTG10 S000378 2062 GTGA
(+) CACTFTPPCA1 S000449 2063 YACT
(-) CACTFTPPCA1 S000449 2069 YACT
(-) GT1CONSENSUS S000198 2084 GRWAAW
(-) IBOXCORE S000199 2085 GATAA
(-) GATABOX S000039 2086 GATA
(+) INRNTPSADB S000395 2089 YTCANTYY
(+) CAATBOX1 S000028 2091 CAAT
(-) AMYBOX1 S000020 2094 TAACARA
(-) MYBGAHV S000181 2094 TAACAAA
(-) GAREAT S000439 2094 TAACAAR
(-) IBOXCORE S000199 2098 GATAA
(-) GATABOX S000039 2099 GATA
(-) NODCON1GM S000461 2100 AAAGAT
(-) OSE1ROOTNODULE S000467 2100 AAAGAT

2101 TCTTTCTTTTTTATTACAATCTTTTCCAACAAAGNAAAACAATTTTCCGA
(-) DOFCOREZM S000265 2102 AAAG
(-) POLLEN1LELAT52 S000245 2103 AGAAA
(-) DOFCOREZM S000265 2106 AAAG
(-) POLASIG1 S000080 2110 AATAAA
(+) CAATBOX1 S000028 2117 CAAT
(-) ARR1AT S000454 2118 NGATT
(-) NODCON1GM S000461 2119 AAAGAT
(-) OSE1ROOTNODULE S000467 2119 AAAGAT
(-) DOFCOREZM S000265 2121 AAAG
(-) GT1CONSENSUS S000198 2122 GRWAAW
(+) RAV1AAT S000314 2127 CAACA
(+) DOFCOREZM S000265 2131 AAAG
(+) CAATBOX1 S000028 2140 CAAT
(-) GT1CONSENSUS S000198 2142 GRWAAW
(-) GT1CONSENSUS S000198 2143 GRWAAW
(+) POLLEN1LELAT52 S000245 2150 AGAAA

2151 GAAAATAACTACTATTAATTGAATGACCATCTGAAAAATCATCTCNGGCA
(+) GT1CONSENSUS S000198 2151 GRWAAW
(-) TATABOX5 S000203 2153 TTATTT
(+) SP8BFIBSP8BIB S000184 2160 TACTATT
(+) CACTFTPPCA1 S000449 2160 YACT
(-) CARGCW8GAT S000431 2162 CWWWWWWWWG
(+) CARGCW8GAT S000431 2162 CWWWWWWWWG
(+) TATABOX3 S000110 2163 TATTAAT
(-) CAATBOX1 S000028 2168 CAAT
(+) WRKY71OS S000447 2174 TGAC
(+) WBOXNTERF3 S000457 2174 TGACY
(-) EBOXBNNAPA S000144 2178 CANNTG
(-) MYCCONSENSUSAT S000407 2178 CANNTG
(+) EBOXBNNAPA S000144 2178 CANNTG
(+) MYCCONSENSUSAT S000407 2178 CANNTG
(+) -300ELEMENT S000122 2182 TGHAAARK
(+) GT1CONSENSUS S000198 2183 GRWAAW
(+) GT1GMSCAM4 S000453 2183 GAAAAA
(-) ARR1AT S000454 2187 NGATT
(-) NODCON1GM S000461 2200 AAAGAT
(-) OSE1ROOTNODULE S000467 2200 AAAGAT

2201 TCTTTCTAACCCTATCTTATGGCTTCTACTAAAAAGAAAAAGAAGACTTC
(-) DOFCOREZM S000265 2202 AAAG
(-) POLLEN1LELAT52 S000245 2203 AGAAA

(-) GATABOX S000039 2213 GATA
(+) CACTFTPPCA1 S000449 2227 YACT
(+) DOFCOREZM S000265 2233 AAAG
(+) POLLEN1LELAT52 S000245 2235 AGAAA
(+) GT1CONSENSUS S000198 2236 GRWAAW
(+) GT1GMSCAM4 S000453 2236 GAAAAA
(+) DOFCOREZM S000265 2239 AAAG

2251 GGTTTANATTTCACAAGATTTGCTTTCAAAAAATCTAGAAAAAAGTGGAA
(-) GTGANTG10 S000378 2261 GTGA
(+) ARR1AT S000454 2266 NGATT
(+) EECCRCAH1 S000494 2267 GANTTNC
(-) DOFCOREZM S000265 2273 AAAG
(+) CCA1ATLHCB1 S000149 2279 AAMAATCT
(-) ARR1AT S000454 2282 NGATT
(+) POLLEN1LELAT52 S000245 2287 AGAAA
(+) GT1CONSENSUS S000198 2288 GRWAAW
(+) GT1GMSCAM4 S000453 2288 GAAAAA
(+) DOFCOREZM S000265 2292 AAAG
(-) CACTFTPPCA1 S000449 2294 YACT

2301 CAAAATTAAACTTTGAANCTTGTATTCATCATCATAGACAAAATGAGAGC
(+) POLASIG2 S000081 2304 AATTAAA
(+) TBOXATGAPB S000383 2310 ACTTTG
(-) DOFCOREZM S000265 2311 AAAG
(-) INRNTPSADB S000395 2340 YTCANTYY

2351 TCAGAAAAATGGCTCTTAATTTCTTCAANACGATTCGATCATGCATTTGA
(+) POLLEN1LELAT52 S000245 2353 AGAAA
(+) GT1CONSENSUS S000198 2354 GRWAAW
(+) GT1GMSCAM4 S000453 2354 GAAAAA
(+) NODCON2GM S000462 2363 CTCTT
(+) OSE2ROOTNODULE S000468 2363 CTCTT
(-) POLLEN1LELAT52 S000245 2370 AGAAA
(+) ARR1AT S000454 2381 NGATT
(+) RYREPEATLEGUMINBOX S000100 2390 CATGCAY
(+) RYREPEATGMGY2 S000105 2390 CATGCAT
(+) RYREPEATBNNAPA S000264 2390 CATGCA
(-) EBOXBNNAPA S000144 2394 CANNTG
(-) MYCCONSENSUSAT S000407 2394 CANNTG
(+) EBOXBNNAPA S000144 2394 CANNTG
(+) MYCCONSENSUSAT S000407 2394 CANNTG

2401 AAACCAGCTTAGCATATATAAAGGGTTTTAAAATTTTTGNAAGGAGATGG
(+) MYB1AT S000408 2401 WAACCA
(-) TATAPVTRNALEU S000340 2415 TTTATATA
(+) TATABOX4 S000111 2415 TATATAA
(+) TAAAGSTKST1 S000387 2419 TAAAG
(+) DOFCOREZM S000265 2420 AAAG
(+) SEF4MOTIFGM7S S000103 2433 RTTTTTR

2451 ATGAAAAAATTAAAATAAAAGAAGTGTACCGCAAGAATTGTGTTGCAGAA
(+) GT1CONSENSUS S000198 2453 GRWAAW
(+) GT1GMSCAM4 S000453 2453 GAAAAA
(+) POLASIG2 S000081 2458 AATTAAA
(-) TATABOX5 S000203 2463 TTATTT
(+) POLASIG1 S000080 2464 AATAAA
(+) DOFCOREZM S000265 2468 AAAG
(-) CACTFTPPCA1 S000449 2473 YACT
(-) CURECORECR S000493 2476 GTAC
(+) CURECORECR S000493 2476 GTAC
(-) CAATBOX1 S000028 2487 CAAT
(-) RAV1AAT S000314 2491 CAACA

2501 NGTTGCAATGAACATAGCAAACACGTTCCAAGGAAGATGAATATGTCATG
(+) CAATBOX1 S000028 2506 CAAT
(+) 2SSEEDPROTBANAPA S000143 2518 CAAACAC
(+) CANBNNAPA S000148 2518 CNAACAC

```
                           (-) QARBNEXTA S000244 2521 AACGTGT
                           (-) ABRERATCAL S000507 2521 MACGYGB
                            (-) ABRELATERD1 S000414 2522 ACGTG
                            (-) T/GBOXATPIN2 S000458 2522 AACGTG
                             (-) ACGTATERD1 S000415 2523 ACGT
                             (+) ACGTATERD1 S000415 2523 ACGT
                                              (-) ROOTMOTIFTAPOX1 S000098 2540 ATATT
                                                  (+) BIHD1OS S000498 2544 TGTCA
                                                   (-) WRKY71OS S000447 2545 TGAC

2551   TCTAGTGACATNTTTTCTTAAAGGTGTTTTTCTTTTCTTTTTCTTTTTTT
          (-) CACTFTPPCA1 S000449 2554 YACT
           (+) GTGANTG10 S000378 2555 GTGA
            (-) BIHD1OS S000498 2556 TGTCA
            (+) WRKY71OS S000447 2556 TGAC
                  (-) GT1CONSENSUS S000198 2562 GRWAAW
                    (-) POLLEN1LELAT52 S000245 2564 AGAAA
                         (+) TAAAGSTKST1 S000387 2569 TAAAG
                          (+) DOFCOREZM S000265 2570 AAAG
                                 (-) GT1CONSENSUS S000198 2577 GRWAAW
                                 (-) GT1GMSCAM4 S000453 2577 GAAAAA
                                   (-) POLLEN1LELAT52 S000245 2579 AGAAA
                                      (-) DOFCOREZM S000265 2582 AAAG
                                        (-) POLLEN1LELAT52 S000245 2584 AGAAA
                                           (-) DOFCOREZM S000265 2587 AAAG
                                            (-) GT1CONSENSUS S000198 2588 GRWAAW
                                            (-) GT1GMSCAM4 S000453 2588 GAAAAA
                                              (-) POLLEN1LELAT52 S000245 2590 AGAAA
                                                 (-) DOFCOREZM S000265 2593 AAAG
                                                 (-) CARGCW8GAT S000431 2593 CWWWWWWWWG
                                                 (+) CARGCW8GAT S000431 2593 CWWWWWWWWG

2601   AGCCCTGGAATCACGGATCGAANGAAGAGACGGATCAACAAGGAGCTGTG
               (-) ARR1AT S000454 2609 NGATT
                 (-) GTGANTG10 S000378 2611 GTGA
                               (-) NODCON2GM S000462 2625 CTCTT
                               (-) OSE2ROOTNODULE S000468 2625 CTCTT
                                 (+) SURECOREATSULTR11 S000499 2627 GAGAC
                                          (+) RAV1AAT S000314 2636 CAACA
                                                      (+) GTGANTG10 S000378 2648 GTGA

2651   AACTGA
```

Factor or Site Name	Loc.(Str.)	Signal Sequence	SITE #
INRNTPSADB	1 (+)	YTCANTYY	S000395
GTGANTG10	2 (-)	GTGA	S000378
CACTFTPPCA1	3 (+)	YACT	S000449
DOFCOREZM	5 (-)	AAAG	S000265
POLLEN1LELAT52	14 (+)	AGAAA	S000245
DOFCOREZM	16 (+)	AAAG	S000265
CACTFTPPCA1	18 (-)	YACT	S000449
CACTFTPPCA1	22 (-)	YACT	S000449
ROOTMOTIFTAPOX1	29 (-)	ATATT	S000098
CACTFTPPCA1	33 (+)	YACT	S000449
TAAAGSTKST1	48 (+)	TAAAG	S000387
DOFCOREZM	49 (+)	AAAG	S000265
ARR1AT	53 (-)	NGATT	S000454
RAV1AAT	56 (+)	CAACA	S000314
SP8BFIBSP8BIB	67 (+)	TACTATT	S000184
CACTFTPPCA1	67 (+)	YACT	S000449
CACTFTPPCA1	77 (-)	YACT	S000449
DOFCOREZM	81 (+)	AAAG	S000265
NODCON2GM	82 (-)	CTCTT	S000462
OSE2ROOTNODULE	82 (-)	CTCTT	S000468
WBOXHVISO1	89 (-)	TGACT	S000442
WBOXNTERF3	89 (-)	TGACY	S000457

WRKY71OS	90	(-)	TGAC	S000447
GTGANTG10	91	(-)	GTGA	S000378
EBOXBNNAPA	94	(-)	CANNTG	S000144
MYCCONSENSUSAT	94	(-)	CANNTG	S000407
EBOXBNNAPA	94	(+)	CANNTG	S000144
MYCCONSENSUSAT	94	(+)	CANNTG	S000407
POLASIG2	105	(-)	AATTAAA	S000081
POLASIG2	108	(+)	AATTAAA	S000081
TAAAGSTKST1	111	(+)	TAAAG	S000387
DOFCOREZM	112	(+)	AAAG	S000265
WBBOXPCWRKY1	133	(+)	TTTGACY	S000310
WBOXATNPR1	134	(+)	TTGAC	S000390
WBOXHVISO1	135	(+)	TGACT	S000442
WRKY71OS	135	(+)	TGAC	S000447
WBOXNTERF3	135	(+)	TGACY	S000457
SORLIP2AT	148	(+)	GGGCC	S000483
CGCGBOXAT	151	(-)	VCGCGB	S000501
CGCGBOXAT	151	(+)	VCGCGB	S000501
CPBCSPOR	162	(+)	TATTAG	S000491
ARR1AT	169	(+)	NGATT	S000454
XYLAT	172	(-)	ACAAAGAA	S000510
DOFCOREZM	174	(-)	AAAG	S000265
RAV1AAT	177	(-)	CAACA	S000314
ROOTMOTIFTAPOX1	184	(-)	ATATT	S000098
GATABOX	196	(+)	GATA	S000039
S1FBOXSORPS1L21	204	(+)	ATGGTA	S000223
GT1CORE	212	(-)	GGTTAA	S000125
MYB1AT	213	(+)	WAACCA	S000408
REALPHALGLHCB21	214	(+)	AACCAA	S000362
ROOTMOTIFTAPOX1	222	(-)	ATATT	S000098
SORLREP3AT	226	(+)	TGTATATAT	S000488
ROOTMOTIFTAPOX1	248	(+)	ATATT	S000098
CPBCSPOR	249	(+)	TATTAG	S000491
ARR1AT	256	(+)	NGATT	S000454
DOFCOREZM	262	(+)	AAAG	S000265
NODCON2GM	263	(-)	CTCTT	S000462
OSE2ROOTNODULE	263	(-)	CTCTT	S000468
GTGANTG10	286	(+)	GTGA	S000378
ROOTMOTIFTAPOX1	290	(-)	ATATT	S000098
ARR1AT	299	(+)	NGATT	S000454
CACTFTPPCA1	318	(+)	YACT	S000449
ROOTMOTIFTAPOX1	325	(-)	ATATT	S000098
RAV1AAT	335	(+)	CAACA	S000314
CAATBOX1	338	(+)	CAAT	S000028
POLASIG1	341	(-)	AATAAA	S000080
TATABOX5	342	(+)	TTATTT	S000203
TATABOX2	344	(-)	TATAAAT	S000109
GATABOX	349	(-)	GATA	S000039
ROOTMOTIFTAPOX1	353	(+)	ATATT	S000098
WBOXATNPR1	357	(+)	TTGAC	S000390
BIHD1OS	358	(-)	TGTCA	S000498
WRKY71OS	358	(+)	TGAC	S000447
GT1CONSENSUS	371	(-)	GRWAAW	S000198
POLLEN1LELAT52	373	(-)	AGAAA	S000245
GATABOX	378	(+)	GATA	S000039
GT1CONSENSUS	378	(+)	GRWAAW	S000198
IBOXCORE	378	(+)	GATAA	S000199
DOFCOREZM	382	(+)	AAAG	S000265
NODCON2GM	383	(-)	CTCTT	S000462
OSE2ROOTNODULE	383	(-)	CTCTT	S000468
MYBCORE	395	(+)	CNGTTR	S000176
POLASIG3	411	(-)	AATAAT	S000088
CAATBOX1	414	(-)	CAAT	S000028
GT1CONSENSUS	432	(-)	GRWAAW	S000198
CCAATBOX1	436	(+)	CCAAT	S000030
EBOXBNNAPA	437	(-)	CANNTG	S000144
MYCCONSENSUSAT	437	(-)	CANNTG	S000407
CAATBOX1	437	(+)	CAAT	S000028

EBOXBNNAPA	437	(+)	CANNTG	S000144
MYCCONSENSUSAT	437	(+)	CANNTG	S000407
CAATBOX1	439	(-)	CAAT	S000028
POLLEN1LELAT52	443	(+)	AGAAA	S000245
DOFCOREZM	445	(+)	AAAG	S000265
NODCON2GM	446	(-)	CTCTT	S000462
OSE2ROOTNODULE	446	(-)	CTCTT	S000468
EBOXBNNAPA	459	(-)	CANNTG	S000144
MYCCONSENSUSAT	459	(-)	CANNTG	S000407
EBOXBNNAPA	459	(+)	CANNTG	S000144
MYCCONSENSUSAT	459	(+)	CANNTG	S000407
ARR1AT	470	(+)	NGATT	S000454
CACTFTPPCA1	476	(+)	YACT	S000449
TATABOX5	480	(-)	TTATTT	S000203
POLASIG3	481	(+)	AATAAT	S000088
CURECORECR	497	(-)	GTAC	S000493
CURECORECR	497	(+)	GTAC	S000493
CACTFTPPCA1	498	(+)	YACT	S000449
EBOXBNNAPA	508	(-)	CANNTG	S000144
MYCCONSENSUSAT	508	(-)	CANNTG	S000407
CAATBOX1	508	(+)	CAAT	S000028
EBOXBNNAPA	508	(+)	CANNTG	S000144
MYCCONSENSUSAT	508	(+)	CANNTG	S000407
CAATBOX1	510	(-)	CAAT	S000028
CACTFTPPCA1	518	(-)	YACT	S000449
AMMORESIVDCRNIA1	531	(+)	CGAACTT	S000375
DOFCOREZM	535	(-)	AAAG	S000265
POLLEN1LELAT52	537	(-)	AGAAA	S000245
CAREOSREP1	543	(-)	CAACTC	S000421
GATABOX	558	(+)	GATA	S000039
WBOXHVISO1	561	(-)	TGACT	S000442
WBOXNTERF3	561	(-)	TGACY	S000457
WBOXNTCHN48	561	(-)	CTGACY	S000508
WRKY71OS	562	(-)	TGAC	S000447
CACTFTPPCA1	565	(-)	YACT	S000449
POLLEN1LELAT52	569	(-)	AGAAA	S000245
CPBCSPOR	572	(-)	TATTAG	S000491
CBFHV	578	(-)	RYCGAC	S000497
GATABOX	581	(+)	GATA	S000039
ROOTMOTIFTAPOX1	582	(+)	ATATT	S000098
POLLEN1LELAT52	589	(+)	AGAAA	S000245
EECCRCAH1	590	(-)	GANTTNC	S000494
GT1CONSENSUS	590	(+)	GRWAAW	S000198
ARR1AT	593	(-)	NGATT	S000454
GTGANTG10	595	(-)	GTGA	S000378
EBOXBNNAPA	596	(-)	CANNTG	S000144
MYCCONSENSUSAT	596	(-)	CANNTG	S000407
EBOXBNNAPA	596	(+)	CANNTG	S000144
MYCCONSENSUSAT	596	(+)	CANNTG	S000407
CACTFTPPCA1	596	(+)	YACT	S000449
RAV1AAT	605	(-)	CAACA	S000314
WBBOXPCWRKY1	620	(+)	TTTGACY	S000310
WBOXATNPR1	621	(+)	TTGAC	S000390
WBOXHVISO1	622	(+)	TGACT	S000442
WRKY71OS	622	(+)	TGAC	S000447
WBOXNTERF3	622	(+)	TGACY	S000457
POLLEN1LELAT52	628	(+)	AGAAA	S000245
GT1CONSENSUS	629	(+)	GRWAAW	S000198
GT1GMSCAM4	629	(+)	GAAAAA	S000453
WBOXHVISO1	636	(+)	TGACT	S000442
WRKY71OS	636	(+)	TGAC	S000447
WBOXNTERF3	636	(+)	TGACY	S000457
NTBBF1ARROLB	638	(+)	ACTTTA	S000273
DOFCOREZM	639	(-)	AAAG	S000265
TAAAGSTKST1	639	(-)	TAAAG	S000387
GATABOX	646	(+)	GATA	S000039
GT1CONSENSUS	646	(+)	GRWAAW	S000198
IBOXCORE	646	(+)	GATAA	S000199

ARR1AT	657	(-)	NGATT	S000454
TELOBOXATEEF1AA1	661	(+)	AAACCCTAA	S000308
UP2ATMSD	661	(+)	AAACCCTA	S000472
GT1CONSENSUS	672	(-)	GRWAAW	S000198
GT1GMSCAM4	672	(-)	GAAAAA	S000453
POLLEN1LELAT52	674	(-)	AGAAA	S000245
GT1CONSENSUS	692	(-)	GRWAAW	S000198
BIHD1OS	700	(-)	TGTCA	S000498
WRKY71OS	700	(+)	TGAC	S000447
CAATBOX1	704	(-)	CAAT	S000028
GTGANTG10	707	(+)	GTGA	S000378
ARR1AT	708	(+)	NGATT	S000454
GTGANTG10	714	(+)	GTGA	S000378
ACGTTBOX	722	(-)	AACGTT	S000132
ACGTTBOX	722	(+)	AACGTT	S000132
ACGTATERD1	723	(-)	ACGT	S000415
ACGTATERD1	723	(+)	ACGT	S000415
EECCRCAH1	734	(-)	GANTTNC	S000494
RBCSCONSENSUS	741	(-)	AATCCAA	S000127
ARR1AT	743	(+)	NGATT	S000454
CACTFTPPCA1	751	(-)	YACT	S000449
ANAERO1CONSENSUS	756	(-)	AAACAAA	S000477
AMYBOX1	761	(-)	TAACARA	S000020
MYBGAHV	761	(-)	TAACAAA	S000181
GAREAT	761	(-)	TAACAAR	S000439
RAV1AAT	779	(+)	CAACA	S000314
CAATBOX1	782	(+)	CAAT	S000028
RAV1AAT	788	(+)	CAACA	S000314
CACTFTPPCA1	799	(-)	YACT	S000449
S1FBOXSORPS1L21	804	(+)	ATGGTA	S000223
GT1CONSENSUS	806	(+)	GRWAAW	S000198
NTBBF1ARROLB	808	(-)	ACTTTA	S000273
TAAAGSTKST1	808	(+)	TAAAG	S000387
DOFCOREZM	809	(+)	AAAG	S000265
CACTFTPPCA1	811	(-)	YACT	S000449
CURECORECR	814	(-)	GTAC	S000493
CURECORECR	814	(+)	GTAC	S000493
LTRECOREATCOR15	828	(+)	CCGAC	S000153
PRECONSCRHSP70A	828	(+)	SCGAYNRNNNNNNNNNNNNNNNHD	S000506
CACTFTPPCA1	833	(+)	YACT	S000449
EBOXBNNAPA	841	(-)	CANNTG	S000144
MYCCONSENSUSAT	841	(-)	CANNTG	S000407
EBOXBNNAPA	841	(+)	CANNTG	S000144
MYCCONSENSUSAT	841	(+)	CANNTG	S000407
CACTFTPPCA1	848	(-)	YACT	S000449
PRECONSCRHSP70A	865	(+)	SCGAYNRNNNNNNNNNNNNNNNHD	S000506
GATABOX	867	(+)	GATA	S000039
CAATBOX1	897	(-)	CAAT	S000028
WBOXATNPR1	898	(+)	TTGAC	S000390
BIHD1OS	899	(-)	TGTCA	S000498
WRKY71OS	899	(+)	TGAC	S000447
CAATBOX1	902	(+)	CAAT	S000028
BOXIINTPATPB	904	(+)	ATAGAA	S000296
POLLEN1LELAT52	906	(+)	AGAAA	S000245
EECCRCAH1	907	(-)	GANTTNC	S000494
DOFCOREZM	917	(-)	AAAG	S000265
S1FSORPL21	927	(+)	ATGGTATT	S000215
S1FBOXSORPS1L21	927	(+)	ATGGTA	S000223
GT1CONSENSUS	932	(-)	GRWAAW	S000198
POLLEN1LELAT52	934	(-)	AGAAA	S000245
PYRIMIDINEBOXOSRAMY1A	942	(+)	CCTTTT	S000259
DOFCOREZM	943	(-)	AAAG	S000265
POLLEN1LELAT52	945	(-)	AGAAA	S000245
CACTFTPPCA1	954	(+)	YACT	S000449
GT1CONSENSUS	961	(-)	GRWAAW	S000198
IBOXCORE	962	(-)	GATAA	S000199
GATABOX	963	(-)	GATA	S000039
GTGANTG10	965	(-)	GTGA	S000378

CACTFTPPCA1	966	(+)	YACT	S000449
CACTFTPPCA1	970	(-)	YACT	S000449
PYRIMIDINEBOXOSRAMY1A	978	(-)	CCTTTT	S000259
DOFCOREZM	979	(+)	AAAG	S000265
NODCON2GM	989	(-)	CTCTT	S000462
OSE2ROOTNODULE	989	(-)	CTCTT	S000468
SURECOREATSULTR11	993	(+)	GAGAC	S000499
EECCRCAH1	1000	(+)	GANTTNC	S000494
-300ELEMENT	1005	(-)	TGHAAARK	S000122
PYRIMIDINEBOXOSRAMY1A	1005	(+)	CCTTTT	S000259
DOFCOREZM	1006	(-)	AAAG	S000265
GT1CONSENSUS	1015	(+)	GRWAAW	S000198
MYB1LEPR	1021	(-)	GTTAGTT	S000443
DOFCOREZM	1038	(+)	AAAG	S000265
CACTFTPPCA1	1040	(-)	YACT	S000449
NODCON2GM	1046	(+)	CTCTT	S000462
OSE2ROOTNODULE	1046	(+)	CTCTT	S000468
PREATPRODH	1053	(+)	ACTCAT	S000450
NODCON2GM	1059	(+)	CTCTT	S000462
OSE2ROOTNODULE	1059	(+)	CTCTT	S000468
DOFCOREZM	1061	(-)	AAAG	S000265
POLASIG1	1063	(-)	AATAAA	S000080
MARTBOX	1064	(+)	TTWTWTTWTT	S000067
TATABOX5	1064	(+)	TTATTT	S000203
POLASIG1	1068	(-)	AATAAA	S000080
TATABOX5	1069	(+)	TTATTT	S000203
GT1CONSENSUS	1071	(-)	GRWAAW	S000198
POLLEN1LELAT52	1073	(-)	AGAAA	S000245
DOFCOREZM	1076	(-)	AAAG	S000265
DOFCOREZM	1083	(-)	AAAG	S000265
LTRE1HVBLT49	1084	(-)	CCGAAA	S000250
DOFCOREZM	1105	(-)	AAAG	S000265
GT1CONSENSUS	1106	(-)	GRWAAW	S000198
GT1GMSCAM4	1106	(-)	GAAAAA	S000453
POLLEN1LELAT52	1108	(-)	AGAAA	S000245
WBOXNTCHN48	1111	(+)	CTGACY	S000508
WRKY71OS	1112	(+)	TGAC	S000447
WBOXNTERF3	1112	(+)	TGACY	S000457
CAATBOX1	1117	(-)	CAAT	S000028
WBOXHVISO1	1124	(-)	TGACT	S000442
WBOXNTERF3	1124	(-)	TGACY	S000457
WRKY71OS	1125	(-)	TGAC	S000447
GTGANTG10	1126	(-)	GTGA	S000378
POLLEN1LELAT52	1132	(+)	AGAAA	S000245
DOFCOREZM	1135	(+)	AAAG	S000265
WBOXHVISO1	1137	(-)	TGACT	S000442
WBOXNTERF3	1137	(-)	TGACY	S000457
WBOXNTCHN48	1137	(-)	CTGACY	S000508
WRKY71OS	1138	(-)	TGAC	S000447
CACTFTPPCA1	1141	(-)	YACT	S000449
POLLEN1LELAT52	1150	(+)	AGAAA	S000245
GT1CONSENSUS	1151	(+)	GRWAAW	S000198
WRKY71OS	1162	(+)	TGAC	S000447
WBOXNTERF3	1162	(+)	TGACY	S000457
ARR1AT	1175	(-)	NGATT	S000454
CACTFTPPCA1	1183	(-)	YACT	S000449
CURECORECR	1184	(-)	GTAC	S000493
CURECORECR	1184	(+)	GTAC	S000493
ROOTMOTIFTAPOX1	1190	(-)	ATATT	S000098
CACTFTPPCA1	1198	(-)	YACT	S000449
ERELEE4	1206	(+)	AWTTCAAA	S000037
ROOTMOTIFTAPOX1	1212	(-)	ATATT	S000098
GATABOX	1214	(-)	GATA	S000039
MARTBOX	1223	(-)	TTWTWTTWTT	S000067
MARTBOX	1224	(-)	TTWTWTTWTT	S000067
MARTBOX	1225	(-)	TTWTWTTWTT	S000067
MARTBOX	1226	(-)	TTWTWTTWTT	S000067
MARTBOX	1227	(-)	TTWTWTTWTT	S000067

ARR1AT	1235	(-)	NGATT	S000454
NODCON2GM	1239	(-)	CTCTT	S000462
OSE2ROOTNODULE	1239	(-)	CTCTT	S000468
POLLEN1LELAT52	1242	(+)	AGAAA	S000245
GT1CONSENSUS	1243	(+)	GRWAAW	S000198
GT1GMSCAM4	1243	(+)	GAAAAA	S000453
ANAERO1CONSENSUS	1247	(+)	AAACAAA	S000477
BP5OSWX	1256	(+)	CAACGTG	S000436
QARBNEXTA	1257	(+)	AACGTGT	S000244
T/GBOXATPIN2	1257	(+)	AACGTG	S000458
ABRERATCAL	1257	(+)	MACGYGB	S000507
ACGTATERD1	1258	(-)	ACGT	S000415
ABRELATERD1	1258	(+)	ACGTG	S000414
ACGTATERD1	1258	(+)	ACGT	S000415
GTGANTG10	1262	(+)	GTGA	S000378
GATABOX	1264	(+)	GATA	S000039
GATABOX	1266	(-)	GATA	S000039
MYBST1	1266	(-)	GGATA	S000180
TATCCAOSAMY	1266	(+)	TATCCA	S000403
TBOXATGAPB	1270	(-)	ACTTTG	S000383
DOFCOREZM	1271	(+)	AAAG	S000265
CACTFTPPCA1	1273	(-)	YACT	S000449
ARR1AT	1291	(-)	NGATT	S000454
GTGANTG10	1293	(-)	GTGA	S000378
CACTFTPPCA1	1294	(+)	YACT	S000449
POLASIG1	1311	(-)	AATAAA	S000080
TATABOX5	1312	(+)	TTATTT	S000203
SEBFCONSSTPR10A	1317	(+)	YTGTCWC	S000391
ARFAT	1318	(+)	TGTCTC	S000270
SURECOREATSULTR11	1319	(-)	GAGAC	S000499
NODCON2GM	1321	(+)	CTCTT	S000462
OSE2ROOTNODULE	1321	(+)	CTCTT	S000468
ARR1AT	1328	(-)	NGATT	S000454
TATABOX5	1332	(-)	TTATTT	S000203
GARE2OSREP1	1335	(+)	TAACGTA	S000420
ACGTATERD1	1337	(-)	ACGT	S000415
ACGTATERD1	1337	(+)	ACGT	S000415
ROOTMOTIFTAPOX1	1355	(+)	ATATT	S000098
TATABOX4	1358	(-)	TATATAA	S000111
TATAPVTRNALEU	1359	(-)	TTTATATA	S000340
TATABOX4	1359	(+)	TATATAA	S000111
TATABOX2	1361	(+)	TATAAAT	S000109
GT1CONSENSUS	1371	(+)	GRWAAW	S000198
GT1CONSENSUS	1372	(+)	GRWAAW	S000198
GT1GMSCAM4	1372	(+)	GAAAAA	S000453
DOFCOREZM	1375	(+)	AAAG	S000265
NODCON1GM	1375	(+)	AAAGAT	S000461
OSE1ROOTNODULE	1375	(+)	AAAGAT	S000467
ARR1AT	1377	(+)	NGATT	S000454
CAATBOX1	1379	(-)	CAAT	S000028
GATABOX	1382	(+)	GATA	S000039
GT1CONSENSUS	1382	(+)	GRWAAW	S000198
IBOXCORE	1382	(+)	GATAA	S000199
DOFCOREZM	1386	(+)	AAAG	S000265
POLLEN1LELAT52	1388	(+)	AGAAA	S000245
GT1CONSENSUS	1389	(+)	GRWAAW	S000198
GT1GMSCAM4	1389	(+)	GAAAAA	S000453
TATABOX5	1392	(-)	TTATTT	S000203
POLASIG3	1393	(+)	AATAAT	S000088
CACTFTPPCA1	1405	(-)	YACT	S000449
CURECORECR	1406	(-)	GTAC	S000493
CURECORECR	1406	(+)	GTAC	S000493
CACTFTPPCA1	1411	(+)	YACT	S000449
CPBCSPOR	1413	(-)	TATTAG	S000491
CAATBOX1	1420	(+)	CAAT	S000028
NODCON2GM	1425	(-)	CTCTT	S000462
OSE2ROOTNODULE	1425	(-)	CTCTT	S000468
PYRIMIDINEBOXHVEPB1	1429	(-)	TTTTTTCC	S000298

GT1CONSENSUS	1429	(+)	GRWAAW	S000198
GT1CONSENSUS	1430	(+)	GRWAAW	S000198
GT1GMSCAM4	1430	(+)	GAAAAA	S000453
POLASIG2	1435	(+)	AATTAAA	S000081
TAAAGSTKST1	1438	(+)	TAAAG	S000387
DOFCOREZM	1439	(+)	AAAG	S000265
ROOTMOTIFTAPOX1	1446	(-)	ATATT	S000098
RAV1AAT	1454	(-)	CAACA	S000314
GTGANTG10	1458	(+)	GTGA	S000378
WBOXHVISO1	1459	(+)	TGACT	S000442
WRKY71OS	1459	(+)	TGAC	S000447
WBOXNTERF3	1459	(+)	TGACY	S000457
GTGANTG10	1466	(+)	GTGA	S000378
ARR1AT	1472	(+)	NGATT	S000454
BIHD1OS	1478	(+)	TGTCA	S000498
WBOXATNPR1	1479	(-)	TTGAC	S000390
WRKY71OS	1479	(-)	TGAC	S000447
CAATBOX1	1481	(+)	CAAT	S000028
GT1CONSENSUS	1483	(-)	GRWAAW	S000198
IBOXCORE	1484	(-)	GATAA	S000199
SREATMSD	1484	(+)	TTATCC	S000470
GATABOX	1485	(-)	GATA	S000039
MYBST1	1485	(-)	GGATA	S000180
TATCCAOSAMY	1485	(+)	TATCCA	S000403
CACTFTPPCA1	1490	(-)	YACT	S000449
EBOXBNNAPA	1496	(-)	CANNTG	S000144
MYCCONSENSUSAT	1496	(-)	CANNTG	S000407
EBOXBNNAPA	1496	(+)	CANNTG	S000144
MYCCONSENSUSAT	1496	(+)	CANNTG	S000407
CACTFTPPCA1	1504	(+)	YACT	S000449
CACTFTPPCA1	1517	(+)	YACT	S000449
TATABOX5	1526	(+)	TTATTT	S000203
GT1CONSENSUS	1528	(-)	GRWAAW	S000198
POLLEN1LELAT52	1534	(+)	AGAAA	S000245
GT1CONSENSUS	1535	(+)	GRWAAW	S000198
GT1GMSCAM4	1535	(+)	GAAAAA	S000453
DOFCOREZM	1539	(+)	AAAG	S000265
GTGANTG10	1546	(+)	GTGA	S000378
MYB1AT	1550	(-)	WAACCA	S000408
ARR1AT	1558	(+)	NGATT	S000454
CACTFTPPCA1	1563	(-)	YACT	S000449
CURECORECR	1564	(-)	GTAC	S000493
CURECORECR	1564	(+)	GTAC	S000493
CAATBOX1	1567	(+)	CAAT	S000028
POLASIG3	1569	(-)	AATAAT	S000088
TATABOX5	1570	(+)	TTATTT	S000203
CACTFTPPCA1	1582	(-)	YACT	S000449
CACTFTPPCA1	1588	(-)	YACT	S000449
CACTFTPPCA1	1593	(-)	YACT	S000449
CACTFTPPCA1	1596	(-)	YACT	S000449
CBFHV	1613	(+)	RYCGAC	S000497
CARGNCAT	1618	(-)	CCWWWWWWWWGG	S000446
CCAATBOX1	1618	(+)	CCAAT	S000030
CARGNCAT	1618	(+)	CCWWWWWWWWGG	S000446
CARGCW8GAT	1619	(-)	CWWWWWWWWG	S000431
CAATBOX1	1619	(+)	CAAT	S000028
CARGCW8GAT	1619	(+)	CWWWWWWWWG	S000431
POLASIG1	1620	(+)	AATAAA	S000080
CAATBOX1	1625	(-)	CAAT	S000028
CCAATBOX1	1625	(-)	CCAAT	S000030
NODCON2GM	1630	(-)	CTCTT	S000462
OSE2ROOTNODULE	1630	(-)	CTCTT	S000468
GT1CONSENSUS	1634	(+)	GRWAAW	S000198
TATABOX5	1636	(-)	TTATTT	S000203
POLASIG1	1637	(+)	AATAAA	S000080
PYRIMIDINEBOXOSRAMY1A	1640	(-)	CCTTTT	S000259
DOFCOREZM	1641	(+)	AAAG	S000265
GATABOX	1652	(+)	GATA	S000039

IBOXCORE	1652	(+)	GATAA	S000199
MYB1AT	1654	(+)	WAACCA	S000408
REALPHALGLHCB21	1655	(+)	AACCAA	S000362
POLASIG3	1670	(+)	AATAAT	S000088
AMMORESIVDCRNIA1	1679	(+)	CGAACTT	S000375
GAREAT	1683	(-)	TAACAAR	S000439
CPBCSPOR	1699	(-)	TATTAG	S000491
POLASIG3	1701	(+)	AATAAT	S000088
CAATBOX1	1709	(-)	CAAT	S000028
CCAATBOX1	1709	(-)	CCAAT	S000030
ARR1AT	1714	(-)	NGATT	S000454
ROOTMOTIFTAPOX1	1726	(-)	ATATT	S000098
ROOTMOTIFTAPOX1	1727	(+)	ATATT	S000098
ROOTMOTIFTAPOX1	1732	(+)	ATATT	S000098
GTGANTG10	1736	(-)	GTGA	S000378
POLLEN1LELAT52	1741	(+)	AGAAA	S000245
UP2ATMSD	1743	(+)	AAACCCTA	S000472
GATABOX	1755	(-)	GATA	S000039
MYBST1	1755	(-)	GGATA	S000180
BOXIINTPATPB	1778	(-)	ATAGAA	S000296
SEF1MOTIF	1781	(-)	ATATTTAWW	S000006
TATABOX2	1781	(+)	TATAAAT	S000109
ROOTMOTIFTAPOX1	1785	(-)	ATATT	S000098
ROOTMOTIFTAPOX1	1786	(+)	ATATT	S000098
POLASIG2	1792	(+)	AATTAAA	S000081
SEF1MOTIF	1793	(-)	ATATTTAWW	S000006
TATABOXOSPAL	1794	(-)	TATTTAA	S000400
ROOTMOTIFTAPOX1	1797	(-)	ATATT	S000098
SEF1MOTIF	1798	(+)	ATATTTAWW	S000006
ROOTMOTIFTAPOX1	1798	(+)	ATATT	S000098
TATABOXOSPAL	1799	(+)	TATTTAA	S000400
POLASIG2	1801	(-)	AATTAAA	S000081
TAAAGSTKST1	1808	(+)	TAAAG	S000387
DOFCOREZM	1809	(+)	AAAG	S000265
GTGANTG10	1814	(+)	GTGA	S000378
CACTFTPPCA1	1817	(-)	YACT	S000449
POLASIG3	1820	(-)	AATAAT	S000088
TATABOX5	1821	(+)	TTATTT	S000203
TATABOXOSPAL	1822	(+)	TATTTAA	S000400
CURECORECR	1841	(-)	GTAC	S000493
CURECORECR	1841	(+)	GTAC	S000493
CACTFTPPCA1	1842	(+)	YACT	S000449
RAV1AAT	1846	(-)	CAACA	S000314
TATCCAOSAMY	1849	(-)	TATCCA	S000403
MYBST1	1850	(+)	GGATA	S000180
GATABOX	1851	(+)	GATA	S000039
UP2ATMSD	1853	(-)	AAACCCTA	S000472
MYBCORE	1870	(-)	CNGTTR	S000176
MYB2AT	1870	(+)	TAACTG	S000177
MYB2CONSENSUSAT	1870	(+)	YAACKG	S000409
ROOTMOTIFTAPOX1	1893	(+)	ATATT	S000098
CACTFTPPCA1	1898	(+)	YACT	S000449
WUSATAg	1903	(-)	TTAATGG	S000433
GT1CORE	1906	(-)	GGTTAA	S000125
SEF4MOTIFGM7S	1914	(-)	RTTTTTR	S000103
ARR1AT	1918	(-)	NGATT	S000454
CAATBOX1	1921	(+)	CAAT	S000028
ARR1AT	1922	(-)	NGATT	S000454
PYRIMIDINEBOXHVEPB1	1928	(-)	TTTTTTCC	S000298
GT1CONSENSUS	1928	(+)	GRWAAW	S000198
GT1CONSENSUS	1929	(+)	GRWAAW	S000198
GT1GMSCAM4	1929	(+)	GAAAAA	S000453
DOFCOREZM	1933	(+)	AAAG	S000265
NODCON2GM	1934	(-)	CTCTT	S000462
OSE2ROOTNODULE	1934	(-)	CTCTT	S000468
POLLEN1LELAT52	1937	(+)	AGAAA	S000245
CACTFTPPCA1	1944	(-)	YACT	S000449
GTGANTG10	1945	(+)	GTGA	S000378

TBOXATGAPB	1953	(+)	ACTTTG	S000383
DOFCOREZM	1954	(-)	AAAG	S000265
ANAERO1CONSENSUS	1955	(-)	AAACAAA	S000477
POLASIG3	1965	(-)	AATAAT	S000088
TATABOX5	1966	(+)	TTATTT	S000203
GT1CONSENSUS	1972	(+)	GRWAAW	S000198
LECPLEACS2	1983	(+)	TAAAATAT	S000465
ROOTMOTIFTAPOX1	1986	(-)	ATATT	S000098
BIHD1OS	1990	(-)	TGTCA	S000498
WRKY71OS	1990	(+)	TGAC	S000447
CAATBOX1	1993	(+)	CAAT	S000028
GT1CONSENSUS	1995	(-)	GRWAAW	S000198
POLLEN1LELAT52	1997	(-)	AGAAA	S000245
CANBNNAPA	2000	(+)	CNAACAC	S000148
CACTFTPPCA1	2004	(+)	YACT	S000449
TBOXATGAPB	2005	(+)	ACTTTG	S000383
DOFCOREZM	2006	(-)	AAAG	S000265
POLLEN1LELAT52	2020	(-)	AGAAA	S000245
POLLEN1LELAT52	2025	(+)	AGAAA	S000245
GTGANTG10	2032	(-)	GTGA	S000378
NODCON1GM	2036	(-)	AAAGAT	S000461
OSE1ROOTNODULE	2036	(-)	AAAGAT	S000467
DOFCOREZM	2038	(-)	AAAG	S000265
TAAAGSTKST1	2038	(-)	TAAAG	S000387
MYBCORE	2043	(+)	CNGTTR	S000176
ARR1AT	2047	(+)	NGATT	S000454
GT1CONSENSUS	2050	(-)	GRWAAW	S000198
IBOXCORE	2051	(-)	GATAA	S000199
GATABOX	2052	(-)	GATA	S000039
EBOXBNNAPA	2055	(-)	CANNTG	S000144
MYCCONSENSUSAT	2055	(-)	CANNTG	S000407
EBOXBNNAPA	2055	(+)	CANNTG	S000144
MYCCONSENSUSAT	2055	(+)	CANNTG	S000407
WBOXNTERF3	2060	(-)	TGACY	S000457
WRKY71OS	2061	(-)	TGAC	S000447
GTGANTG10	2062	(-)	GTGA	S000378
CACTFTPPCA1	2063	(+)	YACT	S000449
CACTFTPPCA1	2069	(-)	YACT	S000449
GT1CONSENSUS	2084	(-)	GRWAAW	S000198
IBOXCORE	2085	(-)	GATAA	S000199
GATABOX	2086	(-)	GATA	S000039
INRNTPSADB	2089	(+)	YTCANTYY	S000395
CAATBOX1	2091	(+)	CAAT	S000028
AMYBOX1	2094	(-)	TAACARA	S000020
MYBGAHV	2094	(-)	TAACAAA	S000181
GAREAT	2094	(-)	TAACAAR	S000439
IBOXCORE	2098	(-)	GATAA	S000199
GATABOX	2099	(-)	GATA	S000039
NODCON1GM	2100	(-)	AAAGAT	S000461
OSE1ROOTNODULE	2100	(-)	AAAGAT	S000467
DOFCOREZM	2102	(-)	AAAG	S000265
POLLEN1LELAT52	2103	(-)	AGAAA	S000245
DOFCOREZM	2106	(-)	AAAG	S000265
POLASIG1	2110	(-)	AATAAA	S000080
CAATBOX1	2117	(+)	CAAT	S000028
ARR1AT	2118	(-)	NGATT	S000454
NODCON1GM	2119	(-)	AAAGAT	S000461
OSE1ROOTNODULE	2119	(-)	AAAGAT	S000467
DOFCOREZM	2121	(-)	AAAG	S000265
GT1CONSENSUS	2122	(-)	GRWAAW	S000198
RAV1AAT	2127	(+)	CAACA	S000314
DOFCOREZM	2131	(+)	AAAG	S000265
CAATBOX1	2140	(+)	CAAT	S000028
GT1CONSENSUS	2142	(-)	GRWAAW	S000198
GT1CONSENSUS	2143	(-)	GRWAAW	S000198
POLLEN1LELAT52	2150	(+)	AGAAA	S000245
GT1CONSENSUS	2151	(+)	GRWAAW	S000198
TATABOX5	2153	(-)	TTATTT	S000203

SP8BFIBSP8BIB	2160	(+)	TACTATT	S000184
CACTFTPPCA1	2160	(+)	YACT	S000449
CARGCW8GAT	2162	(-)	CWWWWWWWWG	S000431
CARGCW8GAT	2162	(+)	CWWWWWWWWG	S000431
TATABOX3	2163	(+)	TATTAAT	S000110
CAATBOX1	2168	(-)	CAAT	S000028
WRKY71OS	2174	(+)	TGAC	S000447
WBOXNTERF3	2174	(+)	TGACY	S000457
EBOXBNNAPA	2178	(-)	CANNTG	S000144
MYCCONSENSUSAT	2178	(-)	CANNTG	S000407
EBOXBNNAPA	2178	(+)	CANNTG	S000144
MYCCONSENSUSAT	2178	(+)	CANNTG	S000407
-300ELEMENT	2182	(+)	TGHAAARK	S000122
GT1CONSENSUS	2183	(+)	GRWAAW	S000198
GT1GMSCAM4	2183	(+)	GAAAAA	S000453
ARR1AT	2187	(-)	NGATT	S000454
NODCON1GM	2200	(-)	AAAGAT	S000461
OSE1ROOTNODULE	2200	(-)	AAAGAT	S000467
DOFCOREZM	2202	(-)	AAAG	S000265
POLLEN1LELAT52	2203	(-)	AGAAA	S000245
GATABOX	2213	(-)	GATA	S000039
CACTFTPPCA1	2227	(+)	YACT	S000449
DOFCOREZM	2233	(+)	AAAG	S000265
POLLEN1LELAT52	2235	(+)	AGAAA	S000245
GT1CONSENSUS	2236	(+)	GRWAAW	S000198
GT1GMSCAM4	2236	(+)	GAAAAA	S000453
DOFCOREZM	2239	(+)	AAAG	S000265
GTGANTG10	2261	(-)	GTGA	S000378
ARR1AT	2266	(+)	NGATT	S000454
EECCRCAH1	2267	(+)	GANTTNC	S000494
DOFCOREZM	2273	(-)	AAAG	S000265
CCA1ATLHCB1	2279	(+)	AAMAATCT	S000149
ARR1AT	2282	(-)	NGATT	S000454
POLLEN1LELAT52	2287	(+)	AGAAA	S000245
GT1CONSENSUS	2288	(+)	GRWAAW	S000198
GT1GMSCAM4	2288	(+)	GAAAAA	S000453
DOFCOREZM	2292	(+)	AAAG	S000265
CACTFTPPCA1	2294	(-)	YACT	S000449
POLASIG2	2304	(+)	AATTAAA	S000081
TBOXATGAPB	2310	(+)	ACTTTG	S000383
DOFCOREZM	2311	(-)	AAAG	S000265
INRNTPSADB	2340	(-)	YTCANTYY	S000395
POLLEN1LELAT52	2353	(+)	AGAAA	S000245
GT1CONSENSUS	2354	(+)	GRWAAW	S000198
GT1GMSCAM4	2354	(+)	GAAAAA	S000453
NODCON2GM	2363	(+)	CTCTT	S000462
OSE2ROOTNODULE	2363	(+)	CTCTT	S000468
POLLEN1LELAT52	2370	(-)	AGAAA	S000245
ARR1AT	2381	(+)	NGATT	S000454
RYREPEATLEGUMINBOX	2390	(+)	CATGCAY	S000100
RYREPEATGMGY2	2390	(+)	CATGCAT	S000105
RYREPEATBNNAPA	2390	(+)	CATGCA	S000264
EBOXBNNAPA	2394	(-)	CANNTG	S000144
MYCCONSENSUSAT	2394	(-)	CANNTG	S000407
EBOXBNNAPA	2394	(+)	CANNTG	S000144
MYCCONSENSUSAT	2394	(+)	CANNTG	S000407
MYB1AT	2401	(+)	WAACCA	S000408
TATAPVTRNALEU	2415	(-)	TTTATATA	S000340
TATABOX4	2415	(+)	TATATAA	S000111
TAAAGSTKST1	2419	(+)	TAAAG	S000387
DOFCOREZM	2420	(+)	AAAG	S000265
SEF4MOTIFGM7S	2433	(+)	RTTTTTR	S000103
GT1CONSENSUS	2453	(+)	GRWAAW	S000198
GT1GMSCAM4	2453	(+)	GAAAAA	S000453
POLASIG2	2458	(+)	AATTAAA	S000081
TATABOX5	2463	(-)	TTATTT	S000203
POLASIG1	2464	(+)	AATAAA	S000080
DOFCOREZM	2468	(+)	AAAG	S000265

CACTFTPPCA1	2473	(-)	YACT	S000449
CURECORECR	2476	(-)	GTAC	S000493
CURECORECR	2476	(+)	GTAC	S000493
CAATBOX1	2487	(-)	CAAT	S000028
RAV1AAT	2491	(-)	CAACA	S000314
CAATBOX1	2506	(+)	CAAT	S000028
2SSEEDPROTBANAPA	2518	(+)	CAAACAC	S000143
CANBNNAPA	2518	(+)	CNAACAC	S000148
QARBNEXTA	2521	(-)	AACGTGT	S000244
ABRERATCAL	2521	(-)	MACGYGB	S000507
ABRELATERD1	2522	(-)	ACGTG	S000414
T/GBOXATPIN2	2522	(-)	AACGTG	S000458
ACGTATERD1	2523	(-)	ACGT	S000415
ACGTATERD1	2523	(+)	ACGT	S000415
ROOTMOTIFTAPOX1	2540	(-)	ATATT	S000098
BIHD1OS	2544	(+)	TGTCA	S000498
WRKY71OS	2545	(-)	TGAC	S000447
CACTFTPPCA1	2554	(-)	YACT	S000449
GTGANTG10	2555	(+)	GTGA	S000378
BIHD1OS	2556	(-)	TGTCA	S000498
WRKY71OS	2556	(+)	TGAC	S000447
GT1CONSENSUS	2562	(-)	GRWAAW	S000198
POLLEN1LELAT52	2564	(-)	AGAAA	S000245
TAAAGSTKST1	2569	(+)	TAAAG	S000387
DOFCOREZM	2570	(+)	AAAG	S000265
GT1CONSENSUS	2577	(-)	GRWAAW	S000198
GT1GMSCAM4	2577	(-)	GAAAAA	S000453
POLLEN1LELAT52	2579	(-)	AGAAA	S000245
DOFCOREZM	2582	(-)	AAAG	S000265
POLLEN1LELAT52	2584	(-)	AGAAA	S000245
DOFCOREZM	2587	(-)	AAAG	S000265
GT1CONSENSUS	2588	(-)	GRWAAW	S000198
GT1GMSCAM4	2588	(-)	GAAAAA	S000453
POLLEN1LELAT52	2590	(-)	AGAAA	S000245
DOFCOREZM	2593	(-)	AAAG	S000265
CARGCW8GAT	2593	(-)	CWWWWWWWWG	S000431
CARGCW8GAT	2593	(+)	CWWWWWWWWG	S000431
ARR1AT	2609	(-)	NGATT	S000454
GTGANTG10	2611	(-)	GTGA	S000378
NODCON2GM	2625	(-)	CTCTT	S000462
OSE2ROOTNODULE	2625	(-)	CTCTT	S000468
SURECOREATSULTR11	2627	(+)	GAGAC	S000499
RAV1AAT	2636	(+)	CAACA	S000314
GTGANTG10	2648	(+)	GTGA	S000378

//

©2011-2019 National Agriculture and Food Research Organization 1080284
