# Supplementary material for: Whole genome resequencing of four Italian sweet pepper landraces provides insights on sequence variation in genes of agronomic value
Source: Sci Rep. 2020 Jun 8;10:9189. doi: 10.1038/s41598-020-66053-2 (PMC7280500; doi:10.1038/s41598-020-66053-2)
Supplement: Supplementary file 1 — Supplementary File S1. [file 41598_2020_66053_MOESM1_ESM.zip › cuneo-quadrato_WUS_PLACE.pdf]

New PLACE

A Database of Plant Cis-acting Regulatory DNA Elements

Tue Jul 23 20:10:16 JST 2019

TTAACATAAATTCTCATTTTTACCCTTGAAGTYTCTTTTATCCAAAAAAAGAAAAARCTTMAGTCTCTTTTACTTGTCAAATCATAAGRGTAATTTTRRAAAARAATTCAAYGAWTMAYT

RESULTS OF YOUR SIGNAL SCAN SEARCH REQUEST

This result is the output of the new signal scan program which was completely rewritten
from a scratch by Akio Miyao ($Id: 649.pl,v 1.11 2016/04/20 08:43:39 miyao Exp $).

The original program of signal scan was reported in
Prestridge, D.S. (1991) SIGNAL SCAN: A computer program that scans DNA sequences for
eukaryotic transcriptional elements. CABIOS 7, 203-206.

191 base pairs

(+) = Current Strand
(-) = Opposite Strand

```
1      TTAACATAAATTCTCATTTTTACCCTTGAAGTNTCTTTTATCCAAAAAAA
                   (+) INRNTPSADB S000395 13 YTCANTYY
                      (+) SEF4MOTIFGM7S S000103 16 RTTTTTR
                         (-) GT1CONSENSUS S000198 19 GRWAAW
                                         (-) DOFCOREZM S000265 35 AAAG
                                           (-) GT1CONSENSUS S000198 37 GRWAAW
                                            (-) IBOXCORE S000199 38 GATAA
                                            (+) SREATMSD S000470 38 TTATCC
                                             (-) GATABOX S000039 39 GATA
                                             (-) MYBST1 S000180 39 GGATA
                                             (+) TATCCAOSAMY S000403 39 TATCCA
                                                      (+) DOFCOREZM S000265 48 AAAG
                                                        (+) POLLEN1LELAT52 S000245 50 AGAAA

51     GAAAAANCTTNAGTCTCTTTTACTTGTCAAATCATAAGNGTAATTTTNNA
       (+) GT1CONSENSUS S000198 51 GRWAAW
       (+) GT1GMSCAM4 S000453 51 GAAAAA
                   (-) SURECOREATSULTR11 S000499 63 GAGAC
                     (+) NODCON2GM S000462 65 CTCTT
                     (+) OSE2ROOTNODULE S000468 65 CTCTT
                       (-) DOFCOREZM S000265 67 AAAG
                           (+) CACTFTPPCA1 S000449 71 YACT
                               (+) BIHD1OS S000498 75 TGTCA
                                (-) WBOXATNPR1 S000390 76 TTGAC
                                (-) WRKY71OS S000447 76 TGAC
                                    (-) ARR1AT S000454 80 NGATT

101    AAANAATTCAANGANTNANTTATTTTGAAACANCANTAAATACTNNAACA
                          (+) TATABOX5 S000203 120 TTATTT
                                               (+) CACTFTPPCA1 S000449 141 YACT
                                                       (+) CAATBOX1 S000028 149 CAAT

151    ATTNATTTATTNNGAAATGGAGGGAGTATTTCTTTTAGCGC
            (-) POLASIG1 S000080 156 AATAAA
                               (-) CACTFTPPCA1 S000449 175 YACT
                                   (-) POLLEN1LELAT52 S000245 179 AGAAA
                                      (-) DOFCOREZM S000265 182 AAAG
```

Factor or Site Name	Loc.(Str.)	Signal Sequence	SITE #
INRNTPSADB	13 (+)	YTCANTYY	S000395
SEF4MOTIFGM7S	16 (+)	RTTTTTR	S000103
GT1CONSENSUS	19 (-)	GRWAAW	S000198
DOFCOREZM	35 (-)	AAAG	S000265
GT1CONSENSUS	37 (-)	GRWAAW	S000198
IBOXCORE	38 (-)	GATAA	S000199
SREATMSD	38 (+)	TTATCC	S000470
GATABOX	39 (-)	GATA	S000039
MYBST1	39 (-)	GGATA	S000180
TATCCAOSAMY	39 (+)	TATCCA	S000403
DOFCOREZM	48 (+)	AAAG	S000265
POLLEN1LELAT52	50 (+)	AGAAA	S000245
GT1CONSENSUS	51 (+)	GRWAAW	S000198
GT1GMSCAM4	51 (+)	GAAAAA	S000453
SURECOREATSULTR11	63 (-)	GAGAC	S000499
NODCON2GM	65 (+)	CTCTT	S000462

```
OSE2ROOTNODULE                  65 (+) CTCTT                          S000468
DOFCOREZM                       67 (-) AAAG                           S000265
CACTFTPPCA1                     71 (+) YACT                           S000449
BIHD1OS                         75 (+) TGTCA                          S000498
WBOXATNPR1                      76 (-) TTGAC                          S000390
WRKY71OS                        76 (-) TGAC                           S000447
ARR1AT                          80 (-) NGATT                          S000454
TATABOX5                       120 (+) TTATTT                         S000203
CACTFTPPCA1                    141 (+) YACT                           S000449
CAATBOX1                       149 (+) CAAT                           S000028
POLASIG1                       156 (-) AATAAA                         S000080
CACTFTPPCA1                    175 (-) YACT                           S000449
POLLEN1LELAT52                 179 (-) AGAAA                          S000245
DOFCOREZM                      182 (-) AAAG                           S000265
//
```

©2011-2019 National Agriculture and Food Research Organization 1077513
