# Supplementary material for: Whole genome resequencing of four Italian sweet pepper landraces provides insights on sequence variation in genes of agronomic value
Source: Sci Rep. 2020 Jun 8;10:9189. doi: 10.1038/s41598-020-66053-2 (PMC7280500; doi:10.1038/s41598-020-66053-2)
Supplement: Supplementary file 1 — Supplementary File S1. [file 41598_2020_66053_MOESM1_ESM.zip › cuneo_CLV3_PLACE_plus_boxes.pdf]

New PLACE

A Database of Plant Cis-acting Regulatory DNA Elements

```
Fri Jul 26 18:21:07 JST 2019

CTCACTTTTGTCTAGAAAGTAAGTAAAAAATATACTTATATGATCCTTAAAGAATCAACA
GATTTTACTATTTGCAGTAAAAGAGCCAGTCACATTTGGCAAATTTAATTAAAGACAAAT
TCTGTGTAATTTTGACTAGGAGTTAGTAAGGCCGCGCATTCTTATTAGGGGATTCTTTGT
TGGAATATAACTTACGATACCTGATGGTAGTTTAACCAAAAAATATGTATATATATATAT
ATATATATATTAGGGGATTCAAAGAGGATCAAGCTAGAATGTATGTGAAATATAATGAGA
TTCAAATTTGCGATCTACTTCAAATATAAACACAACAATTTATTTATATCATATTTGACA
CAGTATAATTTTCTGATAAAAGAGATCGATCCTGTTAAACATCCTTCATTATTGTAGATC
CGCCCCCATTTCCAATTGAGAAAGAGGGAACAAGCAAATGGGGGTGGATTTTACTAAATA
ATTTAAGCCATGTACTCCATCTCAATTGTGGAAGTATTCCACAAACGAACTTTTCTTGAG
TTGATCTCTCAGATAGTCAGTATTTCTAATAGTCGATATTTCAGAAAATCACTTGTTTTG
TTGAACAAAAAATTTGACTTAGAAAAAATGACTTTAGAGATAATAACTAAATCAAACCCT
AAATTTTTCTCTAAGGACCCCAAATTACCTATGACATTGTGATTTGTGATGGAAACGTTG
GTGCGGAATTCTTGGATTCCTAGTATTTTGTTTTGTTAGGGTAACATTACAACAATAACA
ACATATTCAGTGTATGGTAAAGTGTACGCAGTCCATCCCGACCACTACCTCAGATGAAGT
AGAGAGGTTGTTTCCGATAGACCCCTCGGCTCATGTTAGTGTAACATTGACAATAGAAAT
TCCTCTTTCCCTTCATGGTATTTTCTCCTCCTTTTCTTCTCTACTAGCATTATCACTAGT
AATAAAAAGGCTATGAAGAGAGACTAGAATTCCTTTTCATGGGTAATAACTAACATAAGG
AGTAAAGTAGCCTCTTGGACTCATCTCTTTTATTTTATTTTCTTTTGCCTTTCGGTGCTC
TATCTTCGACTTTTTCTGACCATTGTTCAGTCACACAGAAAAGTCAGTAAACTAAGAAAA
TGACATGACCACAACTAAAATCATGAAGTACATAATATGAGAGTAAGCAAATTCAAATAT
CTTTTAAAAAAAAAAAAAATCAAGAGAAAAAACAAATTCAACGTGTGATATCCAAAGTAA
ATTTTCTGATGTAATCACTCTAGCATCATCATTTTATTTTGTCTCTTCAAATCAAATAAC
GTAACTACATACATTATATTATATAAATGGAGGAAAAAGATTGATAAAAGAAAAATAATT
TATTAGTACACACTAATAACAATGAAGAGGAAAAAATTAAAGCAAAATATATATGTTGTG
ACTTGTGAGAGGATTATGTCAATTATCCAGTGGACAAATGCATACTCCTATAACCTACTT
ATTTTATTTTCAGAAAAAAGAATGTGATGGTTTCAAGATTAGTACAATTATTTATGATCA
GTGCAGTGCAGTAGTAGAACCTGATGAGGATCGACCAATAAATTGGAAGAGGAAATAAAA
GGAAAATGATAACCAAATTATGAGGAATAATTAACGAACTTGTTAAATGGTGGACTAATA
ATTATTGGAATCCTTAAAAAAATATTATATTCACAAGAAACCCTAGTCCTATCCTATGTA
TAGAGATCTAGTTCTATAAATATTAAATTAAATATTTAATTTAAAGCGTGAGTATTATTT
AATTTCTGCCTAGGTACTTGTTGGATAGGGTTTGAACAAAATTAACTGGATCTGAACTAA
AATCATATTTACTTCCATTAACCTTCAAAAATCAATCAAGGAAAAAAGAGAAAACAGTGA
AACACTTTGTTTTAAATTATTTGAAAATTAGCATAAAATATGACAATTTTCTAACACTTT
GAAGCAAAATTTCTAGAAACATCACATCTTTACGGTTGATTTATCAAATGGTCACTCAAG
TAATATATAGTTATTATCTCAATTTGTTATCTTTCTTTTTTATTACAATCTTTTCCAACA
AAGAAAACAATTTTCCGAGAAAATAACTACTATTAATTGAATGACCATCTGAAAAATCAT
CTCGGCATCTTTCTAACCCTATCTTATGGCTTCTACTAAAAAGAAAAAGAAGACTTCGGT
TTAATTTCACAAGATTTGCTTTCAAAAAATCTAGAAAAAAGTGGAACAAAATTAAACTTT
GAACTTGTATTCATCATCATAGACAAAATGAGAGCTCAGAAAAATGGCTCTTAATTTCTT
CAAACGATTCGATCATGCATTTGAAAACCAGCTTAGCATATATAAAGGGTTTTAAAATTT
TTGAAGGAGATGGATGAAAAAATTAAAATAAAAGAAGTGTACCGCAAGAATTGTGTTGCA
GAAGTTGCAATGAACATAGCAAACACGTTCCAAGGAAGATGAATATGTCATGTCTAGTGA
CATTTTTCTTAAAGGTGTTTTTCTTTTCTTTTTCTTTTTTTAGCCCTGGAATCACGGATC
GAAGAAGAGACGGATCAACAAGGAGCTGTGAACTGA


RESULTS OF YOUR SIGNAL SCAN SEARCH REQUEST

This result is the output of the new signal scan program which was completely rewritten
from a scratch by Akio Miyao ($Id: 649.pl,v 1.11 2016/04/20 08:43:39 miyao Exp $).

The original program of signal scan was reported in
Prestridge, D.S. (1991) SIGNAL SCAN: A computer program that scans DNA sequences for
eukaryotic transcriptional elements. CABIOS 7, 203-206.


2659 base pairs
```

(+) = Current Strand
(-) = Opposite Strand

1 CTCACTTTTGTCTAGAAAGTAAGTAAAAAATATACTTATATGATCCTTAA
(+) INRNTPSADB S000395 1 YTCANTYY
(-) GTGANTG10 S000378 2 GTGA
(+) CACTFTPPCA1 S000449 3 YACT
(-) DOFCOREZM S000265 5 AAAG
(+) POLLEN1LELAT52 S000245 14 AGAAA
(+) DOFCOREZM S000265 16 AAAG
(-) CACTFTPPCA1 S000449 18 YACT
(-) CACTFTPPCA1 S000449 22 YACT
(-) ROOTMOTIFTAPOX1 S000098 29 ATATT
(+) CACTFTPPCA1 S000449 33 YACT
(+) TAAAGSTKST1 S000387 48 TAAAG
(+) DOFCOREZM S000265 49 AAAG

51 AGAATCAACANGATTTTACTATTTGCAGTAAAAGAGCCAGTCACATTTGG
(-) ARR1AT S000454 53 NGATT
(+) RAV1AAT S000314 56 CAACA
(+) SP8BFIBSP8BIB S000184 67 TACTATT
(+) CACTFTPPCA1 S000449 67 YACT
(-) CACTFTPPCA1 S000449 77 YACT
(+) DOFCOREZM S000265 81 AAAG
(-) NODCON2GM S000462 82 CTCTT
(-) OSE2ROOTNODULE S000468 82 CTCTT
(-) WBOXHVISO1 S000442 89 TGACT
(-) WBOXNTERF3 S000457 89 TGACY
(-) WRKY71OS S000447 90 TGAC
(-) GTGANTG10 S000378 91 GTGA
(-) EBOXBNNAPA S000144 94 CANNTG
(-) MYCCONSENSUSAT S000407 94 CANNTG
(+) EBOXBNNAPA S000144 94 CANNTG
(+) MYCCONSENSUSAT S000407 94 CANNTG

101 CAAATTTAATTAAAGACAAATNTCTGTGTAATTTTGACTAGGAGTTAGTA
(-) POLASIG2 S000081 105 AATTAAA
(+) POLASIG2 S000081 108 AATTAAA
(+) TAAAGSTKST1 S000387 111 TAAAG
(+) DOFCOREZM S000265 112 AAAG
(+) WBBOXPCWRKY1 S000310 133 TTTGACY
(+) WBOXATNPR1 S000390 134 TTGAC
(+) WBOXHVISO1 S000442 135 TGACT
(+) WRKY71OS S000447 135 TGAC
(+) WBOXNTERF3 S000457 135 TGACY
(-) CACTFTPPCA1 S000449 147 YACT

151 AGGCCGCGCATTCTTATTAGGGGATTCTTTGTNTGGAATATAACTTACGA
(-) CGCGBOXAT S000501 154 VCGCGB
(+) CGCGBOXAT S000501 154 VCGCGB
(+) CPBCSPOR S000491 165 TATTAG
(+) ARR1AT S000454 172 NGATT
(-) XYLAT S000510 175 ACAAAGAA
(-) DOFCOREZM S000265 177 AAAG
(-) ROOTMOTIFTAPOX1 S000098 187 ATATT
(+) GATABOX S000039 199 GATA

201 TACCTGATGGTAGTTTAACCAAAAAATATGTATATATATATATNATATAT
(+) S1FBOXSORPS1L21 S000223 207 ATGGTA
(-) GT1CORE S000125 215 GGTTAA
(+) MYB1AT S000408 216 WAACCA
(+) REALPHALGLHCB21 S000362 217 AACCAA
(-) ROOTMOTIFTAPOX1 S000098 225 ATATT
(+) SORLREP3AT S000488 229 TGTATATAT

251 ATATTAGGGGATTCAAAGAGGATCAAGCTAGAATGTATGTGAAATATAAT
(+) ROOTMOTIFTAPOX1 S000098 251 ATATT
(+) CPBCSPOR S000491 252 TATTAG
(+) ARR1AT S000454 259 NGATT
(+) DOFCOREZM S000265 265 AAAG

```
                   (-) NODCON2GM S000462 266 CTCTT
                   (-) OSE2ROOTNODULE S000468 266 CTCTT
                                            (+) GTGANTG10 S000378 289 GTGA
                                                (-) ROOTMOTIFTAPOX1 S000098 293 ATATT

301     GAGANTTCAAATTTGCGATCTACTTCAAATATAAACACAACAATTTATTT
                        (+) CACTFTPPCA1 S000449 321 YACT
                               (-) ROOTMOTIFTAPOX1 S000098 328 ATATT
                                         (+) RAV1AAT S000314 338 CAACA
                                            (+) CAATBOX1 S000028 341 CAAT
                                               (-) POLASIG1 S000080 344 AATAAA
                                                (+) TATABOX5 S000203 345 TTATTT
                                                  (-) TATABOX2 S000109 347 TATAAAT

351     ATATCATATTTGACANCAGTATAATTTTCTGATAAAAGAGATCGATCCTG
         (-) GATABOX S000039 352 GATA
             (+) ROOTMOTIFTAPOX1 S000098 356 ATATT
                 (+) WBOXATNPR1 S000390 360 TTGAC
                  (-) BIHD1OS S000498 361 TGTCA
                  (+) WRKY71OS S000447 361 TGAC
                         (-) CACTFTPPCA1 S000449 368 YACT
                               (-) GT1CONSENSUS S000198 374 GRWAAW
                                 (-) POLLEN1LELAT52 S000245 376 AGAAA
                                      (+) GATABOX S000039 381 GATA
                                      (+) GT1CONSENSUS S000198 381 GRWAAW
                                      (+) IBOXCORE S000199 381 GATAA
                                          (+) DOFCOREZM S000265 385 AAAG
                                           (-) NODCON2GM S000462 386 CTCTT
                                           (-) OSE2ROOTNODULE S000468 386 CTCTT
                                                       (+) MYBCORE S000176 398 CNGTTR

401     TTAAACATCCTTCATTATTGTAGATCNCGCCCCCATTTCCAATTGAGAAA
                     (-) POLASIG3 S000088 414 AATAAT
                        (-) CAATBOX1 S000028 417 CAAT
                                         (-) GT1CONSENSUS S000198 435 GRWAAW
                                             (+) CCAATBOX1 S000030 439 CCAAT
                                              (-) EBOXBNNAPA S000144 440 CANNTG
                                              (-) MYCCONSENSUSAT S000407 440 CANNTG
                                              (+) CAATBOX1 S000028 440 CAAT
                                              (+) EBOXBNNAPA S000144 440 CANNTG
                                              (+) MYCCONSENSUSAT S000407 440 CANNTG
                                                (-) CAATBOX1 S000028 442 CAAT
                                                    (+) POLLEN1LELAT52 S000245 446 AGAAA
                                                      (+) DOFCOREZM S000265 448 AAAG
                                                       (-) NODCON2GM S000462 449 CTCTT
                                                       (-) OSE2ROOTNODULE S000468 449 CTCTT

451     GAGGGAACAAGCAAATGGGGGTGGATTTTACTAAATANATTTAAGCCATG
                   (-) EBOXBNNAPA S000144 462 CANNTG
                   (-) MYCCONSENSUSAT S000407 462 CANNTG
                   (+) EBOXBNNAPA S000144 462 CANNTG
                   (+) MYCCONSENSUSAT S000407 462 CANNTG
                              (+) ARR1AT S000454 473 NGATT
                                    (+) CACTFTPPCA1 S000449 479 YACT
                                                         (-) CURECORECR S000493 500 GTAC
                                                         (+) CURECORECR S000493 500 GTAC

501     TACTCCATCTCAATTGTGGAAGTATTCCACAAACGAACTTTTCTTGAGNT
        (+) CACTFTPPCA1 S000449 501 YACT
                  (-) EBOXBNNAPA S000144 511 CANNTG
                  (-) MYCCONSENSUSAT S000407 511 CANNTG
                  (+) CAATBOX1 S000028 511 CAAT
                  (+) EBOXBNNAPA S000144 511 CANNTG
                  (+) MYCCONSENSUSAT S000407 511 CANNTG
                    (-) CAATBOX1 S000028 513 CAAT
                            (-) CACTFTPPCA1 S000449 521 YACT
                                         (+) AMMORESIVDCRNIA1 S000375 534 CGAACTT
                                             (-) DOFCOREZM S000265 538 AAAG
                                               (-) POLLEN1LELAT52 S000245 540 AGAAA
```

```
551     TGATCTCTCAGATAGTCAGTATTTCTAATAGTCGATATTTCAGAAAATCA
                  (+) GATABOX S000039 561 GATA
                     (-) WBOXHVISO1 S000442 564 TGACT
                     (-) WBOXNTERF3 S000457 564 TGACY
                     (-) WBOXNTCHN48 S000508 564 CTGACY
                      (-) WRKY71OS S000447 565 TGAC
                         (-) CACTFTPPCA1 S000449 568 YACT
                             (-) POLLEN1LELAT52 S000245 572 AGAAA
                                (-) CPBCSPOR S000491 575 TATTAG
                                      (-) CBFHV S000497 581 RYCGAC
                                         (+) GATABOX S000039 584 GATA
                                          (+) ROOTMOTIFTAPOX1 S000098 585 ATATT
                                                 (+) POLLEN1LELAT52 S000245 592 AGAAA
                                                  (-) EECCRCAH1 S000494 593 GANTTNC
                                                  (+) GT1CONSENSUS S000198 593 GRWAAW
                                                     (-) ARR1AT S000454 596 NGATT
                                                       (-) GTGANTG10 S000378 598 GTGA
                                                        (-) EBOXBNNAPA S000144 599 CANNTG
                                                        (-) MYCCONSENSUSAT S000407 599 CANNTG
                                                        (+) EBOXBNNAPA S000144 599 CANNTG
                                                        (+) MYCCONSENSUSAT S000407 599 CANNTG
                                                        (+) CACTFTPPCA1 S000449 599 YACT

601     CTTGTTTTGNTTGAACAAAAAATTTGACTTAGAAAAAATGACTTTAGAGA
                              (+) WBBOXPCWRKY1 S000310 623 TTTGACY
                               (+) WBOXATNPR1 S000390 624 TTGAC
                                (+) WBOXHVISO1 S000442 625 TGACT
                                (+) WRKY71OS S000447 625 TGAC
                                (+) WBOXNTERF3 S000457 625 TGACY
                                      (+) POLLEN1LELAT52 S000245 631 AGAAA
                                       (+) GT1CONSENSUS S000198 632 GRWAAW
                                       (+) GT1GMSCAM4 S000453 632 GAAAAA
                                              (+) WBOXHVISO1 S000442 639 TGACT
                                              (+) WRKY71OS S000447 639 TGAC
                                              (+) WBOXNTERF3 S000457 639 TGACY
                                                (+) NTBBF1ARROLB S000273 641 ACTTTA
                                                 (-) DOFCOREZM S000265 642 AAAG
                                                 (-) TAAAGSTKST1 S000387 642 TAAAG
                                                        (+) GATABOX S000039 649 GATA
                                                        (+) GT1CONSENSUS S000198 649 GRWAAW
                                                        (+) IBOXCORE S000199 649 GATAA

651     TAATAACTAAATCAAACCCTNAAATTTTTCTCTAAGGACCCCAAATTACC
                 (-) ARR1AT S000454 660 NGATT
                                (-) GT1CONSENSUS S000198 675 GRWAAW
                                (-) GT1GMSCAM4 S000453 675 GAAAAA
                                  (-) POLLEN1LELAT52 S000245 677 AGAAA
                                                  (-) GT1CONSENSUS S000198 695 GRWAAW

701     TATGACATTGTGATTTGTGATGGAAACGTTGNGTGCGGAATTCTTGGATT
          (-) BIHD1OS S000498 703 TGTCA
          (+) WRKY71OS S000447 703 TGAC
              (-) CAATBOX1 S000028 707 CAAT
                 (+) GTGANTG10 S000378 710 GTGA
                  (+) ARR1AT S000454 711 NGATT
                        (+) GTGANTG10 S000378 717 GTGA
                                (-) ACGTTBOX S000132 725 AACGTT
                                (+) ACGTTBOX S000132 725 AACGTT
                                 (-) ACGTATERD1 S000415 726 ACGT
                                 (+) ACGTATERD1 S000415 726 ACGT
                                           (-) EECCRCAH1 S000494 737 GANTTNC
                                                  (-) RBCSCONSENSUS S000127 744 AATCCAA
                                                    (+) ARR1AT S000454 746 NGATT

751     CCTAGTATTTTGTTTTGTTAGGGTAACATTACAACAATAACANACATATT
           (-) CACTFTPPCA1 S000449 754 YACT
                (-) ANAERO1CONSENSUS S000477 759 AAACAAA
                     (-) AMYBOX1 S000020 764 TAACARA
                     (-) MYBGAHV S000181 764 TAACAAA
                     (-) GAREAT S000439 764 TAACAAR
```

(+) RAV1AAT S000314 782 CAACA
(+) CAATBOX1 S000028 785 CAAT
(+) AMYBOX1 S000020 788 TAACARA
(+) ROOTMOTIFTAPOX1 S000098 796 ATATT

801 CAGTGTATGGTAAAGTGTACGCAGTCCATCCCGACCACTACCTCAGATGA
(-) CACTFTPPCA1 S000449 802 YACT
(+) S1FBOXSORPS1L21 S000223 807 ATGGTA
(+) GT1CONSENSUS S000198 809 GRWAAW
(-) NTBBF1ARROLB S000273 811 ACTTTA
(+) TAAAGSTKST1 S000387 811 TAAAG
(+) DOFCOREZM S000265 812 AAAG
(-) CACTFTPPCA1 S000449 814 YACT
(-) CURECORECR S000493 817 GTAC
(+) CURECORECR S000493 817 GTAC
(+) LTRECOREATCOR15 S000153 831 CCGAC
(+) PRECONSCRHSP70A S000506 831 SCGAYNRNNNNNNNNNNNNNNNHD
(+) CACTFTPPCA1 S000449 836 YACT
(-) EBOXBNNAPA S000144 844 CANNTG
(-) MYCCONSENSUSAT S000407 844 CANNTG
(+) EBOXBNNAPA S000144 844 CANNTG
(+) MYCCONSENSUSAT S000407 844 CANNTG

851 AGTNAGAGAGGTTGTTTCCGATAGACCCCTCGGCTCATGTTAGTGTAACA
(+) PRECONSCRHSP70A S000506 868 SCGAYNRNNNNNNNNNNNNNNNHD
(+) GATABOX S000039 870 GATA
(-) CACTFTPPCA1 S000449 892 YACT
(-) CAATBOX1 S000028 900 CAAT

901 TTGACAATAGAAATNTCCTCTTTCCCTTCATGGTATTTTCTCCTCCTTTT
(+) WBOXATNPR1 S000390 901 TTGAC
(-) BIHD1OS S000498 902 TGTCA
(+) WRKY71OS S000447 902 TGAC
(+) CAATBOX1 S000028 905 CAAT
(+) BOXIINTPATPB S000296 907 ATAGAA
(+) POLLEN1LELAT52 S000245 909 AGAAA
(+) NODCON2GM S000462 918 CTCTT
(+) OSE2ROOTNODULE S000468 918 CTCTT
(-) DOFCOREZM S000265 920 AAAG
(+) S1FSORPL21 S000215 930 ATGGTATT
(+) S1FBOXSORPS1L21 S000223 930 ATGGTA
(-) GT1CONSENSUS S000198 935 GRWAAW
(-) POLLEN1LELAT52 S000245 937 AGAAA
(+) PYRIMIDINEBOXOSRAMY1A S000259 945 CCTTTT
(-) DOFCOREZM S000265 946 AAAG
(-) POLLEN1LELAT52 S000245 948 AGAAA

951 CTTCTCTACTAGCATTATCACTAGTNAATAAAAAGGCTATGAAGAGAGAC
(+) CACTFTPPCA1 S000449 957 YACT
(-) GT1CONSENSUS S000198 964 GRWAAW
(-) IBOXCORE S000199 965 GATAA
(-) GATABOX S000039 966 GATA
(-) GTGANTG10 S000378 968 GTGA
(+) CACTFTPPCA1 S000449 969 YACT
(+) POLASIG1 S000080 977 AATAAA
(-) PYRIMIDINEBOXOSRAMY1A S000259 981 CCTTTT
(+) DOFCOREZM S000265 982 AAAG
(-) NODCON2GM S000462 992 CTCTT
(-) OSE2ROOTNODULE S000468 992 CTCTT
(+) SURECOREATSULTR11 S000499 996 GAGAC

1001 TAGAATTCCTTTTCATGGGTAATAACTAACATAAGGNAGTAAAGTAGCCT
(+) EECCRCAH1 S000494 1003 GANTTNC
(-) -300ELEMENT S000122 1008 TGHAAARK
(+) PYRIMIDINEBOXOSRAMY1A S000259 1008 CCTTTT
(-) DOFCOREZM S000265 1009 AAAG
(+) GT1CONSENSUS S000198 1018 GRWAAW
(-) MYB1LEPR S000443 1024 GTTAGTT
(-) CACTFTPPCA1 S000449 1038 YACT
(-) NTBBF1ARROLB S000273 1040 ACTTTA

(+) TAAAGSTKST1 S000387 1040 TAAAG
(+) DOFCOREZM S000265 1041 AAAG
(-) CACTFTPPCA1 S000449 1043 YACT
(+) NODCON2GM S000462 1049 CTCTT
(+) OSE2ROOTNODULE S000468 1049 CTCTT

1051 CTTGGACTCATCTCTTTTATTTTATTTTCTTTTGCCTTTCGGTGCTCNTA
(+) PREATPRODH S000450 1056 ACTCAT
(+) NODCON2GM S000462 1062 CTCTT
(+) OSE2ROOTNODULE S000468 1062 CTCTT
(-) DOFCOREZM S000265 1064 AAAG
(-) POLASIG1 S000080 1066 AATAAA
(+) MARTBOX S000067 1067 TTWTWTTWTT
(+) TATABOX5 S000203 1067 TTATTT
(-) POLASIG1 S000080 1071 AATAAA
(+) TATABOX5 S000203 1072 TTATTT
(-) GT1CONSENSUS S000198 1074 GRWAAW
(-) POLLEN1LELAT52 S000245 1076 AGAAA
(-) DOFCOREZM S000265 1079 AAAG
(-) DOFCOREZM S000265 1086 AAAG
(-) LTRE1HVBLT49 S000250 1087 CCGAAA
(-) GATABOX S000039 1099 GATA

1101 TCTTCGACTTTTTCTGACCATTGTTCAGTCACACAGAAAAGTCAGTAAAC
(-) DOFCOREZM S000265 1108 AAAG
(-) GT1CONSENSUS S000198 1109 GRWAAW
(-) GT1GMSCAM4 S000453 1109 GAAAAA
(-) POLLEN1LELAT52 S000245 1111 AGAAA
(+) WBOXNTCHN48 S000508 1114 CTGACY
(+) WRKY71OS S000447 1115 TGAC
(+) WBOXNTERF3 S000457 1115 TGACY
(-) CAATBOX1 S000028 1120 CAAT
(-) WBOXHVISO1 S000442 1127 TGACT
(-) WBOXNTERF3 S000457 1127 TGACY
(-) WRKY71OS S000447 1128 TGAC
(-) GTGANTG10 S000378 1129 GTGA
(+) POLLEN1LELAT52 S000245 1135 AGAAA
(+) DOFCOREZM S000265 1138 AAAG
(-) WBOXHVISO1 S000442 1140 TGACT
(-) WBOXNTERF3 S000457 1140 TGACY
(-) WBOXNTCHN48 S000508 1140 CTGACY
(-) WRKY71OS S000447 1141 TGAC
(-) CACTFTPPCA1 S000449 1144 YACT

1151 TAAGAAAANTGACATGACCACAACTAAAATCATGAAGTACATAATATGAG
(+) POLLEN1LELAT52 S000245 1153 AGAAA
(+) GT1CONSENSUS S000198 1154 GRWAAW
(-) BIHD1OS S000498 1160 TGTCA
(+) WRKY71OS S000447 1160 TGAC
(+) WRKY71OS S000447 1165 TGAC
(+) WBOXNTERF3 S000457 1165 TGACY
(-) ARR1AT S000454 1178 NGATT
(-) CACTFTPPCA1 S000449 1186 YACT
(-) CURECORECR S000493 1187 GTAC
(+) CURECORECR S000493 1187 GTAC
(-) ROOTMOTIFTAPOX1 S000098 1193 ATATT

1201 AGTAAGCAAATTCAAATATNCTTTTAAAAAAAAAAAAAATCAAGAGAAAA
(-) CACTFTPPCA1 S000449 1201 YACT
(+) ERELEE4 S000037 1209 AWTTCAAA
(-) ROOTMOTIFTAPOX1 S000098 1215 ATATT
(-) DOFCOREZM S000265 1221 AAAG
(-) MARTBOX S000067 1226 TTWTWTTWTT
(-) MARTBOX S000067 1227 TTWTWTTWTT
(-) MARTBOX S000067 1228 TTWTWTTWTT
(-) MARTBOX S000067 1229 TTWTWTTWTT
(-) MARTBOX S000067 1230 TTWTWTTWTT
(-) ARR1AT S000454 1238 NGATT
(-) NODCON2GM S000462 1242 CTCTT
(-) OSE2ROOTNODULE S000468 1242 CTCTT

(+) POLLEN1LELAT52 S000245 1245 AGAAA
(+) GT1CONSENSUS S000198 1246 GRWAAW
(+) GT1GMSCAM4 S000453 1246 GAAAAA
(+) ANAERO1CONSENSUS S000477 1250 AAACAAA

1251 AACAAATTCAACGTGTGATATCCAAAGTAANATTTTCTGATGTAATCACT
(+) BP5OSWX S000436 1259 CAACGTG
(+) QARBNEXTA S000244 1260 AACGTGT
(+) T/GBOXATPIN2 S000458 1260 AACGTG
(+) ABRERATCAL S000507 1260 MACGYGB
(-) ACGTATERD1 S000415 1261 ACGT
(+) ABRELATERD1 S000414 1261 ACGTG
(+) ACGTATERD1 S000415 1261 ACGT
(+) GTGANTG10 S000378 1265 GTGA
(+) GATABOX S000039 1267 GATA
(-) GATABOX S000039 1269 GATA
(-) MYBST1 S000180 1269 GGATA
(+) TATCCAOSAMY S000403 1269 TATCCA
(-) TBOXATGAPB S000383 1273 ACTTTG
(+) DOFCOREZM S000265 1274 AAAG
(-) CACTFTPPCA1 S000449 1276 YACT
(-) GT1CONSENSUS S000198 1282 GRWAAW
(-) POLLEN1LELAT52 S000245 1284 AGAAA
(-) ARR1AT S000454 1294 NGATT
(-) GTGANTG10 S000378 1296 GTGA
(+) CACTFTPPCA1 S000449 1297 YACT

1301 CTAGCATCATCATTTTATTTTGTCTCTTCAAATCAAATAACNGTAACTAC
(-) POLASIG1 S000080 1314 AATAAA
(+) TATABOX5 S000203 1315 TTATTT
(+) SEBFCONSSTPR10A S000391 1320 YTGTCWC
(+) ARFAT S000270 1321 TGTCTC
(-) SURECOREATSULTR11 S000499 1322 GAGAC
(+) NODCON2GM S000462 1324 CTCTT
(+) OSE2ROOTNODULE S000468 1324 CTCTT
(-) ARR1AT S000454 1331 NGATT
(-) TATABOX5 S000203 1335 TTATTT
(+) MYB2CONSENSUSAT S000409 1338 YAACKG

1351 ATACATTATATTATATAAATGGAGGAAAAAGATTGATAAAAGAAAAATAA
(+) ROOTMOTIFTAPOX1 S000098 1358 ATATT
(-) TATABOX4 S000111 1361 TATATAA
(-) TATAPVTRNALEU S000340 1362 TTTATATA
(+) TATABOX4 S000111 1362 TATATAA
(+) TATABOX2 S000109 1364 TATAAAT
(+) GT1CONSENSUS S000198 1374 GRWAAW
(+) GT1CONSENSUS S000198 1375 GRWAAW
(+) GT1GMSCAM4 S000453 1375 GAAAAA
(+) DOFCOREZM S000265 1378 AAAG
(+) NODCON1GM S000461 1378 AAAGAT
(+) OSE1ROOTNODULE S000467 1378 AAAGAT
(+) ARR1AT S000454 1380 NGATT
(-) CAATBOX1 S000028 1382 CAAT
(+) GATABOX S000039 1385 GATA
(+) GT1CONSENSUS S000198 1385 GRWAAW
(+) IBOXCORE S000199 1385 GATAA
(+) DOFCOREZM S000265 1389 AAAG
(+) POLLEN1LELAT52 S000245 1391 AGAAA
(+) GT1CONSENSUS S000198 1392 GRWAAW
(+) GT1GMSCAM4 S000453 1392 GAAAAA
(-) TATABOX5 S000203 1395 TTATTT
(+) POLASIG3 S000088 1396 AATAAT

1401 TTNTATTAGTACACACTAATAACAATGAAGAGGAAAAAATTAAAGCAAAA
(+) CPBCSPOR S000491 1404 TATTAG
(-) CACTFTPPCA1 S000449 1408 YACT
(-) CURECORECR S000493 1409 GTAC
(+) CURECORECR S000493 1409 GTAC
(+) CACTFTPPCA1 S000449 1414 YACT
(-) CPBCSPOR S000491 1416 TATTAG

(+) CAATBOX1 S000028 1423 CAAT
(-) NODCON2GM S000462 1428 CTCTT
(-) OSE2ROOTNODULE S000468 1428 CTCTT
(-) PYRIMIDINEBOXHVEPB1 S000298 1432 TTTTTTCC
(+) GT1CONSENSUS S000198 1432 GRWAAW
(+) GT1CONSENSUS S000198 1433 GRWAAW
(+) GT1GMSCAM4 S000453 1433 GAAAAA
(+) POLASIG2 S000081 1438 AATTAAA
(+) TAAAGSTKST1 S000387 1441 TAAAG
(+) DOFCOREZM S000265 1442 AAAG
(-) ROOTMOTIFTAPOX1 S000098 1449 ATATT

1451 TATATATGTTGTGNACTTGTGAGAGGATTATGTCAATTATCCAGTGGACA
(-) RAV1AAT S000314 1457 CAACA
(+) GTGANTG10 S000378 1469 GTGA
(+) ARR1AT S000454 1475 NGATT
(+) BIHD1OS S000498 1481 TGTCA
(-) WBOXATNPR1 S000390 1482 TTGAC
(-) WRKY71OS S000447 1482 TGAC
(+) CAATBOX1 S000028 1484 CAAT
(-) GT1CONSENSUS S000198 1486 GRWAAW
(-) IBOXCORE S000199 1487 GATAA
(+) SREATMSD S000470 1487 TTATCC
(-) GATABOX S000039 1488 GATA
(-) MYBST1 S000180 1488 GGATA
(+) TATCCAOSAMY S000403 1488 TATCCA
(-) CACTFTPPCA1 S000449 1493 YACT
(-) EBOXBNNAPA S000144 1499 CANNTG
(-) MYCCONSENSUSAT S000407 1499 CANNTG
(+) EBOXBNNAPA S000144 1499 CANNTG
(+) MYCCONSENSUSAT S000407 1499 CANNTG

1501 AATGCATACTCCTATAACCTACTTNATTTTATTTTCAGAAAAAAGAATGT
(+) CACTFTPPCA1 S000449 1507 YACT
(+) CACTFTPPCA1 S000449 1520 YACT
(-) POLASIG1 S000080 1528 AATAAA
(+) TATABOX5 S000203 1529 TTATTT
(-) GT1CONSENSUS S000198 1531 GRWAAW
(+) POLLEN1LELAT52 S000245 1537 AGAAA
(+) GT1CONSENSUS S000198 1538 GRWAAW
(+) GT1GMSCAM4 S000453 1538 GAAAAA
(+) DOFCOREZM S000265 1542 AAAG
(+) GTGANTG10 S000378 1549 GTGA

1551 GATGGTTTCAAGATTAGTACAATTATTTATGATCANGTGCAGTGCAGTAG
(-) MYB1AT S000408 1553 WAACCA
(+) ARR1AT S000454 1561 NGATT
(-) CACTFTPPCA1 S000449 1566 YACT
(-) CURECORECR S000493 1567 GTAC
(+) CURECORECR S000493 1567 GTAC
(+) CAATBOX1 S000028 1570 CAAT
(-) POLASIG3 S000088 1572 AATAAT
(+) TATABOX5 S000203 1573 TTATTT
(-) CACTFTPPCA1 S000449 1591 YACT
(-) CACTFTPPCA1 S000449 1596 YACT
(-) CACTFTPPCA1 S000449 1599 YACT

1601 TAGAACCTGATGAGGATCGACCAATAAATTGGAAGAGGAAATAAAANGGA
(+) CBFHV S000497 1616 RYCGAC
(-) CARGNCAT S000446 1621 CCWWWWWWWWGG
(+) CCAATBOX1 S000030 1621 CCAAT
(+) CARGNCAT S000446 1621 CCWWWWWWWWGG
(-) CARGCW8GAT S000431 1622 CWWWWWWWWG
(+) CAATBOX1 S000028 1622 CAAT
(+) CARGCW8GAT S000431 1622 CWWWWWWWWG
(+) POLASIG1 S000080 1623 AATAAA
(-) CAATBOX1 S000028 1628 CAAT
(-) CCAATBOX1 S000030 1628 CCAAT
(-) NODCON2GM S000462 1633 CTCTT
(-) OSE2ROOTNODULE S000468 1633 CTCTT

```
(+) GT1CONSENSUS S000198 1637 GRWAAW
(-) TATABOX5 S000203 1639 TTATTT
(+) POLASIG1 S000080 1640 AATAAA
(+) GT1CONSENSUS S000198 1648 GRWAAW
(+) GT1CONSENSUS S000198 1649 GRWAAW

1651 AAATGATAACCAAATTATGAGGAATAATTAACGAACTTGTTAAATGGTGG
(+) GATABOX S000039 1655 GATA
(+) IBOXCORE S000199 1655 GATAA
(+) MYB1AT S000408 1657 WAACCA
(+) REALPHALGLHCB21 S000362 1658 AACCAA
(+) POLASIG3 S000088 1673 AATAAT
(+) AMMORESIVDCRNIA1 S000375 1682 CGAACTT
(-) GAREAT S000439 1686 TAACAAR

1701 ACTAATANATTATTGGAATCCTTAAAAAAATATTATATTCACAAGAAACC
(-) CPBCSPOR S000491 1702 TATTAG
(-) POLASIG3 S000088 1709 AATAAT
(-) CAATBOX1 S000028 1712 CAAT
(-) CCAATBOX1 S000030 1712 CCAAT
(-) ARR1AT S000454 1717 NGATT
(-) ROOTMOTIFTAPOX1 S000098 1729 ATATT
(+) ROOTMOTIFTAPOX1 S000098 1730 ATATT
(+) ROOTMOTIFTAPOX1 S000098 1735 ATATT
(-) GTGANTG10 S000378 1739 GTGA
(+) POLLEN1LELAT52 S000245 1744 AGAAA
(+) UP2ATMSD S000472 1746 AAACCCTA

1751 CTAGTCCTATCCTATGTANTAGAGATCTAGTTCTATAAATATTAAATTAA
(-) GATABOX S000039 1758 GATA
(-) MYBST1 S000180 1758 GGATA
(-) BOXIINTPATPB S000296 1781 ATAGAA
(-) SEF1MOTIF S000006 1784 ATATTTAWW
(+) TATABOX2 S000109 1784 TATAAAT
(-) ROOTMOTIFTAPOX1 S000098 1788 ATATT
(+) ROOTMOTIFTAPOX1 S000098 1789 ATATT
(+) POLASIG2 S000081 1795 AATTAAA
(-) SEF1MOTIF S000006 1796 ATATTTAWW
(-) TATABOXOSPAL S000400 1797 TATTTAA
(-) ROOTMOTIFTAPOX1 S000098 1800 ATATT

1801 ATATTTAATTTAAAGCGTGAGTATTATTTNAATTTCTGCCTAGGTACTTG
(+) SEF1MOTIF S000006 1801 ATATTTAWW
(+) ROOTMOTIFTAPOX1 S000098 1801 ATATT
(+) TATABOXOSPAL S000400 1802 TATTTAA
(-) POLASIG2 S000081 1804 AATTAAA
(+) TAAAGSTKST1 S000387 1811 TAAAG
(+) DOFCOREZM S000265 1812 AAAG
(+) GTGANTG10 S000378 1817 GTGA
(-) CACTFTPPCA1 S000449 1820 YACT
(-) POLASIG3 S000088 1823 AATAAT
(+) TATABOX5 S000203 1824 TTATTT
(-) POLLEN1LELAT52 S000245 1833 AGAAA
(-) CURECORECR S000493 1844 GTAC
(+) CURECORECR S000493 1844 GTAC
(+) CACTFTPPCA1 S000449 1845 YACT
(-) RAV1AAT S000314 1849 CAACA

1851 TTGGATAGGGTTTGAACAAAATTAACTGGATCTGAACTAANAATCATATT
(-) TATCCAOSAMY S000403 1852 TATCCA
(+) MYBST1 S000180 1853 GGATA
(+) GATABOX S000039 1854 GATA
(-) UP2ATMSD S000472 1856 AAACCCTA
(-) MYBCORE S000176 1873 CNGTTR
(+) MYB2AT S000177 1873 TAACTG
(+) MYB2CONSENSUSAT S000409 1873 YAACKG
(-) ARR1AT S000454 1892 NGATT
(+) ROOTMOTIFTAPOX1 S000098 1896 ATATT

1901 TACTTCCATTAACCTTCAAAAATCAATCAAGGAAAAAAGAGAAAACAGTG
```

(+) CACTFTPPCA1 S000449 1901 YACT
(-) WUSATAg S000433 1906 TTAATGG
(-) GT1CORE S000125 1909 GGTTAA
(-) SEF4MOTIFGM7S S000103 1917 RTTTTTR
(-) ARR1AT S000454 1921 NGATT
(+) CAATBOX1 S000028 1924 CAAT
(-) ARR1AT S000454 1925 NGATT
(-) PYRIMIDINEBOXHVEPB1 S000298 1931 TTTTTTCC
(+) GT1CONSENSUS S000198 1931 GRWAAW
(+) GT1CONSENSUS S000198 1932 GRWAAW
(+) GT1GMSCAM4 S000453 1932 GAAAAA
(+) DOFCOREZM S000265 1936 AAAG
(-) NODCON2GM S000462 1937 CTCTT
(-) OSE2ROOTNODULE S000468 1937 CTCTT
(+) POLLEN1LELAT52 S000245 1940 AGAAA
(-) CACTFTPPCA1 S000449 1947 YACT
(+) GTGANTG10 S000378 1948 GTGA

1951 ANAACACTTTGTTTTAAATTATTTGAAAATTAGCATAAAATATGACAATT
(+) CACTFTPPCA1 S000449 1955 YACT
(+) TBOXATGAPB S000383 1956 ACTTTG
(-) DOFCOREZM S000265 1957 AAAG
(-) ANAERO1CONSENSUS S000477 1958 AAACAAA
(-) POLASIG3 S000088 1968 AATAAT
(+) TATABOX5 S000203 1969 TTATTT
(+) GT1CONSENSUS S000198 1975 GRWAAW
(+) LECPLEACS2 S000465 1986 TAAAATAT
(-) ROOTMOTIFTAPOX1 S000098 1989 ATATT
(-) BIHD1OS S000498 1993 TGTCA
(+) WRKY71OS S000447 1993 TGAC
(+) CAATBOX1 S000028 1996 CAAT
(-) GT1CONSENSUS S000198 1998 GRWAAW
(-) POLLEN1LELAT52 S000245 2000 AGAAA

2001 TTCTAACACTTTNGAAGCAAAATTTCTAGAAACATCACATCTTTACGGTT
(+) CANBNNAPA S000148 2003 CNAACAC
(+) CACTFTPPCA1 S000449 2007 YACT
(-) DOFCOREZM S000265 2009 AAAG
(-) POLLEN1LELAT52 S000245 2023 AGAAA
(+) POLLEN1LELAT52 S000245 2028 AGAAA
(-) GTGANTG10 S000378 2035 GTGA
(-) NODCON1GM S000461 2039 AAAGAT
(-) OSE1ROOTNODULE S000467 2039 AAAGAT
(-) DOFCOREZM S000265 2041 AAAG
(-) TAAAGSTKST1 S000387 2041 TAAAG
(+) MYBCORE S000176 2046 CNGTTR
(+) ARR1AT S000454 2050 NGATT

2051 GATTTATCAAATGGTCACTCAAGNTAATATATAGTTATTATCTCAATTTG
(-) GT1CONSENSUS S000198 2053 GRWAAW
(-) IBOXCORE S000199 2054 GATAA
(-) GATABOX S000039 2055 GATA
(-) EBOXBNNAPA S000144 2058 CANNTG
(-) MYCCONSENSUSAT S000407 2058 CANNTG
(+) EBOXBNNAPA S000144 2058 CANNTG
(+) MYCCONSENSUSAT S000407 2058 CANNTG
(-) WBOXNTERF3 S000457 2063 TGACY
(-) WRKY71OS S000447 2064 TGAC
(-) GTGANTG10 S000378 2065 GTGA
(+) CACTFTPPCA1 S000449 2066 YACT
(-) ROOTMOTIFTAPOX1 S000098 2076 ATATT
(-) GT1CONSENSUS S000198 2087 GRWAAW
(-) IBOXCORE S000199 2088 GATAA
(-) GATABOX S000039 2089 GATA
(+) INRNTPSADB S000395 2092 YTCANTYY
(+) CAATBOX1 S000028 2094 CAAT
(-) AMYBOX1 S000020 2097 TAACARA
(-) MYBGAHV S000181 2097 TAACAAA
(-) GAREAT S000439 2097 TAACAAR

2101 TTATCTTTCTTTTTTATTACAATCTTTTCCAACANAAGAAAACAATTTTC
(-) IBOXCORE S000199 2101 GATAA
(-) GATABOX S000039 2102 GATA
(-) NODCON1GM S000461 2103 AAAGAT
(-) OSE1ROOTNODULE S000467 2103 AAAGAT
(-) DOFCOREZM S000265 2105 AAAG
(-) POLLEN1LELAT52 S000245 2106 AGAAA
(-) DOFCOREZM S000265 2109 AAAG
(-) POLASIG1 S000080 2113 AATAAA
(+) CAATBOX1 S000028 2120 CAAT
(-) ARR1AT S000454 2121 NGATT
(-) NODCON1GM S000461 2122 AAAGAT
(-) OSE1ROOTNODULE S000467 2122 AAAGAT
(-) DOFCOREZM S000265 2124 AAAG
(-) GT1CONSENSUS S000198 2125 GRWAAW
(+) RAV1AAT S000314 2130 CAACA
(+) POLLEN1LELAT52 S000245 2137 AGAAA
(+) CAATBOX1 S000028 2143 CAAT
(-) GT1CONSENSUS S000198 2145 GRWAAW
(-) GT1CONSENSUS S000198 2146 GRWAAW

2151 CGAGAAAATAACTACTATTAATTGAATGACCATCTGAAAAATCATNCTCG
(+) POLLEN1LELAT52 S000245 2153 AGAAA
(+) GT1CONSENSUS S000198 2154 GRWAAW
(-) TATABOX5 S000203 2156 TTATTT
(+) SP8BFIBSP8BIB S000184 2163 TACTATT
(+) CACTFTPPCA1 S000449 2163 YACT
(-) CARGCW8GAT S000431 2165 CWWWWWWWWG
(+) CARGCW8GAT S000431 2165 CWWWWWWWWG
(+) TATABOX3 S000110 2166 TATTAAT
(-) CAATBOX1 S000028 2171 CAAT
(+) WRKY71OS S000447 2177 TGAC
(+) WBOXNTERF3 S000457 2177 TGACY
(-) EBOXBNNAPA S000144 2181 CANNTG
(-) MYCCONSENSUSAT S000407 2181 CANNTG
(+) EBOXBNNAPA S000144 2181 CANNTG
(+) MYCCONSENSUSAT S000407 2181 CANNTG
(+) -300ELEMENT S000122 2185 TGHAAARK
(+) GT1CONSENSUS S000198 2186 GRWAAW
(+) GT1GMSCAM4 S000453 2186 GAAAAA
(-) ARR1AT S000454 2190 NGATT

2201 GCATCTTTCTAACCCTATCTTATGGCTTCTACTAAAAAGAAAAAGAAGAC
(-) NODCON1GM S000461 2203 AAAGAT
(-) OSE1ROOTNODULE S000467 2203 AAAGAT
(-) DOFCOREZM S000265 2205 AAAG
(-) POLLEN1LELAT52 S000245 2206 AGAAA
(-) GATABOX S000039 2216 GATA
(+) CACTFTPPCA1 S000449 2230 YACT
(+) DOFCOREZM S000265 2236 AAAG
(+) POLLEN1LELAT52 S000245 2238 AGAAA
(+) GT1CONSENSUS S000198 2239 GRWAAW
(+) GT1GMSCAM4 S000453 2239 GAAAAA
(+) DOFCOREZM S000265 2242 AAAG

2251 TTCGGTNTTAATTTCACAAGATTTGCTTTCAAAAAATCTAGAAAAAAGTG
(-) GTGANTG10 S000378 2264 GTGA
(+) ARR1AT S000454 2269 NGATT
(+) EECCRCAH1 S000494 2270 GANTTNC
(-) DOFCOREZM S000265 2276 AAAG
(+) CCA1ATLHCB1 S000149 2282 AAMAATCT
(-) ARR1AT S000454 2285 NGATT
(+) POLLEN1LELAT52 S000245 2290 AGAAA
(+) GT1CONSENSUS S000198 2291 GRWAAW
(+) GT1GMSCAM4 S000453 2291 GAAAAA
(+) DOFCOREZM S000265 2295 AAAG
(-) CACTFTPPCA1 S000449 2297 YACT

2301 GAACAAAATTAAACTTTNGAACTTGTATTCATCATCATAGACAAAATGAG
(+) POLASIG2 S000081 2307 AATTAAA

(-) DOFCOREZM S000265 2314 AAAG
(-) INRNTPSADB S000395 2343 YTCANTYY

2351 AGCTCAGAAAAATGGCTCTTAATTTCTTNCAAACGATTCGATCATGCATT
(+) POLLEN1LELAT52 S000245 2356 AGAAA
(+) GT1CONSENSUS S000198 2357 GRWAAW
(+) GT1GMSCAM4 S000453 2357 GAAAAA
(+) NODCON2GM S000462 2366 CTCTT
(+) OSE2ROOTNODULE S000468 2366 CTCTT
(-) POLLEN1LELAT52 S000245 2373 AGAAA
(+) ARR1AT S000454 2384 NGATT
(+) RYREPEATLEGUMINBOX S000100 2393 CATGCAY
(+) RYREPEATGMGY2 S000105 2393 CATGCAT
(+) RYREPEATBNNAPA S000264 2393 CATGCA
(-) EBOXBNNAPA S000144 2397 CANNTG
(-) MYCCONSENSUSAT S000407 2397 CANNTG
(+) EBOXBNNAPA S000144 2397 CANNTG
(+) MYCCONSENSUSAT S000407 2397 CANNTG

2401 TGAAAACCAGCTTAGCATATATAAAGGGTTTTAAAATTTNTTGAAGGAGA
(+) MYB1AT S000408 2404 WAACCA
(-) TATAPVTRNALEU S000340 2418 TTTATATA
(+) TATABOX4 S000111 2418 TATATAA
(+) TAAAGSTKST1 S000387 2422 TAAAG
(+) DOFCOREZM S000265 2423 AAAG

2451 TGGATGAAAAAATTAAAATAAAAGAAGTGTACCGCAAGAATTGTGTTGCA
(+) GT1CONSENSUS S000198 2456 GRWAAW
(+) GT1GMSCAM4 S000453 2456 GAAAAA
(+) POLASIG2 S000081 2461 AATTAAA
(-) TATABOX5 S000203 2466 TTATTT
(+) POLASIG1 S000080 2467 AATAAA
(+) DOFCOREZM S000265 2471 AAAG
(-) CACTFTPPCA1 S000449 2476 YACT
(-) CURECORECR S000493 2479 GTAC
(+) CURECORECR S000493 2479 GTAC
(-) CAATBOX1 S000028 2490 CAAT
(-) RAV1AAT S000314 2494 CAACA

2501 NGAAGTTGCAATGAACATAGCAAACACGTTCCAAGGAAGATGAATATGTC
(+) CAATBOX1 S000028 2509 CAAT
(+) 2SSEEDPROTBANAPA S000143 2521 CAAACAC
(+) CANBNNAPA S000148 2521 CNAACAC
(-) QARBNEXTA S000244 2524 AACGTGT
(-) ABRERATCAL S000507 2524 MACGYGB
(-) ABRELATERD1 S000414 2525 ACGTG
(-) T/GBOXATPIN2 S000458 2525 AACGTG
(-) ACGTATERD1 S000415 2526 ACGT
(+) ACGTATERD1 S000415 2526 ACGT
(-) ROOTMOTIFTAPOX1 S000098 2543 ATATT
(+) BIHD1OS S000498 2547 TGTCA
(-) WRKY71OS S000447 2548 TGAC

2551 ATGTCTAGTGANCATTTTTCTTAAAGGTGTTTTTCTTTTCTTTTTCTTTT
(-) CACTFTPPCA1 S000449 2557 YACT
(+) GTGANTG10 S000378 2558 GTGA
(-) GT1CONSENSUS S000198 2565 GRWAAW
(-) GT1GMSCAM4 S000453 2565 GAAAAA
(-) POLLEN1LELAT52 S000245 2567 AGAAA
(+) TAAAGSTKST1 S000387 2572 TAAAG
(+) DOFCOREZM S000265 2573 AAAG
(-) GT1CONSENSUS S000198 2580 GRWAAW
(-) GT1GMSCAM4 S000453 2580 GAAAAA
(-) POLLEN1LELAT52 S000245 2582 AGAAA
(-) DOFCOREZM S000265 2585 AAAG
(-) POLLEN1LELAT52 S000245 2587 AGAAA
(-) DOFCOREZM S000265 2590 AAAG
(-) GT1CONSENSUS S000198 2591 GRWAAW
(-) GT1GMSCAM4 S000453 2591 GAAAAA
(-) POLLEN1LELAT52 S000245 2593 AGAAA

```
                                                      (-) DOFCOREZM S000265 2596 AAAG
                                                      (-) CARGCW8GAT S000431 2596 CWWWWWWWWG
                                                      (+) CARGCW8GAT S000431 2596 CWWWWWWWWG

2601    TTTAGCCCTGGAATCACGGATCNGAAGAAGAGACGGATCAACAAGGAGCT
                   (-) ARR1AT S000454 2612 NGATT
                     (-) GTGANTG10 S000378 2614 GTGA
                                   (-) NODCON2GM S000462 2628 CTCTT
                                   (-) OSE2ROOTNODULE S000468 2628 CTCTT
                                     (+) SURECOREATSULTR11 S000499 2630 GAGAC
                                             (+) RAV1AAT S000314 2639 CAACA

2651    GTGAACTGA
        (+) GTGANTG10 S000378 2651 GTGA
```

Factor or Site Name	Loc.(Str.)	Signal Sequence	SITE #
INRNTPSADB	1 (+)	YTCANTYY	S000395
GTGANTG10	2 (-)	GTGA	S000378
CACTFTPPCA1	3 (+)	YACT	S000449
DOFCOREZM	5 (-)	AAAG	S000265
POLLEN1LELAT52	14 (+)	AGAAA	S000245
DOFCOREZM	16 (+)	AAAG	S000265
CACTFTPPCA1	18 (-)	YACT	S000449
CACTFTPPCA1	22 (-)	YACT	S000449
ROOTMOTIFTAPOX1	29 (-)	ATATT	S000098
CACTFTPPCA1	33 (+)	YACT	S000449
TAAAGSTKST1	48 (+)	TAAAG	S000387
DOFCOREZM	49 (+)	AAAG	S000265
ARR1AT	53 (-)	NGATT	S000454
RAV1AAT	56 (+)	CAACA	S000314
SP8BFIBSP8BIB	67 (+)	TACTATT	S000184
CACTFTPPCA1	67 (+)	YACT	S000449
CACTFTPPCA1	77 (-)	YACT	S000449
DOFCOREZM	81 (+)	AAAG	S000265
NODCON2GM	82 (-)	CTCTT	S000462
OSE2ROOTNODULE	82 (-)	CTCTT	S000468
WBOXHVISO1	89 (-)	TGACT	S000442
WBOXNTERF3	89 (-)	TGACY	S000457
WRKY71OS	90 (-)	TGAC	S000447
GTGANTG10	91 (-)	GTGA	S000378
EBOXBNNAPA	94 (-)	CANNTG	S000144
MYCCONSENSUSAT	94 (-)	CANNTG	S000407
EBOXBNNAPA	94 (+)	CANNTG	S000144
MYCCONSENSUSAT	94 (+)	CANNTG	S000407
POLASIG2	105 (-)	AATTAAA	S000081
POLASIG2	108 (+)	AATTAAA	S000081
TAAAGSTKST1	111 (+)	TAAAG	S000387
DOFCOREZM	112 (+)	AAAG	S000265
WBBOXPCWRKY1	133 (+)	TTTGACY	S000310
WBOXATNPR1	134 (+)	TTGAC	S000390
WBOXHVISO1	135 (+)	TGACT	S000442
WRKY71OS	135 (+)	TGAC	S000447
WBOXNTERF3	135 (+)	TGACY	S000457
CACTFTPPCA1	147 (-)	YACT	S000449
CGCGBOXAT	154 (-)	VCGCGB	S000501
CGCGBOXAT	154 (+)	VCGCGB	S000501
CPBCSPOR	165 (+)	TATTAG	S000491
ARR1AT	172 (+)	NGATT	S000454
XYLAT	175 (-)	ACAAAGAA	S000510
DOFCOREZM	177 (-)	AAAG	S000265
ROOTMOTIFTAPOX1	187 (-)	ATATT	S000098
GATABOX	199 (+)	GATA	S000039
S1FBOXSORPS1L21	207 (+)	ATGGTA	S000223
GT1CORE	215 (-)	GGTTAA	S000125
MYB1AT	216 (+)	WAACCA	S000408
REALPHALGLHCB21	217 (+)	AACCAA	S000362
ROOTMOTIFTAPOX1	225 (-)	ATATT	S000098
SORLREP3AT	229 (+)	TGTATATAT	S000488

ROOTMOTIFTAPOX1	251	(+)	ATATT	S000098
CPBCSPOR	252	(+)	TATTAG	S000491
ARR1AT	259	(+)	NGATT	S000454
DOFCOREZM	265	(+)	AAAG	S000265
NODCON2GM	266	(-)	CTCTT	S000462
OSE2ROOTNODULE	266	(-)	CTCTT	S000468
GTGANTG10	289	(+)	GTGA	S000378
ROOTMOTIFTAPOX1	293	(-)	ATATT	S000098
CACTFTPPCA1	321	(+)	YACT	S000449
ROOTMOTIFTAPOX1	328	(-)	ATATT	S000098
RAV1AAT	338	(+)	CAACA	S000314
CAATBOX1	341	(+)	CAAT	S000028
POLASIG1	344	(-)	AATAAA	S000080
TATABOX5	345	(+)	TTATTT	S000203
TATABOX2	347	(-)	TATAAAT	S000109
GATABOX	352	(-)	GATA	S000039
ROOTMOTIFTAPOX1	356	(+)	ATATT	S000098
WBOXATNPR1	360	(+)	TTGAC	S000390
BIHD1OS	361	(-)	TGTCA	S000498
WRKY71OS	361	(+)	TGAC	S000447
CACTFTPPCA1	368	(-)	YACT	S000449
GT1CONSENSUS	374	(-)	GRWAAW	S000198
POLLEN1LELAT52	376	(-)	AGAAA	S000245
GATABOX	381	(+)	GATA	S000039
GT1CONSENSUS	381	(+)	GRWAAW	S000198
IBOXCORE	381	(+)	GATAA	S000199
DOFCOREZM	385	(+)	AAAG	S000265
NODCON2GM	386	(-)	CTCTT	S000462
OSE2ROOTNODULE	386	(-)	CTCTT	S000468
MYBCORE	398	(+)	CNGTTR	S000176
POLASIG3	414	(-)	AATAAT	S000088
CAATBOX1	417	(-)	CAAT	S000028
GT1CONSENSUS	435	(-)	GRWAAW	S000198
CCAATBOX1	439	(+)	CCAAT	S000030
EBOXBNNAPA	440	(-)	CANNTG	S000144
MYCCONSENSUSAT	440	(-)	CANNTG	S000407
CAATBOX1	440	(+)	CAAT	S000028
EBOXBNNAPA	440	(+)	CANNTG	S000144
MYCCONSENSUSAT	440	(+)	CANNTG	S000407
CAATBOX1	442	(-)	CAAT	S000028
POLLEN1LELAT52	446	(+)	AGAAA	S000245
DOFCOREZM	448	(+)	AAAG	S000265
NODCON2GM	449	(-)	CTCTT	S000462
OSE2ROOTNODULE	449	(-)	CTCTT	S000468
EBOXBNNAPA	462	(-)	CANNTG	S000144
MYCCONSENSUSAT	462	(-)	CANNTG	S000407
EBOXBNNAPA	462	(+)	CANNTG	S000144
MYCCONSENSUSAT	462	(+)	CANNTG	S000407
ARR1AT	473	(+)	NGATT	S000454
CACTFTPPCA1	479	(+)	YACT	S000449
CURECORECR	500	(-)	GTAC	S000493
CURECORECR	500	(+)	GTAC	S000493
CACTFTPPCA1	501	(+)	YACT	S000449
EBOXBNNAPA	511	(-)	CANNTG	S000144
MYCCONSENSUSAT	511	(-)	CANNTG	S000407
CAATBOX1	511	(+)	CAAT	S000028
EBOXBNNAPA	511	(+)	CANNTG	S000144
MYCCONSENSUSAT	511	(+)	CANNTG	S000407
CAATBOX1	513	(-)	CAAT	S000028
CACTFTPPCA1	521	(-)	YACT	S000449
AMMORESIVDCRNIA1	534	(+)	CGAACTT	S000375
DOFCOREZM	538	(-)	AAAG	S000265
POLLEN1LELAT52	540	(-)	AGAAA	S000245
GATABOX	561	(+)	GATA	S000039
WBOXHVISO1	564	(-)	TGACT	S000442
WBOXNTERF3	564	(-)	TGACY	S000457
WBOXNTCHN48	564	(-)	CTGACY	S000508
WRKY71OS	565	(-)	TGAC	S000447
CACTFTPPCA1	568	(-)	YACT	S000449
POLLEN1LELAT52	572	(-)	AGAAA	S000245

CPBCSPOR	575	(-)	TATTAG	S000491
CBFHV	581	(-)	RYCGAC	S000497
GATABOX	584	(+)	GATA	S000039
ROOTMOTIFTAPOX1	585	(+)	ATATT	S000098
POLLEN1LELAT52	592	(+)	AGAAA	S000245
EECCRCAH1	593	(-)	GANTTNC	S000494
GT1CONSENSUS	593	(+)	GRWAAW	S000198
ARR1AT	596	(-)	NGATT	S000454
GTGANTG10	598	(-)	GTGA	S000378
EBOXBNNAPA	599	(-)	CANNTG	S000144
MYCCONSENSUSAT	599	(-)	CANNTG	S000407
EBOXBNNAPA	599	(+)	CANNTG	S000144
MYCCONSENSUSAT	599	(+)	CANNTG	S000407
CACTFTPPCA1	599	(+)	YACT	S000449
WBBOXPCWRKY1	623	(+)	TTTGACY	S000310
WBOXATNPR1	624	(+)	TTGAC	S000390
WBOXHVISO1	625	(+)	TGACT	S000442
WRKY71OS	625	(+)	TGAC	S000447
WBOXNTERF3	625	(+)	TGACY	S000457
POLLEN1LELAT52	631	(+)	AGAAA	S000245
GT1CONSENSUS	632	(+)	GRWAAW	S000198
GT1GMSCAM4	632	(+)	GAAAAA	S000453
WBOXHVISO1	639	(+)	TGACT	S000442
WRKY71OS	639	(+)	TGAC	S000447
WBOXNTERF3	639	(+)	TGACY	S000457
NTBBF1ARROLB	641	(+)	ACTTTA	S000273
DOFCOREZM	642	(-)	AAAG	S000265
TAAAGSTKST1	642	(-)	TAAAG	S000387
GATABOX	649	(+)	GATA	S000039
GT1CONSENSUS	649	(+)	GRWAAW	S000198
IBOXCORE	649	(+)	GATAA	S000199
ARR1AT	660	(-)	NGATT	S000454
GT1CONSENSUS	675	(-)	GRWAAW	S000198
GT1GMSCAM4	675	(-)	GAAAAA	S000453
POLLEN1LELAT52	677	(-)	AGAAA	S000245
GT1CONSENSUS	695	(-)	GRWAAW	S000198
BIHD1OS	703	(-)	TGTCA	S000498
WRKY71OS	703	(+)	TGAC	S000447
CAATBOX1	707	(-)	CAAT	S000028
GTGANTG10	710	(+)	GTGA	S000378
ARR1AT	711	(+)	NGATT	S000454
GTGANTG10	717	(+)	GTGA	S000378
ACGTTBOX	725	(-)	AACGTT	S000132
ACGTTBOX	725	(+)	AACGTT	S000132
ACGTATERD1	726	(-)	ACGT	S000415
ACGTATERD1	726	(+)	ACGT	S000415
EECCRCAH1	737	(-)	GANTTNC	S000494
RBCSCONSENSUS	744	(-)	AATCCAA	S000127
ARR1AT	746	(+)	NGATT	S000454
CACTFTPPCA1	754	(-)	YACT	S000449
ANAERO1CONSENSUS	759	(-)	AAACAAA	S000477
AMYBOX1	764	(-)	TAACARA	S000020
MYBGAHV	764	(-)	TAACAAA	S000181
GAREAT	764	(-)	TAACAAR	S000439
RAV1AAT	782	(+)	CAACA	S000314
CAATBOX1	785	(+)	CAAT	S000028
AMYBOX1	788	(+)	TAACARA	S000020
ROOTMOTIFTAPOX1	796	(+)	ATATT	S000098
CACTFTPPCA1	802	(-)	YACT	S000449
S1FBOXSORPS1L21	807	(+)	ATGGTA	S000223
GT1CONSENSUS	809	(+)	GRWAAW	S000198
NTBBF1ARROLB	811	(-)	ACTTTA	S000273
TAAAGSTKST1	811	(+)	TAAAG	S000387
DOFCOREZM	812	(+)	AAAG	S000265
CACTFTPPCA1	814	(-)	YACT	S000449
CURECORECR	817	(-)	GTAC	S000493
CURECORECR	817	(+)	GTAC	S000493
LTRECOREATCOR15	831	(+)	CCGAC	S000153
PRECONSCRHSP70A	831	(+)	SCGAYNRNNNNNNNNNNNNNNNHD	S000506
CACTFTPPCA1	836	(+)	YACT	S000449

EBOXBNNAPA	844	(-)	CANNTG	S000144
MYCCONSENSUSAT	844	(-)	CANNTG	S000407
EBOXBNNAPA	844	(+)	CANNTG	S000144
MYCCONSENSUSAT	844	(+)	CANNTG	S000407
PRECONSCRHSP70A	868	(+)	SCGAYNRNNNNNNNNNNNNNNNHD	S000506
GATABOX	870	(+)	GATA	S000039
CACTFTPPCA1	892	(-)	YACT	S000449
CAATBOX1	900	(-)	CAAT	S000028
WBOXATNPR1	901	(+)	TTGAC	S000390
BIHD1OS	902	(-)	TGTCA	S000498
WRKY71OS	902	(+)	TGAC	S000447
CAATBOX1	905	(+)	CAAT	S000028
BOXIINTPATPB	907	(+)	ATAGAA	S000296
POLLEN1LELAT52	909	(+)	AGAAA	S000245
NODCON2GM	918	(+)	CTCTT	S000462
OSE2ROOTNODULE	918	(+)	CTCTT	S000468
DOFCOREZM	920	(-)	AAAG	S000265
S1FSORPL21	930	(+)	ATGGTATT	S000215
S1FBOXSORPS1L21	930	(+)	ATGGTA	S000223
GT1CONSENSUS	935	(-)	GRWAAW	S000198
POLLEN1LELAT52	937	(-)	AGAAA	S000245
PYRIMIDINEBOXOSRAMY1A	945	(+)	CCTTTT	S000259
DOFCOREZM	946	(-)	AAAG	S000265
POLLEN1LELAT52	948	(-)	AGAAA	S000245
CACTFTPPCA1	957	(+)	YACT	S000449
GT1CONSENSUS	964	(-)	GRWAAW	S000198
IBOXCORE	965	(-)	GATAA	S000199
GATABOX	966	(-)	GATA	S000039
GTGANTG10	968	(-)	GTGA	S000378
CACTFTPPCA1	969	(+)	YACT	S000449
POLASIG1	977	(+)	AATAAA	S000080
PYRIMIDINEBOXOSRAMY1A	981	(-)	CCTTTT	S000259
DOFCOREZM	982	(+)	AAAG	S000265
NODCON2GM	992	(-)	CTCTT	S000462
OSE2ROOTNODULE	992	(-)	CTCTT	S000468
SURECOREATSULTR11	996	(+)	GAGAC	S000499
EECCRCAH1	1003	(+)	GANTTNC	S000494
-300ELEMENT	1008	(-)	TGHAAARK	S000122
PYRIMIDINEBOXOSRAMY1A	1008	(+)	CCTTTT	S000259
DOFCOREZM	1009	(-)	AAAG	S000265
GT1CONSENSUS	1018	(+)	GRWAAW	S000198
MYB1LEPR	1024	(-)	GTTAGTT	S000443
CACTFTPPCA1	1038	(-)	YACT	S000449
NTBBF1ARROLB	1040	(-)	ACTTTA	S000273
TAAAGSTKST1	1040	(+)	TAAAG	S000387
DOFCOREZM	1041	(+)	AAAG	S000265
CACTFTPPCA1	1043	(-)	YACT	S000449
NODCON2GM	1049	(+)	CTCTT	S000462
OSE2ROOTNODULE	1049	(+)	CTCTT	S000468
PREATPRODH	1056	(+)	ACTCAT	S000450
NODCON2GM	1062	(+)	CTCTT	S000462
OSE2ROOTNODULE	1062	(+)	CTCTT	S000468
DOFCOREZM	1064	(-)	AAAG	S000265
POLASIG1	1066	(-)	AATAAA	S000080
MARTBOX	1067	(+)	TTWTWTTWTT	S000067
TATABOX5	1067	(+)	TTATTT	S000203
POLASIG1	1071	(-)	AATAAA	S000080
TATABOX5	1072	(+)	TTATTT	S000203
GT1CONSENSUS	1074	(-)	GRWAAW	S000198
POLLEN1LELAT52	1076	(-)	AGAAA	S000245
DOFCOREZM	1079	(-)	AAAG	S000265
DOFCOREZM	1086	(-)	AAAG	S000265
LTRE1HVBLT49	1087	(-)	CCGAAA	S000250
GATABOX	1099	(-)	GATA	S000039
DOFCOREZM	1108	(-)	AAAG	S000265
GT1CONSENSUS	1109	(-)	GRWAAW	S000198
GT1GMSCAM4	1109	(-)	GAAAAA	S000453
POLLEN1LELAT52	1111	(-)	AGAAA	S000245
WBOXNTCHN48	1114	(+)	CTGACY	S000508
WRKY71OS	1115	(+)	TGAC	S000447

WBOXNTERF3	1115	(+)	TGACY	S000457
CAATBOX1	1120	(-)	CAAT	S000028
WBOXHVISO1	1127	(-)	TGACT	S000442
WBOXNTERF3	1127	(-)	TGACY	S000457
WRKY71OS	1128	(-)	TGAC	S000447
GTGANTG10	1129	(-)	GTGA	S000378
POLLEN1LELAT52	1135	(+)	AGAAA	S000245
DOFCOREZM	1138	(+)	AAAG	S000265
WBOXHVISO1	1140	(-)	TGACT	S000442
WBOXNTERF3	1140	(-)	TGACY	S000457
WBOXNTCHN48	1140	(-)	CTGACY	S000508
WRKY71OS	1141	(-)	TGAC	S000447
CACTFTPPCA1	1144	(-)	YACT	S000449
POLLEN1LELAT52	1153	(+)	AGAAA	S000245
GT1CONSENSUS	1154	(+)	GRWAAW	S000198
BIHD1OS	1160	(-)	TGTCA	S000498
WRKY71OS	1160	(+)	TGAC	S000447
WRKY71OS	1165	(+)	TGAC	S000447
WBOXNTERF3	1165	(+)	TGACY	S000457
ARR1AT	1178	(-)	NGATT	S000454
CACTFTPPCA1	1186	(-)	YACT	S000449
CURECORECR	1187	(-)	GTAC	S000493
CURECORECR	1187	(+)	GTAC	S000493
ROOTMOTIFTAPOX1	1193	(-)	ATATT	S000098
CACTFTPPCA1	1201	(-)	YACT	S000449
ERELEE4	1209	(+)	AWTTCAAA	S000037
ROOTMOTIFTAPOX1	1215	(-)	ATATT	S000098
DOFCOREZM	1221	(-)	AAAG	S000265
MARTBOX	1226	(-)	TTWTWTTWTT	S000067
MARTBOX	1227	(-)	TTWTWTTWTT	S000067
MARTBOX	1228	(-)	TTWTWTTWTT	S000067
MARTBOX	1229	(-)	TTWTWTTWTT	S000067
MARTBOX	1230	(-)	TTWTWTTWTT	S000067
ARR1AT	1238	(-)	NGATT	S000454
NODCON2GM	1242	(-)	CTCTT	S000462
OSE2ROOTNODULE	1242	(-)	CTCTT	S000468
POLLEN1LELAT52	1245	(+)	AGAAA	S000245
GT1CONSENSUS	1246	(+)	GRWAAW	S000198
GT1GMSCAM4	1246	(+)	GAAAAA	S000453
ANAERO1CONSENSUS	1250	(+)	AAACAAA	S000477
BP5OSWX	1259	(+)	CAACGTG	S000436
QARBNEXTA	1260	(+)	AACGTGT	S000244
T/GBOXATPIN2	1260	(+)	AACGTG	S000458
ABRERATCAL	1260	(+)	MACGYGB	S000507
ACGTATERD1	1261	(-)	ACGT	S000415
ABRELATERD1	1261	(+)	ACGTG	S000414
ACGTATERD1	1261	(+)	ACGT	S000415
GTGANTG10	1265	(+)	GTGA	S000378
GATABOX	1267	(+)	GATA	S000039
GATABOX	1269	(-)	GATA	S000039
MYBST1	1269	(-)	GGATA	S000180
TATCCAOSAMY	1269	(+)	TATCCA	S000403
TBOXATGAPB	1273	(-)	ACTTTG	S000383
DOFCOREZM	1274	(+)	AAAG	S000265
CACTFTPPCA1	1276	(-)	YACT	S000449
GT1CONSENSUS	1282	(-)	GRWAAW	S000198
POLLEN1LELAT52	1284	(-)	AGAAA	S000245
ARR1AT	1294	(-)	NGATT	S000454
GTGANTG10	1296	(-)	GTGA	S000378
CACTFTPPCA1	1297	(+)	YACT	S000449
POLASIG1	1314	(-)	AATAAA	S000080
TATABOX5	1315	(+)	TTATTT	S000203
SEBFCONSSTPR10A	1320	(+)	YTGTCWC	S000391
ARFAT	1321	(+)	TGTCTC	S000270
SURECOREATSULTR11	1322	(-)	GAGAC	S000499
NODCON2GM	1324	(+)	CTCTT	S000462
OSE2ROOTNODULE	1324	(+)	CTCTT	S000468
ARR1AT	1331	(-)	NGATT	S000454
TATABOX5	1335	(-)	TTATTT	S000203
MYB2CONSENSUSAT	1338	(+)	YAACKG	S000409

ROOTMOTIFTAPOX1	1358	(+)	ATATT	S000098
TATABOX4	1361	(-)	TATATAA	S000111
TATAPVTRNALEU	1362	(-)	TTTATATA	S000340
TATABOX4	1362	(+)	TATATAA	S000111
TATABOX2	1364	(+)	TATAAAT	S000109
GT1CONSENSUS	1374	(+)	GRWAAW	S000198
GT1CONSENSUS	1375	(+)	GRWAAW	S000198
GT1GMSCAM4	1375	(+)	GAAAAA	S000453
DOFCOREZM	1378	(+)	AAAG	S000265
NODCON1GM	1378	(+)	AAAGAT	S000461
OSE1ROOTNODULE	1378	(+)	AAAGAT	S000467
ARR1AT	1380	(+)	NGATT	S000454
CAATBOX1	1382	(-)	CAAT	S000028
GATABOX	1385	(+)	GATA	S000039
GT1CONSENSUS	1385	(+)	GRWAAW	S000198
IBOXCORE	1385	(+)	GATAA	S000199
DOFCOREZM	1389	(+)	AAAG	S000265
POLLEN1LELAT52	1391	(+)	AGAAA	S000245
GT1CONSENSUS	1392	(+)	GRWAAW	S000198
GT1GMSCAM4	1392	(+)	GAAAAA	S000453
TATABOX5	1395	(-)	TTATTT	S000203
POLASIG3	1396	(+)	AATAAT	S000088
CPBCSPOR	1404	(+)	TATTAG	S000491
CACTFTPPCA1	1408	(-)	YACT	S000449
CURECORECR	1409	(-)	GTAC	S000493
CURECORECR	1409	(+)	GTAC	S000493
CACTFTPPCA1	1414	(+)	YACT	S000449
CPBCSPOR	1416	(-)	TATTAG	S000491
CAATBOX1	1423	(+)	CAAT	S000028
NODCON2GM	1428	(-)	CTCTT	S000462
OSE2ROOTNODULE	1428	(-)	CTCTT	S000468
PYRIMIDINEBOXHVEPB1	1432	(-)	TTTTTTCC	S000298
GT1CONSENSUS	1432	(+)	GRWAAW	S000198
GT1CONSENSUS	1433	(+)	GRWAAW	S000198
GT1GMSCAM4	1433	(+)	GAAAAA	S000453
POLASIG2	1438	(+)	AATTAAA	S000081
TAAAGSTKST1	1441	(+)	TAAAG	S000387
DOFCOREZM	1442	(+)	AAAG	S000265
ROOTMOTIFTAPOX1	1449	(-)	ATATT	S000098
RAV1AAT	1457	(-)	CAACA	S000314
GTGANTG10	1469	(+)	GTGA	S000378
ARR1AT	1475	(+)	NGATT	S000454
BIHD1OS	1481	(+)	TGTCA	S000498
WBOXATNPR1	1482	(-)	TTGAC	S000390
WRKY71OS	1482	(-)	TGAC	S000447
CAATBOX1	1484	(+)	CAAT	S000028
GT1CONSENSUS	1486	(-)	GRWAAW	S000198
IBOXCORE	1487	(-)	GATAA	S000199
SREATMSD	1487	(+)	TTATCC	S000470
GATABOX	1488	(-)	GATA	S000039
MYBST1	1488	(-)	GGATA	S000180
TATCCAOSAMY	1488	(+)	TATCCA	S000403
CACTFTPPCA1	1493	(-)	YACT	S000449
EBOXBNNAPA	1499	(-)	CANNTG	S000144
MYCCONSENSUSAT	1499	(-)	CANNTG	S000407
EBOXBNNAPA	1499	(+)	CANNTG	S000144
MYCCONSENSUSAT	1499	(+)	CANNTG	S000407
CACTFTPPCA1	1507	(+)	YACT	S000449
CACTFTPPCA1	1520	(+)	YACT	S000449
POLASIG1	1528	(-)	AATAAA	S000080
TATABOX5	1529	(+)	TTATTT	S000203
GT1CONSENSUS	1531	(-)	GRWAAW	S000198
POLLEN1LELAT52	1537	(+)	AGAAA	S000245
GT1CONSENSUS	1538	(+)	GRWAAW	S000198
GT1GMSCAM4	1538	(+)	GAAAAA	S000453
DOFCOREZM	1542	(+)	AAAG	S000265
GTGANTG10	1549	(+)	GTGA	S000378
MYB1AT	1553	(-)	WAACCA	S000408
ARR1AT	1561	(+)	NGATT	S000454
CACTFTPPCA1	1566	(-)	YACT	S000449

CURECORECR	1567	(-)	GTAC	S000493
CURECORECR	1567	(+)	GTAC	S000493
CAATBOX1	1570	(+)	CAAT	S000028
POLASIG3	1572	(-)	AATAAT	S000088
TATABOX5	1573	(+)	TTATTT	S000203
CACTFTPPCA1	1591	(-)	YACT	S000449
CACTFTPPCA1	1596	(-)	YACT	S000449
CACTFTPPCA1	1599	(-)	YACT	S000449
CBFHV	1616	(+)	RYCGAC	S000497
CARGNCAT	1621	(-)	CCWWWWWWWWGG	S000446
CCAATBOX1	1621	(+)	CCAAT	S000030
CARGNCAT	1621	(+)	CCWWWWWWWWGG	S000446
CARGCW8GAT	1622	(-)	CWWWWWWWWG	S000431
CAATBOX1	1622	(+)	CAAT	S000028
CARGCW8GAT	1622	(+)	CWWWWWWWWG	S000431
POLASIG1	1623	(+)	AATAAA	S000080
CAATBOX1	1628	(-)	CAAT	S000028
CCAATBOX1	1628	(-)	CCAAT	S000030
NODCON2GM	1633	(-)	CTCTT	S000462
OSE2ROOTNODULE	1633	(-)	CTCTT	S000468
GT1CONSENSUS	1637	(+)	GRWAAW	S000198
TATABOX5	1639	(-)	TTATTT	S000203
POLASIG1	1640	(+)	AATAAA	S000080
GT1CONSENSUS	1648	(+)	GRWAAW	S000198
GT1CONSENSUS	1649	(+)	GRWAAW	S000198
GATABOX	1655	(+)	GATA	S000039
IBOXCORE	1655	(+)	GATAA	S000199
MYB1AT	1657	(+)	WAACCA	S000408
REALPHALGLHCB21	1658	(+)	AACCAA	S000362
POLASIG3	1673	(+)	AATAAT	S000088
AMMORESIVDCRNIA1	1682	(+)	CGAACTT	S000375
GAREAT	1686	(-)	TAACAAR	S000439
CPBCSPOR	1702	(-)	TATTAG	S000491
POLASIG3	1709	(-)	AATAAT	S000088
CAATBOX1	1712	(-)	CAAT	S000028
CCAATBOX1	1712	(-)	CCAAT	S000030
ARR1AT	1717	(-)	NGATT	S000454
ROOTMOTIFTAPOX1	1729	(-)	ATATT	S000098
ROOTMOTIFTAPOX1	1730	(+)	ATATT	S000098
ROOTMOTIFTAPOX1	1735	(+)	ATATT	S000098
GTGANTG10	1739	(-)	GTGA	S000378
POLLEN1LELAT52	1744	(+)	AGAAA	S000245
UP2ATMSD	1746	(+)	AAACCCTA	S000472
GATABOX	1758	(-)	GATA	S000039
MYBST1	1758	(-)	GGATA	S000180
BOXIINTPATPB	1781	(-)	ATAGAA	S000296
SEF1MOTIF	1784	(-)	ATATTTAWW	S000006
TATABOX2	1784	(+)	TATAAAT	S000109
ROOTMOTIFTAPOX1	1788	(-)	ATATT	S000098
ROOTMOTIFTAPOX1	1789	(+)	ATATT	S000098
POLASIG2	1795	(+)	AATTAAA	S000081
SEF1MOTIF	1796	(-)	ATATTTAWW	S000006
TATABOXOSPAL	1797	(-)	TATTTAA	S000400
ROOTMOTIFTAPOX1	1800	(-)	ATATT	S000098
SEF1MOTIF	1801	(+)	ATATTTAWW	S000006
ROOTMOTIFTAPOX1	1801	(+)	ATATT	S000098
TATABOXOSPAL	1802	(+)	TATTTAA	S000400
POLASIG2	1804	(-)	AATTAAA	S000081
TAAAGSTKST1	1811	(+)	TAAAG	S000387
DOFCOREZM	1812	(+)	AAAG	S000265
GTGANTG10	1817	(+)	GTGA	S000378
CACTFTPPCA1	1820	(-)	YACT	S000449
POLASIG3	1823	(-)	AATAAT	S000088
TATABOX5	1824	(+)	TTATTT	S000203
POLLEN1LELAT52	1833	(-)	AGAAA	S000245
CURECORECR	1844	(-)	GTAC	S000493
CURECORECR	1844	(+)	GTAC	S000493
CACTFTPPCA1	1845	(+)	YACT	S000449
RAV1AAT	1849	(-)	CAACA	S000314
TATCCAOSAMY	1852	(-)	TATCCA	S000403

MYBST1	1853	(+)	GGATA	S000180
GATABOX	1854	(+)	GATA	S000039
UP2ATMSD	1856	(-)	AAACCCTA	S000472
MYBCORE	1873	(-)	CNGTTR	S000176
MYB2AT	1873	(+)	TAACTG	S000177
MYB2CONSENSUSAT	1873	(+)	YAACKG	S000409
ARR1AT	1892	(-)	NGATT	S000454
ROOTMOTIFTAPOX1	1896	(+)	ATATT	S000098
CACTFTPPCA1	1901	(+)	YACT	S000449
WUSATAg	1906	(-)	TTAATGG	S000433
GT1CORE	1909	(-)	GGTTAA	S000125
SEF4MOTIFGM7S	1917	(-)	RTTTTTR	S000103
ARR1AT	1921	(-)	NGATT	S000454
CAATBOX1	1924	(+)	CAAT	S000028
ARR1AT	1925	(-)	NGATT	S000454
PYRIMIDINEBOXHVEPB1	1931	(-)	TTTTTTCC	S000298
GT1CONSENSUS	1931	(+)	GRWAAW	S000198
GT1CONSENSUS	1932	(+)	GRWAAW	S000198
GT1GMSCAM4	1932	(+)	GAAAAA	S000453
DOFCOREZM	1936	(+)	AAAG	S000265
NODCON2GM	1937	(-)	CTCTT	S000462
OSE2ROOTNODULE	1937	(-)	CTCTT	S000468
POLLEN1LELAT52	1940	(+)	AGAAA	S000245
CACTFTPPCA1	1947	(-)	YACT	S000449
GTGANTG10	1948	(+)	GTGA	S000378
CACTFTPPCA1	1955	(+)	YACT	S000449
TBOXATGAPB	1956	(+)	ACTTTG	S000383
DOFCOREZM	1957	(-)	AAAG	S000265
ANAERO1CONSENSUS	1958	(-)	AAACAAA	S000477
POLASIG3	1968	(-)	AATAAT	S000088
TATABOX5	1969	(+)	TTATTT	S000203
GT1CONSENSUS	1975	(+)	GRWAAW	S000198
LECPLEACS2	1986	(+)	TAAAATAT	S000465
ROOTMOTIFTAPOX1	1989	(-)	ATATT	S000098
BIHD1OS	1993	(-)	TGTCA	S000498
WRKY71OS	1993	(+)	TGAC	S000447
CAATBOX1	1996	(+)	CAAT	S000028
GT1CONSENSUS	1998	(-)	GRWAAW	S000198
POLLEN1LELAT52	2000	(-)	AGAAA	S000245
CANBNNAPA	2003	(+)	CNAACAC	S000148
CACTFTPPCA1	2007	(+)	YACT	S000449
DOFCOREZM	2009	(-)	AAAG	S000265
POLLEN1LELAT52	2023	(-)	AGAAA	S000245
POLLEN1LELAT52	2028	(+)	AGAAA	S000245
GTGANTG10	2035	(-)	GTGA	S000378
NODCON1GM	2039	(-)	AAAGAT	S000461
OSE1ROOTNODULE	2039	(-)	AAAGAT	S000467
DOFCOREZM	2041	(-)	AAAG	S000265
TAAAGSTKST1	2041	(-)	TAAAG	S000387
MYBCORE	2046	(+)	CNGTTR	S000176
ARR1AT	2050	(+)	NGATT	S000454
GT1CONSENSUS	2053	(-)	GRWAAW	S000198
IBOXCORE	2054	(-)	GATAA	S000199
GATABOX	2055	(-)	GATA	S000039
EBOXBNNAPA	2058	(-)	CANNTG	S000144
MYCCONSENSUSAT	2058	(-)	CANNTG	S000407
EBOXBNNAPA	2058	(+)	CANNTG	S000144
MYCCONSENSUSAT	2058	(+)	CANNTG	S000407
WBOXNTERF3	2063	(-)	TGACY	S000457
WRKY71OS	2064	(-)	TGAC	S000447
GTGANTG10	2065	(-)	GTGA	S000378
CACTFTPPCA1	2066	(+)	YACT	S000449
ROOTMOTIFTAPOX1	2076	(-)	ATATT	S000098
GT1CONSENSUS	2087	(-)	GRWAAW	S000198
IBOXCORE	2088	(-)	GATAA	S000199
GATABOX	2089	(-)	GATA	S000039
INRNTPSADB	2092	(+)	YTCANTYY	S000395
CAATBOX1	2094	(+)	CAAT	S000028
AMYBOX1	2097	(-)	TAACARA	S000020
MYBGAHV	2097	(-)	TAACAAA	S000181

GAREAT	2097	(-)	TAACAAR	S000439
IBOXCORE	2101	(-)	GATAA	S000199
GATABOX	2102	(-)	GATA	S000039
NODCON1GM	2103	(-)	AAAGAT	S000461
OSE1ROOTNODULE	2103	(-)	AAAGAT	S000467
DOFCOREZM	2105	(-)	AAAG	S000265
POLLEN1LELAT52	2106	(-)	AGAAA	S000245
DOFCOREZM	2109	(-)	AAAG	S000265
POLASIG1	2113	(-)	AATAAA	S000080
CAATBOX1	2120	(+)	CAAT	S000028
ARR1AT	2121	(-)	NGATT	S000454
NODCON1GM	2122	(-)	AAAGAT	S000461
OSE1ROOTNODULE	2122	(-)	AAAGAT	S000467
DOFCOREZM	2124	(-)	AAAG	S000265
GT1CONSENSUS	2125	(-)	GRWAAW	S000198
RAV1AAT	2130	(+)	CAACA	S000314
POLLEN1LELAT52	2137	(+)	AGAAA	S000245
CAATBOX1	2143	(+)	CAAT	S000028
GT1CONSENSUS	2145	(-)	GRWAAW	S000198
GT1CONSENSUS	2146	(-)	GRWAAW	S000198
POLLEN1LELAT52	2153	(+)	AGAAA	S000245
GT1CONSENSUS	2154	(+)	GRWAAW	S000198
TATABOX5	2156	(-)	TTATTT	S000203
SP8BFIBSP8BIB	2163	(+)	TACTATT	S000184
CACTFTPPCA1	2163	(+)	YACT	S000449
CARGCW8GAT	2165	(-)	CWWWWWWWWG	S000431
CARGCW8GAT	2165	(+)	CWWWWWWWWG	S000431
TATABOX3	2166	(+)	TATTAAT	S000110
CAATBOX1	2171	(-)	CAAT	S000028
WRKY71OS	2177	(+)	TGAC	S000447
WBOXNTERF3	2177	(+)	TGACY	S000457
EBOXBNNAPA	2181	(-)	CANNTG	S000144
MYCCONSENSUSAT	2181	(-)	CANNTG	S000407
EBOXBNNAPA	2181	(+)	CANNTG	S000144
MYCCONSENSUSAT	2181	(+)	CANNTG	S000407
-300ELEMENT	2185	(+)	TGHAAARK	S000122
GT1CONSENSUS	2186	(+)	GRWAAW	S000198
GT1GMSCAM4	2186	(+)	GAAAAA	S000453
ARR1AT	2190	(-)	NGATT	S000454
NODCON1GM	2203	(-)	AAAGAT	S000461
OSE1ROOTNODULE	2203	(-)	AAAGAT	S000467
DOFCOREZM	2205	(-)	AAAG	S000265
POLLEN1LELAT52	2206	(-)	AGAAA	S000245
GATABOX	2216	(-)	GATA	S000039
CACTFTPPCA1	2230	(+)	YACT	S000449
DOFCOREZM	2236	(+)	AAAG	S000265
POLLEN1LELAT52	2238	(+)	AGAAA	S000245
GT1CONSENSUS	2239	(+)	GRWAAW	S000198
GT1GMSCAM4	2239	(+)	GAAAAA	S000453
DOFCOREZM	2242	(+)	AAAG	S000265
GTGANTG10	2264	(-)	GTGA	S000378
ARR1AT	2269	(+)	NGATT	S000454
EECCRCAH1	2270	(+)	GANTTNC	S000494
DOFCOREZM	2276	(-)	AAAG	S000265
CCA1ATLHCB1	2282	(+)	AAMAATCT	S000149
ARR1AT	2285	(-)	NGATT	S000454
POLLEN1LELAT52	2290	(+)	AGAAA	S000245
GT1CONSENSUS	2291	(+)	GRWAAW	S000198
GT1GMSCAM4	2291	(+)	GAAAAA	S000453
DOFCOREZM	2295	(+)	AAAG	S000265
CACTFTPPCA1	2297	(-)	YACT	S000449
POLASIG2	2307	(+)	AATTAAA	S000081
DOFCOREZM	2314	(-)	AAAG	S000265
INRNTPSADB	2343	(-)	YTCANTYY	S000395
POLLEN1LELAT52	2356	(+)	AGAAA	S000245
GT1CONSENSUS	2357	(+)	GRWAAW	S000198
GT1GMSCAM4	2357	(+)	GAAAAA	S000453
NODCON2GM	2366	(+)	CTCTT	S000462
OSE2ROOTNODULE	2366	(+)	CTCTT	S000468
POLLEN1LELAT52	2373	(-)	AGAAA	S000245

ARR1AT	2384	(+)	NGATT	S000454
RYREPEATLEGUMINBOX	2393	(+)	CATGCAY	S000100
RYREPEATGMGY2	2393	(+)	CATGCAT	S000105
RYREPEATBNNAPA	2393	(+)	CATGCA	S000264
EBOXBNNAPA	2397	(-)	CANNTG	S000144
MYCCONSENSUSAT	2397	(-)	CANNTG	S000407
EBOXBNNAPA	2397	(+)	CANNTG	S000144
MYCCONSENSUSAT	2397	(+)	CANNTG	S000407
MYB1AT	2404	(+)	WAACCA	S000408
TATAPVTRNALEU	2418	(-)	TTTATATA	S000340
TATABOX4	2418	(+)	TATATAA	S000111
TAAAGSTKST1	2422	(+)	TAAAG	S000387
DOFCOREZM	2423	(+)	AAAG	S000265
GT1CONSENSUS	2456	(+)	GRWAAW	S000198
GT1GMSCAM4	2456	(+)	GAAAAA	S000453
POLASIG2	2461	(+)	AATTAAA	S000081
TATABOX5	2466	(-)	TTATTT	S000203
POLASIG1	2467	(+)	AATAAA	S000080
DOFCOREZM	2471	(+)	AAAG	S000265
CACTFTPPCA1	2476	(-)	YACT	S000449
CURECORECR	2479	(-)	GTAC	S000493
CURECORECR	2479	(+)	GTAC	S000493
CAATBOX1	2490	(-)	CAAT	S000028
RAV1AAT	2494	(-)	CAACA	S000314
CAATBOX1	2509	(+)	CAAT	S000028
2SSEEDPROTBANAPA	2521	(+)	CAAACAC	S000143
CANBNNAPA	2521	(+)	CNAACAC	S000148
QARBNEXTA	2524	(-)	AACGTGT	S000244
ABRERATCAL	2524	(-)	MACGYGB	S000507
ABRELATERD1	2525	(-)	ACGTG	S000414
T/GBOXATPIN2	2525	(-)	AACGTG	S000458
ACGTATERD1	2526	(-)	ACGT	S000415
ACGTATERD1	2526	(+)	ACGT	S000415
ROOTMOTIFTAPOX1	2543	(-)	ATATT	S000098
BIHD1OS	2547	(+)	TGTCA	S000498
WRKY71OS	2548	(-)	TGAC	S000447
CACTFTPPCA1	2557	(-)	YACT	S000449
GTGANTG10	2558	(+)	GTGA	S000378
GT1CONSENSUS	2565	(-)	GRWAAW	S000198
GT1GMSCAM4	2565	(-)	GAAAAA	S000453
POLLEN1LELAT52	2567	(-)	AGAAA	S000245
TAAAGSTKST1	2572	(+)	TAAAG	S000387
DOFCOREZM	2573	(+)	AAAG	S000265
GT1CONSENSUS	2580	(-)	GRWAAW	S000198
GT1GMSCAM4	2580	(-)	GAAAAA	S000453
POLLEN1LELAT52	2582	(-)	AGAAA	S000245
DOFCOREZM	2585	(-)	AAAG	S000265
POLLEN1LELAT52	2587	(-)	AGAAA	S000245
DOFCOREZM	2590	(-)	AAAG	S000265
GT1CONSENSUS	2591	(-)	GRWAAW	S000198
GT1GMSCAM4	2591	(-)	GAAAAA	S000453
POLLEN1LELAT52	2593	(-)	AGAAA	S000245
DOFCOREZM	2596	(-)	AAAG	S000265
CARGCW8GAT	2596	(-)	CWWWWWWWWG	S000431
CARGCW8GAT	2596	(+)	CWWWWWWWWG	S000431
ARR1AT	2612	(-)	NGATT	S000454
GTGANTG10	2614	(-)	GTGA	S000378
NODCON2GM	2628	(-)	CTCTT	S000462
OSE2ROOTNODULE	2628	(-)	CTCTT	S000468
SURECOREATSULTR11	2630	(+)	GAGAC	S000499
RAV1AAT	2639	(+)	CAACA	S000314
GTGANTG10	2651	(+)	GTGA	S000378

//

©2011-2019 National Agriculture and Food Research Organization
