# Supplementary material for: Whole genome resequencing of four Italian sweet pepper landraces provides insights on sequence variation in genes of agronomic value
Source: Sci Rep. 2020 Jun 8;10:9189. doi: 10.1038/s41598-020-66053-2 (PMC7280500; doi:10.1038/s41598-020-66053-2)
Supplement: Supplementary file 1 — Supplementary File S1. [file 41598_2020_66053_MOESM1_ESM.zip › File_S1/Manuscript information.pdf]

Whole genome resequencing of four Italian sweet pepper landraces provides insights on sequence variation in genes of agronomic value.

Alberto Acquadro[1], Lorenzo Barchi[1*], Ezio Portis[1], Mohamed Nourdine[1], Cristiano Carli[2], Simone Monge[3], Danila Valentino[1], Sergio Lanteri[1]

Supplementary Figures
