# Supplementary material for: Whole genome resequencing of four Italian sweet pepper landraces provides insights on sequence variation in genes of agronomic value
Source: Sci Rep. 2020 Jun 8;10:9189. doi: 10.1038/s41598-020-66053-2 (PMC7280500; doi:10.1038/s41598-020-66053-2)
Supplement: Supplementary file 1 — Supplementary File S1. [file 41598_2020_66053_MOESM1_ESM.zip › File_S1/perennial_CLV3_PLACE_plus_boxes.pdf]

Fri Jul 26 18:16:35 JST 2019

```
CTCACTTTTGTCTAGAAAGTAAGTAAAAAATATACTTATATGATCCTTAAAGAATCAACA
GATTTTACTATTTGCAGTAAAAGAGCCAGTCACATTTGGCAAATTTAATTAAAGACAAAT
TCTGTATAATTTTGACTAGGAGTTAGGGCCGCGCATTCTTATTAGGGGATTCTTTGTTGG
AATATAACTTACGATACCTGATGGTAGTTTAACCAAAAAATATGTATATATATATATATA
TATATATTAGGGGATTCAAAGAGGATCAAGCTAGAATGTATGTGAAATATAATGAGATTC
AAATTTGCGATCTACTTCAAATATAAACACAACAATTTATTTATATCATATTTGACACAG
TATAATTTTCTGATAAAAGAGATCGATCCTGTTAAACATCCTTCATTATTGTAGATCCGC
CCCCATTTCCAATTGAGAAAGAGGGAACAAGCAAATGGGGGTGGATTTTACTAAATAATT
TAAGCCATGTACTCCATCTCAATTGTGGAAGTATTCCACAAACGAACTTTTCTTGAGTTG
ATCTCTCAGATAGTCAGTATTTCTAATAGTCGATATTTCAGAAAATCACTTGTTTTGTTG
AACAAAAAATTTGACTTAGAAAAAATGACTTTAGAGATAATAACTAAATCAAACCCTAAA
TTTTTCTCTAAGGACCCCAAATTACCTATGACATTGTGATTTGTGATGGAAACGTTGGTG
CGGAATTCTTGGATTCCTAGTATTTTGTTTTGTTAGGGTAACATTACAACAATAACAACA
TATTCAGTGTATGGTAAAGTGTACGCAGTCCATCCCGACCACTAACTCAGATGAAGTAGA
GAGGTTGTTTCCGATAGACCCCTCGGCTCATGTTAGTGTAACATTGACAATAGAAATTCC
TCTTTCCCTTCATGGTATTTTCTCCTCCTTTTCTTCTCTACTAGCATTATCACTAGTAAT
AAAAAGGCTATGAAGAGAGACTAGAATTCCTGTTCATGGGTAATAACTAACATAAGGAGT
AAAGTAGCCTCTTGGACTCATCTCTTTTATTTTATTTTCTTTTGCCTTTCGGTGCTCTAT
CTTCGACTTTTTCTGACCATTGTTCAGTCACACAGAAAAGTCAGTAAACTAAGAAAATGA
CATGACCACAACTAAAATCATGAAGTACATAATATGAGAGTAAGCAAATTCAAATATCTT
TTAAAAAAAAAAAAAATCAAGAGAAAAACAAATTCAACGTGTGATATCCAAAGTAAATTT
TCTGATGTAATCACTCTAGCATCATCATTTTATTTTGTCTCTTCAAATCAAATAACGTAA
CTACATACATTATATTATATAAATGGAGGAAAAAGATTGATAAAAGAAAAATAATTTATT
AGTACACACTAATAACAATGAAGAGGAAAAAATTAAAGCAAAATATATATGTTGTGACTT
GTGAGAGGATTACGTCAATTATCCAGTGGACAAATGCATACTCCTATAACCTACTTATTT
TATTTTCAGAAAAAAGAATGTGATGGTTTCAAGATTAGTACAATTATTTATGATCAGTGC
AGTGCAGTAGTACGAACCTGATTAGGATCGACCAATAAATTGGAAGAGGAAATAAAAGGA
AAATGATCACCAAATTATGAGGAATAATTAACGAACTTGTTAAATGGTGGACTAATAATT
AGTTGAATCCTTAAAAAAATATTATATTCACAAGAAACCCTAGTCCTATCCTATGTATAG
AGATCTAGTTCTATAAATATTAAATTAAATATTTAATTTAAAGCGTGAGTATTATTTAAT
TTCTGCCTAGGTACTTGTTGGATAGGGTTTGAACAAAATTAACTGGATCTGAACTAAAAT
CATATTTACTTCCATTAACCTTCAAAAATCAATCAAGGAAAAAAGAGAAAACAGTAAAAC
ACTTTGTTTTAAATTATTTGAAAATTAGCATAAAATATGACAATTTTCTAACACTTTGAA
GCAAAATTTCTACAAACATCACATCTTTACGATTGATTTATCAAATGGTCACTCAAGTAA
TATATAGTTATTATCTCAATTTGTTATCTTTCTTTTTTATTACAATCTTTTCCAACAAAG
AAAACAATTTTCCGAGAAAATAACTACTATTAGTTGAATGACCATCTGAAAAATCATCTC
GGCATCTTTCTAACCCTATCTTATGGCTTGTACTAAAAGAAAAAGAAGACTTCGGTTTA
ATTTCACAAGATTTGCTTTCAAAAAATCTAGAAAAAAGTGGAACAAAATTAAACTTTGAA
CTTGTATTCATCATCATAGACAAAATGAGAGCTCAGAAAAATGGCTCTTAATTTCTTCAA
ACGATTCGATCATGCATTTGAAAACCAGCTTAGCATATATAAAGGGTTTTAAAATTTTTG
AAGGAGATGGATGAAAAAATTAAAATAAAAGAAGTGTACCGCAAGAATTGTGTTGCAGAA
GTTGCAATGAACATAGCAAACACGTTCCAAGGAAGATGAATATGTCATGTCTAGTGACAT
TTTTCTTAAAGGTGTTTTTCTTTTCTTTTTCTTTTTTTAGCCCTGGAATCACGGATCGAA
GAAGAGACGGATCAACAAGGAGCTGTGAACTGA
```

RESULTS OF YOUR SIGNAL SCAN SEARCH REQUEST

This result is the output of the new signal scan program which was completely rewritten from a scratch by Akio Miyao ($Id: 649.pl,v 1.11 2016/04/20 08:43:39 miyao Exp $).

The original program of signal scan was reported in
Prestridge, D.S. (1991) SIGNAL SCAN: A computer program that scans DNA sequences for eukaryotic transcriptional elements. CABIOS 7, 203-206.

```
2656 base pairs

 (+) = Current Strand
 (-) = Opposite Strand

1      CTCACTTTTGTCTAGAAAGTAAGTAAAAAATATACTTATATGATCCTTAA
       (+) INRNTPSADB S000395 1 YTCANTYY
        (-) GTGANTG10 S000378 2 GTGA
         (+) CACTFTPPCA1 S000449 3 YACT
           (-) DOFCOREZM S000265 5 AAAG
                    (+) POLLEN1LELAT52 S000245 14 AGAAA
                      (+) DOFCOREZM S000265 16 AAAG
                        (-) CACTFTPPCA1 S000449 18 YACT
                            (-) CACTFTPPCA1 S000449 22 YACT
                                   (-) ROOTMOTIFTAPOX1 S000098 29 ATATT
                                       (+) CACTFTPPCA1 S000449 33 YACT
                                                      (+) TAAAGSTKST1 S000387 48 TAAAG
                                                       (+) DOFCOREZM S000265 49 AAAG

51     AGAATCAACANGATTTTACTATTTGCAGTAAAAGAGCCAGTCACATTTGG
         (-) ARR1AT S000454 53 NGATT
            (+) RAV1AAT S000314 56 CAACA
                       (+) SP8BFIBSP8BIB S000184 67 TACTATT
                       (+) CACTFTPPCA1 S000449 67 YACT
                                 (-) CACTFTPPCA1 S000449 77 YACT
                                     (+) DOFCOREZM S000265 81 AAAG
                                      (-) NODCON2GM S000462 82 CTCTT
                                      (-) OSE2ROOTNODULE S000468 82 CTCTT
                                             (-) WBOXHVISO1 S000442 89 TGACT
                                             (-) WBOXNTERF3 S000457 89 TGACY
                                              (-) WRKY71OS S000447 90 TGAC
                                               (-) GTGANTG10 S000378 91 GTGA
                                                  (-) EBOXBNNAPA S000144 94 CANNTG
                                                  (-) MYCCONSENSUSAT S000407 94 CANNTG
                                                  (+) EBOXBNNAPA S000144 94 CANNTG
                                                  (+) MYCCONSENSUSAT S000407 94 CANNTG

101    CAAATTTAATTAAAGACAAATNTCTGTATAATTTTGACTAGGAGTTAGGG
           (-) POLASIG2 S000081 105 AATTAAA
              (+) POLASIG2 S000081 108 AATTAAA
                 (+) TAAAGSTKST1 S000387 111 TAAAG
                  (+) DOFCOREZM S000265 112 AAAG
                                       (+) WBBOXPCWRKY1 S000310 133 TTTGACY
                                        (+) WBOXATNPR1 S000390 134 TTGAC
                                         (+) WBOXHVISO1 S000442 135 TGACT
                                         (+) WRKY71OS S000447 135 TGAC
                                         (+) WBOXNTERF3 S000457 135 TGACY
                                                      (+) SORLIP2AT S000483 148 GGGCC

151    CCGCGCATTCTTATTAGGGGATTCTTTGTTGGNAATATAACTTACGATAC
       (-) CGCGBOXAT S000501 151 VCGCGB
       (+) CGCGBOXAT S000501 151 VCGCGB
                  (+) CPBCSPOR S000491 162 TATTAG
                         (+) ARR1AT S000454 169 NGATT
                            (-) XYLAT S000510 172 ACAAAGAA
                              (-) DOFCOREZM S000265 174 AAAG
                                 (-) RAV1AAT S000314 177 CAACA
                                        (-) ROOTMOTIFTAPOX1 S000098 184 ATATT
                                                    (+) GATABOX S000039 196 GATA

201    CTGATGGTAGTTTAACCAAAAAATATGTATATATATATATATANTATATA
          (+) S1FBOXSORPS1L21 S000223 204 ATGGTA
                  (-) GT1CORE S000125 212 GGTTAA
                   (+) MYB1AT S000408 213 WAACCA
                    (+) REALPHALGLHCB21 S000362 214 AACCAA
                            (-) ROOTMOTIFTAPOX1 S000098 222 ATATT
                                (+) SORLREP3AT S000488 226 TGTATATAT
                                                      (+) ROOTMOTIFTAPOX1 S000098 248 ATATT
```

(+) CPBCSPOR S000491 249 TATTAG

251 TTAGGGGATTCAAAGAGGATCAAGCTAGAATGTATGTGAAATATAATGAG
(+) ARR1AT S000454 256 NGATT
(+) DOFCOREZM S000265 262 AAAG
(-) NODCON2GM S000462 263 CTCTT
(-) OSE2ROOTNODULE S000468 263 CTCTT
(+) GTGANTG10 S000378 286 GTGA
(-) ROOTMOTIFTAPOX1 S000098 290 ATATT
(+) ARR1AT S000454 299 NGATT

301 ATTCNAAATTTGCGATCTACTTCAAATATAAACACAACAATTTATTTATA
(+) CACTFTPPCA1 S000449 318 YACT
(-) ROOTMOTIFTAPOX1 S000098 325 ATATT
(+) RAV1AAT S000314 335 CAACA
(+) CAATBOX1 S000028 338 CAAT
(-) POLASIG1 S000080 341 AATAAA
(+) TATABOX5 S000203 342 TTATTT
(-) TATABOX2 S000109 344 TATAAAT
(-) GATABOX S000039 349 GATA

351 TCATATTTGACACAGNTATAATTTTCTGATAAAAGAGATCGATCCTGTTA
(+) ROOTMOTIFTAPOX1 S000098 353 ATATT
(+) WBOXATNPR1 S000390 357 TTGAC
(-) BIHD1OS S000498 358 TGTCA
(+) WRKY71OS S000447 358 TGAC
(-) GT1CONSENSUS S000198 371 GRWAAW
(-) POLLEN1LELAT52 S000245 373 AGAAA
(+) GATABOX S000039 378 GATA
(+) GT1CONSENSUS S000198 378 GRWAAW
(+) IBOXCORE S000199 378 GATAA
(+) DOFCOREZM S000265 382 AAAG
(-) NODCON2GM S000462 383 CTCTT
(-) OSE2ROOTNODULE S000468 383 CTCTT
(+) **MYB**CORE S000176 395 CNGTTR

401 AACATCCTTCATTATTGTAGATCCGCNCCCCATTTCCAATTGAGAAAGAG
(-) POLASIG3 S000088 411 AATAAT
(-) CAATBOX1 S000028 414 CAAT
(-) GT1CONSENSUS S000198 432 GRWAAW
(+) CCAATBOX1 S000030 436 CCAAT
(-) EBOXBNNAPA S000144 437 CANNTG
(-) MYCCONSENSUSAT S000407 437 CANNTG
(+) CAATBOX1 S000028 437 CAAT
(+) EBOXBNNAPA S000144 437 CANNTG
(+) MYCCONSENSUSAT S000407 437 CANNTG
(-) CAATBOX1 S000028 439 CAAT
(+) POLLEN1LELAT52 S000245 443 AGAAA
(+) DOFCOREZM S000265 445 AAAG
(-) NODCON2GM S000462 446 CTCTT
(-) OSE2ROOTNODULE S000468 446 CTCTT

451 GGAACAAGCAAATGGGGGTGGATTTTACTAAATAATTNTAAGCCATGTAC
(-) EBOXBNNAPA S000144 459 CANNTG
(-) MYCCONSENSUSAT S000407 459 CANNTG
(+) EBOXBNNAPA S000144 459 CANNTG
(+) MYCCONSENSUSAT S000407 459 CANNTG
(+) ARR1AT S000454 470 NGATT
(+) CACTFTPPCA1 S000449 476 YACT
(-) TATABOX5 S000203 480 TTATTT
(+) POLASIG3 S000088 481 AATAAT
(-) CURECORECR S000493 497 GTAC
(+) CURECORECR S000493 497 GTAC
(+) CACTFTPPCA1 S000449 498 YACT

501 TCCATCTCAATTGTGGAAGTATTCCACAAACGAACTTTTCTTGAGTTGNA
(-) EBOXBNNAPA S000144 508 CANNTG
(-) MYCCONSENSUSAT S000407 508 CANNTG

(+) CAATBOX1 S000028 508 CAAT
(+) EBOXBNNAPA S000144 508 CANNTG
(+) MYCCONSENSUSAT S000407 508 CANNTG
(-) CAATBOX1 S000028 510 CAAT
(-) CACTFTPPCA1 S000449 518 YACT
(+) AMMORESIVDCRNIA1 S000375 531 CGAACTT
(-) DOFCOREZM S000265 535 AAAG
(-) POLLEN1LELAT52 S000245 537 AGAAA
(-) CAREOSREP1 S000421 543 CAACTC

551 TCTCTCAGATAGTCAGTATTTCTAATAGTCGATATTTCAGAAAATCACTT
(+) GATABOX S000039 558 GATA
(-) WBOXHVISO1 S000442 561 TGACT
(-) WBOXNTERF3 S000457 561 TGACY
(-) WBOXNTCHN48 S000508 561 CTGACY
(-) WRKY71OS S000447 562 TGAC
(-) CACTFTPPCA1 S000449 565 YACT
(-) POLLEN1LELAT52 S000245 569 AGAAA
(-) CPBCSPOR S000491 572 TATTAG
(-) CBFHV S000497 578 RYCGAC
(+) GATABOX S000039 581 GATA
(+) ROOTMOTIFTAPOX1 S000098 582 ATATT
(+) POLLEN1LELAT52 S000245 589 AGAAA
(-) EECCRCAH1 S000494 590 GANTTNC
(+) GT1CONSENSUS S000198 590 GRWAAW
(-) ARR1AT S000454 593 NGATT
(-) GTGANTG10 S000378 595 GTGA
(-) EBOXBNNAPA S000144 596 CANNTG
(-) MYCCONSENSUSAT S000407 596 CANNTG
(+) EBOXBNNAPA S000144 596 CANNTG
(+) MYCCONSENSUSAT S000407 596 CANNTG
(+) CACTFTPPCA1 S000449 596 YACT

601 GTTTTGTTGNAACAAAAAATTTGACTTAGAAAAAATGACTTTAGAGATAA
(-) RAV1AAT S000314 605 CAACA
(+) WBBOXPCWRKY1 S000310 620 TTTGACY
(+) WBOXATNPR1 S000390 621 TTGAC
(+) WBOXHVISO1 S000442 622 TGACT
(+) WRKY71OS S000447 622 TGAC
(+) WBOXNTERF3 S000457 622 TGACY
(+) POLLEN1LELAT52 S000245 628 AGAAA
(+) GT1CONSENSUS S000198 629 GRWAAW
(+) GT1GMSCAM4 S000453 629 GAAAAA
(+) WBOXHVISO1 S000442 636 TGACT
(+) WRKY71OS S000447 636 TGAC
(+) WBOXNTERF3 S000457 636 TGACY
(+) NTBBF1ARROLB S000273 638 ACTTTA
(-) DOFCOREZM S000265 639 AAAG
(-) TAAAGSTKST1 S000387 639 TAAAG
(+) GATABOX S000039 646 GATA
(+) GT1CONSENSUS S000198 646 GRWAAW
(+) IBOXCORE S000199 646 GATAA

651 TAACTAAATCAAACCCTAAANTTTTTCTCTAAGGACCCCAAATTACCTAT
(-) ARR1AT S000454 657 NGATT
(+) TELOBOXATEEF1AA1 S000308 661 AAACCCTAA
(+) UP2ATMSD S000472 661 AAACCCTA
(-) GT1CONSENSUS S000198 672 GRWAAW
(-) GT1GMSCAM4 S000453 672 GAAAAA
(-) POLLEN1LELAT52 S000245 674 AGAAA
(-) GT1CONSENSUS S000198 692 GRWAAW
(-) BIHD1OS S000498 700 TGTCA
(+) WRKY71OS S000447 700 TGAC

701 GACATTGTGATTTGTGATGGAAACGTTGGTGNCGGAATTCTTGGATTCCT
(-) CAATBOX1 S000028 704 CAAT
(+) GTGANTG10 S000378 707 GTGA
(+) ARR1AT S000454 708 NGATT

```
                  (+) GTGANTG10 S000378 714 GTGA
                          (-) ACGTTBOX S000132 722 AACGTT
                          (+) ACGTTBOX S000132 722 AACGTT
                           (-) ACGTATERD1 S000415 723 ACGT
                           (+) ACGTATERD1 S000415 723 ACGT
                                      (-) EECCRCAH1 S000494 734 GANTTNC
                                             (-) RBCSCONSENSUS S000127 741 AATCCAA
                                               (+) ARR1AT S000454 743 NGATT

751    AGTATTTTGTTTTGTTAGGGTAACATTACAACAATAACAACANTATTCAG
       (-) CACTFTPPCA1 S000449 751 YACT
            (-) ANAERO1CONSENSUS S000477 756 AAACAAA
                 (-) AMYBOX1 S000020 761 TAACARA
                 (-) MYBGAHV S000181 761 TAACAAA
                 (-) GAREAT S000439 761 TAACAAR
                                   (+) RAV1AAT S000314 779 CAACA
                                      (+) CAATBOX1 S000028 782 CAAT
                                            (+) RAV1AAT S000314 788 CAACA
                                                       (-) CACTFTPPCA1 S000449 799 YACT

801    TGTATGGTAAAGTGTACGCAGTCCATCCCGACCACTAACTCAGATGAAGT
          (+) S1FBOXSORPS1L21 S000223 804 ATGGTA
            (+) GT1CONSENSUS S000198 806 GRWAAW
              (-) NTBBF1ARROLB S000273 808 ACTTTA
              (+) TAAAGSTKST1 S000387 808 TAAAG
               (+) DOFCOREZM S000265 809 AAAG
                 (-) CACTFTPPCA1 S000449 811 YACT
                    (-) CURECORECR S000493 814 GTAC
                    (+) CURECORECR S000493 814 GTAC
                                  (+) LTRECOREATCOR15 S000153 828 CCGAC
                                  (+) PRECONSCRHSP70A S000506 828 SCGAYNRNNNNNNNNNNNNNNNHD
                                       (+) CACTFTPPCA1 S000449 833 YACT
                                               (-) EBOXBNNAPA S000144 841 CANNTG
                                               (-) MYCCONSENSUSAT S000407 841 CANNTG
                                               (+) EBOXBNNAPA S000144 841 CANNTG
                                               (+) MYCCONSENSUSAT S000407 841 CANNTG
                                                      (-) CACTFTPPCA1 S000449 848 YACT

851    AGANGAGGTTGTTTCCGATAGACCCCTCGGCTCATGTTAGTGTAACATTG
                     (+) PRECONSCRHSP70A S000506 865 SCGAYNRNNNNNNNNNNNNNNNHD
                       (+) GATABOX S000039 867 GATA
                                             (-) CACTFTPPCA1 S000449 889 YACT
                                                     (-) CAATBOX1 S000028 897 CAAT
                                                      (+) WBOXATNPR1 S000390 898 TTGAC
                                                       (-) BIHD1OS S000498 899 TGTCA
                                                       (+) WRKY71OS S000447 899 TGAC

901    ACAATAGAAATTCCNTCTTTCCCTTCATGGTATTTTCTCCTCCTTTTCTT
        (+) CAATBOX1 S000028 902 CAAT
          (+) BOXIINTPATPB S000296 904 ATAGAA
            (+) POLLEN1LELAT52 S000245 906 AGAAA
             (-) EECCRCAH1 S000494 907 GANTTNC
                       (-) DOFCOREZM S000265 917 AAAG
                                 (+) S1FSORPL21 S000215 927 ATGGTATT
                                 (+) S1FBOXSORPS1L21 S000223 927 ATGGTA
                                      (-) GT1CONSENSUS S000198 932 GRWAAW
                                        (-) POLLEN1LELAT52 S000245 934 AGAAA
                                                (+) PYRIMIDINEBOXOSRAMY1A S000259 942 CCTTTT
                                                 (-) DOFCOREZM S000265 943 AAAG
                                                   (-) POLLEN1LELAT52 S000245 945 AGAAA

951    CTCTACTAGCATTATCACTAGTAATNAAAAAGGCTATGAAGAGAGACTAG
          (+) CACTFTPPCA1 S000449 954 YACT
                 (-) GT1CONSENSUS S000198 961 GRWAAW
                  (-) IBOXCORE S000199 962 GATAA
                   (-) GATABOX S000039 963 GATA
                     (-) GTGANTG10 S000378 965 GTGA
                      (+) CACTFTPPCA1 S000449 966 YACT
```

(-) CACTFTPPCA1 S000449 970 YACT
(-) PYRIMIDINEBOXOSRAMY1A S000259 978 CCTTTT
(+) DOFCOREZM S000265 979 AAAG
(-) NODCON2GM S000462 989 CTCTT
(-) OSE2ROOTNODULE S000468 989 CTCTT
(+) SURECOREATSULTR11 S000499 993 GAGAC
(+) EECCRCAH1 S000494 1000 GANTTNC

1001 AATTCCTGTTCATGGGTAATAACTAACATAAGGAGTNAAAGTAGCCTCTT
(+) GT1CONSENSUS S000198 1015 GRWAAW
(-) **MYB**1LEPR S000443 1021 GTTAGTT
(+) DOFCOREZM S000265 1038 AAAG
(-) CACTFTPPCA1 S000449 1040 YACT
(+) NODCON2GM S000462 1046 CTCTT
(+) OSE2ROOTNODULE S000468 1046 CTCTT

1051 GGACTCATCTCTTTTATTTTATTTTCTTTTGCCTTTCGGTGCTCTATNCT
(+) PREATPRODH S000450 1053 ACTCAT
(+) NODCON2GM S000462 1059 CTCTT
(+) OSE2ROOTNODULE S000468 1059 CTCTT
(-) DOFCOREZM S000265 1061 AAAG
(-) POLASIG1 S000080 1063 AATAAA
(+) MARTBOX S000067 1064 TTWTWTTWTT
(+) TATABOX5 S000203 1064 TTATTT
(-) POLASIG1 S000080 1068 AATAAA
(+) TATABOX5 S000203 1069 TTATTT
(-) GT1CONSENSUS S000198 1071 GRWAAW
(-) POLLEN1LELAT52 S000245 1073 AGAAA
(-) DOFCOREZM S000265 1076 AAAG
(-) DOFCOREZM S000265 1083 AAAG
(-) LTRE1HVBLT49 S000250 1084 CCGAAA

1101 TCGACTTTTTCTGACCATTGTTCAGTCACACAGAAAAGTCAGTAAACTAA
(-) DOFCOREZM S000265 1105 AAAG
(-) GT1CONSENSUS S000198 1106 GRWAAW
(-) GT1GMSCAM4 S000453 1106 GAAAAA
(-) POLLEN1LELAT52 S000245 1108 AGAAA
(+) WBOXNTCHN48 S000508 1111 CTGACY
(+) WRKY71OS S000447 1112 TGAC
(+) WBOXNTERF3 S000457 1112 TGACY
(-) CAATBOX1 S000028 1117 CAAT
(-) WBOXHVISO1 S000442 1124 TGACT
(-) WBOXNTERF3 S000457 1124 TGACY
(-) WRKY71OS S000447 1125 TGAC
(-) GTGANTG10 S000378 1126 GTGA
(+) POLLEN1LELAT52 S000245 1132 AGAAA
(+) DOFCOREZM S000265 1135 AAAG
(-) WBOXHVISO1 S000442 1137 TGACT
(-) WBOXNTERF3 S000457 1137 TGACY
(-) WBOXNTCHN48 S000508 1137 CTGACY
(-) WRKY71OS S000447 1138 TGAC
(-) CACTFTPPCA1 S000449 1141 YACT
(+) POLLEN1LELAT52 S000245 1150 AGAAA

1151 GAAAATGANCATGACCACAACTAAAATCATGAAGTACATAATATGAGAGT
(+) GT1CONSENSUS S000198 1151 GRWAAW
(+) WRKY71OS S000447 1162 TGAC
(+) WBOXNTERF3 S000457 1162 TGACY
(-) ARR1AT S000454 1175 NGATT
(-) CACTFTPPCA1 S000449 1183 YACT
(-) CURECORECR S000493 1184 GTAC
(+) CURECORECR S000493 1184 GTAC
(-) ROOTMOTIFTAPOX1 S000098 1190 ATATT
(-) CACTFTPPCA1 S000449 1198 YACT

1201 AAGCAAATTCAAATATCTTNTTAAAAAAAAAAAAAATCAAGAGAAAAACA
(+) ERELEE4 S000037 1206 AWTTCAAA
(-) ROOTMOTIFTAPOX1 S000098 1212 ATATT

```
                   (-) GATABOX S000039 1214 GATA
                            (-) MARTBOX S000067 1223 TTWTWTTWTT
                             (-) MARTBOX S000067 1224 TTWTWTTWTT
                              (-) MARTBOX S000067 1225 TTWTWTTWTT
                               (-) MARTBOX S000067 1226 TTWTWTTWTT
                                (-) MARTBOX S000067 1227 TTWTWTTWTT
                                        (-) ARR1AT S000454 1235 NGATT
                                            (-) NODCON2GM S000462 1239 CTCTT
                                            (-) OSE2ROOTNODULE S000468 1239 CTCTT
                                               (+) POLLEN1LELAT52 S000245 1242 AGAAA
                                                (+) GT1CONSENSUS S000198 1243 GRWAAW
                                                (+) GT1GMSCAM4 S000453 1243 GAAAAA
                                                   (+) ANAERO1CONSENSUS S000477 1246 AAACAAA

1251   AATTCAACGTGTGATATCCAAAGTAAATTTNTCTGATGTAATCACTCTAG
           (+) BP5OSWX S000436 1255 CAACGTG
            (+) QARBNEXTA S000244 1256 AACGTGT
            (+) T/GBOXATPIN2 S000458 1256 AACGTG
            (+) ABRERATCAL S000507 1256 MACGYGB
             (-) ACGTATERD1 S000415 1257 ACGT
             (+) ABRELATERD1 S000414 1257 ACGTG
             (+) ACGTATERD1 S000415 1257 ACGT
                 (+) GTGANTG10 S000378 1261 GTGA
                   (+) GATABOX S000039 1263 GATA
                     (-) GATABOX S000039 1265 GATA
                     (-) MYBST1 S000180 1265 GGATA
                     (+) TATCCAOSAMY S000403 1265 TATCCA
                         (-) TBOXATGAPB S000383 1269 ACTTTG
                          (+) DOFCOREZM S000265 1270 AAAG
                            (-) CACTFTPPCA1 S000449 1272 YACT
                                              (-) ARR1AT S000454 1290 NGATT
                                                (-) GTGANTG10 S000378 1292 GTGA
                                                 (+) CACTFTPPCA1 S000449 1293 YACT

1301   CATCATCATTTTATTTTGTCTCTTCAAATCAAATAACGTAANCTACATAC
                (-) POLASIG1 S000080 1310 AATAAA
                 (+) TATABOX5 S000203 1311 TTATTT
                      (+) SEBFCONSSTPR10A S000391 1316 YTGTCWC
                       (+) ARFAT S000270 1317 TGTCTC
                        (-) SURECOREATSULTR11 S000499 1318 GAGAC
                          (+) NODCON2GM S000462 1320 CTCTT
                          (+) OSE2ROOTNODULE S000468 1320 CTCTT
                                 (-) ARR1AT S000454 1327 NGATT
                                     (-) TATABOX5 S000203 1331 TTATTT
                                        (+) GARE2OSREP1 S000420 1334 TAACGTA
                                          (-) ACGTATERD1 S000415 1336 ACGT
                                          (+) ACGTATERD1 S000415 1336 ACGT

1351   ATTATATTATATAAATGGAGGAAAAAGATTGATAAAAGAAAAATAATTTA
          (+) ROOTMOTIFTAPOX1 S000098 1354 ATATT
             (-) TATABOX4 S000111 1357 TATATAA
              (-) TATAPVTRNALEU S000340 1358 TTTATATA
              (+) TATABOX4 S000111 1358 TATATAA
                (+) TATABOX2 S000109 1360 TATAAAT
                          (+) GT1CONSENSUS S000198 1370 GRWAAW
                           (+) GT1CONSENSUS S000198 1371 GRWAAW
                           (+) GT1GMSCAM4 S000453 1371 GAAAAA
                              (+) DOFCOREZM S000265 1374 AAAG
                              (+) NODCON1GM S000461 1374 AAAGAT
                              (+) OSE1ROOTNODULE S000467 1374 AAAGAT
                                (+) ARR1AT S000454 1376 NGATT
                                  (-) CAATBOX1 S000028 1378 CAAT
                                     (+) GATABOX S000039 1381 GATA
                                     (+) GT1CONSENSUS S000198 1381 GRWAAW
                                     (+) IBOXCORE S000199 1381 GATAA
                                         (+) DOFCOREZM S000265 1385 AAAG
                                           (+) POLLEN1LELAT52 S000245 1387 AGAAA
                                            (+) GT1CONSENSUS S000198 1388 GRWAAW
```

(+) GT1GMSCAM4 S000453 1388 GAAAAA
(-) TATABOX5 S000203 1391 TTATTT
(+) POLASIG3 S000088 1392 AATAAT
(-) POLASIG1 S000080 1397 AATAAA

1401 TTNAGTACACACTAATAACAATGAAGAGGAAAAAATTAAAGCAAAATATA
(-) CACTFTPPCA1 S000449 1404 YACT
(-) CURECORECR S000493 1405 GTAC
(+) CURECORECR S000493 1405 GTAC
(+) CACTFTPPCA1 S000449 1410 YACT
(-) CPBCSPOR S000491 1412 TATTAG
(+) CAATBOX1 S000028 1419 CAAT
(-) NODCON2GM S000462 1424 CTCTT
(-) OSE2ROOTNODULE S000468 1424 CTCTT
(-) PYRIMIDINEBOXHVEPB1 S000298 1428 TTTTTTCC
(+) GT1CONSENSUS S000198 1428 GRWAAW
(+) GT1CONSENSUS S000198 1429 GRWAAW
(+) GT1GMSCAM4 S000453 1429 GAAAAA
(+) POLASIG2 S000081 1434 AATTAAA
(+) TAAAGSTKST1 S000387 1437 TAAAG
(+) DOFCOREZM S000265 1438 AAAG
(-) ROOTMOTIFTAPOX1 S000098 1445 ATATT

1451 TATGTTGTGACTTNGTGAGAGGATTACGTCAATTATCCAGTGGACAAATG
(-) RAV1AAT S000314 1453 CAACA
(+) GTGANTG10 S000378 1457 GTGA
(+) WBOXHVISO1 S000442 1458 TGACT
(+) WRKY71OS S000447 1458 TGAC
(+) WBOXNTERF3 S000457 1458 TGACY
(+) GTGANTG10 S000378 1465 GTGA
(+) ARR1AT S000454 1471 NGATT
(-) AUXRETGA1GMGH3 S000234 1474 TGACGTAA
(-) TGACGTVMAMY S000377 1476 TGACGT
(-) ACGTATERD1 S000415 1476 ACGT
(+) HEXMOTIFTAH3H4 S000053 1476 ACGTCA
(+) ACGTATERD1 S000415 1476 ACGT
(-) ASF1MOTIFCAMV S000024 1477 TGACG
(-) WBOXATNPR1 S000390 1478 TTGAC
(-) WRKY71OS S000447 1478 TGAC
(+) CAATBOX1 S000028 1480 CAAT
(-) GT1CONSENSUS S000198 1482 GRWAAW
(-) IBOXCORE S000199 1483 GATAA
(+) SREATMSD S000470 1483 TTATCC
(-) GATABOX S000039 1484 GATA
(-) **MYB**ST1 S000180 1484 GGATA
(+) TATCCAOSAMY S000403 1484 TATCCA
(-) CACTFTPPCA1 S000449 1489 YACT
(-) EBOXBNNAPA S000144 1495 CANNTG
(-) MYCCONSENSUSAT S000407 1495 CANNTG
(+) EBOXBNNAPA S000144 1495 CANNTG
(+) MYCCONSENSUSAT S000407 1495 CANNTG

1501 CATACTCCTATAACCTACTTATTTNTATTTTCAGAAAAAAGAATGTGATG
(+) CACTFTPPCA1 S000449 1503 YACT
(+) CACTFTPPCA1 S000449 1516 YACT
(+) TATABOX5 S000203 1519 TTATTT
(-) GT1CONSENSUS S000198 1527 GRWAAW
(+) POLLEN1LELAT52 S000245 1533 AGAAA
(+) GT1CONSENSUS S000198 1534 GRWAAW
(+) GT1GMSCAM4 S000453 1534 GAAAAA
(+) DOFCOREZM S000265 1538 AAAG
(+) GTGANTG10 S000378 1545 GTGA
(-) **MYB**1AT S000408 1549 WAACCA

1551 GTTTCAAGATTAGTACAATTATTTATGATCAGTGCNAGTGCAGTAGTACG
(+) ARR1AT S000454 1557 NGATT
(-) CACTFTPPCA1 S000449 1562 YACT
(-) CURECORECR S000493 1563 GTAC

```
                   (+) CURECORECR S000493 1563 GTAC
                      (+) CAATBOX1 S000028 1566 CAAT
                        (-) POLASIG3 S000088 1568 AATAAT
                         (+) TATABOX5 S000203 1569 TTATTT
                                     (-) CACTFTPPCA1 S000449 1581 YACT
                                           (-) CACTFTPPCA1 S000449 1587 YACT
                                                (-) CACTFTPPCA1 S000449 1592 YACT
                                                   (-) CACTFTPPCA1 S000449 1595 YACT
                                                    (-) CURECORECR S000493 1596 GTAC
                                                    (+) CURECORECR S000493 1596 GTAC

1601   AACCTGATTAGGATCGACCAATAAATTGGAAGAGGAAATAAAAGGANAAA
           (+) ARR1AT S000454 1605 NGATT
                   (+) CBFHV S000497 1613 RYCGAC
                        (-) CARGNCAT S000446 1618 CCWWWWWWWWGG
                        (+) CCAATBOX1 S000030 1618 CCAAT
                        (+) CARGNCAT S000446 1618 CCWWWWWWWWGG
                         (-) CARGCW8GAT S000431 1619 CWWWWWWWWG
                         (+) CAATBOX1 S000028 1619 CAAT
                         (+) CARGCW8GAT S000431 1619 CWWWWWWWWG
                          (+) POLASIG1 S000080 1620 AATAAA
                               (-) CAATBOX1 S000028 1625 CAAT
                               (-) CCAATBOX1 S000030 1625 CCAAT
                                    (-) NODCON2GM S000462 1630 CTCTT
                                    (-) OSE2ROOTNODULE S000468 1630 CTCTT
                                        (+) GT1CONSENSUS S000198 1634 GRWAAW
                                          (-) TATABOX5 S000203 1636 TTATTT
                                           (+) POLASIG1 S000080 1637 AATAAA
                                              (-) PYRIMIDINEBOXOSRAMY1A S000259 1640 CCTTTT
                                               (+) DOFCOREZM S000265 1641 AAAG

1651   TGATCACCAAATTATGAGGAATAATTAACGAACTTGTTAAATGGTGGACT
          (-) GTGANTG10 S000378 1654 GTGA
                          (+) POLASIG3 S000088 1670 AATAAT
                                   (+) AMMORESIVDCRNIA1 S000375 1679 CGAACTT
                                       (-) GAREAT S000439 1683 TAACAAR
                                                       (-) CPBCSPOR S000491 1699 TATTAG

1701   AATAATTNAGTTGAATCCTTAAAAAAATATTATATTCACAAGAAACCCTA
       (+) POLASIG3 S000088 1701 AATAAT
                    (-) ARR1AT S000454 1714 NGATT
                                (-) ROOTMOTIFTAPOX1 S000098 1726 ATATT
                                 (+) ROOTMOTIFTAPOX1 S000098 1727 ATATT
                                      (+) ROOTMOTIFTAPOX1 S000098 1732 ATATT
                                          (-) GTGANTG10 S000378 1736 GTGA
                                               (+) POLLEN1LELAT52 S000245 1741 AGAAA
                                                 (+) UP2ATMSD S000472 1743 AAACCCTA

1751   GTCCTATCCTATGTATAGNAGATCTAGTTCTATAAATATTAAATTAAATA
           (-) GATABOX S000039 1755 GATA
           (-) MYBST1 S000180 1755 GGATA
                                  (-) BOXIINTPATPB S000296 1778 ATAGAA
                                     (-) SEF1MOTIF S000006 1781 ATATTTAWW
                                     (+) TATABOX2 S000109 1781 TATAAAT
                                         (-) ROOTMOTIFTAPOX1 S000098 1785 ATATT
                                          (+) ROOTMOTIFTAPOX1 S000098 1786 ATATT
                                                (+) POLASIG2 S000081 1792 AATTAAA
                                                 (-) SEF1MOTIF S000006 1793 ATATTTAWW
                                                  (-) TATABOXOSPAL S000400 1794 TATTTAA
                                                     (-) ROOTMOTIFTAPOX1 S000098 1797 ATATT
                                                      (+) SEF1MOTIF S000006 1798 ATATTTAWW
                                                      (+) ROOTMOTIFTAPOX1 S000098 1798 ATATT
                                                       (+) TATABOXOSPAL S000400 1799 TATTTAA

1801   TTTAATTTAAAGCGTGAGTATTATTTAATNTTCTGCCTAGGTACTTGTTG
       (-) POLASIG2 S000081 1801 AATTAAA
              (+) TAAAGSTKST1 S000387 1808 TAAAG
               (+) DOFCOREZM S000265 1809 AAAG
```

(+) GTGANTG10 S000378 1814 GTGA
(-) CACTFTPPCA1 S000449 1817 YACT
(-) POLASIG3 S000088 1820 AATAAT
(+) TATABOX5 S000203 1821 TTATTT
(+) TATABOXOSPAL S000400 1822 TATTTAA
(-) CURECORECR S000493 1841 GTAC
(+) CURECORECR S000493 1841 GTAC
(+) CACTFTPPCA1 S000449 1842 YACT
(-) RAV1AAT S000314 1846 CAACA
(-) TATCCAOSAMY S000403 1849 TATCCA
(+) **MYB**ST1 S000180 1850 GGATA

1851 GATAGGGTTTGAACAAAATTAACTGGATCTGAACTAAAATNCATATTTAC
(+) GATABOX S000039 1851 GATA
(-) UP2ATMSD S000472 1853 AAACCCTA
(-) **MYB**CORE S000176 1870 CNGTTR
(+) **MYB**2AT S000177 1870 TAACTG
(+) **MYB**2CONSENSUSAT S000409 1870 YAACKG
(+) ROOTMOTIFTAPOX1 S000098 1893 ATATT
(+) CACTFTPPCA1 S000449 1898 YACT

1901 TTCCATTAACCTTCAAAAATCAATCAAGGAAAAAAGAGAAAACAGTAAAA
(-) WUSATAg S000433 1903 TTAATGG
(-) GT1CORE S000125 1906 GGTTAA
(-) SEF4MOTIFGM7S S000103 1914 RTTTTTR
(-) ARR1AT S000454 1918 NGATT
(+) CAATBOX1 S000028 1921 CAAT
(-) ARR1AT S000454 1922 NGATT
(-) PYRIMIDINEBOXHVEPB1 S000298 1928 TTTTTTCC
(+) GT1CONSENSUS S000198 1928 GRWAAW
(+) GT1CONSENSUS S000198 1929 GRWAAW
(+) GT1GMSCAM4 S000453 1929 GAAAAA
(+) DOFCOREZM S000265 1933 AAAG
(-) NODCON2GM S000462 1934 CTCTT
(-) OSE2ROOTNODULE S000468 1934 CTCTT
(+) POLLEN1LELAT52 S000245 1937 AGAAA
(-) CACTFTPPCA1 S000449 1944 YACT

1951 CNACTTTGTTTTAAATTATTTGAAAATTAGCATAAAATATGACAATTTTC
(+) TBOXATGAPB S000383 1953 ACTTTG
(-) DOFCOREZM S000265 1954 AAAG
(-) ANAERO1CONSENSUS S000477 1955 AAACAAA
(-) POLASIG3 S000088 1965 AATAAT
(+) TATABOX5 S000203 1966 TTATTT
(+) GT1CONSENSUS S000198 1972 GRWAAW
(+) LECPLEACS2 S000465 1983 TAAAATAT
(-) ROOTMOTIFTAPOX1 S000098 1986 ATATT
(-) BIHD1OS S000498 1990 TGTCA
(+) WRKY71OS S000447 1990 TGAC
(+) CAATBOX1 S000028 1993 CAAT
(-) GT1CONSENSUS S000198 1995 GRWAAW
(-) POLLEN1LELAT52 S000245 1997 AGAAA
(+) CANBNNAPA S000148 2000 CNAACAC

2001 TAACACTTTGAANGCAAAATTTCTACAAACATCACATCTTTACGATTGAT
(+) CACTFTPPCA1 S000449 2004 YACT
(+) TBOXATGAPB S000383 2005 ACTTTG
(-) DOFCOREZM S000265 2006 AAAG
(-) POLLEN1LELAT52 S000245 2020 AGAAA
(-) GTGANTG10 S000378 2032 GTGA
(-) NODCON1GM S000461 2036 AAAGAT
(-) OSE1ROOTNODULE S000467 2036 AAAGAT
(-) DOFCOREZM S000265 2038 AAAG
(-) TAAAGSTKST1 S000387 2038 TAAAG
(+) ARR1AT S000454 2043 NGATT
(-) CAATBOX1 S000028 2045 CAAT
(+) ARR1AT S000454 2047 NGATT
(-) GT1CONSENSUS S000198 2050 GRWAAW

2051 TTATCAAATGGTCACTCAAGTAANTATATAGTTATTATCTCAATTTGTTA
(-) IBOXCORE S000199 2051 GATAA
(-) GATABOX S000039 2052 GATA
(-) EBOXBNNAPA S000144 2055 CANNTG
(-) MYCCONSENSUSAT S000407 2055 CANNTG
(+) EBOXBNNAPA S000144 2055 CANNTG
(+) MYCCONSENSUSAT S000407 2055 CANNTG
(-) WBOXNTERF3 S000457 2060 TGACY
(-) WRKY71OS S000447 2061 TGAC
(-) GTGANTG10 S000378 2062 GTGA
(+) CACTFTPPCA1 S000449 2063 YACT
(-) CACTFTPPCA1 S000449 2069 YACT
(-) GT1CONSENSUS S000198 2084 GRWAAW
(-) IBOXCORE S000199 2085 GATAA
(-) GATABOX S000039 2086 GATA
(+) INRNTPSADB S000395 2089 YTCANTYY
(+) CAATBOX1 S000028 2091 CAAT
(-) A**MYB**OX1 S000020 2094 TAACARA
(-) **MYB**GAHV S000181 2094 TAACAAA
(-) GAREAT S000439 2094 TAACAAR
(-) IBOXCORE S000199 2098 GATAA
(-) GATABOX S000039 2099 GATA
(-) NODCON1GM S000461 2100 AAAGAT
(-) OSE1ROOTNODULE S000467 2100 AAAGAT

2101 TCTTTCTTTTTTATTACAATCTTTTCCAACAAAGNAAAACAATTTTCCGA
(-) DOFCOREZM S000265 2102 AAAG
(-) POLLEN1LELAT52 S000245 2103 AGAAA
(-) DOFCOREZM S000265 2106 AAAG
(-) POLASIG1 S000080 2110 AATAAA
(+) CAATBOX1 S000028 2117 CAAT
(-) ARR1AT S000454 2118 NGATT
(-) NODCON1GM S000461 2119 AAAGAT
(-) OSE1ROOTNODULE S000467 2119 AAAGAT
(-) DOFCOREZM S000265 2121 AAAG
(-) GT1CONSENSUS S000198 2122 GRWAAW
(+) RAV1AAT S000314 2127 CAACA
(+) DOFCOREZM S000265 2131 AAAG
(+) CAATBOX1 S000028 2140 CAAT
(-) GT1CONSENSUS S000198 2142 GRWAAW
(-) GT1CONSENSUS S000198 2143 GRWAAW
(+) POLLEN1LELAT52 S000245 2150 AGAAA

2151 GAAAATAACTACTATTAGTTGAATGACCATCTGAAAAATCATCTCNGGCA
(+) GT1CONSENSUS S000198 2151 GRWAAW
(-) TATABOX5 S000203 2153 TTATTT
(+) SP8BFIBSP8BIB S000184 2160 TACTATT
(+) CACTFTPPCA1 S000449 2160 YACT
(+) CPBCSPOR S000491 2163 TATTAG
(+) WRKY71OS S000447 2174 TGAC
(+) WBOXNTERF3 S000457 2174 TGACY
(-) EBOXBNNAPA S000144 2178 CANNTG
(-) MYCCONSENSUSAT S000407 2178 CANNTG
(+) EBOXBNNAPA S000144 2178 CANNTG
(+) MYCCONSENSUSAT S000407 2178 CANNTG
(+) -300ELEMENT S000122 2182 TGHAAARK
(+) GT1CONSENSUS S000198 2183 GRWAAW
(+) GT1GMSCAM4 S000453 2183 GAAAAA
(-) ARR1AT S000454 2187 NGATT
(-) NODCON1GM S000461 2200 AAAGAT
(-) OSE1ROOTNODULE S000467 2200 AAAGAT

2201 TCTTTCTAACCCTATCTTATGGCTTGTACTAAAAAGAAAAAGAAGACTTC
(-) DOFCOREZM S000265 2202 AAAG
(-) POLLEN1LELAT52 S000245 2203 AGAAA
(-) GATABOX S000039 2213 GATA
(-) CURECORECR S000493 2226 GTAC

(+) CURECORECR S000493 2226 GTAC
(+) CACTFTPPCA1 S000449 2227 YACT
(+) DOFCOREZM S000265 2233 AAAG
(+) POLLEN1LELAT52 S000245 2235 AGAAA
(+) GT1CONSENSUS S000198 2236 GRWAAW
(+) GT1GMSCAM4 S000453 2236 GAAAAA
(+) DOFCOREZM S000265 2239 AAAG

2251 GGTTTANATTTCACAAGATTTGCTTTCAAAAAATCTAGAAAAAAGTGGAA
(-) GTGANTG10 S000378 2261 GTGA
(+) ARR1AT S000454 2266 NGATT
(+) EECCRCAH1 S000494 2267 GANTTNC
(-) DOFCOREZM S000265 2273 AAAG
(+) CCA1ATLHCB1 S000149 2279 AAMAATCT
(-) ARR1AT S000454 2282 NGATT
(+) POLLEN1LELAT52 S000245 2287 AGAAA
(+) GT1CONSENSUS S000198 2288 GRWAAW
(+) GT1GMSCAM4 S000453 2288 GAAAAA
(+) DOFCOREZM S000265 2292 AAAG
(-) CACTFTPPCA1 S000449 2294 YACT

2301 CAAAATTAAACTTTGAANCTTGTATTCATCATCATAGACAAAATGAGAGC
(+) POLASIG2 S000081 2304 AATTAAA
(+) TBOXATGAPB S000383 2310 ACTTTG
(-) DOFCOREZM S000265 2311 AAAG
(-) INRNTPSADB S000395 2340 YTCANTYY

2351 TCAGAAAAATGGCTCTTAATTTCTTCAANACGATTCGATCATGCATTTGA
(+) POLLEN1LELAT52 S000245 2353 AGAAA
(+) GT1CONSENSUS S000198 2354 GRWAAW
(+) GT1GMSCAM4 S000453 2354 GAAAAA
(+) NODCON2GM S000462 2363 CTCTT
(+) OSE2ROOTNODULE S000468 2363 CTCTT
(-) POLLEN1LELAT52 S000245 2370 AGAAA
(+) ARR1AT S000454 2381 NGATT
(+) RYREPEATLEGUMINBOX S000100 2390 CATGCAY
(+) RYREPEATGMGY2 S000105 2390 CATGCAT
(+) RYREPEATBNNAPA S000264 2390 CATGCA
(-) EBOXBNNAPA S000144 2394 CANNTG
(-) MYCCONSENSUSAT S000407 2394 CANNTG
(+) EBOXBNNAPA S000144 2394 CANNTG
(+) MYCCONSENSUSAT S000407 2394 CANNTG

2401 AAACCAGCTTAGCATATATAAAGGGTTTTAAAATTTTTGNAAGGAGATGG
(+) MYB1AT S000408 2401 WAACCA
(-) TATAPVTRNALEU S000340 2415 TTTATATA
(+) TATABOX4 S000111 2415 TATATAA
(+) TAAAGSTKST1 S000387 2419 TAAAG
(+) DOFCOREZM S000265 2420 AAAG
(+) SEF4MOTIFGM7S S000103 2433 RTTTTTR

2451 ATGAAAAAATTAAAATAAAAGAAGTGTACCGCAAGAATTGTGTTGCAGAA
(+) GT1CONSENSUS S000198 2453 GRWAAW
(+) GT1GMSCAM4 S000453 2453 GAAAAA
(+) POLASIG2 S000081 2458 AATTAAA
(-) TATABOX5 S000203 2463 TTATTT
(+) POLASIG1 S000080 2464 AATAAA
(+) DOFCOREZM S000265 2468 AAAG
(-) CACTFTPPCA1 S000449 2473 YACT
(-) CURECORECR S000493 2476 GTAC
(+) CURECORECR S000493 2476 GTAC
(-) CAATBOX1 S000028 2487 CAAT
(-) RAV1AAT S000314 2491 CAACA

2501 NGTTGCAATGAACATAGCAAACACGTTCCAAGGAAGATGAATATGTCATG
(+) CAATBOX1 S000028 2506 CAAT
(+) 2SSEEDPROTBANAPA S000143 2518 CAAACAC
(+) CANBNNAPA S000148 2518 CNAACAC

```
                                   (-) QARBNEXTA S000244 2521 AACGTGT
                                   (-) ABRERATCAL S000507 2521 MACGYGB
                                    (-) ABRELATERD1 S000414 2522 ACGTG
                                    (-) T/GBOXATPIN2 S000458 2522 AACGTG
                                     (-) ACGTATERD1 S000415 2523 ACGT
                                     (+) ACGTATERD1 S000415 2523 ACGT
                                                      (-) ROOTMOTIFTAPOX1 S000098 2540 ATATT
                                                          (+) BIHD1OS S000498 2544 TGTCA
                                                           (-) WRKY71OS S000447 2545 TGAC

2551   TCTAGTGACATNTTTTCTTAAAGGTGTTTTTCTTTTCTTTTTCTTTTTTT
          (-) CACTFTPPCA1 S000449 2554 YACT
           (+) GTGANTG10 S000378 2555 GTGA
            (-) BIHD1OS S000498 2556 TGTCA
            (+) WRKY71OS S000447 2556 TGAC
                  (-) GT1CONSENSUS S000198 2562 GRWAAW
                    (-) POLLEN1LELAT52 S000245 2564 AGAAA
                         (+) TAAAGSTKST1 S000387 2569 TAAAG
                          (+) DOFCOREZM S000265 2570 AAAG
                                 (-) GT1CONSENSUS S000198 2577 GRWAAW
                                 (-) GT1GMSCAM4 S000453 2577 GAAAAA
                                   (-) POLLEN1LELAT52 S000245 2579 AGAAA
                                      (-) DOFCOREZM S000265 2582 AAAG
                                        (-) POLLEN1LELAT52 S000245 2584 AGAAA
                                           (-) DOFCOREZM S000265 2587 AAAG
                                            (-) GT1CONSENSUS S000198 2588 GRWAAW
                                            (-) GT1GMSCAM4 S000453 2588 GAAAAA
                                              (-) POLLEN1LELAT52 S000245 2590 AGAAA
                                                 (-) DOFCOREZM S000265 2593 AAAG
                                                 (-) CARGCW8GAT S000431 2593 CWWWWWWWWG
                                                 (+) CARGCW8GAT S000431 2593 CWWWWWWWWG

2601   AGCCCTGGAATCACGGATCGAANGAAGAGACGGATCAACAAGGAGCTGTG
               (-) ARR1AT S000454 2609 NGATT
                 (-) GTGANTG10 S000378 2611 GTGA
                               (-) NODCON2GM S000462 2625 CTCTT
                               (-) OSE2ROOTNODULE S000468 2625 CTCTT
                                 (+) SURECOREATSULTR11 S000499 2627 GAGAC
                                          (+) RAV1AAT S000314 2636 CAACA
                                                      (+) GTGANTG10 S000378 2648 GTGA

2651   AACTGA
```

Factor or Site Name	Loc.(Str.)	Signal Sequence	SITE #
INRNTPSADB	1 (+)	YTCANTYY	S000395
GTGANTG10	2 (-)	GTGA	S000378
CACTFTPPCA1	3 (+)	YACT	S000449
DOFCOREZM	5 (-)	AAAG	S000265
POLLEN1LELAT52	14 (+)	AGAAA	S000245
DOFCOREZM	16 (+)	AAAG	S000265
CACTFTPPCA1	18 (-)	YACT	S000449
CACTFTPPCA1	22 (-)	YACT	S000449
ROOTMOTIFTAPOX1	29 (-)	ATATT	S000098
CACTFTPPCA1	33 (+)	YACT	S000449
TAAAGSTKST1	48 (+)	TAAAG	S000387
DOFCOREZM	49 (+)	AAAG	S000265
ARR1AT	53 (-)	NGATT	S000454
RAV1AAT	56 (+)	CAACA	S000314
SP8BFIBSP8BIB	67 (+)	TACTATT	S000184
CACTFTPPCA1	67 (+)	YACT	S000449
CACTFTPPCA1	77 (-)	YACT	S000449
DOFCOREZM	81 (+)	AAAG	S000265
NODCON2GM	82 (-)	CTCTT	S000462
OSE2ROOTNODULE	82 (-)	CTCTT	S000468
WBOXHVISO1	89 (-)	TGACT	S000442
WBOXNTERF3	89 (-)	TGACY	S000457

WRKY71OS	90	(-)	TGAC	S000447
GTGANTG10	91	(-)	GTGA	S000378
EBOXBNNAPA	94	(-)	CANNTG	S000144
MYCCONSENSUSAT	94	(-)	CANNTG	S000407
EBOXBNNAPA	94	(+)	CANNTG	S000144
MYCCONSENSUSAT	94	(+)	CANNTG	S000407
POLASIG2	105	(-)	AATTAAA	S000081
POLASIG2	108	(+)	AATTAAA	S000081
TAAAGSTKST1	111	(+)	TAAAG	S000387
DOFCOREZM	112	(+)	AAAG	S000265
WBBOXPCWRKY1	133	(+)	TTTGACY	S000310
WBOXATNPR1	134	(+)	TTGAC	S000390
WBOXHVISO1	135	(+)	TGACT	S000442
WRKY71OS	135	(+)	TGAC	S000447
WBOXNTERF3	135	(+)	TGACY	S000457
SORLIP2AT	148	(+)	GGGCC	S000483
CGCGBOXAT	151	(-)	VCGCGB	S000501
CGCGBOXAT	151	(+)	VCGCGB	S000501
CPBCSPOR	162	(+)	TATTAG	S000491
ARR1AT	169	(+)	NGATT	S000454
XYLAT	172	(-)	ACAAAGAA	S000510
DOFCOREZM	174	(-)	AAAG	S000265
RAV1AAT	177	(-)	CAACA	S000314
ROOTMOTIFTAPOX1	184	(-)	ATATT	S000098
GATABOX	196	(+)	GATA	S000039
S1FBOXSORPS1L21	204	(+)	ATGGTA	S000223
GT1CORE	212	(-)	GGTTAA	S000125
MYB1AT	213	(+)	WAACCA	S000408
REALPHALGLHCB21	214	(+)	AACCAA	S000362
ROOTMOTIFTAPOX1	222	(-)	ATATT	S000098
SORLREP3AT	226	(+)	TGTATATAT	S000488
ROOTMOTIFTAPOX1	248	(+)	ATATT	S000098
CPBCSPOR	249	(+)	TATTAG	S000491
ARR1AT	256	(+)	NGATT	S000454
DOFCOREZM	262	(+)	AAAG	S000265
NODCON2GM	263	(-)	CTCTT	S000462
OSE2ROOTNODULE	263	(-)	CTCTT	S000468
GTGANTG10	286	(+)	GTGA	S000378
ROOTMOTIFTAPOX1	290	(-)	ATATT	S000098
ARR1AT	299	(+)	NGATT	S000454
CACTFTPPCA1	318	(+)	YACT	S000449
ROOTMOTIFTAPOX1	325	(-)	ATATT	S000098
RAV1AAT	335	(+)	CAACA	S000314
CAATBOX1	338	(+)	CAAT	S000028
POLASIG1	341	(-)	AATAAA	S000080
TATABOX5	342	(+)	TTATTT	S000203
TATABOX2	344	(-)	TATAAAT	S000109
GATABOX	349	(-)	GATA	S000039
ROOTMOTIFTAPOX1	353	(+)	ATATT	S000098
WBOXATNPR1	357	(+)	TTGAC	S000390
BIHD1OS	358	(-)	TGTCA	S000498
WRKY71OS	358	(+)	TGAC	S000447
GT1CONSENSUS	371	(-)	GRWAAW	S000198
POLLEN1LELAT52	373	(-)	AGAAA	S000245
GATABOX	378	(+)	GATA	S000039
GT1CONSENSUS	378	(+)	GRWAAW	S000198
IBOXCORE	378	(+)	GATAA	S000199
DOFCOREZM	382	(+)	AAAG	S000265
NODCON2GM	383	(-)	CTCTT	S000462
OSE2ROOTNODULE	383	(-)	CTCTT	S000468
MYBCORE	395	(+)	CNGTTR	S000176
POLASIG3	411	(-)	AATAAT	S000088
CAATBOX1	414	(-)	CAAT	S000028
GT1CONSENSUS	432	(-)	GRWAAW	S000198
CCAATBOX1	436	(+)	CCAAT	S000030
EBOXBNNAPA	437	(-)	CANNTG	S000144
MYCCONSENSUSAT	437	(-)	CANNTG	S000407
CAATBOX1	437	(+)	CAAT	S000028

EBOXBNNAPA	437	(+)	CANNTG	S000144
MYCCONSENSUSAT	437	(+)	CANNTG	S000407
CAATBOX1	439	(-)	CAAT	S000028
POLLEN1LELAT52	443	(+)	AGAAA	S000245
DOFCOREZM	445	(+)	AAAG	S000265
NODCON2GM	446	(-)	CTCTT	S000462
OSE2ROOTNODULE	446	(-)	CTCTT	S000468
EBOXBNNAPA	459	(-)	CANNTG	S000144
MYCCONSENSUSAT	459	(-)	CANNTG	S000407
EBOXBNNAPA	459	(+)	CANNTG	S000144
MYCCONSENSUSAT	459	(+)	CANNTG	S000407
ARR1AT	470	(+)	NGATT	S000454
CACTFTPPCA1	476	(+)	YACT	S000449
TATABOX5	480	(-)	TTATTT	S000203
POLASIG3	481	(+)	AATAAT	S000088
CURECORECR	497	(-)	GTAC	S000493
CURECORECR	497	(+)	GTAC	S000493
CACTFTPPCA1	498	(+)	YACT	S000449
EBOXBNNAPA	508	(-)	CANNTG	S000144
MYCCONSENSUSAT	508	(-)	CANNTG	S000407
CAATBOX1	508	(+)	CAAT	S000028
EBOXBNNAPA	508	(+)	CANNTG	S000144
MYCCONSENSUSAT	508	(+)	CANNTG	S000407
CAATBOX1	510	(-)	CAAT	S000028
CACTFTPPCA1	518	(-)	YACT	S000449
AMMORESIVDCRNIA1	531	(+)	CGAACTT	S000375
DOFCOREZM	535	(-)	AAAG	S000265
POLLEN1LELAT52	537	(-)	AGAAA	S000245
CAREOSREP1	543	(-)	CAACTC	S000421
GATABOX	558	(+)	GATA	S000039
WBOXHVISO1	561	(-)	TGACT	S000442
WBOXNTERF3	561	(-)	TGACY	S000457
WBOXNTCHN48	561	(-)	CTGACY	S000508
WRKY71OS	562	(-)	TGAC	S000447
CACTFTPPCA1	565	(-)	YACT	S000449
POLLEN1LELAT52	569	(-)	AGAAA	S000245
CPBCSPOR	572	(-)	TATTAG	S000491
CBFHV	578	(-)	RYCGAC	S000497
GATABOX	581	(+)	GATA	S000039
ROOTMOTIFTAPOX1	582	(+)	ATATT	S000098
POLLEN1LELAT52	589	(+)	AGAAA	S000245
EECCRCAH1	590	(-)	GANTTNC	S000494
GT1CONSENSUS	590	(+)	GRWAAW	S000198
ARR1AT	593	(-)	NGATT	S000454
GTGANTG10	595	(-)	GTGA	S000378
EBOXBNNAPA	596	(-)	CANNTG	S000144
MYCCONSENSUSAT	596	(-)	CANNTG	S000407
EBOXBNNAPA	596	(+)	CANNTG	S000144
MYCCONSENSUSAT	596	(+)	CANNTG	S000407
CACTFTPPCA1	596	(+)	YACT	S000449
RAV1AAT	605	(-)	CAACA	S000314
WBBOXPCWRKY1	620	(+)	TTTGACY	S000310
WBOXATNPR1	621	(+)	TTGAC	S000390
WBOXHVISO1	622	(+)	TGACT	S000442
WRKY71OS	622	(+)	TGAC	S000447
WBOXNTERF3	622	(+)	TGACY	S000457
POLLEN1LELAT52	628	(+)	AGAAA	S000245
GT1CONSENSUS	629	(+)	GRWAAW	S000198
GT1GMSCAM4	629	(+)	GAAAAA	S000453
WBOXHVISO1	636	(+)	TGACT	S000442
WRKY71OS	636	(+)	TGAC	S000447
WBOXNTERF3	636	(+)	TGACY	S000457
NTBBF1ARROLB	638	(+)	ACTTTA	S000273
DOFCOREZM	639	(-)	AAAG	S000265
TAAAGSTKST1	639	(-)	TAAAG	S000387
GATABOX	646	(+)	GATA	S000039
GT1CONSENSUS	646	(+)	GRWAAW	S000198
IBOXCORE	646	(+)	GATAA	S000199

ARR1AT		657	(-)	NGATT	S000454
TELOBOXATEEF1AA1		661	(+)	AAACCCTAA	S000308
UP2ATMSD		661	(+)	AAACCCTA	S000472
GT1CONSENSUS		672	(-)	GRWAAW	S000198
GT1GMSCAM4		672	(-)	GAAAAA	S000453
POLLEN1LELAT52		674	(-)	AGAAA	S000245
GT1CONSENSUS		692	(-)	GRWAAW	S000198
BIHD1OS		700	(-)	TGTCA	S000498
WRKY71OS		700	(+)	TGAC	S000447
CAATBOX1		704	(-)	CAAT	S000028
GTGANTG10		707	(+)	GTGA	S000378
ARR1AT		708	(+)	NGATT	S000454
GTGANTG10		714	(+)	GTGA	S000378
ACGTTBOX		722	(-)	AACGTT	S000132
ACGTTBOX		722	(+)	AACGTT	S000132
ACGTATERD1		723	(-)	ACGT	S000415
ACGTATERD1		723	(+)	ACGT	S000415
EECCRCAH1		734	(-)	GANTTNC	S000494
RBCSCONSENSUS		741	(-)	AATCCAA	S000127
ARR1AT		743	(+)	NGATT	S000454
CACTFTPPCA1		751	(-)	YACT	S000449
ANAERO1CONSENSUS		756	(-)	AAACAAA	S000477
AMYBOX1		761	(-)	TAACARA	S000020
MYBGAHV		761	(-)	TAACAAA	S000181
GAREAT		761	(-)	TAACAAR	S000439
RAV1AAT		779	(+)	CAACA	S000314
CAATBOX1	← missing	782	(+)	CAAT	S000028
RAV1AAT	MYBox	788	(+)	CAACA	S000314
CACTFTPPCA1		799	(-)	YACT	S000449
S1FBOXSORPS1L21		804	(+)	ATGGTA	S000223
GT1CONSENSUS		806	(+)	GRWAAW	S000198
NTBBF1ARROLB		808	(-)	ACTTTA	S000273
TAAAGSTKST1		808	(+)	TAAAG	S000387
DOFCOREZM		809	(+)	AAAG	S000265
CACTFTPPCA1		811	(-)	YACT	S000449
CURECORECR		814	(-)	GTAC	S000493
CURECORECR		814	(+)	GTAC	S000493
LTRECOREATCOR15		828	(+)	CCGAC	S000153
PRECONSCRHSP70A		828	(+)	SCGAYNRNNNNNNNNNNNNNNNHD	S000506
CACTFTPPCA1		833	(+)	YACT	S000449
EBOXBNNAPA		841	(-)	CANNTG	S000144
MYCCONSENSUSAT		841	(-)	CANNTG	S000407
EBOXBNNAPA		841	(+)	CANNTG	S000144
MYCCONSENSUSAT		841	(+)	CANNTG	S000407
CACTFTPPCA1		848	(-)	YACT	S000449
PRECONSCRHSP70A		865	(+)	SCGAYNRNNNNNNNNNNNNNNNHD	S000506
GATABOX		867	(+)	GATA	S000039
CACTFTPPCA1		889	(-)	YACT	S000449
CAATBOX1		897	(-)	CAAT	S000028
WBOXATNPR1		898	(+)	TTGAC	S000390
BIHD1OS		899	(-)	TGTCA	S000498
WRKY71OS		899	(+)	TGAC	S000447
CAATBOX1		902	(+)	CAAT	S000028
BOXIINTPATPB		904	(+)	ATAGAA	S000296
POLLEN1LELAT52		906	(+)	AGAAA	S000245
EECCRCAH1		907	(-)	GANTTNC	S000494
DOFCOREZM		917	(-)	AAAG	S000265
S1FSORPL21		927	(+)	ATGGTATT	S000215
S1FBOXSORPS1L21		927	(+)	ATGGTA	S000223
GT1CONSENSUS		932	(-)	GRWAAW	S000198
POLLEN1LELAT52		934	(-)	AGAAA	S000245
PYRIMIDINEBOXOSRAMY1A		942	(+)	CCTTTT	S000259
DOFCOREZM		943	(-)	AAAG	S000265
POLLEN1LELAT52		945	(-)	AGAAA	S000245
CACTFTPPCA1		954	(+)	YACT	S000449
GT1CONSENSUS		961	(-)	GRWAAW	S000198
IBOXCORE		962	(-)	GATAA	S000199
GATABOX		963	(-)	GATA	S000039

GTGANTG10	965	(-)	GTGA	S000378
CACTFTPPCA1	966	(+)	YACT	S000449
CACTFTPPCA1	970	(-)	YACT	S000449
PYRIMIDINEBOXOSRAMY1A	978	(-)	CCTTTT	S000259
DOFCOREZM	979	(+)	AAAG	S000265
NODCON2GM	989	(-)	CTCTT	S000462
OSE2ROOTNODULE	989	(-)	CTCTT	S000468
SURECOREATSULTR11	993	(+)	GAGAC	S000499
EECCRCAH1	1000	(+)	GANTTNC	S000494
GT1CONSENSUS	1015	(+)	GRWAAW	S000198
MYB1LEPR	1021	(-)	GTTAGTT	S000443
DOFCOREZM	1038	(+)	AAAG	S000265
CACTFTPPCA1	1040	(-)	YACT	S000449
NODCON2GM	1046	(+)	CTCTT	S000462
OSE2ROOTNODULE	1046	(+)	CTCTT	S000468
PREATPRODH	1053	(+)	ACTCAT	S000450
NODCON2GM	1059	(+)	CTCTT	S000462
OSE2ROOTNODULE	1059	(+)	CTCTT	S000468
DOFCOREZM	1061	(-)	AAAG	S000265
POLASIG1	1063	(-)	AATAAA	S000080
MARTBOX	1064	(+)	TTWTWTTWTT	S000067
TATABOX5	1064	(+)	TTATTT	S000203
POLASIG1	1068	(-)	AATAAA	S000080
TATABOX5	1069	(+)	TTATTT	S000203
GT1CONSENSUS	1071	(-)	GRWAAW	S000198
POLLEN1LELAT52	1073	(-)	AGAAA	S000245
DOFCOREZM	1076	(-)	AAAG	S000265
DOFCOREZM	1083	(-)	AAAG	S000265
LTRE1HVBLT49	1084	(-)	CCGAAA	S000250
DOFCOREZM	1105	(-)	AAAG	S000265
GT1CONSENSUS	1106	(-)	GRWAAW	S000198
GT1GMSCAM4	1106	(-)	GAAAAA	S000453
POLLEN1LELAT52	1108	(-)	AGAAA	S000245
WBOXNTCHN48	1111	(+)	CTGACY	S000508
WRKY71OS	1112	(+)	TGAC	S000447
WBOXNTERF3	1112	(+)	TGACY	S000457
CAATBOX1	1117	(-)	CAAT	S000028
WBOXHVISO1	1124	(-)	TGACT	S000442
WBOXNTERF3	1124	(-)	TGACY	S000457
WRKY71OS	1125	(-)	TGAC	S000447
GTGANTG10	1126	(-)	GTGA	S000378
POLLEN1LELAT52	1132	(+)	AGAAA	S000245
DOFCOREZM	1135	(+)	AAAG	S000265
WBOXHVISO1	1137	(-)	TGACT	S000442
WBOXNTERF3	1137	(-)	TGACY	S000457
WBOXNTCHN48	1137	(-)	CTGACY	S000508
WRKY71OS	1138	(-)	TGAC	S000447
CACTFTPPCA1	1141	(-)	YACT	S000449
POLLEN1LELAT52	1150	(+)	AGAAA	S000245
GT1CONSENSUS	1151	(+)	GRWAAW	S000198
WRKY71OS	1162	(+)	TGAC	S000447
WBOXNTERF3	1162	(+)	TGACY	S000457
ARR1AT	1175	(-)	NGATT	S000454
CACTFTPPCA1	1183	(-)	YACT	S000449
CURECORECR	1184	(-)	GTAC	S000493
CURECORECR	1184	(+)	GTAC	S000493
ROOTMOTIFTAPOX1	1190	(-)	ATATT	S000098
CACTFTPPCA1	1198	(-)	YACT	S000449
ERELEE4	1206	(+)	AWTTCAAA	S000037
ROOTMOTIFTAPOX1	1212	(-)	ATATT	S000098
GATABOX	1214	(-)	GATA	S000039
MARTBOX	1223	(-)	TTWTWTTWTT	S000067
MARTBOX	1224	(-)	TTWTWTTWTT	S000067
MARTBOX	1225	(-)	TTWTWTTWTT	S000067
MARTBOX	1226	(-)	TTWTWTTWTT	S000067
MARTBOX	1227	(-)	TTWTWTTWTT	S000067
ARR1AT	1235	(-)	NGATT	S000454
NODCON2GM	1239	(-)	CTCTT	S000462

OSE2ROOTNODULE		1239 (-)	CTCTT	S000468
POLLEN1LELAT52		1242 (+)	AGAAA	S000245
GT1CONSENSUS		1243 (+)	GRWAAW	S000198
GT1GMSCAM4		1243 (+)	GAAAAA	S000453
ANAERO1CONSENSUS		1246 (+)	AAACAAA	S000477
BP5OSWX		1255 (+)	CAACGTG	S000436
QARBNEXTA		1256 (+)	AACGTGT	S000244
T/GBOXATPIN2		1256 (+)	AACGTG	S000458
ABRERATCAL		1256 (+)	MACGYGB	S000507
ACGTATERD1		1257 (-)	ACGT	S000415
ABRELATERD1		1257 (+)	ACGTG	S000414
ACGTATERD1		1257 (+)	ACGT	S000415
GTGANTG10		1261 (+)	GTGA	S000378
GATABOX		1263 (+)	GATA	S000039
GATABOX		1265 (-)	GATA	S000039
MYBST1		1265 (-)	GGATA	S000180
TATCCAOSAMY		1265 (+)	TATCCA	S000403
TBOXATGAPB		1269 (-)	ACTTTG	S000383
DOFCOREZM		1270 (+)	AAAG	S000265
CACTFTPPCA1		1272 (-)	YACT	S000449
ARR1AT		1290 (-)	NGATT	S000454
GTGANTG10		1292 (-)	GTGA	S000378
CACTFTPPCA1		1293 (+)	YACT	S000449
POLASIG1		1310 (-)	AATAAA	S000080
TATABOX5		1311 (+)	TTATTT	S000203
SEBFCONSSTPR10A		1316 (+)	YTGTCWC	S000391
ARFAT		1317 (+)	TGTCTC	S000270
SURECOREATSULTR11		1318 (-)	GAGAC	S000499
NODCON2GM		1320 (+)	CTCTT	S000462
OSE2ROOTNODULE		1320 (+)	CTCTT	S000468
ARR1AT		1327 (-)	NGATT	S000454
TATABOX5		1331 (-)	TTATTT	S000203
GARE2OSREP1		1334 (+)	TAACGTA	S000420
ACGTATERD1		1336 (-)	ACGT	S000415
ACGTATERD1		1336 (+)	ACGT	S000415
ROOTMOTIFTAPOX1		1354 (+)	ATATT	S000098
TATABOX4		1357 (-)	TATATAA	S000111
TATAPVTRNALEU		1358 (-)	TTTATATA	S000340
TATABOX4		1358 (+)	TATATAA	S000111
TATABOX2		1360 (+)	TATAAAT	S000109
GT1CONSENSUS		1370 (+)	GRWAAW	S000198
GT1CONSENSUS		1371 (+)	GRWAAW	S000198
GT1GMSCAM4		1371 (+)	GAAAAA	S000453
DOFCOREZM		1374 (+)	AAAG	S000265
NODCON1GM		1374 (+)	AAAGAT	S000461
OSE1ROOTNODULE		1374 (+)	AAAGAT	S000467
ARR1AT	missing	1376 (+)	NGATT	S000454
CAATBOX1	MYBox	1378 (-)	CAAT	S000028
GATABOX		1381 (+)	GATA	S000039
GT1CONSENSUS		1381 (+)	GRWAAW	S000198
IBOXCORE		1381 (+)	GATAA	S000199
DOFCOREZM		1385 (+)	AAAG	S000265
POLLEN1LELAT52		1387 (+)	AGAAA	S000245
GT1CONSENSUS		1388 (+)	GRWAAW	S000198
GT1GMSCAM4		1388 (+)	GAAAAA	S000453
TATABOX5		1391 (-)	TTATTT	S000203
POLASIG3		1392 (+)	AATAAT	S000088
POLASIG1		1397 (-)	AATAAA	S000080
CACTFTPPCA1		1404 (-)	YACT	S000449
CURECORECR		1405 (-)	GTAC	S000493
CURECORECR		1405 (+)	GTAC	S000493
CACTFTPPCA1		1410 (+)	YACT	S000449
CPBCSPOR		1412 (-)	TATTAG	S000491
CAATBOX1		1419 (+)	CAAT	S000028
NODCON2GM		1424 (-)	CTCTT	S000462
OSE2ROOTNODULE		1424 (-)	CTCTT	S000468
PYRIMIDINEBOXHVEPB1		1428 (-)	TTTTTTCC	S000298
GT1CONSENSUS		1428 (+)	GRWAAW	S000198

GT1CONSENSUS	1429	(+)	GRWAAW	S000198
GT1GMSCAM4	1429	(+)	GAAAAA	S000453
POLASIG2	1434	(+)	AATTAAA	S000081
TAAAGSTKST1	1437	(+)	TAAAG	S000387
DOFCOREZM	1438	(+)	AAAG	S000265
ROOTMOTIFTAPOX1	1445	(-)	ATATT	S000098
RAV1AAT	1453	(-)	CAACA	S000314
GTGANTG10	1457	(+)	GTGA	S000378
WBOXHVISO1	1458	(+)	TGACT	S000442
WRKY71OS	1458	(+)	TGAC	S000447
WBOXNTERF3	1458	(+)	TGACY	S000457
GTGANTG10	1465	(+)	GTGA	S000378
ARR1AT	1471	(+)	NGATT	S000454
AUXRETGA1GMGH3	1474	(-)	TGACGTAA	S000234
TGACGTVMAMY	1476	(-)	TGACGT	S000377
ACGTATERD1	1476	(-)	ACGT	S000415
HEXMOTIFTAH3H4	1476	(+)	ACGTCA	S000053
ACGTATERD1	1476	(+)	ACGT	S000415
ASF1MOTIFCAMV	1477	(-)	TGACG	S000024
WBOXATNPR1	1478	(-)	TTGAC	S000390
WRKY71OS	1478	(-)	TGAC	S000447
CAATBOX1	1480	(+)	CAAT	S000028
GT1CONSENSUS	1482	(-)	GRWAAW	S000198
IBOXCORE	1483	(-)	GATAA	S000199
SREATMSD	1483	(+)	TTATCC	S000470
GATABOX	1484	(-)	GATA	S000039
MYBST1	1484	(-)	GGATA	S000180
TATCCAOSAMY	1484	(+)	TATCCA	S000403
CACTFTPPCA1	1489	(-)	YACT	S000449
EBOXBNNAPA	1495	(-)	CANNTG	S000144
MYCCONSENSUSAT	1495	(-)	CANNTG	S000407
EBOXBNNAPA	1495	(+)	CANNTG	S000144
MYCCONSENSUSAT	1495	(+)	CANNTG	S000407
CACTFTPPCA1	1503	(+)	YACT	S000449
CACTFTPPCA1	1516	(+)	YACT	S000449
TATABOX5	1519	(+)	TTATTT	S000203
GT1CONSENSUS	1527	(-)	GRWAAW	S000198
POLLEN1LELAT52	1533	(+)	AGAAA	S000245
GT1CONSENSUS	1534	(+)	GRWAAW	S000198
GT1GMSCAM4	1534	(+)	GAAAAA	S000453
DOFCOREZM	1538	(+)	AAAG	S000265
GTGANTG10	1545	(+)	GTGA	S000378
MYB1AT	1549	(-)	WAACCA	S000408
ARR1AT	1557	(+)	NGATT	S000454
CACTFTPPCA1	1562	(-)	YACT	S000449
CURECORECR	1563	(-)	GTAC	S000493
CURECORECR	1563	(+)	GTAC	S000493
CAATBOX1	1566	(+)	CAAT	S000028
POLASIG3	1568	(-)	AATAAT	S000088
TATABOX5	1569	(+)	TTATTT	S000203
CACTFTPPCA1	1581	(-)	YACT	S000449
CACTFTPPCA1	1587	(-)	YACT	S000449
CACTFTPPCA1	1592	(-)	YACT	S000449
CACTFTPPCA1	1595	(-)	YACT	S000449
CURECORECR	1596	(-)	GTAC	S000493
CURECORECR	1596	(+)	GTAC	S000493
ARR1AT	1605	(+)	NGATT	S000454
CBFHV	1613	(+)	RYCGAC	S000497
CARGNCAT	1618	(-)	CCWWWWWWWWGG	S000446
CCAATBOX1	1618	(+)	CCAAT	S000030
CARGNCAT	1618	(+)	CCWWWWWWWWGG	S000446
CARGCW8GAT	1619	(-)	CWWWWWWWWG	S000431
CAATBOX1	1619	(+)	CAAT	S000028
CARGCW8GAT	1619	(+)	CWWWWWWWWG	S000431
POLASIG1	1620	(+)	AATAAA	S000080
CAATBOX1	1625	(-)	CAAT	S000028
CCAATBOX1	1625	(-)	CCAAT	S000030
NODCON2GM	1630	(-)	CTCTT	S000462

OSE2ROOTNODULE		1630	(-)	CTCTT	S000468
GT1CONSENSUS		1634	(+)	GRWAAW	S000198
TATABOX5		1636	(-)	TTATTT	S000203
POLASIG1		1637	(+)	AATAAA	S000080
PYRIMIDINEBOXOSRAMY1A		1640	(-)	CCTTTT	S000259
DOFCOREZM		1641	(+)	AAAG	S000265
GTGANTG10	← missing	1654	(-)	GTGA	S000378
POLASIG3	MYBox	1670	(+)	AATAAT	S000088
AMMORESIVDCRNIA1		1679	(+)	CGAACTT	S000375
GAREAT		1683	(-)	TAACAAR	S000439
CPBCSPOR		1699	(-)	TATTAG	S000491
POLASIG3	← missing	1701	(+)	AATAAT	S000088
ARR1AT	CAAT Box	1714	(-)	NGATT	S000454
ROOTMOTIFTAPOX1		1726	(-)	ATATT	S000098
ROOTMOTIFTAPOX1		1727	(+)	ATATT	S000098
ROOTMOTIFTAPOX1		1732	(+)	ATATT	S000098
GTGANTG10		1736	(-)	GTGA	S000378
POLLEN1LELAT52		1741	(+)	AGAAA	S000245
UP2ATMSD		1743	(+)	AAACCCTA	S000472
GATABOX		1755	(-)	GATA	S000039
MYBST1		1755	(-)	GGATA	S000180
BOXIINTPATPB		1778	(-)	ATAGAA	S000296
SEF1MOTIF		1781	(-)	ATATTTAWW	S000006
TATABOX2		1781	(+)	TATAAAT	S000109
ROOTMOTIFTAPOX1		1785	(-)	ATATT	S000098
ROOTMOTIFTAPOX1		1786	(+)	ATATT	S000098
POLASIG2		1792	(+)	AATTAAA	S000081
SEF1MOTIF		1793	(-)	ATATTTAWW	S000006
TATABOXOSPAL		1794	(-)	TATTTAA	S000400
ROOTMOTIFTAPOX1		1797	(-)	ATATT	S000098
SEF1MOTIF		1798	(+)	ATATTTAWW	S000006
ROOTMOTIFTAPOX1		1798	(+)	ATATT	S000098
TATABOXOSPAL		1799	(+)	TATTTAA	S000400
POLASIG2		1801	(-)	AATTAAA	S000081
TAAAGSTKST1		1808	(+)	TAAAG	S000387
DOFCOREZM		1809	(+)	AAAG	S000265
GTGANTG10		1814	(+)	GTGA	S000378
CACTFTPPCA1		1817	(-)	YACT	S000449
POLASIG3		1820	(-)	AATAAT	S000088
TATABOX5		1821	(+)	TTATTT	S000203
TATABOXOSPAL		1822	(+)	TATTTAA	S000400
CURECORECR		1841	(-)	GTAC	S000493
CURECORECR		1841	(+)	GTAC	S000493
CACTFTPPCA1		1842	(+)	YACT	S000449
RAV1AAT		1846	(-)	CAACA	S000314
TATCCAOSAMY		1849	(-)	TATCCA	S000403
MYBST1		1850	(+)	GGATA	S000180
GATABOX		1851	(+)	GATA	S000039
UP2ATMSD		1853	(-)	AAACCCTA	S000472
MYBCORE		1870	(-)	CNGTTR	S000176
MYB2AT		1870	(+)	TAACTG	S000177
MYB2CONSENSUSAT		1870	(+)	YAACKG	S000409
ROOTMOTIFTAPOX1		1893	(+)	ATATT	S000098
CACTFTPPCA1		1898	(+)	YACT	S000449
WUSATAg		1903	(-)	TTAATGG	S000433
GT1CORE		1906	(-)	GGTTAA	S000125
SEF4MOTIFGM7S		1914	(-)	RTTTTTR	S000103
ARR1AT		1918	(-)	NGATT	S000454
CAATBOX1		1921	(+)	CAAT	S000028
ARR1AT		1922	(-)	NGATT	S000454
PYRIMIDINEBOXHVEPB1		1928	(-)	TTTTTTCC	S000298
GT1CONSENSUS		1928	(+)	GRWAAW	S000198
GT1CONSENSUS		1929	(+)	GRWAAW	S000198
GT1GMSCAM4		1929	(+)	GAAAAA	S000453
DOFCOREZM		1933	(+)	AAAG	S000265
NODCON2GM		1934	(-)	CTCTT	S000462
OSE2ROOTNODULE		1934	(-)	CTCTT	S000468
POLLEN1LELAT52		1937	(+)	AGAAA	S000245

CACTFTPPCA1		1944	(-)	YACT	S000449
TBOXATGAPB		1953	(+)	ACTTTG	S000383
DOFCOREZM		1954	(-)	AAAG	S000265
ANAERO1CONSENSUS		1955	(-)	AAACAAA	S000477
POLASIG3		1965	(-)	AATAAT	S000088
TATABOX5		1966	(+)	TTATTT	S000203
GT1CONSENSUS		1972	(+)	GRWAAW	S000198
LECPLEACS2		1983	(+)	TAAAATAT	S000465
ROOTMOTIFTAPOX1		1986	(-)	ATATT	S000098
BIHD1OS		1990	(-)	TGTCA	S000498
WRKY71OS		1990	(+)	TGAC	S000447
CAATBOX1		1993	(+)	CAAT	S000028
GT1CONSENSUS		1995	(-)	GRWAAW	S000198
POLLEN1LELAT52		1997	(-)	AGAAA	S000245
CANBNNAPA		2000	(+)	CNAACAC	S000148
CACTFTPPCA1		2004	(+)	YACT	S000449
TBOXATGAPB		2005	(+)	ACTTTG	S000383
DOFCOREZM		2006	(-)	AAAG	S000265
POLLEN1LELAT52		2020	(-)	AGAAA	S000245
GTGANTG10		2032	(-)	GTGA	S000378
NODCON1GM		2036	(-)	AAAGAT	S000461
OSE1ROOTNODULE		2036	(-)	AAAGAT	S000467
DOFCOREZM		2038	(-)	AAAG	S000265
TAAAGSTKST1	missing	2038	(-)	TAAAG	S000387
ARR1AT	MYBox	2043	(+)	NGATT	S000454
CAATBOX1		2045	(-)	CAAT	S000028
ARR1AT		2047	(+)	NGATT	S000454
GT1CONSENSUS		2050	(-)	GRWAAW	S000198
IBOXCORE		2051	(-)	GATAA	S000199
GATABOX		2052	(-)	GATA	S000039
EBOXBNNAPA		2055	(-)	CANNTG	S000144
MYCCONSENSUSAT		2055	(-)	CANNTG	S000407
EBOXBNNAPA		2055	(+)	CANNTG	S000144
MYCCONSENSUSAT		2055	(+)	CANNTG	S000407
WBOXNTERF3		2060	(-)	TGACY	S000457
WRKY71OS		2061	(-)	TGAC	S000447
GTGANTG10		2062	(-)	GTGA	S000378
CACTFTPPCA1		2063	(+)	YACT	S000449
CACTFTPPCA1		2069	(-)	YACT	S000449
GT1CONSENSUS		2084	(-)	GRWAAW	S000198
IBOXCORE		2085	(-)	GATAA	S000199
GATABOX		2086	(-)	GATA	S000039
INRNTPSADB		2089	(+)	YTCANTYY	S000395
CAATBOX1		2091	(+)	CAAT	S000028
AMYBOX1		2094	(-)	TAACARA	S000020
MYBGAHV		2094	(-)	TAACAAA	S000181
GAREAT		2094	(-)	TAACAAR	S000439
IBOXCORE		2098	(-)	GATAA	S000199
GATABOX		2099	(-)	GATA	S000039
NODCON1GM		2100	(-)	AAAGAT	S000461
OSE1ROOTNODULE		2100	(-)	AAAGAT	S000467
DOFCOREZM		2102	(-)	AAAG	S000265
POLLEN1LELAT52		2103	(-)	AGAAA	S000245
DOFCOREZM		2106	(-)	AAAG	S000265
POLASIG1		2110	(-)	AATAAA	S000080
CAATBOX1		2117	(+)	CAAT	S000028
ARR1AT		2118	(-)	NGATT	S000454
NODCON1GM		2119	(-)	AAAGAT	S000461
OSE1ROOTNODULE		2119	(-)	AAAGAT	S000467
DOFCOREZM		2121	(-)	AAAG	S000265
GT1CONSENSUS		2122	(-)	GRWAAW	S000198
RAV1AAT		2127	(+)	CAACA	S000314
DOFCOREZM		2131	(+)	AAAG	S000265
CAATBOX1		2140	(+)	CAAT	S000028
GT1CONSENSUS		2142	(-)	GRWAAW	S000198
GT1CONSENSUS		2143	(-)	GRWAAW	S000198
POLLEN1LELAT52		2150	(+)	AGAAA	S000245
GT1CONSENSUS		2151	(+)	GRWAAW	S000198

TATABOX5		2153	(-)	TTATTT	S000203
SP8BFIBSP8BIB		2160	(+)	TACTATT	S000184
CACTFTPPCA1		2160	(+)	YACT	S000449
CPBCSPOR		2163	(+)	TATTAG	S000491
WRKY71OS	← missing	2174	(+)	TGAC	S000447
WBOXNTERF3	CAAT Box	2174	(+)	TGACY	S000457
EBOXBNNAPA		2178	(-)	CANNTG	S000144
MYCCONSENSUSAT		2178	(-)	CANNTG	S000407
EBOXBNNAPA		2178	(+)	CANNTG	S000144
MYCCONSENSUSAT		2178	(+)	CANNTG	S000407
-300ELEMENT		2182	(+)	TGHAAARK	S000122
GT1CONSENSUS		2183	(+)	GRWAAW	S000198
GT1GMSCAM4		2183	(+)	GAAAAA	S000453
ARR1AT		2187	(-)	NGATT	S000454
NODCON1GM		2200	(-)	AAAGAT	S000461
OSE1ROOTNODULE		2200	(-)	AAAGAT	S000467
DOFCOREZM		2202	(-)	AAAG	S000265
POLLEN1LELAT52		2203	(-)	AGAAA	S000245
GATABOX		2213	(-)	GATA	S000039
CURECORECR		2226	(-)	GTAC	S000493
CURECORECR		2226	(+)	GTAC	S000493
CACTFTPPCA1		2227	(+)	YACT	S000449
DOFCOREZM		2233	(+)	AAAG	S000265
POLLEN1LELAT52		2235	(+)	AGAAA	S000245
GT1CONSENSUS		2236	(+)	GRWAAW	S000198
GT1GMSCAM4		2236	(+)	GAAAAA	S000453
DOFCOREZM		2239	(+)	AAAG	S000265
GTGANTG10		2261	(-)	GTGA	S000378
ARR1AT		2266	(+)	NGATT	S000454
EECCRCAH1		2267	(+)	GANTTNC	S000494
DOFCOREZM		2273	(-)	AAAG	S000265
CCA1ATLHCB1		2279	(+)	AAMAATCT	S000149
ARR1AT		2282	(-)	NGATT	S000454
POLLEN1LELAT52		2287	(+)	AGAAA	S000245
GT1CONSENSUS		2288	(+)	GRWAAW	S000198
GT1GMSCAM4		2288	(+)	GAAAAA	S000453
DOFCOREZM		2292	(+)	AAAG	S000265
CACTFTPPCA1		2294	(-)	YACT	S000449
POLASIG2		2304	(+)	AATTAAA	S000081
TBOXATGAPB		2310	(+)	ACTTTG	S000383
DOFCOREZM		2311	(-)	AAAG	S000265
INRNTPSADB		2340	(-)	YTCANTYY	S000395
POLLEN1LELAT52		2353	(+)	AGAAA	S000245
GT1CONSENSUS		2354	(+)	GRWAAW	S000198
GT1GMSCAM4		2354	(+)	GAAAAA	S000453
NODCON2GM		2363	(+)	CTCTT	S000462
OSE2ROOTNODULE		2363	(+)	CTCTT	S000468
POLLEN1LELAT52		2370	(-)	AGAAA	S000245
ARR1AT		2381	(+)	NGATT	S000454
RYREPEATLEGUMINBOX		2390	(+)	CATGCAY	S000100
RYREPEATGMGY2		2390	(+)	CATGCAT	S000105
RYREPEATBNNAPA		2390	(+)	CATGCA	S000264
EBOXBNNAPA		2394	(-)	CANNTG	S000144
MYCCONSENSUSAT		2394	(-)	CANNTG	S000407
EBOXBNNAPA		2394	(+)	CANNTG	S000144
MYCCONSENSUSAT		2394	(+)	CANNTG	S000407
MYB1AT		2401	(+)	WAACCA	S000408
TATAPVTRNALEU		2415	(-)	TTTATATA	S000340
TATABOX4		2415	(+)	TATATAA	S000111
TAAAGSTKST1		2419	(+)	TAAAG	S000387
DOFCOREZM		2420	(+)	AAAG	S000265
SEF4MOTIFGM7S		2433	(+)	RTTTTTR	S000103
GT1CONSENSUS		2453	(+)	GRWAAW	S000198
GT1GMSCAM4		2453	(+)	GAAAAA	S000453
POLASIG2		2458	(+)	AATTAAA	S000081
TATABOX5		2463	(-)	TTATTT	S000203
POLASIG1		2464	(+)	AATAAA	S000080
DOFCOREZM		2468	(+)	AAAG	S000265

CACTFTPPCA1	2473	(-)	YACT	S000449
CURECORECR	2476	(-)	GTAC	S000493
CURECORECR	2476	(+)	GTAC	S000493
CAATBOX1	2487	(-)	CAAT	S000028
RAV1AAT	2491	(-)	CAACA	S000314
CAATBOX1	2506	(+)	CAAT	S000028
2SSEEDPROTBANAPA	2518	(+)	CAAACAC	S000143
CANBNNAPA	2518	(+)	CNAACAC	S000148
QARBNEXTA	2521	(-)	AACGTGT	S000244
ABRERATCAL	2521	(-)	MACGYGB	S000507
ABRELATERD1	2522	(-)	ACGTG	S000414
T/GBOXATPIN2	2522	(-)	AACGTG	S000458
ACGTATERD1	2523	(-)	ACGT	S000415
ACGTATERD1	2523	(+)	ACGT	S000415
ROOTMOTIFTAPOX1	2540	(-)	ATATT	S000098
BIHD1OS	2544	(+)	TGTCA	S000498
WRKY71OS	2545	(-)	TGAC	S000447
CACTFTPPCA1	2554	(-)	YACT	S000449
GTGANTG10	2555	(+)	GTGA	S000378
BIHD1OS	2556	(-)	TGTCA	S000498
WRKY71OS	2556	(+)	TGAC	S000447
GT1CONSENSUS	2562	(-)	GRWAAW	S000198
POLLEN1LELAT52	2564	(-)	AGAAA	S000245
TAAAGSTKST1	2569	(+)	TAAAG	S000387
DOFCOREZM	2570	(+)	AAAG	S000265
GT1CONSENSUS	2577	(-)	GRWAAW	S000198
GT1GMSCAM4	2577	(-)	GAAAAA	S000453
POLLEN1LELAT52	2579	(-)	AGAAA	S000245
DOFCOREZM	2582	(-)	AAAG	S000265
POLLEN1LELAT52	2584	(-)	AGAAA	S000245
DOFCOREZM	2587	(-)	AAAG	S000265
GT1CONSENSUS	2588	(-)	GRWAAW	S000198
GT1GMSCAM4	2588	(-)	GAAAAA	S000453
POLLEN1LELAT52	2590	(-)	AGAAA	S000245
DOFCOREZM	2593	(-)	AAAG	S000265
CARGCW8GAT	2593	(-)	CWWWWWWWWG	S000431
CARGCW8GAT	2593	(+)	CWWWWWWWWG	S000431
ARR1AT	2609	(-)	NGATT	S000454
GTGANTG10	2611	(-)	GTGA	S000378
NODCON2GM	2625	(-)	CTCTT	S000462
OSE2ROOTNODULE	2625	(-)	CTCTT	S000468
SURECOREATSULTR11	2627	(+)	GAGAC	S000499
RAV1AAT	2636	(+)	CAACA	S000314
GTGANTG10	2648	(+)	GTGA	S000378

//

©2011-2019 National Agriculture and Food Research Organization 1080271
