# Supplementary material for: Whole genome resequencing of four Italian sweet pepper landraces provides insights on sequence variation in genes of agronomic value
Source: Sci Rep. 2020 Jun 8;10:9189. doi: 10.1038/s41598-020-66053-2 (PMC7280500; doi:10.1038/s41598-020-66053-2)
Supplement: Supplementary file 1 — Supplementary File S1. [file 41598_2020_66053_MOESM1_ESM.zip › File_S1/perennial_WUS_PLACE.pdf]

New PLACE

A Database of Plant Cis-acting Regulatory DNA Elements

Fri Feb 28 19:26:17 JST 2020

TTAACATAAATTCTCATTTTTACCCTTGAAGTCTCTTTTATCCAAAAAAAAGAAAAAGCTTCAGTCTCTTTTACTTGTCAAATCATAAGGGTAATTTTGGAAAAAAATTCAACGATTCAT

RESULTS OF YOUR SIGNAL SCAN SEARCH REQUEST

This result is the output of the new signal scan program which was completely rewritten
from a scratch by Akio Miyao ($Id: 649.pl,v 1.11 2016/04/20 08:43:39 miyao Exp $).

The original program of signal scan was reported in
Prestridge, D.S. (1991) SIGNAL SCAN: A computer program that scans DNA sequences for
eukaryotic transcriptional elements. CABIOS 7, 203-206.

193 base pairs

```
 (+) = Current Strand
 (-) = Opposite Strand

1      TTAACATAAATTCTCATTTTTACCCTTGAAGTCTCTTTTATCCAAAAAAA
                   (+) INRNTPSADB S000395 13 YTCANTYY
                      (+) SEF4MOTIFGM7S S000103 16 RTTTTTR
                         (-) GT1CONSENSUS S000198 19 GRWAAW
                                     (-) SURECOREATSULTR11 S000499 31 GAGAC
                                       (+) NODCON2GM S000462 33 CTCTT
                                       (+) OSE2ROOTNODULE S000468 33 CTCTT
                                         (-) DOFCOREZM S000265 35 AAAG
                                           (-) GT1CONSENSUS S000198 37 GRWAAW
                                            (-) IBOXCORE S000199 38 GATAA
                                            (+) SREATMSD S000470 38 TTATCC
                                             (-) GATABOX S000039 39 GATA
                                             (-) MYBST1 S000180 39 GGATA
                                             (+) TATCCAOSAMY S000403 39 TATCCA
                                                 (-) CARGCW8GAT S000431 43 CWWWWWWWWG
                                                 (+) CARGCW8GAT S000431 43 CWWWWWWWWG
                                                       (+) DOFCOREZM S000265 49 AAAG

51     AGAAAAAGCTTCAGTCTCTTTTACTTGTCAAATCATAAGGGTAATTTTGG
       (+) POLLEN1LELAT52 S000245 51 AGAAA
        (+) GT1CONSENSUS S000198 52 GRWAAW
        (+) GT1GMSCAM4 S000453 52 GAAAAA
           (+) DOFCOREZM S000265 55 AAAG
                (+) INRNTPSADB S000395 60 YTCANTYY
                    (-) SURECOREATSULTR11 S000499 64 GAGAC
                      (+) NODCON2GM S000462 66 CTCTT
                      (+) OSE2ROOTNODULE S000468 66 CTCTT
                        (-) DOFCOREZM S000265 68 AAAG
                            (+) CACTFTPPCA1 S000449 72 YACT
                                (+) BIHD1OS S000498 76 TGTCA
                                 (-) WBOXATNPR1 S000390 77 TTGAC
                                 (-) WRKY71OS S000447 77 TGAC
                                     (-) ARR1AT S000454 81 NGATT
                                              (+) GT1CONSENSUS S000198 90 GRWAAW
                                                       (-) PYRIMIDINEBOXHVEPB1 S000298 99 TTTTTTCC
                                                       (+) GT1CONSENSUS S000198 99 GRWAAW
                                                        (+) GT1CONSENSUS S000198 100 GRWAAW
                                                        (+) GT1GMSCAM4 S000453 100 GAAAAA

101    AAAAAAATTCAACGATTCATTTATTTGAAACACCAATAAATACTCCAAC
                   (+) ARR1AT S000454 113 NGATT
                          (-) POLASIG1 S000080 120 AATAAA
                           (+) TATABOX5 S000203 121 TTATTT
                                        (+) CCAATBOX1 S000030 134 CCAAT
                                         (+) CAATBOX1 S000028 135 CAAT
                                          (+) POLASIG1 S000080 136 AATAAA
                                                (+) CACTFTPPCA1 S000449 142 YACT
                                                     (+) RAV1AAT S000314 147 CAACA
                                                        (+) CAATBOX1 S000028 150 CAAT

151    AATTCATTTATTCCGAAATGGAGGGAGTATTTCTTTTAGCGCN
             (-) POLASIG1 S000080 157 AATAAA
                   (+) LTRE1HVBLT49 S000250 163 CCGAAA
                                (-) CACTFTPPCA1 S000449 176 YACT
                                    (-) POLLEN1LELAT52 S000245 180 AGAAA
                                       (-) DOFCOREZM S000265 183 AAAG
```

Factor or Site Name	Loc.(Str.)	Signal Sequence	SITE #
INRNTPSADB	13 (+)	YTCANTYY	S000395
SEF4MOTIFGM7S	16 (+)	RTTTTTR	S000103
GT1CONSENSUS	19 (-)	GRWAAW	S000198
SURECOREATSULTR11	31 (-)	GAGAC	S000499
NODCON2GM	33 (+)	CTCTT	S000462
OSE2ROOTNODULE	33 (+)	CTCTT	S000468
DOFCOREZM	35 (-)	AAAG	S000265
GT1CONSENSUS	37 (-)	GRWAAW	S000198
IBOXCORE	38 (-)	GATAA	S000199
SREATMSD	38 (+)	TTATCC	S000470
GATABOX	39 (-)	GATA	S000039
MYBST1	39 (-)	GGATA	S000180
TATCCAOSAMY	39 (+)	TATCCA	S000403
CARGCW8GAT	43 (-)	CWWWWWWWWG	S000431
CARGCW8GAT	43 (+)	CWWWWWWWWG	S000431
DOFCOREZM	49 (+)	AAAG	S000265
POLLEN1LELAT52	51 (+)	AGAAA	S000245
GT1CONSENSUS	52 (+)	GRWAAW	S000198
GT1GMSCAM4	52 (+)	GAAAAA	S000453
DOFCOREZM	55 (+)	AAAG	S000265
INRNTPSADB	60 (+)	YTCANTYY	S000395
SURECOREATSULTR11	64 (-)	GAGAC	S000499
NODCON2GM	66 (+)	CTCTT	S000462
OSE2ROOTNODULE	66 (+)	CTCTT	S000468
DOFCOREZM	68 (-)	AAAG	S000265
CACTFTPPCA1	72 (+)	YACT	S000449
BIHD1OS	76 (+)	TGTCA	S000498
WBOXATNPR1	77 (-)	TTGAC	S000390
WRKY71OS	77 (-)	TGAC	S000447
ARR1AT	81 (-)	NGATT	S000454
GT1CONSENSUS	90 (+)	GRWAAW	S000198
PYRIMIDINEBOXHVEPB1	99 (-)	TTTTTTCC	S000298
GT1CONSENSUS	99 (+)	GRWAAW	S000198
GT1CONSENSUS	100 (+)	GRWAAW	S000198
GT1GMSCAM4	100 (+)	GAAAAA	S000453
ARR1AT	113 (+)	NGATT	S000454
POLASIG1	120 (-)	AATAAA	S000080
TATABOX5	121 (+)	TTATTT	S000203
CCAATBOX1	134 (+)	CCAAT	S000030
CAATBOX1	135 (+)	CAAT	S000028
POLASIG1	136 (+)	AATAAA	S000080
CACTFTPPCA1	142 (+)	YACT	S000449
RAV1AAT	147 (+)	CAACA	S000314
CAATBOX1	150 (+)	CAAT	S000028
POLASIG1	157 (-)	AATAAA	S000080
LTRE1HVBLT49	163 (+)	CCGAAA	S000250
CACTFTPPCA1	176 (-)	YACT	S000449
POLLEN1LELAT52	180 (-)	AGAAA	S000245
DOFCOREZM	183 (-)	AAAG	S000265

//

©2011-2019 National Agriculture and Food Research Organization 1252070
