# Supplementary material for: Whole genome resequencing of four Italian sweet pepper landraces provides insights on sequence variation in genes of agronomic value
Source: Sci Rep. 2020 Jun 8;10:9189. doi: 10.1038/s41598-020-66053-2 (PMC7280500; doi:10.1038/s41598-020-66053-2)
Supplement: Supplementary file 1 — Supplementary File S1. [file 41598_2020_66053_MOESM1_ESM.zip › File_S1/tumaticot_WUS_PLACE.pdf]

New PLACE

A Database of Plant Cis-acting Regulatory DNA Elements

Tue Jul 23 20:16:27 JST 2019

â□□TTAACATAAATTCTCATTTTTACCCTTGAAGTCTCTTTTATCCAAAAAAAGAAAAARCTTMAGTCTCTTTTACTTGTCAAATCATAAGRGTAATTTTGGAAAAAAAATTCAACGATT

RESULTS OF YOUR SIGNAL SCAN SEARCH REQUEST

This result is the output of the new signal scan program which was completely rewritten
from a scratch by Akio Miyao ($Id: 649.pl,v 1.11 2016/04/20 08:43:39 miyao Exp $).

The original program of signal scan was reported in
Prestridge, D.S. (1991) SIGNAL SCAN: A computer program that scans DNA sequences for
eukaryotic transcriptional elements. CABIOS 7, 203-206.

197 base pairs

```
 (+) = Current Strand
 (-) = Opposite Strand

1      NNNTTAACATAAATTCTCATTTTTACCCTTGAAGTCTCTTTTATCCAAAA
                      (+) INRNTPSADB S000395 16 YTCANTYY
                         (+) SEF4MOTIFGM7S S000103 19 RTTTTTR
                            (-) GT1CONSENSUS S000198 22 GRWAAW
                                        (-) SURECOREATSULTR11 S000499 34 GAGAC
                                          (+) NODCON2GM S000462 36 CTCTT
                                          (+) OSE2ROOTNODULE S000468 36 CTCTT
                                            (-) DOFCOREZM S000265 38 AAAG
                                              (-) GT1CONSENSUS S000198 40 GRWAAW
                                               (-) IBOXCORE S000199 41 GATAA
                                               (+) SREATMSD S000470 41 TTATCC
                                                (-) GATABOX S000039 42 GATA
                                                (-) MYBST1 S000180 42 GGATA
                                                (+) TATCCAOSAMY S000403 42 TATCCA

51     AAAGAAAAANCTTNAGTCTCTTTTACTTGTCAAATCATAAGNGTAATTTT
       (+) DOFCOREZM S000265 51 AAAG
         (+) POLLEN1LELAT52 S000245 53 AGAAA
          (+) GT1CONSENSUS S000198 54 GRWAAW
          (+) GT1GMSCAM4 S000453 54 GAAAAA
                      (-) SURECOREATSULTR11 S000499 66 GAGAC
                        (+) NODCON2GM S000462 68 CTCTT
                        (+) OSE2ROOTNODULE S000468 68 CTCTT
                          (-) DOFCOREZM S000265 70 AAAG
                              (+) CACTFTPPCA1 S000449 74 YACT
                                  (+) BIHD1OS S000498 78 TGTCA
                                   (-) WBOXATNPR1 S000390 79 TTGAC
                                   (-) WRKY71OS S000447 79 TGAC
                                       (-) ARR1AT S000454 83 NGATT

101    GGAAAAAAAATTCAACGATTCATTTATTTTGAAACANCANTAAATACTNNA
       (-) PYRIMIDINEBOXHVEPB1 S000298 101 TTTTTTCC
       (+) GT1CONSENSUS S000198 101 GRWAAW
        (+) GT1CONSENSUS S000198 102 GRWAAW
        (+) GT1GMSCAM4 S000453 102 GAAAAA
                     (+) ARR1AT S000454 115 NGATT
                            (-) POLASIG1 S000080 122 AATAAA
                             (+) TATABOX5 S000203 123 TTATTT
                                                 (+) CACTFTPPCA1 S000449 144 YACT

151    ACAATTNATTTATTNNGAAATGGAGGGAGTATTTCTTTTAGCGCNNN
        (+) CAATBOX1 S000028 152 CAAT
               (-) POLASIG1 S000080 159 AATAAA
                                  (-) CACTFTPPCA1 S000449 178 YACT
                                      (-) POLLEN1LELAT52 S000245 182 AGAAA
                                         (-) DOFCOREZM S000265 185 AAAG
```

Factor or Site Name	Loc.(Str.)	Signal Sequence	SITE #
INRNTPSADB	16 (+)	YTCANTYY	S000395
SEF4MOTIFGM7S	19 (+)	RTTTTTR	S000103
GT1CONSENSUS	22 (-)	GRWAAW	S000198
SURECOREATSULTR11	34 (-)	GAGAC	S000499
NODCON2GM	36 (+)	CTCTT	S000462
OSE2ROOTNODULE	36 (+)	CTCTT	S000468
DOFCOREZM	38 (-)	AAAG	S000265

GT1CONSENSUS	40	(-)	GRWAAW	S000198
IBOXCORE	41	(-)	GATAA	S000199
SREATMSD	41	(+)	TTATCC	S000470
GATABOX	42	(-)	GATA	S000039
MYBST1	42	(-)	GGATA	S000180
TATCCAOSAMY	42	(+)	TATCCA	S000403
DOFCOREZM	51	(+)	AAAG	S000265
POLLEN1LELAT52	53	(+)	AGAAA	S000245
GT1CONSENSUS	54	(+)	GRWAAW	S000198
GT1GMSCAM4	54	(+)	GAAAAA	S000453
SURECOREATSULTR11	66	(-)	GAGAC	S000499
NODCON2GM	68	(+)	CTCTT	S000462
OSE2ROOTNODULE	68	(+)	CTCTT	S000468
DOFCOREZM	70	(-)	AAAG	S000265
CACTFTPPCA1	74	(+)	YACT	S000449
BIHD1OS	78	(+)	TGTCA	S000498
WBOXATNPR1	79	(-)	TTGAC	S000390
WRKY71OS	79	(-)	TGAC	S000447
ARR1AT	83	(-)	NGATT	S000454
PYRIMIDINEBOXHVEPB1	101	(-)	TTTTTTCC	S000298
GT1CONSENSUS	101	(+)	GRWAAW	S000198
GT1CONSENSUS	102	(+)	GRWAAW	S000198
GT1GMSCAM4	102	(+)	GAAAAA	S000453
ARR1AT	115	(+)	NGATT	S000454
POLASIG1	122	(-)	AATAAA	S000080
TATABOX5	123	(+)	TTATTT	S000203
CACTFTPPCA1	144	(+)	YACT	S000449
CAATBOX1	152	(+)	CAAT	S000028
POLASIG1	159	(-)	AATAAA	S000080
CACTFTPPCA1	178	(-)	YACT	S000449
POLLEN1LELAT52	182	(-)	AGAAA	S000245
DOFCOREZM	185	(-)	AAAG	S000265

//

©2011-2019 National Agriculture and Food Research Organization 1077527
