# Supplementary material for: Whole genome resequencing of four Italian sweet pepper landraces provides insights on sequence variation in genes of agronomic value
Source: Sci Rep. 2020 Jun 8;10:9189. doi: 10.1038/s41598-020-66053-2 (PMC7280500; doi:10.1038/s41598-020-66053-2)
Supplement: Supplementary file 3 — Supplementary Tables. [file 41598_2020_66053_MOESM3_ESM.pdf]

Whole genome resequencing of four Italian sweet pepper landraces provides insights on sequence variation in genes of agronomic value.

Alberto Acquadro[1], Lorenzo Barchi[1*], Ezio Portis[1], Mohamed Nourdine[1], Cristiano Carli[2], Simone Monge[3], Danila Valentino[1], Sergio Lanteri[1]

Supplementary Figures

Captions to Supplementary figures and file S1

Figure S1 - Resistance genes dendrograms of the 4 inbred lines plus CM334. Trees are separately provided for CNL, NBS, NL, RLK, RLP and TM families. Branches are colored according to the bootstrap values (red = 15 and green = 100).

Figure S2 - Histograms depicting the number of SNP/Indels present in 60 genes related to berry characteristics in all the analysed pepper genotypes. CDS, INTRON upstream and downstream regions are considered separately.

Figure S3 - Histograms depicting the number of SNP/Indels present in *SUN*, *WUS*, *CLV3*, *CLV1, OVATE*, *KLU* and *CNR* genes in all the analysed pepper genotypes. CDS, INTRON upstream and downstream regions are considered separately.

Figure S4 - Global alignment of the 3kb regions of the *SUN*, *WUS*, *CLV3*, *OVATE* and *CNR* genes in ‘Tumaticot', 'Cuneo', 'Corno', 'Quadrato', 'CM334' and 'Perennial' genotypes.

Figure S5 - Presence absence variation (PAV) in the four resequenced pepper genotypes. A detail of the chr. 9 is shown, where 4 genes (in blue, bottom) are exclusively present in 'Tumaticot' and absent in 'Cuneo', 'Corno' and 'Quadrato'. The four presented genes are related to biotic stress response.

File S1 – Plant cis-acting regulatory DNA elements, derived from the PLACE analysis, on regions of the *WUS* and *CLV3* promoters in ‘Tumaticot', 'Cuneo', 'Corno', 'Quadrato', 'CM334' and 'Perennial' genotypes. Yellow and violet shading is used to highlight the variable presence of CAAT and MYB boxes, respectively.

Figure S1

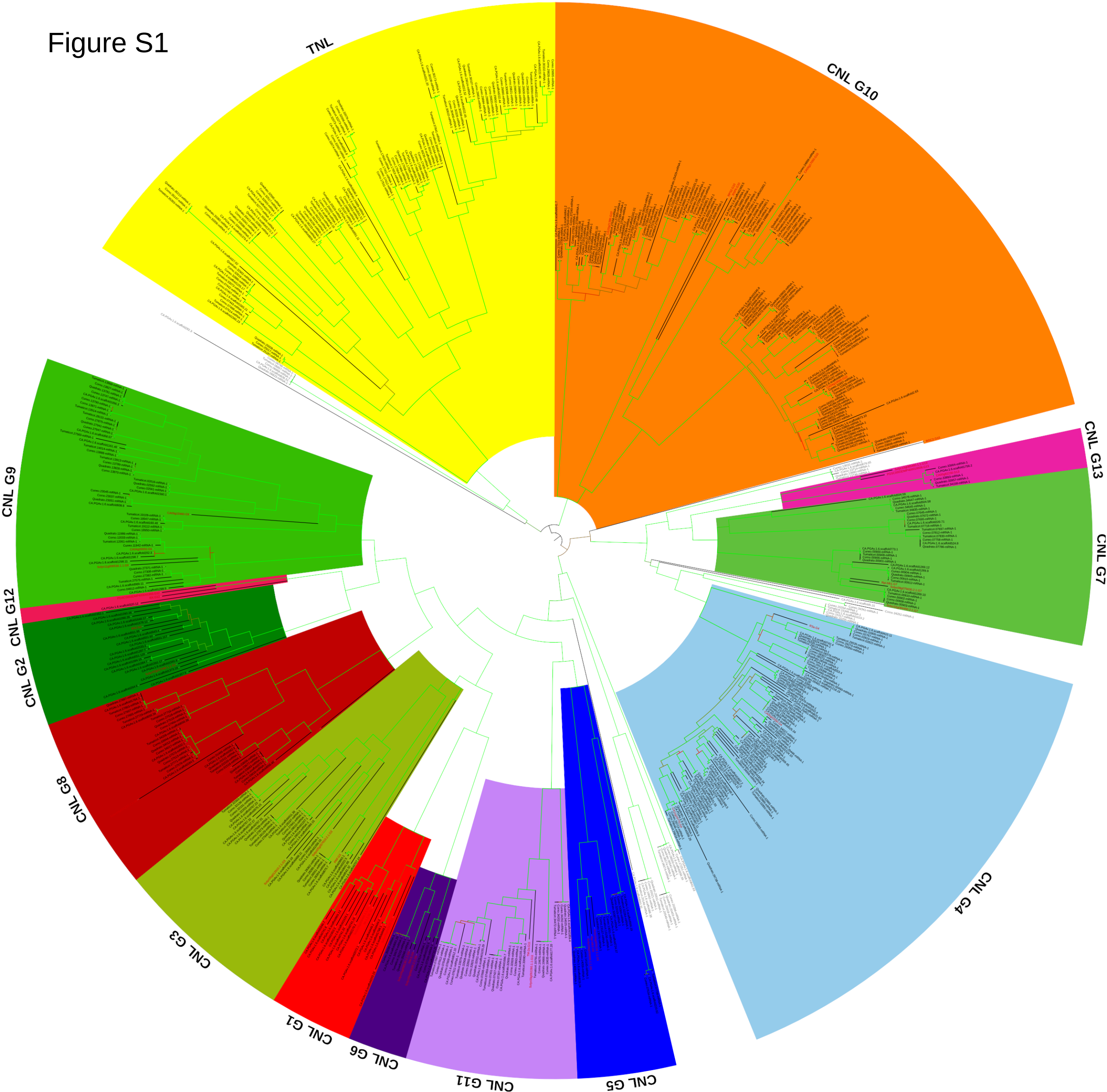
TNL
CNL G10
CNL G13
CNL G7
CNL G4
CNL G5
CNL G11
CNL G6
CNL G1
CNL G3
CNL G8
CNL G2
CNL G12
CNL G9

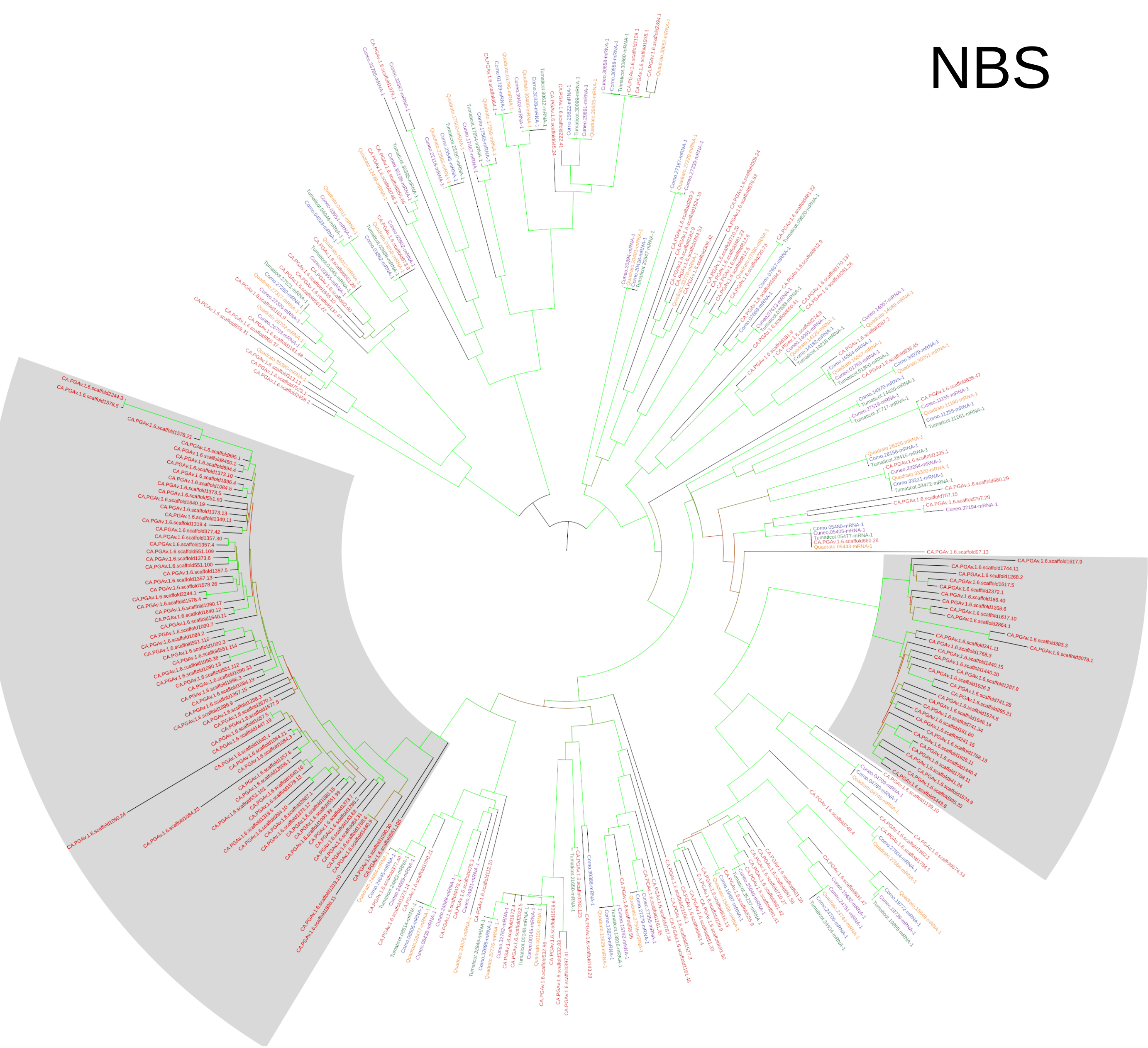
NBS

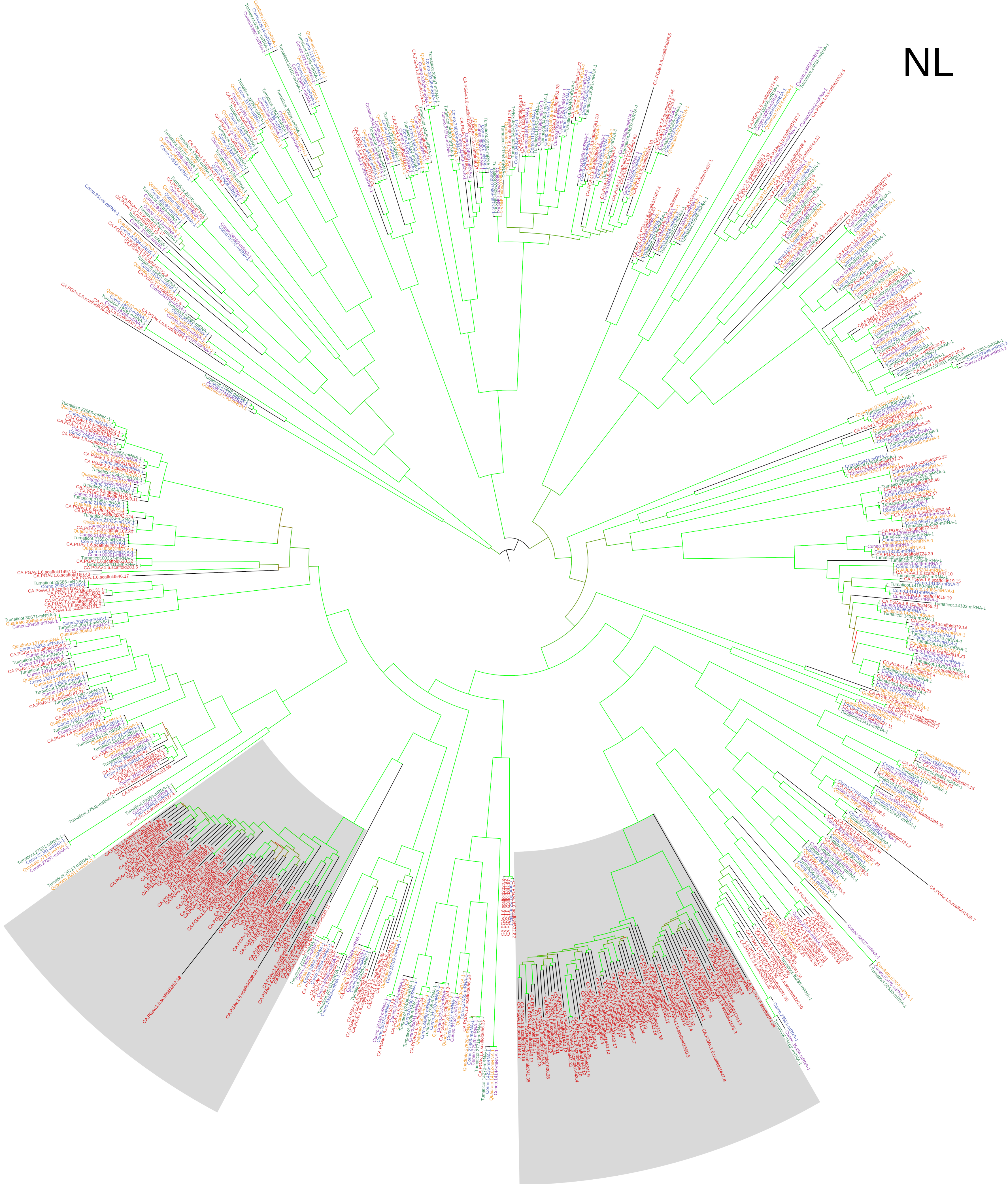
NL

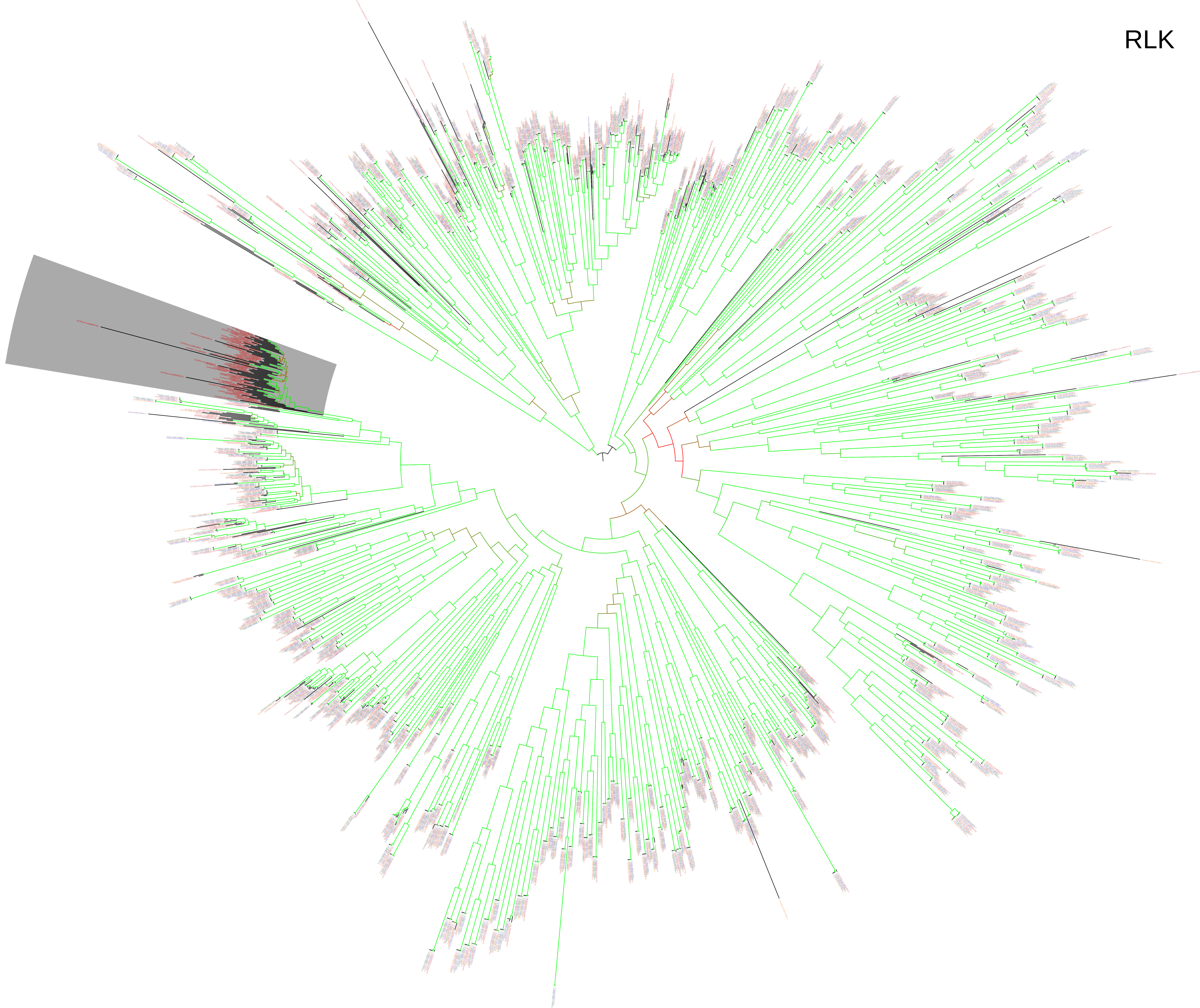
RLK

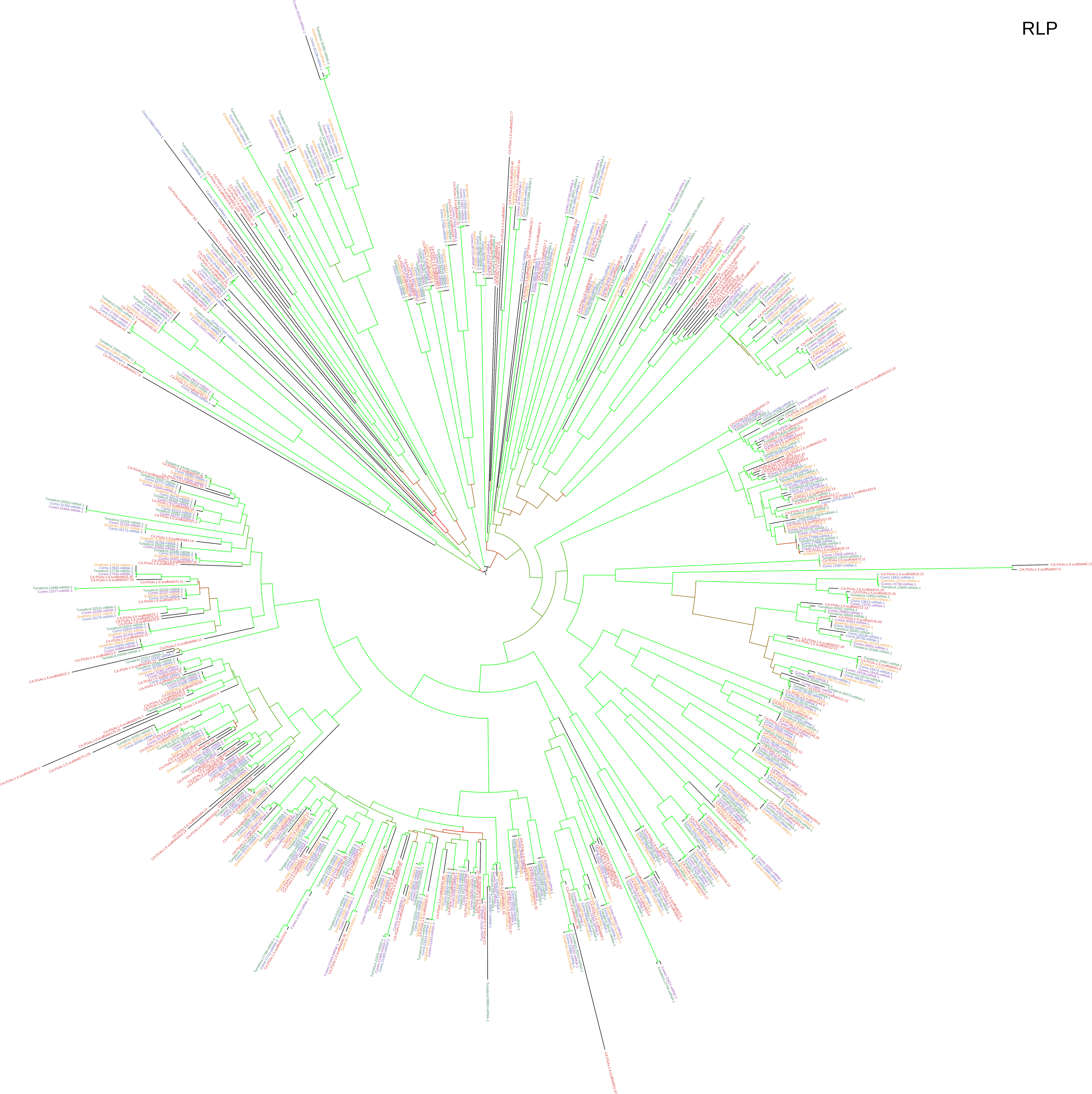
RLP

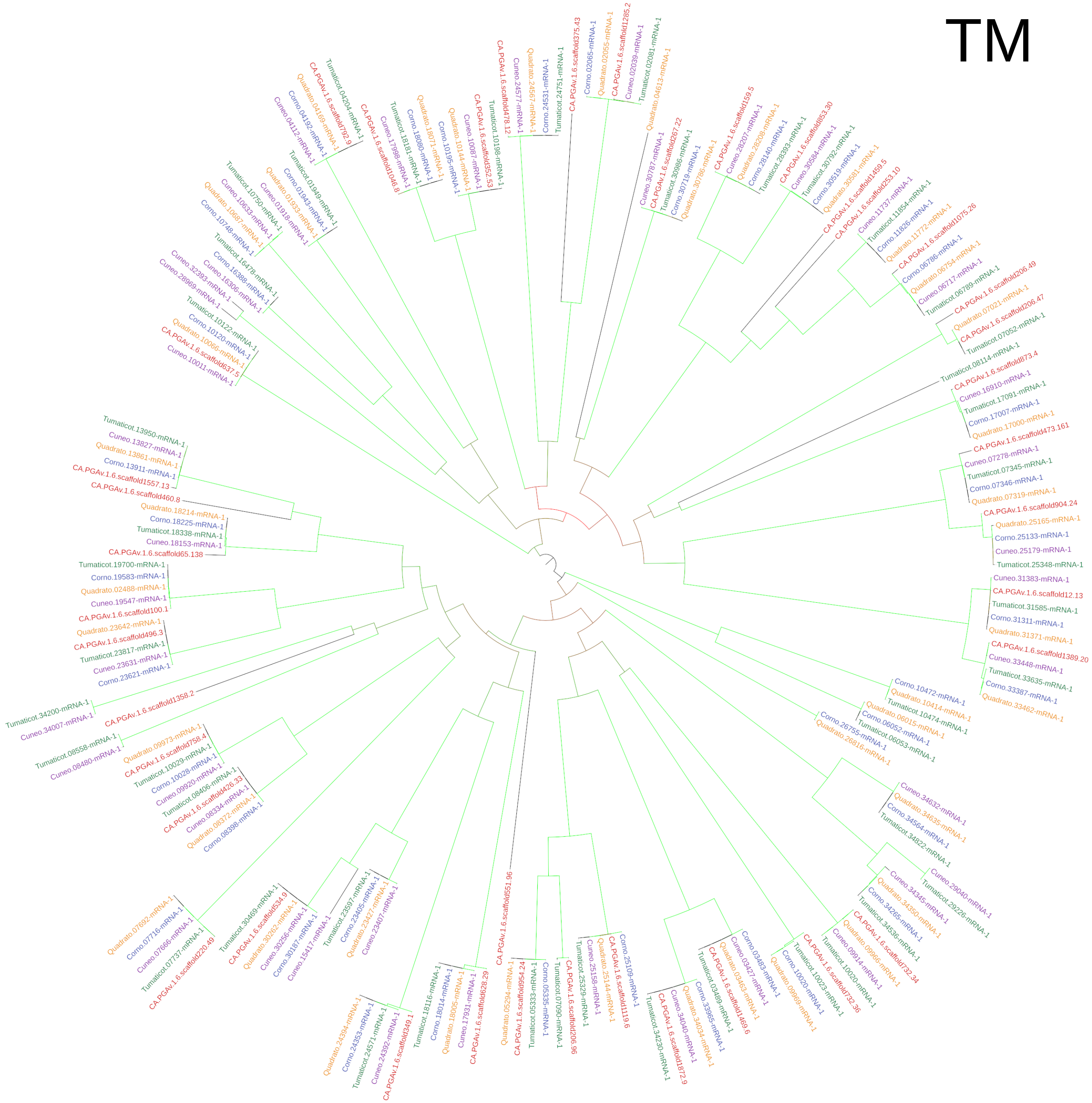
TM
Tumaticot.04204-mRNA-1
CA.PGAv.1.6.scaffold792.9
Quadrato.04169-mRNA-1
Corno.04192-mRNA-1
Cuneo.04112-mRNA-1
CA.PGAv.1.6.scaffold1046.8
Cuneo.17998-mRNA-1
Tumaticot.18181-mRNA-1
Corno.18080-mRNA-1
Quadrato.18071-mRNA-1
Corno.10195-mRNA-1
Quadrato.10144-mRNA-1
Cuneo.10087-mRNA-1
CA.PGAv.1.6.scaffold352.53
Tumaticot.10198-mRNA-1
CA.PGAv.1.6.scaffold478.12
Cuneo.24577-mRNA-1
Quadrato.24567-mRNA-1
Corno.24531-mRNA-1
Tumaticot.24751-mRNA-1
CA.PGAv.1.6.scaffold375.43
Corno.02065-mRNA-1
Quadrato.02055-mRNA-1
CA.PGAv.1.6.scaffold1285.2
Cuneo.02039-mRNA-1
Tumaticot.02081-mRNA-1
Quadrato.04613-mRNA-1
Cuneo.30787-mRNA-1
CA.PGAv.1.6.scaffold267.22
Tumaticot.30986-mRNA-1
Corno.30719-mRNA-1
Quadrato.30786-mRNA-1
CA.PGAv.1.6.scaffold159.5
Cuneo.28207-mRNA-1
Quadrato.28208-mRNA-1
Corno.28140-mRNA-1
Tumaticot.28393-mRNA-1
CA.PGAv.1.6.scaffold853.30
Cuneo.30584-mRNA-1
Tumaticot.30792-mRNA-1
Corno.30519-mRNA-1
Quadrato.30581-mRNA-1
CA.PGAv.1.6.scaffold1459.5
CA.PGAv.1.6.scaffold253.10
Cuneo.11737-mRNA-1
Tumaticot.11854-mRNA-1
Corno.11826-mRNA-1
Quadrato.11772-mRNA-1
CA.PGAv.1.6.scaffold1075.26
Corno.06786-mRNA-1
Quadrato.06754-mRNA-1
Cuneo.06717-mRNA-1
Tumaticot.06789-mRNA-1
CA.PGAv.1.6.scaffold206.49
Quadrato.07021-mRNA-1
CA.PGAv.1.6.scaffold206.47
Tumaticot.07052-mRNA-1
Tumaticot.08114-mRNA-1
CA.PGAv.1.6.scaffold873.4
Cuneo.16910-mRNA-1
Tumaticot.17091-mRNA-1
Corno.17007-mRNA-1
Quadrato.17000-mRNA-1
CA.PGAv.1.6.scaffold473.161
Cuneo.07278-mRNA-1
Tumaticot.07345-mRNA-1
Corno.07346-mRNA-1
Quadrato.07319-mRNA-1
CA.PGAv.1.6.scaffold904.24
Quadrato.25165-mRNA-1
Corno.25133-mRNA-1
Cuneo.25179-mRNA-1
Tumaticot.25348-mRNA-1
Cuneo.31383-mRNA-1
CA.PGAv.1.6.scaffold12.13
Tumaticot.31585-mRNA-1
Corno.31311-mRNA-1
Quadrato.31371-mRNA-1
CA.PGAv.1.6.scaffold1389.20
Cuneo.33448-mRNA-1
Tumaticot.33635-mRNA-1
Corno.33387-mRNA-1
Quadrato.33462-mRNA-1
Corno.10472-mRNA-1
Quadrato.10414-mRNA-1
Tumaticot.10474-mRNA-1
Quadrato.06015-mRNA-1
Corno.06052-mRNA-1
Tumaticot.06053-mRNA-1
Corno.26755-mRNA-1
Quadrato.26816-mRNA-1
Cuneo.34632-mRNA-1
Quadrato.34635-mRNA-1
Corno.34564-mRNA-1
Tumaticot.34822-mRNA-1
Cuneo.29040-mRNA-1
Tumaticot.29226-mRNA-1
Cuneo.34345-mRNA-1
Quadrato.34350-mRNA-1
Corno.34265-mRNA-1
Tumaticot.34536-mRNA-1
CA.PGAv.1.6.scaffold732.34
Quadrato.09966-mRNA-1
Corno.09914-mRNA-1
Tumaticot.10020-mRNA-1
CA.PGAv.1.6.scaffold732.36
Tumaticot.10023-mRNA-1
Corno.10020-mRNA-1
Quadrato.09969-mRNA-1
Corno.03483-mRNA-1
Cuneo.03427-mRNA-1
Quadrato.03463-mRNA-1
CA.PGAv.1.6.scaffold1469.6
Tumaticot.03489-mRNA-1
Corno.33965-mRNA-1
Quadrato.34034-mRNA-1
Cuneo.34040-mRNA-1
CA.PGAv.1.6.scaffold1872.9
Tumaticot.34230-mRNA-1
Corno.25109-mRNA-1
CA.PGAv.1.6.scaffold1119.6
Quadrato.25144-mRNA-1
Cuneo.25158-mRNA-1
Tumaticot.25329-mRNA-1
CA.PGAv.1.6.scaffold206.96
Tumaticot.07090-mRNA-1
Corno.05335-mRNA-1
Tumaticot.05333-mRNA-1
CA.PGAv.1.6.scaffold954.24
Quadrato.05294-mRNA-1
CA.PGAv.1.6.scaffold551.96
CA.PGAv.1.6.scaffold628.29
Cuneo.17931-mRNA-1
Quadrato.18005-mRNA-1
Corno.18014-mRNA-1
Tumaticot.18116-mRNA-1
CA.PGAv.1.6.scaffold349.1
Cuneo.24392-mRNA-1
Tumaticot.24571-mRNA-1
Corno.24353-mRNA-1
Quadrato.24394-mRNA-1
Cuneo.23407-mRNA-1
Quadrato.23427-mRNA-1
Corno.23405-mRNA-1
Tumaticot.23597-mRNA-1
Cuneo.15417-mRNA-1
Corno.30187-mRNA-1
Cuneo.30256-mRNA-1
Quadrato.30262-mRNA-1
CA.PGAv.1.6.scaffold534.9
Tumaticot.30469-mRNA-1
CA.PGAv.1.6.scaffold220.49
Tumaticot.07737-mRNA-1
Cuneo.07666-mRNA-1
Corno.07716-mRNA-1
Quadrato.07692-mRNA-1
Corno.08398-mRNA-1
Quadrato.08372-mRNA-1
Cuneo.08334-mRNA-1
CA.PGAv.1.6.scaffold426.33
Tumaticot.08406-mRNA-1
Cuneo.09920-mRNA-1
Corno.10028-mRNA-1
Tumaticot.10029-mRNA-1
CA.PGAv.1.6.scaffold758.4
Quadrato.09973-mRNA-1
Cuneo.08480-mRNA-1
Tumaticot.08558-mRNA-1
CA.PGAv.1.6.scaffold1358.2
Cuneo.34007-mRNA-1
Tumaticot.34200-mRNA-1
Corno.23621-mRNA-1
Cuneo.23631-mRNA-1
Tumaticot.23817-mRNA-1
CA.PGAv.1.6.scaffold496.3
Quadrato.23642-mRNA-1
CA.PGAv.1.6.scaffold100.1
Cuneo.19547-mRNA-1
Quadrato.02488-mRNA-1
Corno.19583-mRNA-1
Tumaticot.19700-mRNA-1
CA.PGAv.1.6.scaffold65.138
Cuneo.18153-mRNA-1
Tumaticot.18338-mRNA-1
Corno.18225-mRNA-1
Quadrato.18214-mRNA-1
CA.PGAv.1.6.scaffold460.8
CA.PGAv.1.6.scaffold1557.13
Corno.13911-mRNA-1
Quadrato.13861-mRNA-1
Cuneo.13827-mRNA-1
Tumaticot.13950-mRNA-1
Cuneo.10011-mRNA-1
CA.PGAv.1.6.scaffold637.5
Quadrato.10066-mRNA-1
Corno.10120-mRNA-1
Tumaticot.10122-mRNA-1
Cuneo.28969-mRNA-1
Cuneo.32393-mRNA-1
Cuneo.16306-mRNA-1
Corno.16388-mRNA-1
Tumaticot.16478-mRNA-1
Corno.10748-mRNA-1
Quadrato.10687-mRNA-1
Cuneo.10633-mRNA-1
Tumaticot.10750-mRNA-1
Cuneo.01918-mRNA-1
Quadrato.01933-mRNA-1
Corno.01943-mRNA-1
Tumaticot.01949-mRNA-1

Figure S2

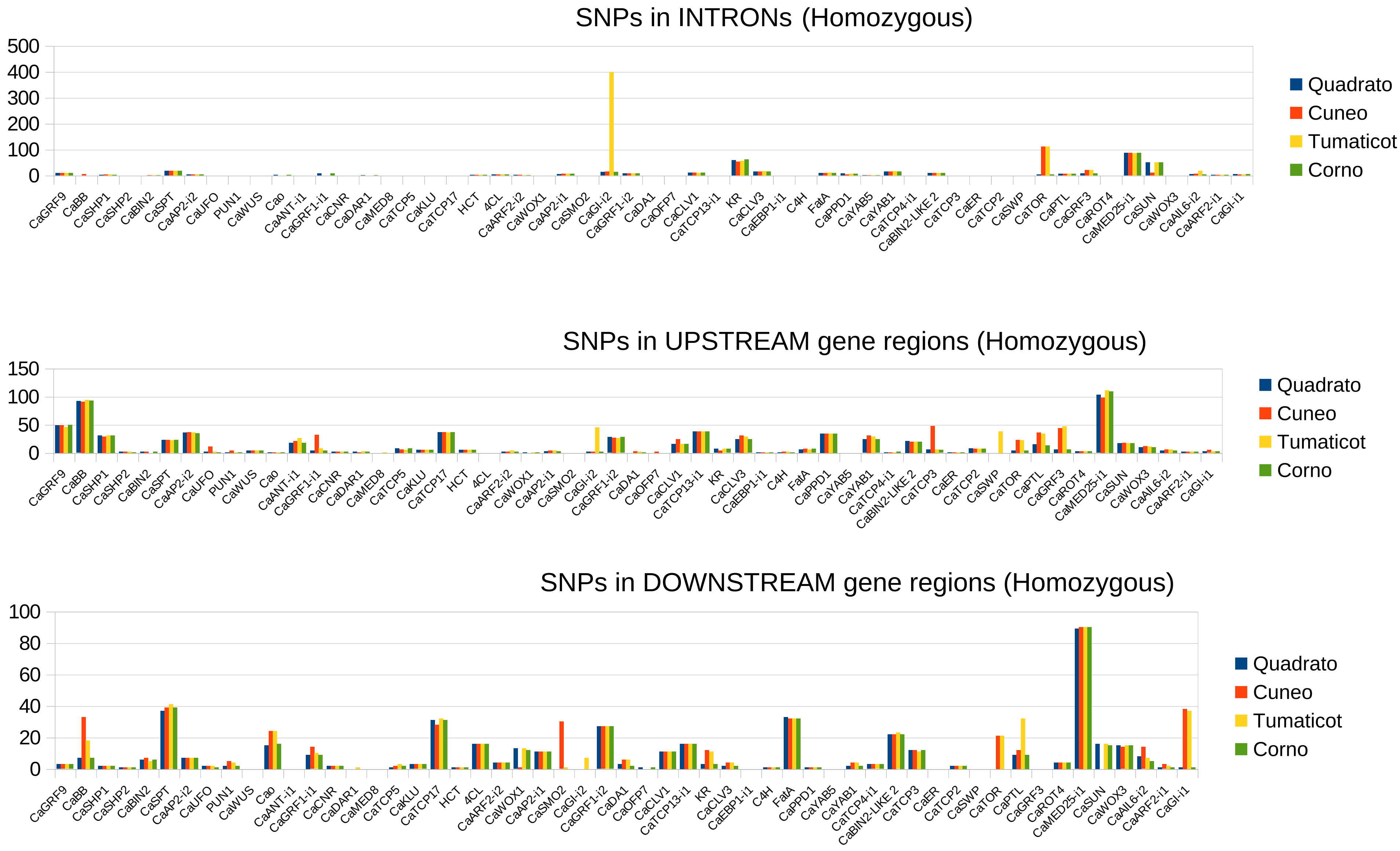
SNPs in INTRONs (Homozygous)
500
400
300
200
100
0
CaGRF9
CaBB
CaSHP1
CaSHP2
CaBIN2
CaSPT
CaAP2-i2
CaUFO
PUN1
CaWUS
Cao
CaANT-i1
CaGRF1-i1
CaCNR
CaDAR1
CaMED8
CaTCP5
CaKLU
CaTCP17
HCT
4CL
CaARF2-i2
CaWOX1
CaAP2-i1
CaSMO2
CaGI-i2
CaGRF1-i2
CaDA1
CaOFP7
CaCLV1
CaTCP13-i1
KR
CaCLV3
CaEBP1-i1
C4H
FatA
CaPPD1
CaYAB5
CaYAB1
CaTCP4-i1
CaBIN2-LIKE 2
CaTCP3
CaER
CaTCP2
CaSWP
CaTOR
CaPTL
CaGRF3
CaROT4
CaMED25-i1
CaSUN
CaWOX3
CaAIL6-i2
CaARF2-i1
CaGI-i1
Quadrato
Cuneo
Tumaticot
Corno
SNPs in UPSTREAM gene regions (Homozygous)
150
100
50
0
CaGRF9
CaBB
CaSHP1
CaSHP2
CaBIN2
CaSPT
CaAP2-i2
CaUFO
PUN1
CaWUS
Cao
CaANT-i1
CaGRF1-i1
CaCNR
CaDAR1
CaMED8
CaTCP5
CaKLU
CaTCP17
HCT
4CL
CaARF2-i2
CaWOX1
CaAP2-i1
CaSMO2
CaGI-i2
CaGRF1-i2
CaDA1
CaOFP7
CaCLV1
CaTCP13-i1
KR
CaCLV3
CaEBP1-i1
C4H
FatA
CaPPD1
CaYAB5
CaYAB1
CaTCP4-i1
CaBIN2-LIKE 2
CaTCP3
CaER
CaTCP2
CaSWP
CaTOR
CaPTL
CaGRF3
CaROT4
CaMED25-i1
CaSUN
CaWOX3
CaAIL6-i2
CaARF2-i1
CaGI-i1
Quadrato
Cuneo
Tumaticot
Corno
SNPs in DOWNSTREAM gene regions (Homozygous)
100
80
60
40
20
0
CaGRF9
CaBB
CaSHP1
CaSHP2
CaBIN2
CaSPT
CaAP2-i2
CaUFO
PUN1
CaWUS
Cao
CaANT-i1
CaGRF1-i1
CaCNR
CaDAR1
CaMED8
CaTCP5
CaKLU
CaTCP17
HCT
4CL
CaARF2-i2
CaWOX1
CaAP2-i1
CaSMO2
CaGI-i2
CaGRF1-i2
CaDA1
CaOFP7
CaCLV1
CaTCP13-i1
KR
CaCLV3
CaEBP1-i1
C4H
FatA
CaPPD1
CaYAB5
CaYAB1
CaTCP4-i1
CaBIN2-LIKE 2
CaTCP3
CaER
CaTCP2
CaSWP
CaTOR
CaPTL
CaGRF3
CaROT4
CaMED25-i1
CaSUN
CaWOX3
CaAIL6-i2
CaARF2-i1
CaGI-i1
Quadrato
Cuneo
Tumaticot
Corno

Figure S2

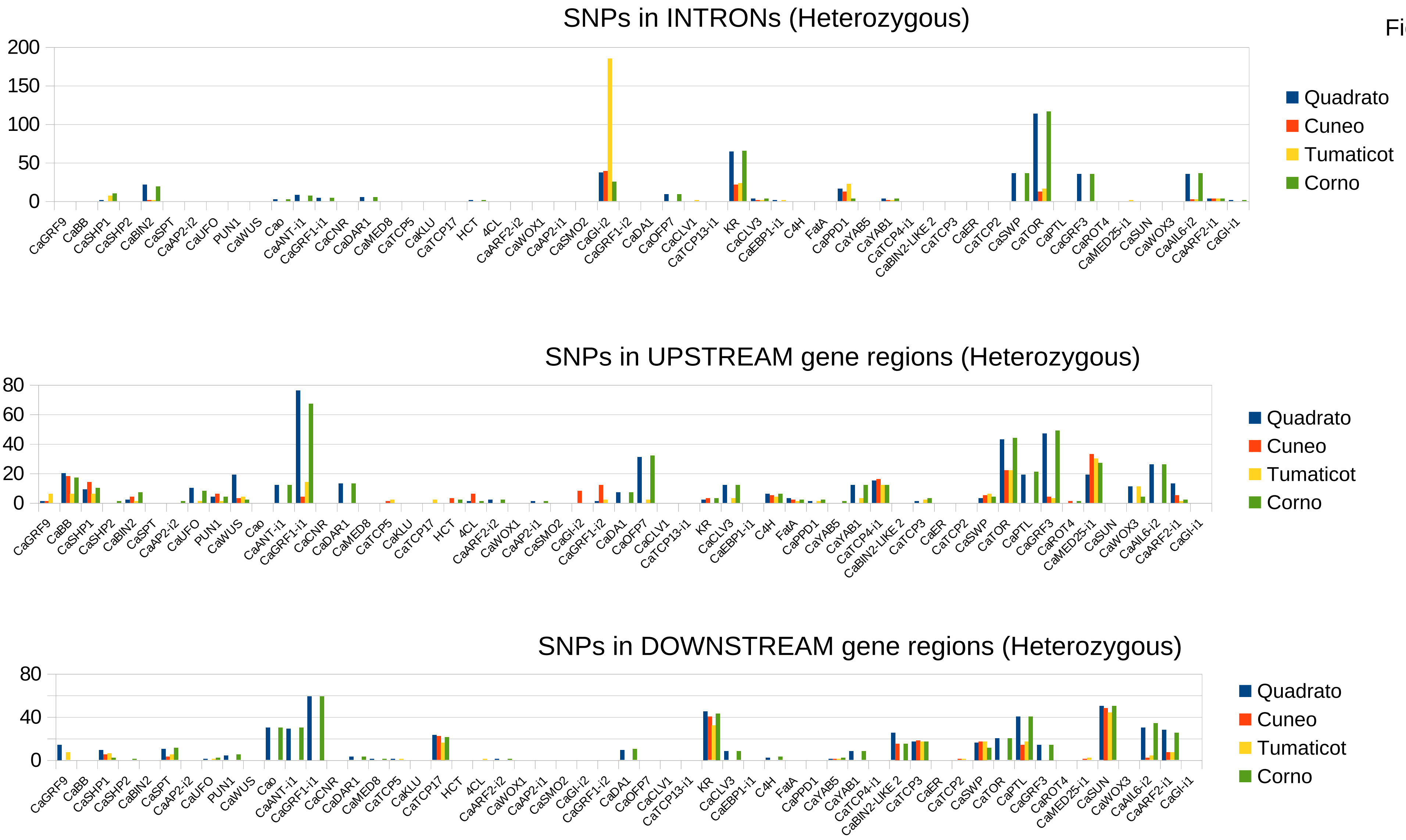
SNPs in INTRONs (Heterozygous)
200
150
100
50
0
CaGRF9 CaBB CaSHP1 CaSHP2 CaBIN2 CaSPT CaAP2-i2 CaUFO PUN1 CaWUS Cao CaANT-i1 CaGRF1-i1 CaCNR CaDAR1 CaMED8 CaTCP5 CaKLU CaTCP17 HCT 4CL CaARF2-i2 CaWOX1 CaAP2-i1 CaSMO2 CaGI-i2 CaGRF1-i2 CaDA1 CaOFP7 CaCLV1 CaTCP13-i1 KR CaCLV3 CaEBP1-i1 C4H FatA CaPPD1 CaYAB5 CaYAB1 CaTCP4-i1 CaBIN2-LIKE 2 CaTCP3 CaER CaTCP2 CaSWP CaTOR CaPTL CaGRF3 CaROT4 CaMED25-i1 CaSUN CaWOX3 CaAIL6-i2 CaARF2-i1 CaGI-i1
Quadrato
Cuneo
Tumaticot
Corno
SNPs in UPSTREAM gene regions (Heterozygous)
80
60
40
20
0
CaGRF9 CaBB CaSHP1 CaSHP2 CaBIN2 CaSPT CaAP2-i2 CaUFO PUN1 CaWUS Cao CaANT-i1 CaGRF1-i1 CaCNR CaDAR1 CaMED8 CaTCP5 CaKLU CaTCP17 HCT 4CL CaARF2-i2 CaWOX1 CaAP2-i1 CaSMO2 CaGI-i2 CaGRF1-i2 CaDA1 CaOFP7 CaCLV1 CaTCP13-i1 KR CaCLV3 CaEBP1-i1 C4H FatA CaPPD1 CaYAB5 CaYAB1 CaTCP4-i1 CaBIN2-LIKE 2 CaTCP3 CaER CaTCP2 CaSWP CaTOR CaPTL CaGRF3 CaROT4 CaMED25-i1 CaSUN CaWOX3 CaAIL6-i2 CaARF2-i1 CaGI-i1
Quadrato
Cuneo
Tumaticot
Corno
SNPs in DOWNSTREAM gene regions (Heterozygous)
80
40
0
CaGRF9 CaBB CaSHP1 CaSHP2 CaBIN2 CaSPT CaAP2-i2 CaUFO PUN1 CaWUS Cao CaANT-i1 CaGRF1-i1 CaCNR CaDAR1 CaMED8 CaTCP5 CaKLU CaTCP17 HCT 4CL CaARF2-i2 CaWOX1 CaAP2-i1 CaSMO2 CaGI-i2 CaGRF1-i2 CaDA1 CaOFP7 CaCLV1 CaTCP13-i1 KR CaCLV3 CaEBP1-i1 C4H FatA CaPPD1 CaYAB5 CaYAB1 CaTCP4-i1 CaBIN2-LIKE 2 CaTCP3 CaER CaTCP2 CaSWP CaTOR CaPTL CaGRF3 CaROT4 CaMED25-i1 CaSUN CaWOX3 CaAIL6-i2 CaARF2-i1 CaGI-i1
Quadrato
Cuneo
Tumaticot
Corno

Figure S3

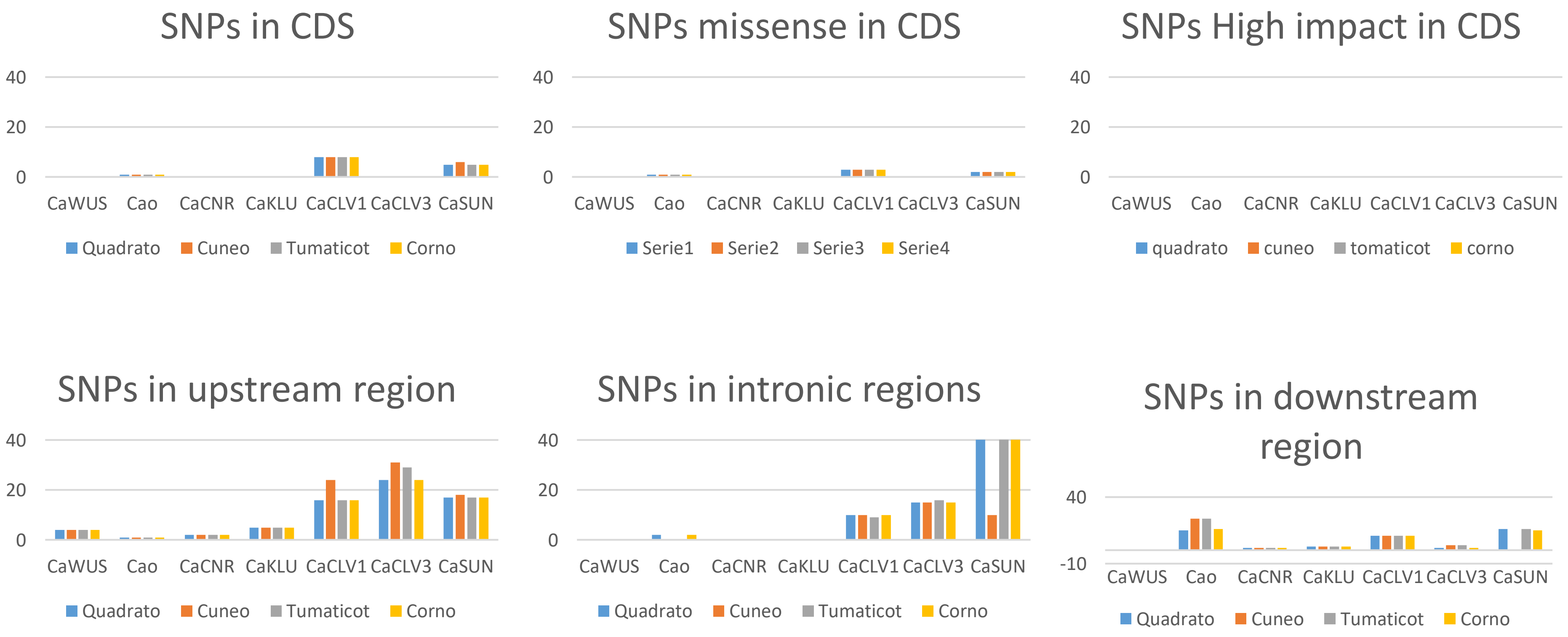
SNPs in CDS
40
20
0
CaWUS Cao CaCNR CaKLU CaCLV1 CaCLV3 CaSUN
Quadrato Cuneo Tumaticot Corno
SNPs missense in CDS
40
20
0
CaWUS Cao CaCNR CaKLU CaCLV1 CaCLV3 CaSUN
Serie1 Serie2 Serie3 Serie4
SNPs High impact in CDS
40
20
0
CaWUS Cao CaCNR CaKLU CaCLV1 CaCLV3 CaSUN
quadrato cuneo tomaticot corno
SNPs in upstream region
40
20
0
CaWUS Cao CaCNR CaKLU CaCLV1 CaCLV3 CaSUN
Quadrato Cuneo Tumaticot Corno
SNPs in intronic regions
40
20
0
CaWUS Cao CaCNR CaKLU CaCLV1 CaCLV3 CaSUN
Quadrato Cuneo Tumaticot Corno
SNPs in downstream region
40
-10
CaWUS Cao CaCNR CaKLU CaCLV1 CaCLV3 CaSUN
Quadrato Cuneo Tumaticot Corno

Figure S4

SUN 3kb upstream

```
                      *        20         *        40         *        60         *        80         *
Perennial_ : TATCAAGAAGGGCATACTGACACGCATATATACTCCTATGGTAAGATCAAATACTATTTGCCAAAGCTCCTTTGAGTTCATTCCGATGGCCATCA :   95
CM334_SUN  : TATCAAGAAGGGCATACTGACACGCATATATACTCCTATGGTAAGATCAAATACTATTTGCCAAAGCTCCTTTGAGTTCATTCCGATGGCCATCA :   95
Quadrato_S : TATCAAGAAGGGCATACTGACACGCATATATACTCCTATGGTAAGATCAAATACTATTTGCCAAAGCTCCTTTGAGTTCATTCCGATGGCCATCA :   95
Tumaticot_ : TATCAAGAAGGGCATACTGACACGCATATATACTCCTATGGTAAGATCAAATACTATTTGCCAAAGCTCCTTTGAGTTCATTCCGATGGCCATCA :   95
Corno_SUN  : TATCAAGAAGGGCATACTGACACGCATATATACTCCTATGGTAAGATCAAATACTATTTGCCAAAGCTCCTTTGAGTTCATTCCGATGGCCATCA :   95
Cuneo_SUN  : GATCAAGAAGGGCATACTGACACGCATATATACTCCTATGGTAAGATCAAATACTATTTGCCAAAGCTCCTTTGAGTTCATTCCGATGGCCATCA :   95
             tATCAAGAAGGGCATACTGACACGCATATATACTCCTATGGTAAGATCAAATACTATTTGCCAAAGCTCCTTTGAGTTCATTCCGATGGCCATCA

               100         *       120         *       140         *       160         *       180         *
Perennial_ : ACCCTCGGATACATAATTACAAGAAGCCGAACTCATAATATAACCTTTTTTCTGCCTTTAGTCCATCATACAGGTTTTGAAGGAATCTGGCGTTT :  190
CM334_SUN  : ACCCTCGGATACATAATTACAAGAAGCCGAACTCATAATATAACCTTTTTTCTGCCTTTAGTCCATCATACAGGTTTTGAAGGAATCTGGCGTTT :  190
Quadrato_S : ACCCTCGGATACATAATTACAAGAAGCCGAACTCATAATATAACCTTTTTTCTGCCTTTAGTCCATCATACAGGTTTTGAAGGAATCTGGCGTTT :  190
Tumaticot_ : ACCCTCGGATACATAATTACAAGAAGCCGAACTCATAATATAACCTTTTTTCTGCCTTTAGTCCATCATACAGGTTTTGAAGGAATCTGGCGTTT :  190
Corno_SUN  : ACCCTCGGATACATAATTACAAGAAGCCGAACTCATAATATAACCTTTTTTCTGCCTTTAGTCCATCATACAGGTTTTGAAGGAATCTGGCGTTT :  190
Cuneo_SUN  : ACCCTCGGATACATAATTACAAGAAGCCGAACTCATAATATAACCTTTTTTCTGCCTTTAGTCCATCATACAGGTTTTGAAGGAATCTGGCGTTT :  190
             ACCCTCGGATACATAATTACAAGAAGCCGAACTCATAATATAACCTTTTTTCTGCCTTTAGTCCATCATACAGGTTTTGAAGGAATCTGGCGTTT

                    200         *       220         *       240         *       260         *       280
Perennial_ : TTGCTTCTGCTATTGCATCATCAAATAGAGATATCATAAAAGAAGAGTCTTTATCTTCTACCGGAAACAGAAACGCTTCTGGATATCCTCATACT :  285
CM334_SUN  : TTGCTTCTGCTATTGCATCATCAAATAGAGATATCATAAAAGAAGAGTCTTTATCTTCTACCGGAAACAGAAACGCTTCTGGATATCCTCATACT :  285
Quadrato_S : TTGCTTCTGCTATTGCATCATCAAATAGAGATATCATAAAAGAAGAGTCTTTATCTTCTACAGGAAACAGAAACGCTTCTGGATATCCTCATACT :  285
Tumaticot_ : TTGCTTCTGCTATTGCATCATCAAATAGAGATATCATAAAAGAAGAGTCTTTATCTTCTACAGGAAACAGAAACGCTTCTGGATATCCTCATACT :  285
Corno_SUN  : TTGCTTCTGCTATTGCATCATCAAATAGAGATATCATAAAAGAAGAGTCTTTATCTTCTACAGGAAACAGAAACGCTTCTGGATATCCTCATACT :  285
Cuneo_SUN  : TTGCTTCTGCTATTGCATCATCAAATAGAGATATCATAAAAGAAGAGTCTTTATCTTCTACCGGAAACAGAAACGCTTCTGGATATCCTCATACT :  285
             TTGCTTCTGCTATTGCATCATCAAATAGAGATATCATAAAAGAAGAGTCTTTATCTTCTAC GGAAACAGAAACGCTTCTGGATATCCTCATACT

                 *       300         *       320         *       340         *       360         *       380
Perennial_ : ACTGATACTTTTCCGAGAAAGATGGGTCGTATACTGGAACCTCATGCAACAAGAAAGAAATTCCTGGAATTTAATGAAACTGATCATCCGGCAAT :  380
CM334_SUN  : ACTGATACTTTTCCGAGAAAGATGGGTCGTATACTGGAACCTCATGCAACAAGAAAGAAATTCCTGGAATTTAATGAAACTGATCATCCGGCAAT :  380
Quadrato_S : ACTGATACTTTTCCGAGAAAGATGGGTCGTATACTGGAACCTCATGCAACAAGAAAGAAATTCCTGGAATTTAATGAAACTGATCATCCGGCAAT :  380
Tumaticot_ : ACTGATACTTTTCCGAGAAAGATGGGTCGTATACTGGAACCTCATGCAACAAGAAAGAAATTCCTGGAATTTAATGAAACTGATCATCCGGCAAT :  380
Corno_SUN  : ACTGATACTTTTCCGAGAAAGATGGGTCGTATACTGGAACCTCATGCAACAAGAAAGAAATTCCTGGAATTTAATGAAACTGATCATCCGGCAAT :  380
Cuneo_SUN  : ACTGATACTTTTCCGAGAAAGATGGGTCGTATACTGGAACCTCATGCAACAAGAAAGAAATTCCTGGAATTTAATGAAACCGATCATCCGGCAAT :  380
             ACTGATACTTTTCCGAGAAAGATGGGTCGTATACTGGAACCTCATGCAACAAGAAAGAAATTCCTGGAATTTAATGAAACtGATCATCCGGCAAT

                      *       400         *       420         *       440         *       460         *
Perennial_ : TCCACCCGAATCACCTCCTGGCATGGATAGAATTCACCTAGACAAAATACCCTCTGAACATTGAAGAAAAACACTGATATATCCTCGGGAAGATG :  475
CM334_SUN  : TCCACCCGAATCACCTCCTGGCATGGATAGAATTCACCTAGACAAAATACCCTCTGAACATTGAAGAAAAACACTGATATATCCTCGGGAAGATG :  475
Quadrato_S : TCCACCCGAATCACCTCCTGGCATGGATAGAATTCACCTAGACAAAATACCCTCTGAACATTGAAGAAAAACACTGATATATCCTCGGGAAGATG :  475
Tumaticot_ : TCCACCCGAATCACCTCCTGGCATGGATAGAATTCACCTAGACAAAATACCCTCTGAACATTGAAGAAAAACACTGATATATCCTCGGGAAGATG :  475
Corno_SUN  : TCCACCCGAATCACCTCCTGGCATGGATAGAATTCACCTAGACAAAATACCCTCTGAACATTGAAGAAAAACACTGATATATCCTCGGGAAGATG :  475
Cuneo_SUN  : TCCACCCGAATCACCTCCTGGCATGGATAGAATTCACCTAGACAAAATACCCTCTGAACATTGAAGAAAAACACTGATATATCCTCGGGAAGATG :  475
             TCCACCCGAATCACCTCCTGGCATGGATAGAATTCACCTAGACAAAATACCCTCTGAACATTGAAGAAAAACACTGATATATCCTCGGGAAGATG

               480         *       500         *       520         *       540         *       560         *
Perennial_ : GCTGTACATGAGTTGTGTCCATACTGGAAATTCGTCTCATAGGATCGCTGAGTATCTAGCCAAGAATTCAGTTTCATATCTTAGATAGAGAAGTA :  570
CM334_SUN  : GCTGTACATGAGTTGTGTCCATACTGGAAATTCGTCTCATAGGATCGCTGAGTATCTAGCCAAGAATTCAGTTTCATATCTTAGATAGAGAAGTA :  570
Quadrato_S : GCTGTACATGAGTTGTGTCCATACTGGAAATTCGTCTCATAGGATCGCTGAGTATCTAGCCAAGAATTCAGTTTCATATCTTAGATAGAGAAGTA :  570
Tumaticot_ : GCTGTACATGAGTTGTGTCCATACTGGAAATTCGTCTCATAGGATCGCTGAGTATCTAGCCAAGAATTCAGTTTCATATCTTAGATAGAGAAGTA :  570
Corno_SUN  : GCTGTACATGAGTTGTGTCCATACTGGAAATTCGTCTCATAGGATCGCTGAGTATCTAGCCAAGAATTCAGTTTCATATCTTAGATAGAGAAGTA :  570
Cuneo_SUN  : GCTGTACATGAGTTGTGTCCATACTGGAAATTCGTCTCATAGGATCGCTGAGTATCTAGCCAAGAATTCAGTTTCATATCTTAGATAGAGAAGTA :  570
             GCTGTACATGAGTTGTGTCCATACTGGAAATTCGTCTCATAGGATCGCTGAGTATCTAGCCAAGAATTCAGTTTCATATCTTAGATAGAGAAGTA

                    580         *       600         *       620         *       640         *       660
Perennial_ : TTCTTATAGATATTGCCTGTTCTTTCTTCTCATTCTTTTTTCTCCATTTTTTCTACCCCAGTGAAAGTTCGATATGTATACATGACAGAATGATT :  665
CM334_SUN  : TTCTTATAGATATTGCCTGTTCTTTCTTCTCATTCTTTTTTCTCCATTTTTTCTACCCCAGTGAAAGTTCGATATGTATACATGACAGAATGATT :  665
Quadrato_S : TTCTTATAGATATTGCCTGTTCTTTCTTCTCATTCTTTTTTCTCCATTTTTTCTACCCCAGTGAAAGTTCGATATGTATACATGACAGAATGATT :  665
Tumaticot_ : TTCTTATAGATATTGCCTGTTCTTTCTTCTCATTCTTTTTTCTCCATTTTTTCTACCCCAGTGAAAGTTCGATATGTATACATGACAGAATGATT :  665
Corno_SUN  : TTCTTATAGATATTGCCTGTTCTTTCTTCTCATTCTTTTTTCTCCATTTTTTCTACCCCAGTGAAAGTTCGATATGTATACATGACAGAATGATT :  665
Cuneo_SUN  : TTCTTATAGATATTGCCTGTTCTTTCTTCTCATTCTTTTTTCTCCATTTTTTCTACCCCAGTGAAAGTTCGATATGTATACATGACAGAATGATT :  665
             TTCTTATAGATATTGCCTGTTCTTTCTTCTCATTCTTTTTTCTCCATTTTTTCTACCCCAGTGAAAGTTCGATATGTATACATGACAGAATGATT

                 *       680         *       700         *       720         *       740         *       760
Perennial_ : TGGAGAATGACAATTTTATTCATGGGGTACATTTATCATTTTTGATGGTACAAGAACAGATCTCTTCAAAGATCTTCTTACTTCTACTCGCGTGC :  760
CM334_SUN  : TGGAGAATGACAATTTTATTCATGGGGTACATTTATCATTTTTGATGGTACAAGAACAGATCTCTTCAAAGATCTTCTTACTTCTACTCGCGTGC :  760
Quadrato_S : TGGAGAATGACAATTTTATTCATGGGGTACATTTATCATTTTTGATGGTACAAGAACAGATCTCTTCAAAGATCTTCTTACTTCTACTCGCGTGC :  760
Tumaticot_ : TGGAGAATGACAATTTTATTCATGGGGTACATTTATCATTTTTGATGGTACAAGAACAGATCTCTTCAAAGATCTTCTTACTTCTACTCGCGTGC :  760
Corno_SUN  : TGGAGAATGACAATTTTATTCATGGGGTACATTTATCATTTTTGATGGTACAAGAACAGATCTCTTCAAAGATCTTCTTACTTCTACTCGCGTGC :  760
Cuneo_SUN  : TGGAGAATGACAATTTTATTCATGGGGTACATTTATCATTTTTGATGGTACAAGAACAGATCTCTTCAAAGATCTTCTTACTTCTACTCGCGTGC :  760
             TGGAGAATGACAATTTTATTCATGGGGTACATTTATCATTTTTGATGGTACAAGAACAGATCTCTTCAAAGATCTTCTTACTTCTACTCGCGTGC

                      *       780         *       800         *       820         *       840         *
Perennial_ : GTCTGTCCGCGATTGAGCTGTTTTTTACTTTATTCAGTATGGAACGACTATATTCGATTCTAAATTTTAGAACTGAATATGATTCTTAGATTGTT :  855
CM334_SUN  : GTCTGTCCGCGATTGAGCTGTTTTTTACTTTATTCAGTATGGAACGACTATATTCGATTCTAAATTTTAGAACTGAATATGATTCTTAGATTGTT :  855
Quadrato_S : GTCTGTCCGTGATTGAGCTGTTTTTTACTTTATTCAGTATGGAACGACTATATTCGATTCTAAATTTTAGAACTGAATATGATTCTTAGATTGTT :  855
Tumaticot_ : GTCTGTCCGTGATTGAGCTGTTTTTTACTTTATTCAGTATGGAACGACTATATTCGATTCTAAATTTTAGAACTGAATATGATTCTTAGATTGTT :  855
Corno_SUN  : GTCTGTCCGTGATTGAGCTGTTTTTTACTTTATTCAGTATGGAACGACTATATTCGATTCTAAATTTTAGAACTGAATATGATTCTTAGATTGTT :  855
Cuneo_SUN  : GTCTGTCCGCGATTGAGCTGTTTTTTACTTTATTCAGTATGGAACGACTATATTCGATTCTAAATTTTAGAACTGAATATGATTCTTAGATTGTT :  855
             GTCTGTCCG GATTGAGCTGTTTTTTACTTTATTCAGTATGGAACGACTATATTCGATTCTAAATTTTAGAACTGAATATGATTCTTAGATTGTT

               860         *       880         *       900         *       920         *       940         *
Perennial_ : GTTAAGTACTTTGTTTCACATCCAGTTGAAGGCCAGTATTTCTGGTCTATGTTGCTCGGACTCTTCAAAAATGTCAATGGGTGTGTGTCGGATTC :  950
CM334_SUN  : GTTAAGTACTTTGTTTCACATCCAGTTGAAGGCCAGTATTTCTGGTCTATGTTGCTCGGACTCTTCAAAAATGTCAATGGGTGTGTGTCGGATTC :  950
Quadrato_S : GTTAAGTACTTTGTTTCACATCCAGTTGAAGGCCAGTATTTCTGGTCTATGTTGCTCGGACTCTTCAAAAATGTCAATGGATGTGTGTCGGATTC :  950
Tumaticot_ : GTTAAGTACTTTGTTTCACATCCAGTTGAAGGCCAGTATTTCTGGTCTATGTTGCTCGGACTCTTCAAAAATGTCAATGGATGTGTGTCGGATTC :  950
Corno_SUN  : GTTAAGTACTTTGTTTCACATCCAGTTGAAGGCCAGTATTTCTGGTCTATGTTGCTCGGACTCTTCAAAAATGTCAATGGATGTGTGTCGGATTC :  950
Cuneo_SUN  : GTTAAGTACTTTGTTTCACATCCAGTTGAAGGCCAGTATTTCTGGTCTATGTTGCTCGGACTCTTCAAAAATGTCAATGGGTGTGTGTCGGATTC :  950
             GTTAAGTACTTTGTTTCACATCCAGTTGAAGGCCAGTATTTCTGGTCTATGTTGCTCGGACTCTTCAAAAATGTCAATGG TGTGTGTCGGATTC

                    960         *       980         *      1000         *      1020         *      1040
Perennial_ : GCCAAAACTAGTGTGTTTTTGAAGAATCCGATACGGGTGCGGCATCAAAAGTGAAGAGTCCGCGCAACTTAGGTTCTGGTTCAGAAACAAGATCC : 1045
CM334_SUN  : GCCAAAACTAGTGTGTTTTTGAAGAATCCGATACGGGTGCGGCATCAAAAGTGAAGAGTCCGCGCAACTTAGGTTCTGGTTCAGAAACAAGATCC : 1045
Quadrato_S : GCCAAAACTAGTGTGTTTTTGAAGAATCCGATACGGGTGCGGCATCAAAAGTGAAGAGTCCGCGCAACTTAGGTTCTGGTTCAGAAACAAGATCC : 1045
Tumaticot_ : GCCAAAACTAGTGTGTTTTTGAAGAATCCGATACGGGTGCGGCATCAAAAGTGAAGAGTCCGCGCAACTTAGGTTCTGGTTCAGAAACAAGATCC : 1045
Corno_SUN  : GCCAAAACTAGTGTGTTTTTGAAGAATCCGATACGGGTGCGGCATCAAAAGTGAAGAGTCCGCGCAACTTAGGTTCTGGTTCAGAAACAAGATCC : 1045
Cuneo_SUN  : GCCAAAACTAGTGTGTTTTTGAAGAATCCGATACGGGTGCGGCATCAAAAGTGAAGAGTCCGCGCAACTTAGGTTCTGGTTCAGAAACAAGATCC : 1045
             GCCAAAACTAGTGTGTTTTTGAAGAATCCGATACGGGTGCGGCATCAAAAGTGAAGAGTCCGCGCAACTTAGGTTCTGGTTCAGAAACAAGATCC
```

```
                 *      1060         *      1080         *      1100         *      1120         *      1140
Perennial_ : AACACCTTGTGCACAAAGGGTAATGTTACATTTGATTAGCTTTCTGGCCTAGACTTTGCAACTTCTCTGTTGTAACGCCTATGTGCTATGTTTCG : 1140
CM334_SUN  : AACACCTTGTGCACAAAGGGTAATGTTACATTTGATTAGCTTTCTGGCCTAGACTTTGCAACTTCTCTGTTGTAACGCCTATGTGCTATGTTTCG : 1140
Quadrato_S : AACACCTTGTGCACAAAGGGTAATGTTACATTTGATTAGCTTTCTGGCCTAGACTTTGCAACTTCTCTGTTGTAACGCCTATGTGCTATGTTTCG : 1140
Tumaticot_ : AACACCTTGTGCACAAAGGGTAATGTTACATTTGATTAGCTTTCTGGCCTAGACTTTGCAACTTCTCTGTTGTAACGCCTATGTGCTATGTTTCG : 1140
Corno_SUN  : AACACCTTGTGCACAAAGGGTAATGTTACATTTGATTAGCTTTCTGGCCTAGACTTTGCAACTTCTCTGTTGTAACGCCTATGTGCTATGTTTCG : 1140
Cuneo_SUN  : AACACCTTGTGCACAAAGGGTAATGTTACATTTGATTAGCTTTCTGGCCTAGACTTTGCAACTTCTCTGTTGTAACGCCTATGTGCTATGTTTCG : 1140
             AACACCTTGTGCACAAAGGGTAATGTTACATTTGATTAGCTTTCTGGCCTAGACTTTGCAACTTCTCTGTTGTAACGCCTATGTGCTATGTTTCG

                      *      1160         *      1180         *      1200         *      1220         *
Perennial_ : CCGATCCAAAGATACACTACTTTTGGCCGAGGCCGATCAAACACGCATCTGACATCTTTTAGAGAGTCCGAACAACATAACCTATGTGTACTCAT : 1235
CM334_SUN  : CCGATCCAAAGATACACTACTTTTGGCCGAGGCCGATCAAACACGCATCTGACATCTTTTAGAGAGTCCGAACAACATAACCTATGTGTACTCAT : 1235
Quadrato_S : CCGATCCAAAGATACACTACTTTTGGCCGAGGCCGATCAAACACGCATCTGACATCTTTTAGAGAGTCCGAACAACATAACCTATGTGTACTCAT : 1235
Tumaticot_ : CCGATCCAAAGATACACTACTTTTGGCCGAGGCCGATCAAACACGCATCTGACATCTTTTAGAGAGTCCGAACAACATAACCTATGTGTACTCAT : 1235
Corno_SUN  : CCGATCCAAAGATACACTACTTTTGGCCGAGGCCGATCAAACACGCATCTGACATCTTTTAGAGAGTCCGAACAACATAACCTATGTGTACTCAT : 1235
Cuneo_SUN  : CCGATCCAAAGATACACTACTTTTGGCCGAGGCCGATCAAACACGCATCTGACATCTTTTAGAGAGTCCGAACAACATAACCTATGTGTACTCAT : 1235
             CCGATCCAAAGATACACTACTTTTGGCCGAGGCCGATCAAACACGCATCTGACATCTTTTAGAGAGTCCGAACAACATAACCTATGTGTACTCAT

              1240         *      1260         *      1280         *      1300         *      1320         *
Perennial_ : GAGAGTTGGGGGTATGCAACTTGCAAACACATATTTTTGATTTGGTAGACTACACATCATATGGGATTGTCTATGACACAATCTATTTTTATAGC : 1330
CM334_SUN  : GAGAGTTGGGGGTATGCAACTTGCAAACACATATTTTTGATTTGGTAGACTACACATCATATGGGATTGTCTATGACACAATCTATTTTTATAGC : 1330
Quadrato_S : GAGAGTTGGGGGTATGCAACTTGCAAACACATATTTTTGATTTGGTAGACTACACATCATATGGGATTGTCTATGACACAATCTATTTTTATAGC : 1330
Tumaticot_ : GAGAGTTGGGGGTATGCAACTTGCAAACACATATTTTTGATTTGGTAGACTACACATCATATGGGATTGTCTATGACACAATCTATTTTTATAGC : 1330
Corno_SUN  : GAGAGTTGGGGGTATGCAACTTGCAAACACATATTTTTGATTTGGTAGACTACACATCATATGGGATTGTCTATGACACAATCTATTTTTATAGC : 1330
Cuneo_SUN  : GAGAGTTGGGGGTATGCAACTTGCAAACACATATTTTTGATTTGGTAGACTACACATCATATGGGATTGTCTATGACACAATCTATTTTTATAGC : 1330
             GAGAGTTGGGGGTATGCAACTTGCAAACACATATTTTTGATTTGGTAGACTACACATCATATGGGATTGTCTATGACACAATCTATTTTTATAGC

                   1340         *      1360         *      1380         *      1400         *      1420
Perennial_ : TCGAACATGTGAGTACGCATCACATGAATTGTCTATGACACATACATCAACAGTTGATTTTTACGGCTTGAATTCGTGACATACACCACATTGAT : 1425
CM334_SUN  : TCGAACATGTGAGTACGCATCACATGAATTGTCTATGACACATACATCAACAGTTGATTTTTACGGCTTGAATTCGTGACATACACCACATTGAT : 1425
Quadrato_S : TCGAACATGTGAGTACGCATCACATGAATTGTCTATGACACATACATCAACAGTTGATTTTTACGGCTTGAATTCGTGACATACACCACATTGAT : 1425
Tumaticot_ : TCGAACATGTGAGTACGCATCACATGAATTGTCTATGACACATACATCAACAGTTGATTTTTACGGCTTGAATTCGTGACATACACCACATTGAT : 1425
Corno_SUN  : TCGAACATGTGAGTACGCATCACATGAATTGTCTATGACACATACATCAACAGTTGATTTTTACGGCTTGAATTCGTGACATACACCACATTGAT : 1425
Cuneo_SUN  : TCGAACATGTGAGTACGCATCACATGAATTGTCTATGACACATACATCAACAGTTGATTTTTACGGCTTGAATTCGTGACATACACCACATTGAT : 1425
             TCGAACATGTGAGTACGCATCACATGAATTGTCTATGACACATACATCAACAGTTGATTTTTACGGCTTGAATTCGTGACATACACCACATTGAT

                 *      1440         *      1460         *      1480         *      1500         *      1520
Perennial_ : ATTCTCCAAAGCTCCCTTCTTTTTCTTTTTCTATTATATATTGATTAAAATGTAATTCTTAATTTCTGGTAGTAATATTTTTGCTGGACCACTTA : 1520
CM334_SUN  : ATTCTCCAAAGCTCCCTTCTTTTTCTTTTTCTATTATATATTGATTAAAATGTAATTCTTAATTTCTGGTAGTAATATTTTTGCTGGACCACTTA : 1520
Quadrato_S : ATTCTCCAAAGCTCCCTTCTTTTTCTTTTTCTATTATATATTGATTAAAATGTAATTCTTAATTTCTGGTAGTAATATTTTTGCTGGACCACTTA : 1520
Tumaticot_ : ATTCTCCAAAGCTCCCTTCTTTTTCTTTTTCTATTATATATTGATTAAAATGTAATTCTTAATTTCTGGTAGTAATATTTTTGCTGGACCACTTA : 1520
Corno_SUN  : ATTCTCCAAAGCTCCCTTCTTTTTCTTTTTCTATTATATATTGATTAAAATGTAATTCTTAATTTCTGGTAGTAATATTTTTGCTGGACCACTTA : 1520
Cuneo_SUN  : ATTCTCCAAAGCTCCCTTCTTTTTCTTTTTCTATTATATATTGATTAAAATGTAATTCTTAATTTCTGGTAGTAATATTTTTGCTGGACCACTTA : 1520
             ATTCTCCAAAGCTCCCTTCTTTTTCTTTTTCTATTATATATTGATTAAAATGTAATTCTTAATTTCTGGTAGTAATATTTTTGCTGGACCACTTA

                      *      1540         *      1560         *      1580         *      1600         *
Perennial_ : ATTTTGTATGAGAGGCAACATATAGTGCTTTGCCTGTAACACAATATTGTAATATAATTGGACTTGCTCTAAATTATATTGTTAAATCAGACCAA : 1615
CM334_SUN  : ATTTTGTATGAGAGGCAACATATAGTGCTTTGCCTGTAACACAATATTGTAATATAATTGGACTTGCTCTAAATTATATTGTTAAATCAGACCAA : 1615
Quadrato_S : ATTTTGTATGAGAGGCAACATATAGTGCTTTGCCTGTAACACAATATTGTAATATAATTGGACTTGCTCTAAATTATATTGTTAAATCAGACCAA : 1615
Tumaticot_ : ATTTTGTATGAGAGGCAACATATAGTGCTTTGCCTGTAACACAATATTGTAATATAATTGGACTTGCTCTAAATTATATTGTTAAATCAGACCAA : 1615
Corno_SUN  : ATTTTGTATGAGAGGCAACATATAGTGCTTTGCCTGTAACACAATATTGTAATATAATTGGACTTGCTCTAAATTATATTGTTAAATCAGACCAA : 1615
Cuneo_SUN  : ATTTTGTATGAGAGGCAACATATAGTGCTTTGCCTGTAACACAATATTGTAATATAATTGGACTTGCTCTAAATTATATTGTTAAATCAGACCAA : 1615
             ATTTTGTATGAGAGGCAACATATAGTGCTTTGCCTGTAACACAATATTGTAATATAATTGGACTTGCTCTAAATTATATTGTTAAATCAGACCAA

              1620         *      1640         *      1660         *      1680         *      1700         *
Perennial_ : GTAAAACAAATAGGTAATGTCCTACATCATTTTCCCCTATTTTTTAAGTATAAAATCAGCATAACCTATTGAAAATTCTGGTTTGCTTCATGTGA : 1710
CM334_SUN  : GTAAAACAAATAGGTAATGTCCTACATCATTTTCCCCTATTTTTTAAGTATAAAATCAGCATAACCTATTGAAAATTCTGGTTTGCTTCATGTGA : 1710
Quadrato_S : GTAAAACAAATAGGTAATGTCCTACATCATTTTCCCCTATTTTTTAAGTATAAAATCAGCATATCCTATTGAAAATTCTGGTTTGCTTCATGTGA : 1710
Tumaticot_ : GTAAAACAAATAGGTAATGTCCTACATCATTTTCCCCTATTTTTTAAGTATAAAATCAGCATATCCTATTGAAAATTCTGGTTTGCTTCATGTGA : 1710
Corno_SUN  : GTAAAACAAATAGGTAATGTCCTACATCATTTTCCCCTATTTTTTAAGTATAAAATCAGCATATCCTATTGAAAATTCTGGTTTGCTTCATGTGA : 1710
Cuneo_SUN  : GTAAAACAAATAGGTAATGTCCTACATCATTTTCCCCTATTTTTTAAGTATAAAATCAGCATATCCTATTGAAAATTCTGGTTTGCTTCATGTGA : 1710
             GTAAAACAAATAGGTAATGTCCTACATCATTTTCCCCTATTTTTTAAGTATAAAATCAGCATA CCTATTGAAAATTCTGGTTTGCTTCATGTGA

                   1720         *      1740         *      1760         *      1780         *      1800
Perennial_ : ACATTGTCCATTTTTTT-ATTAATTATCAATAGAACATTGACATAAAAAAGATATTATATTGTAAAATCAATCAGAGGAATTTTGTGTGAATCAG : 1804
CM334_SUN  : ACATTGTCCATTTTTTT-ATTAATTATCAATAGAACATTGACATAAAAAAGATATTATATTGTAAAATCAATCAGAGGAATTTTGTGTGAATCAG : 1804
Quadrato_S : ACATTGTCCATTTTTTT-ATTAATTATCAATAGAACATTGACATAAAAAAGATATTATATTGTAAAATCAATCAGAGGAATTTTGTGTGAATCAG : 1804
Tumaticot_ : ACATTGTCCATTTTTTT-ATTAATTATCAATAGAACATTGACATAAAAAAGATATTATATTGTAAAATCAATCAGAGGAATTTTGTGTGAATCAG : 1804
Corno_SUN  : ACATTGTCCATTTTTTT-ATTAATTATCAATAGAACATTGACATAAAAAAGATATTATATTGTAAAATCAATCAGAGGAATTTTGTGTGAATCAG : 1804
Cuneo_SUN  : ACATTGTCCATTTTTTTTATTAATTATCAATAGAACATTGACATAAAAAAGATATTATATTGTAAAATCAATCAGAGGAATTTTGTGTGAATCAG : 1805
             ACATTGTCCATTTTTTT ATTAATTATCAATAGAACATTGACATAAAAAAGATATTATATTGTAAAATCAATCAGAGGAATTTTGTGTGAATCAG

                 *      1820         *      1840         *      1860         *      1880         *      1900
Perennial_ : GTTTCTACTATTAGCATAAAAAAATAATTGATTAAAGGAAATCATTCTAAATTGGCAAGTAGTTCATGGTTTAAATAATCAAATTTCATGATTAAG : 1899
CM334_SUN  : GTTTCTACTATTAGCATAAAAAAATAATTGATTAAAGGAAATCATTCTAAATTGGCAAGTAGTTCATGGTTTAAATAATCAAATTTCATGATTAAG : 1899
Quadrato_S : GTTTCTACTATTAGCATAAAAAAATAATTGATTAAAGGAAATCATTCTAAATTGGCAAGTAGTTCATGGTTTAAATAATCAAATTTCATGATTAAG : 1899
Tumaticot_ : GTTTCTACTATTAGCATAAAAAAATAATTGATTAAAGGAAATCATTCTAAATTGGCAAGTAGTTCATGGTTTAAATAATCAAATTTCATGATTAAG : 1899
Corno_SUN  : GTTTCTACTATTAGCATAAAAAAATAATTGATTAAAGGAAATCATTCTAAATTGGCAAGTAGTTCATGGTTTAAATAATCAAATTTCATGATTAAG : 1899
Cuneo_SUN  : GTTTCTACTATTAGCATAAAAAAATAATTGATTAAAGGAAATCATTCTAAATTGGCAAGTAGTTCATGGTTTAAATAATCAAATTTCATGATTAAG : 1900
             GTTTCTACTATTAGCATAAAAAAATAATTGATTAAAGGAAATCATTCTAAATTGGCAAGTAGTTCATGGTTTAAATAATCAAATTTCATGATTAAG

                      *      1920         *      1940         *      1960         *      1980         *
Perennial_ : ACTAGAGGAAGTTATAATCCTACATGAGATAGGGACTCCATATATAAACAATTGACTGGAGAAAGTTATAAAGGAACATTAGAATTGATAGCAAT : 1994
CM334_SUN  : ACTAGAGGAAGTTATAATCCTACATGAGATAGGGACTCCATATATAAACAATTGACTGGAGAAAGTTATAAAGGAACATTAGAATTGATAGCAAT : 1994
Quadrato_S : ACTAGAGGAAGTTATAATCCTACATGAGATAGGGACTCCGTATATAAACAATTGACTGGAGAAAGTTATAAAGGAACATTAGAATTGATAGCAAT : 1994
Tumaticot_ : ACTAGAGGAAGTTATAATCCTACATGAGATAGGGACTCCGTATATAAACAATTGACTGGAGAAAGTTATAAAGGAACATTAGAATTGATAGCAAT : 1994
Corno_SUN  : ACTAGAGGAAGTTATAATCCTACATGAGATAGGGACTCCGTATATAAACAATTGACTGGAGAAAGTTATAAAGGAACATTAGAATTGATAGCAAT : 1994
Cuneo_SUN  : ACTAGAGGAAGTTATAATCCTACATGAGATAGGGACTCCGTATATAAACAATTGACTGGAGAAAGTTATGAAGGAACATTAGAATTGATAGCAAT : 1995
             ACTAGAGGAAGTTATAATCCTACATGAGATAGGGACTCC TATATAAACAATTGACTGGAGAAAGTTATaAAGGAACATTAGAATTGATAGCAAT

              2000         *      2020         *      2040         *      2060         *      2080         *
Perennial_ : AAGAGTAGTTTGGGATGGATAGGACCAGTGGTCTTGGATTCGAGTTCTACATACGGAAAAACTCTTGTTATAGGGGGATTTCCTTCTTAAATAGG : 2089
CM334_SUN  : AAGAGTAGTTTGGGATGGATAGGACCAGTGGTCTTGGATTCGAGTTCTACATACGGAAAAACTCTTGTTATAGGGGGATTTCCTTCTTAAATAGG : 2089
Quadrato_S : AAGAGTAGTTTGGGATGGATAGGACCAGTGGTCTTGGATTCGAGTTCTACATACGGAAAAACTCTTGTTATAGAGGGATTTCCTTCTTAAATAGG : 2089
Tumaticot_ : AAGAGTAGTTTGGGATGGATAGGACCAGTGGTCTTGGATTCGAGTTCTACATACGGAAAAACTCTTGTTATAGAGGGATTTCCTTCTTAAATAGG : 2089
Corno_SUN  : AAGAGTAGTTTGGGATGGATAGGACCAGTGGTCTTGGATTCGAGTTCTACATACGGAAAAACTCTTGTTATAGAGGGATTTCCTTCTTAAATAGG : 2089
Cuneo_SUN  : AAGAGTAGTTTGGGATGGATAGGACCAGTGGTCTTGGATTCGAGTTCTACATACGGAAAAACTCTTGTTATAGAGGGATTTCCTTCTTAAATAGG : 2090
             AAGAGTAGTTTGGGATGGATAGGACCAGTGGTCTTGGATTCGAGTTCTACATACGGAAAAACTCTTGTTATAG GGGATTTCCTTCTTAAATAGG
```

```
                    2100         *       2120         *       2140         *       2160         *       2180
Perennial_  : CCTTACACAACGCGAATCTGTATTACTCGCGATTTCCCCCTTAAATAAGCCTTATACATCGGGAATCTGTATTAGTCGCTCTCGAATGAAGATGT : 2184
CM334_SUN   : CCTTACACAACGCGAATCTGTATTACTCGCGATTTCCCCCTTAAATAAGCCTTATACATCGGGAATCTGTATTAGTCGCTCTCGAATGAAGATGT : 2184
Quadrato_S  : CCTTACACAACGCGAATCTGTATTACTCGCGATTTCCCCCTTAAATAAGCCTTATACATCGGGAATCTGTATTAGTCGCTCTCGAATGAAGATGT : 2184
Tumaticot_  : CCTTACACAACGCGAATCTGTATTACTCGCGATTTCCCCCTTAAATAAGCCTTATACATCGGGAATCTGTATTAGTCGCTCTCGAATGAAGATGT : 2184
Corno_SUN   : CCTTACACAACGCGAATCTGTATTACTCGCGATTTCCCCCTTAAATAAGCCTTATACATCGGGAATCTGTATTAGTCGCTCTCGAATGAAGATGT : 2184
Cuneo_SUN   : CCTTACACAACGCGAATCTGTATTACTCGCGATTTCCCCCTTAAATAAGCCTTATACATCGGGAATCTGTATTAGTCGCTCTCGAATGAAGATGT : 2185
              CCTTACACAACGCGAATCTGTATTACTCGCGATTTCCCCCTTAAATAAGCCTTATACATCGGGAATCTGTATTAGTCGCTCTCGAATGAAGATGT

                  *       2200         *       2220         *       2240         *       2260         *       2280
Perennial_  : GGAAAACCATTGACTGACAACTACTAAATCTTCTTCAATATTCTTGTGAATTAAAAAACAACTACATGGTAGGCTACTTTGTAGTTTGACATTCT : 2279
CM334_SUN   : GGAAAACCATTGACTGACAACTACTAAATCTTCTTCAATATTCTTGTGAATTAAAAAACAACTACATGGTAGGCTACTTTGTAGTTTGACATTCT : 2279
Quadrato_S  : GGAAAGCCATTGACTGACAACTACTAAATCTTCTTCAATATTCTTGTGAATTAAAAAACAACTACATGGTAGGCTACTTTGTAGTTTGACATTCT : 2279
Tumaticot_  : GGAAAGCCATTGACTGACAACTACTAAATCTTCTTCAATATTCTTGTGAATTAAAAAACAACTACATGGTAGGCTACTTTGTAGTTTGACATTCT : 2279
Corno_SUN   : GGAAAGCCATTGACTGACAACTACTAAATCTTCTTCAATATTCTTGTGAATTAAAAAACAACTACATGGTAGGCTACTTTGTAGTTTGACATTCT : 2279
Cuneo_SUN   : GGAAAACCATTGACTGACAACTACTAAATCTTCTTCAATATTCTTGTGAATTAAAAAACAACTACATGGTAGGCTACTTTGTAGTTTGACATTCT : 2280
              GGAAA CCATTGACTGACAACTACTAAATCTTCTTCAATATTCTTGTGAATTAAAAAACAACTACATGGTAGGCTACTTTGTAGTTTGACATTCT

                       *       2300         *       2320         *       2340         *       2360         *
Perennial_  : TTAAGTCCCTAGACTTTTTCAAGTTCCTAAACTTGTTTGCCATTGTTTTTTTTAATAATCGTGGTGTTCGAGCCAACTCGTGCACCTCGACTAAT : 2374
CM334_SUN   : TTAAGTCCCTAGACTTTTTCAAGTTCCTAAACTTGTTTGCCATTGTTTTTTTTAATAATCGTGGTGTTCGAGCCAACTCGTGCACCTCGACTAAT : 2374
Quadrato_S  : TTAAGTCCCTAGACTTTTTCAAGTTCCTAAACTTGTTTGCCATTGTTTTTTTTAATAATCGTGGTGTTCGAGCCAACTCGTGCACCTCGACTAAT : 2374
Tumaticot_  : TTAAGTCCCTAGACTTTTTCAAGTTCCTAAACTTGTTTGCCATTGTTTTTTTTAATAATCGTGGTGTTCGAGCCAACTCGTGCACCTCGACTAAT : 2374
Corno_SUN   : TTAAGTCCCTAGACTTTTTCAAGTTCCTAAACTTGTTTGCCATTGTTTTTTTTAATAATCGTGGTGTTCGAGCCAACTCGTGCACCTCGACTAAT : 2374
Cuneo_SUN   : TTAAGTCCCTAGACTTTTTCAAGTTCCTAAACTTGTTTGCCATTGTTTTTTTTAATAATCGTGGTGATCGAGCCAACTCGTGCACCTCGACTAAT : 2375
              TTAAGTCCCTAGACTTTTTCAAGTTCCTAAACTTGTTTGCCATTGTTTTTTTTAATAATCGTGGTGtTCGAGCCAACTCGTGCACCTCGACTAAT

               2380         *       2400         *       2420         *       2440         *       2460         *
Perennial_  : TCCACGGGATATTTGCCACCTCCAACCAGAAACAAATACCAGGTAATGTGCCTTTTTCTTAATAACCGTTATGTCTAGGCTAGCTTGTGCACCTC : 2469
CM334_SUN   : TCCACGGGATATTTGCCACCTCCAACCAGAAACAAATACCAGGTAATGTGCCTTTTTCTTAATAACCGTTATGTCTAGGCTAGCTTGTGCACCTC : 2469
Quadrato_S  : TCCACGGGATATTTGCCACCTCCAACCAGAAACAAATACCAGGTAATGTGCCTTTTTCTTAATAACCGTTATGTCTAGGCCAGCTTGTGCACCTC : 2469
Tumaticot_  : TCCACGGGATATTTGCCACCTCCAACCAGAAACAAATACCAGGTAATGTGCCTTTTTCTTAATAACCGTTATGTCTAGGCCAGCTTGTGCACCTC : 2469
Corno_SUN   : TCCACGGGATATTTGCCACCTCCAACCAGAAACAAATACCAGGTAATGTGCCTTTTTCTTAATAACCGTTATGTCTAGGCCAGCTTGTGCACCTC : 2469
Cuneo_SUN   : TCCACAGGATATTTGCCACCTCCAACCAGAAACAAATACCAGGTAATGTGCCTTTTTCTTAATATCCGTTATGTCTAGGCTAGCTTGTGCACCTC : 2470
              TCCACgGGATATTTGCCACCTCCAACCAGAAACAAATACCAGGTAATGTGCCTTTTTCTTAATAaCCGTTATGTCTAGGC AGCTTGTGCACCTC

                    2480         *       2500         *       2520         *       2540         *       2560
Perennial_  : GACTAATTCCGATGGATACCTGCTATCTCCCACCAGCAACAAGTACCCCTCCTACCAGTTGCTTTAATAACTATTATGTCTGGGTCAGCTTGTGC : 2564
CM334_SUN   : GACTAATTCCGATGGATACCTGCTATCTCCCACCAGCAACAAGTACCCCTCCTACCAGTTGCTTTAATAACTATTATGTCTGGGTCAGCTTGTGC : 2564
Quadrato_S  : GACTAATTCCGATGGATACCTGCTATCTCCCACCAGCAACAAGTACCCCTCCTACCAGTTGCTTTAATAACTGTTATGTCTGGGTCAGCTTGTGC : 2564
Tumaticot_  : GACTAATTCCGATGGATACCTGCTATCTCCCACCAGCAACAAGTACCCCTCCTACCAGTTGCTTTAATAACTGTTATGTCTGGGTCAGCTTGTGC : 2564
Corno_SUN   : GACTAATTCCGATGGATACCTGCTATCTCCCACCAGCAACAAGTACCCCTCCTACCAGTTGCTTTAATAACTGTTATGTCTGGGTCAGCTTGTGC : 2564
Cuneo_SUN   : GACTAATTCCGATGGATACCTGCTATCTCCCACCAGCAACAAGTACCCCTCCTACCAGTTGCTTTAATAACTGTTATGTCTGGGTCAGCTTGTGC : 2565
              GACTAATTCCGATGGATACCTGCTATCTCCCACCAGCAACAAGTACCCCTCCTACCAGTTGCTTTAATAACT TTATGTCTGGGTCAGCTTGTGC

                  *       2580         *       2600         *       2620         *       2640         *       2660
Perennial_  : ACCTTGACTAATTTCAATGGATACCTGCCATCTCCCACCAGCAACAGGTATCAAGTAATGTGCCCTTTTTTTAATAACCGTTGTGTCTGGTCAG : 2659
CM334_SUN   : ACCTTGACTAATTTCAATGGATACCTGCCATCTCCCACCAGCAACAGGTATCAAGTAATGTGCCCTTTTTTTAATAACCGTTGTGTCTGGTCAG : 2659
Quadrato_S  : ACCTTGACTAATTTCAATGGATACCTGCCATCTCCCACCAGCAACAGGTATCAAGTAATGTGCCCTTTTTGTAATAACCGTTGTGTCTGGGCAG : 2659
Tumaticot_  : ACCTTGACTAATTTCAATGGATACCTGCCATCTCCCACCAGCAACAGGTATCAAGTAATGTGCCCTTTTTGTAATAACCGTTGTGTCTGGGCAG : 2659
Corno_SUN   : ACCTTGACTAATTTCAATGGATACCTGCCATCTCCCACCAGCAACAGGTATCAAGTAATGTGCCCTTTTTGTAATAACCGTTGTGTCTGGGCAG : 2659
Cuneo_SUN   : ACCTTGACTAATTTCAATGGATACCTGCCATCTCCCACCAGCAACAGGTATCAAGTAATGTGCCCTTTTTTTAATAACCGTTGTGTCTGGTCAA : 2660
              ACCTTGACTAATTTCAATGGATACCTGCCATCTCCCACCAGCAACAGGTATCAAGTAATGTGCCCTTTTT TAATAACCGTTGTGTCTGGG CAg

                       *       2680         *       2700         *       2720         *       2740         *
Perennial_  : CTTGTGCACCTCGACTAATTCCGATGGATACTTGCCATTTCGCACCAGCAACAGGTAGCTTGTGCACCTCGACTAATTCCAATGAATACCTGCCA : 2754
CM334_SUN   : CTTGTGCACCTCGACTAATTCCGATGGATACTTGCCATTTCGCACCAGCAACAGGTAGCTTGTGCACCTCGACTAATTCCAATGAATACCTGCCA : 2754
Quadrato_S  : CTTGTGCACCTCGACTAATTCCGATGGATACTTGCCATTTCGCACCAGCAACAGGTAGCTTGTGCACCTCGACTAATTCCAATGAATACCTGCCA : 2754
Tumaticot_  : CTTGTGCACCTCGACTAATTCCGATGGATACTTGCCATTTCGCACCAGCAACAGGTAGCTTGTGCACCTCGACTAATTCCAATGAATACCTGCCA : 2754
Corno_SUN   : CTTGTGCACCTCGACTAATTCCGATGGATACTTGCCATTTCGCACCAGCAACAGGTAGCTTGTGCACCTCGACTAATTCCAATGAATACCTGCCA : 2754
Cuneo_SUN   : CTTGTGCACCTCGACTAATTCCGATGGATACTTGCCATTTCGCACCAGCAACAGGTAGCTTGTGCACCTCGACTAATTCCAATGAATACCTGCCA : 2755
              CTTGTGCACCTCGACTAATTCCGATGGATACTTGCCATTTCGCACCAGCAACAGGTAGCTTGTGCACCTCGACTAATTCCAATGAATACCTGCCA

               2760         *       2780         *       2800         *       2820         *       2840         *
Perennial_  : TCTCCGACCAGCAAAAGGTACCAGGAACAAATGGAAAGAAATCACCTAGTTTATGTCATTGCTGGGAATTGAACGTGAGACCTCATGATACTCAA : 2849
CM334_SUN   : TCTCCGACCAGCAAAAGGTACCAGGAACAAATGGAAAGAAATCACCTAGTTTATGTCATTGCTGGGAATTGAACGTGAGACCTCATGATACTCAA : 2849
Quadrato_S  : TCTCCGACCAGCAAAAGGTACCAGGAACAGATGGAAAGAAATCACCTAGTTTATGTCATTGCTGGGAATTGAACGTGAGACCTCATGATACTCAA : 2849
Tumaticot_  : TCTCCGACCAGCAAAAGGTACCAGGAACAGATGGAAAGAAATCACCTAGTTTATGTCATTGCTGGGAATTGAACGTGAGACCTCATGATACTCAA : 2849
Corno_SUN   : TCTCCGACCAGCAAAAGGTACCAGGAACAGATGGAAAGAAATCACCTAGTTTATGTCATTGCTGGGAATTGAACGTGAGACCTCATGATACTCAA : 2849
Cuneo_SUN   : TCTCCGACCACCAAAAGGTACCAGGAACAGATGGAAAGAAATCACCTAGTTTATGTCATTGCTGGGAATTGAACGTGAGACCTCATGATACTCAA : 2850
              TCTCCGACCAgCAAAAGGTACCAGGAACA ATGGAAAGAAATCACCTAGTTTATGTCATTGCTGGGAATTGAACGTGAGACCTCATGATACTCAA

                    2860         *       2880         *       2900         *       2920         *       2940
Perennial_  : CCCACGTCGTTGACCACTAGCCCACACCCTCGGGTGCCCCAGTGTTCAAAATCTTCAAAATACATATATATGGAGTTCCACACTCACTAGCAAAA : 2944
CM334_SUN   : CCCACGTCGTTGACCACTAGCCCACACCCTCGGGTGCCCCAGTGTTCAAAATCTTCAAAATACATATATATGGAGTTCCACACTCACTAGCAAAA : 2944
Quadrato_S  : CCCACGTCGTTGACCACTAGCCCACACCCTCGGGTGCCCCAGTGTTCAAAATCTTCAAAATACATATATATGGAGTTCCACACTCACTAGCAAAA : 2944
Tumaticot_  : CCCACGTCGTTGACCACTAGCCCACACCCTCGGGTGCCCCAGTGTTCAAAATCTTCAAAATACATATATATGGAGTTCCACACTCACTAGCAAAA : 2944
Corno_SUN   : CCCACGTCGTTGACCACTAGCCCACACCCTCGGGTGCCCCAGTGTTCAAAATCTTCAAAATACATATATATGGAGTTCCACACTCACTAGCAAAA : 2944
Cuneo_SUN   : CCCACGTCGTTGACCACTAGCCCACACCCTCGGGTGCCCCAGTGTTCAAAATCTTCAAAATACATATATATGGAGTTCCACACTCACTAGCAAAA : 2945
              CCCACGTCGTTGACCACTAGCCCACACCCTCGGGTGCCCCAGTGTTCAAAATCTTCAAAATACATATATATGGAGTTCCACACTCACTAGCAAAA

                  *       2960         *       2980         *       3000
Perennial_  : CCACACAAGAAAAAAGGAAGTCTTTTACTACCTCAAAAGCCAAAAACACCCAAACAA : 3001
CM334_SUN   : CCACACAAGAAAAAAGGAAGTCTTTTACTACCTCAAAAGCCAAAAACACCCAAACAA : 3001
Quadrato_S  : GCACACAAGAAAAAAGGAAGTCTTTTACTACCTCAAAAGCCAAAAACACCCAAACAA : 3001
Tumaticot_  : GCACACAAGAAAAAAGGAAGTCTTTTACTACCTCAAAAGCCAAAAACACCCAAACAA : 3001
Corno_SUN   : GCACACAAGAAAAAAGGAAGTCTTTTACTACCTCAAAAGCCAAAAACACCCAAACAA : 3001
Cuneo_SUN   : CCACACAAGAAAAAAGGAAGTCTTTTACTACCTCAAAAGCCAAAAACACCCAAACAA : 3002
               CACACAAGAAAAAAGGAAGTCTTTTACTACCTCAAAAGCCAAAAACACCCAAACAA
```

WUS 3kb upstream

```
                     *        20         *        40         *        60         *        80         *
Perennial : TTATATGACTATAAATCATCTCATCAGAATAGATGAGAAGCTTAAAATTAATTTCTTTTTTAATTTAAAAATACATCATTGTTTTTGCAACAAACC :   96
CM334_WUS : TTATATGACTATAAATCATCTCATCAGAATAGATGAGAAGCTTAAAATTAATTTCTTTTTTAATTTAAAAATACATCATTGTTTTTGCAACAAACC :   96
Corno     : TTATATGACTATAAATCATCTCATCAGAATAGATGAGAAGCTTAAAATTAATTTCTTTTTTAATTTAAAAATACATCATTGTTTTTGCAACAAACC :   96
Cuneo     : TTATATGACTATAAATCATCTCATCAGAATAGATGAGAAGCTTAAAATTAATTTCTTTTTTAATTTAAAAATACATCATTGTTTTTGCAACAAACC :   96
Quadrato  : TTATATGACTATAAATCATCTCATCAGAATAGATGAGAAGCTTAAAATTAATTTCTTTTTTAATTTAAAAATACATCATTGTTTTTGCAACAAACC :   96
Tumaticot : TTATATGACTATAAATCATCTCATCAGAATAGATGAGAAGCTTAAAATTAATTTCTTTTTTAATTTAAAAATACATCATTGTTTTTGCAACAAACC :   96
            TTATATGACTATAAATCATCTCATCAGAATAGATGAGAAGCTTAAAATTAATTTCTTTTTTAATTTAAAAATACATCATTGTTTTTGCAACAAACC

             100         *       120         *       140         *       160         *       180         *
Perennial : AACTAAGTGGAAGAATTTATCACATAAATTGAGACAAATAAATTATTTTATTTGGTGCAGACATACTTGGATCTCGCTATTATATACCCTTCCTAT :  192
CM334_WUS : AACTAAGTGGAAGAATTTATCACATAAATTGAGACAAATAAATTATTTTATTTGGTGCAGACATACTTGGATCTCGCTATTATATACCCTTCCTAT :  192
Corno     : AACTAAGTGGAAGAATTTATCACATAAATTGAGACAAATAAATTATTTTATTTGGTGCAGACATACTTGGATCTCGCTATTATATACCCTTCCTAT :  192
Cuneo     : AACTAAGTGGAAGAATTTATCACATAAATTGAGACAAATAAATTATTTTATTTGGTGCAGACATACTTGGATCTCGCTATTATATACCCTTCCTAT :  192
Quadrato  : AACTAAGTGGAAGAATTTATCACATAAATTGAGACAAATAAATTATTTTATTTGGTGCAGACATACTTGGATCTCGCTATTATATACCCTTCCTAT :  192
Tumaticot : AACTAAGTGGAAGAATTTATCACATAAATTGAGACAAATAAATTATTTTATTTGGTGCAGACATACTTGGATCTCGCTATTATATACCCTTCCTAT :  192
            AACTAAGTGGAAGAATTTATCACATAAATTGAGACAAATAAATTATTTTATTTGGTGCAGACATACTTGGATCTCGCTATTATATACCCTTCCTAT

                 200         *       220         *       240         *       260         *       280
Perennial : TCTATTTACTTTTATTTTCACACTTTGTTCAATACCTATTTTAGTGTTCTCATTAATTAATCTTGATTTGTAATGCATAATACCTATTAGAGTGCA :  288
CM334_WUS : TCTATTTACTTTTATTTTCACACTTTGTTCAATACCTATTTTAGTGTTCTCATTAATTAATCTTGATTTGTAATGCATAATACCTATTAGAGTGCA :  288
Corno     : TCTATTTACTTTTATTTTCACACTTTGTTCAATACCTATTTTAGTGTTCTCATTAATTAATCTTGATTTGTAATGCATAATACCTATTAGAGTGCA :  288
Cuneo     : TCTATTTACTTTTATTTTCACACTTTGTTCAATACCTATTTTAGTGTTCTCATTAATTAATCTTGATTTGTAATGCATAATACCTATTAGAGTGCA :  288
Quadrato  : TCTATTTACTTTTATTTTCACACTTTGTTCAATACCTATTTTAGTGTTCTCATTAATTAATCTTGATTTGTAATGCATAATACCTATTAGAGTGCA :  288
Tumaticot : TCTATTTACTTTTATTTTCACACTTTGTTCAATACCTATTTTAGTGTTCTCATTAATTAATCTTGATTTGTAATGCATAATACCTATTAGAGTGCA :  288
            TCTATTTACTTTTATTTTCACACTTTGTTCAATACCTATTTTAGTGTTCTCATTAATTAATCTTGATTTGTAATGCATAATACCTATTAGAGTGCA

             *       300         *       320         *       340         *       360         *       380
Perennial : TACATACTTGGGTCTCGCTGTGTATCTACCCTATTTTTTTTTCGAAAAAATCTTTGGTTGATGTCTGATATCGTTTTAAAGCTCTCACCAATCAGAA :  384
CM334_WUS : TACATACTTGGGTCTCGCTGTGTATCTACCCTATTTTTTTTTCGAAAAAATCTTTGGTTGATGTCTGATATCGTTTTAAAGCTCTCACCAATCAGAA :  384
Corno     : TACATACTTGGGTCTCGCTGTGTATCTACCCTATTTTTTTTTCGAAAAAATCTTTGGTTGATGTCTGATATCGTTTTAAAGCTCTCACCAATCAGAA :  384
Cuneo     : TACATACTTGGGTCTCGCTGTGTATCTACCCTATTTTTTTTTCGAAAAAATCTTTGGTTGATGTCTGATATCGTTTTAAAGCTCTCACCAATCAGAA :  384
Quadrato  : TACATACTTGGGTCTCGCTGTGTATCTACCCTATTTTTTTTTCGAAAAAATCTTTGGTTGATGTCTGATATCGTTTTAAAGCTCTCACCAATCAGAA :  384
Tumaticot : TACATACTTGGGTCTCGCTGTGTATCTACCCTATTTTTTTTTCGAAAAAATCTTTGGTTGATGTCTGATATCGTTTTAAAGCTCTCACCAATCAGAA :  384
            TACATACTTGGGTCTCGCTGTGTATCTACCCTATTTTTTTTTCGAAAAAATCTTTGGTTGATGTCTGATATCGTTTTAAAGCTCTCACCAATCAGAA

                 *       400         *       420         *       440         *       460         *       480
Perennial : TTCACATAGTATAAGATAATTAAAGGGGAAAATGTTTCTTTAGTGTAAATTCAAAGTAATTCATAATTCGAAACGAAATCTCTAATTAAATTTTAC :  480
CM334_WUS : TTCACATAGTATAAGATAATTAAAGGGGAAAATGTTTCTTTAGTGTAAATTCAAAGTAATTCATAATTCGAAACGAAATCTCTAATTAAATTTTAC :  480
Corno     : TTCACATAGTATAAGATAATTAAAGGGGAAAATGTTTCTTTAGTGTAAATTCAAAGTAATTCATAATTCGAAACGAAATCTCTAATTAAATTTTAC :  480
Cuneo     : TTCACATAGTATAAGATAATTAAAGGGGAAAATGTTTCTTTAGTGTAAATTCAAAGTAATTCATAATTCGAAACGAAATCTCTAATTAAATTTTAC :  480
Quadrato  : TTCACATAGTATAAGATAATTAAAGGGGAAAATGTTTCTTTAGTGTAAATTCAAAGTAATTCATAATTCGAAACGAAATCTCTAATTAAATTTTAC :  480
Tumaticot : TTCACATAGTATAAGATAATTAAAGGGGAAAATGTTTCTTTAGTGTAAATTCAAAGTAATTCATAATTCGAAACGAAATCTCTAATTAAATTTTAC :  480
            TTCACATAGTATAAGATAATTAAAGGGGAAAATGTTTCTTTAGTGTAAATTCAAAGTAATTCATAATTCGAAACGAAATCTCTAATTAAATTTTAC

                     *       500         *       520         *       540         *       560         *
Perennial : CAATCTCACCACAATCCGTACCCTTCGCATCATATGTAAATGTTTTGGCAGTTAATTTAAGATTGGGTAGGTGCAGATTATTATTATATGTATAAT :  576
CM334_WUS : CAATCTCACCACAATCCGTACCCTTCGCATCATATGTAAATGTTTTGGCAGTTAATTTAAGATTGGGTAGGTGCAGATTATTATTATATGTATAAT :  576
Corno     : CAATCTCACCACAATCCGTACCCTTCGCATCATATGTAAATGTTTTGGCAGTTAATTTAAGATTGGGTAGGTGCAGATTATTATTATATGTATAAT :  576
Cuneo     : CAATCTCACCACAATCCGTACCCTTCGCATCATATGTAAATGTTTTGGCAGTTAATTTAAGATTGGGTAGGTGCAGATTATTATTATATGTATAAT :  576
Quadrato  : CAATCTCACCACAATCCGTACCCTTCGCATCATATGTAAATGTTTTGGCAGTTAATTTAAGATTGGGTAGGTGCAGATTATTATTATATGTATAAT :  576
Tumaticot : CAATCTCACCACAATCCGTACCCTTCGCATCATATGTAAATGTTTTGGCAGTTAATTTAAGATTGGGTAGGTGCAGATTATTATTATATGTATAAT :  576
            CAATCTCACCACAATCCGTACCCTTCGCATCATATGTAAATGTTTTGGCAGTTAATTTAAGATTGGGTAGGTGCAGATTATTATTATATGTATAAT

             580         *       600         *       620         *       640         *       660         *
Perennial : AATGTAGAGGTGATATAATATGTCATGGCTTTCAATGTCTGCTCTTTCTCTCATCACTTCACTCTTTTAATTCCATTCTCTTTCCCCCATTTTCTC :  672
CM334_WUS : AATGTAGAGGTGATATAATATGTCATGGCTTTCAATGTCTGCTCTTTCTCTCATCACTTCACTCTTTTAATTCCATTCTCTTTCCCCCATTTTCTC :  672
Corno     : AATGTAGAGGTGATATAATATGTCATGGCTTTCAATGTCTGCTCTTTCTCTCATCACTTCACTCTTTTAATTCCATTCTCTTTCCCCCATTTTCTC :  672
Cuneo     : AATGTAGAGGTGATATAATATGTCATGGCTTTCAATGTCTGCTCTTTCTCTCATCACTTCACTCTTTTAATTCCATTCTCTTTCCCCCATTTTCTC :  672
Quadrato  : AATGTAGAGGTGATATAATATGTCATGGCTTTCAATGTCTGCTCTTTCTCTCATCACTTCACTCTTTTAATTCCATTCTCTTTCCCCCATTTTCTC :  672
Tumaticot : AATGTAGAGGTGATATAATATGTCATGGCTTTCAATGTCTGCTCTTTCTCTCATCACTTCACTCTTTTAATTCCATTCTCTTTCCCCCATTTTCTC :  672
            AATGTAGAGGTGATATAATATGTCATGGCTTTCAATGTCTGCTCTTTCTCTCATCACTTCACTCTTTTAATTCCATTCTCTTTCCCCCATTTTCTC

                 680         *       700         *       720         *       740         *       760
Perennial : TCTTTGTATCCACCTTTAGTTTGTACTTGCTCTTTCCCTAAAAAGTTCATCAATATATACTGTGACTGGTATTTTTGTGTGTATAGTTTTCGTAAC :  768
CM334_WUS : TCTTTGTATCCACCTTTAGTTTGTACTTGCTCTTTCCCTAAAAAGTTCATCAATATATACTGTGACTGGTATTTTTGTGTGTATAGTTTTCGTAAC :  768
Corno     : TCTTTGTATCCACCTTTAGTTTGTACTTGCTCTTTCCCTAAAAAGTTCATCAATATATACTGTGACTGGTATTTTTGTGTGTATAGTTTTCGTAAC :  768
Cuneo     : TCTTTGTATCCACCTTTAGTTTGTACTTGCTCTTTCCCTAAAAAGTTCATCAATATATACTGTGACTGGTATTTTTGTGTGTATAGTTTTCGTAAC :  768
Quadrato  : TCTTTGTATCCACCTTTAGTTTGTACTTGCTCTTTCCCTAAAAAGTTCATCAATATATACTGTGACTGGTATTTTTGTGTGTATAGTTTTCGTAAC :  768
Tumaticot : TCTTTGTATCCACCTTTAGTTTGTACTTGCTCTTTCCCTAAAAAGTTCATCAATATATACTGTGACTGGTATTTTTGTGTGTATAGTTTTCGTAAC :  768
            TCTTTGTATCCACCTTTAGTTTGTACTTGCTCTTTCCCTAAAAAGTTCATCAATATATACTGTGACTGGTATTTTTGTGTGTATAGTTTTCGTAAC

             *       780         *       800         *       820         *       840         *       860
Perennial : CTATTTATTGGCATCCCAAATGAACATATATATATATATATATATATACACTCTATCAAATAGGATGAATGAGGATATATAGTAGTACTAGCTGTG :  864
CM334_WUS : CTATTTATTGGCATCCCAAATGAACATATATATATATATATATATATACACTCTATCAAATAGGATGAATGAGGATATATAGTAGTACTAGCTGTG :  864
Corno     : CTATTTATTGGCATCCCAAATGAACATATATATATATATATATATATACACTCTATCAAATAGGATGAATGAGGATATATAGTAGTACTAGCTGTG :  864
Cuneo     : CTATTTATTGGCATCCCAAATGAACATATATATATATATATATATATACACTCTATCAAATAGGATGAATGAGGATATATAGTAGTACTAGCTGTG :  864
Quadrato  : CTATTTATTGGCATCCCAAATGAACATATATATATATATATATATATACACTCTATCAAATAGGATGAATGAGGATATATAGTAGTACTAGCTGTG :  864
Tumaticot : CTATTTATTGGCATCCCAAATGAACATATATATATATATATATATATACACTCTATCAAATAGGATGAATGAGGATATATAGTAGTACTAGCTGTG :  864
            CTATTTATTGGCATCCCAAATGAACATATATATATATATATATATATACACTCTATCAAATAGGATGAATGAGGATATATAGTAGTACTAGCTGTG

                 *       880         *       900         *       920         *       940         *       960
Perennial : TTTCGTTTTAGCTGGCTCTCTTTCTAAAAATATTTGTTTCAATTTAGTTGTTCCTGTGATGAAATCAAGAGGATTTTATTATGTTCTTCCAATACT :  960
CM334_WUS : TTTCGTTTTAGCTGGCTCTCTTTCTAAAAATATTTGTTTCAATTTAGTTGTTCCTGTGATGAAATCAAGAGGATTTTATTATGTTCTTCCAATACT :  960
Corno     : TTTCGTTTTAGCTGGCTCTCTTTCTAAAAATATTTGTTTCAATTTAGTTGTTCCTGTGATGAAATCAAGAGGATTTTATTATGTTCTTCCAATACT :  960
Cuneo     : TTTCGTTTTAGCTGGCTCTCTTTCTAAAAATATTTGTTTCAATTTAGTTGTTCCTGTGATGAAATCAAGAGGATTTTATTATGTTCTTCCAATACT :  960
Quadrato  : TTTCGTTTTAGCTGGCTCTCTTTCTAAAAATATTTGTTTCAATTTAGTTGTTCCTGTGATGAAATCAAGAGGATTTTATTATGTTCTTCCAATACT :  960
Tumaticot : TTTCGTTTTAGCTGGCTCTCTTTCTAAAAATATTTGTTTCAATTTAGTTGTTCCTGTGATGAAATCAAGAGGATTTTATTATGTTCTTCCAATACT :  960
            TTTCGTTTTAGCTGGCTCTCTTTCTAAAAATATTTGTTTCAATTTAGTTGTTCCTGTGATGAAATCAAGAGGATTTTATTATGTTCTTCCAATACT

                     *       980         *      1000         *      1020         *      1040         *
Perennial : GCCCTCAATATTAAATGACTAGATAAAGTGTATTTACTCAAATTTATATTTTCAAAACATAATTAATAAGATCAATTTAGTAAAATAAATATTTTC : 1056
CM334_WUS : GCCCTCAATATTAAATGACTAGATAAAGTGTATTTACTCAAATTTATATTTTCAAAACATAATTAATAAGATCAATTTAGTAAAATAAATATTTTC : 1056
Corno     : GCCCTCAATATTAAATGACTAGATAAAGTGTATTTACTCAAATTTATATTTTCAAAACATAATTAATAAGATCAATTTAGTAAAATAAATATTTTC : 1056
Cuneo     : GCCCTCAATATTAAATGACTAGATAAAGTGTATTTACTCAAATTTATATTTTCAAAACATAATTAATAAGATCAATTTAGTAAAATAAATATTTTC : 1056
Quadrato  : GCCCTCAATATTAAATGACTAGATAAAGTGTATTTACTCAAATTTATATTTTCAAAACATAATTAATAAGATCAATTTAGTAAAATAAATATTTTC : 1056
Tumaticot : GCCCTCAATATTAAATGACTAGATAAAGTGTATTTACTCAAATTTATATTTTCAAAACATAATTAATAAGATCAATTTAGTAAAATAAATATTTTC : 1056
            GCCCTCAATATTAAATGACTAGATAAAGTGTATTTACTCAAATTTATATTTTCAAAACATAATTAATAAGATCAATTTAGTAAAATAAATATTTTC
```

```
             1060         *       1080         *       1100         *       1120         *       1140         *
Perennial  : TTAAAGAGAATGTCAAGTCAAAAGGGATCAAGTTATTTTGAAACGGAGCAAGTATTACTTTCCCCGTTTGAACTTATAGGATGATTTCTCAATTTC : 1152
CM334_WUS  : TTAAAGAGAATGTCAAGTCAAAAGGGATCAAGTTATTTTGAAACGGAGCAAGTATTACTTTCCCCGTTTGAACTTATAGGATGATTTCTCAATTTC : 1152
Corno      : TTAAAGAGAATGTCAAGTCAAAAGGGATCAAGTTATTTTGAAACGGAGCAAGTATTACTTTCCCCGTTTGAACTTATAGGATGATTTCTCAATTTC : 1152
Cuneo      : TTAAAGAGAATGTCAAGTCAAAAGGGATCAAGTTATTTTGAAACGGAGCAAGTATTACTTTCCCCGTTTGAACTTATAGGATGATTTCTCAATTTC : 1152
Quadrato   : TTAAAGAGAATGTCAAGTCAAAAGGGATCAAGTTATTTTGAAACGGAGCAAGTATTACTTTCCCCGTTTGAACTTATAGGATGATTTCTCAATTTC : 1152
Tumaticot  : TTAAAGAGAATGTCAAGTCAAAAGGGATCAAGTTATTTTGAAACGGAGCAAGTATTACTTTCCCCGTTTGAACTTATAGGATGATTTCTCAATTTC : 1152
             TTAAAGAGAATGTCAAGTCAAAAGGGATCAAGTTATTTTGAAACGGAGCAAGTATTACTTTCCCCGTTTGAACTTATAGGATGATTTCTCAATTTC

                 1160         *       1180         *       1200         *       1220         *       1240
Perennial  : ACGGTCAAATGATATTTTTTTCACAATTTTCATATGTTTTTAAAATATTTTAGACTATTAAGTATTACAAGGTGCAACTTATGATGCTTTTACACT : 1248
CM334_WUS  : ACGGTCAAATGATATTTTTTTCACAATTTTCATATGTTTTTAAAATATTTTAGACTATTAAGTATTACAAGGTGCAACTTATGATGCTTTTACACT : 1248
Corno      : ACGGTCAAATGATATTTTTTTCACAATTTTCATATGTTTTTAAAATATTTTAGACTATTAAGTATTACAAGGTGCAACTTATGATGCTTTTACACT : 1248
Cuneo      : ACGGTCAAATGATATTTTTTTCACAATTTTCATATGTTTTTAAAATATTTTAGACTATTAAGTATTACAAGGTGCAACTTATGATGCTTTTACACT : 1248
Quadrato   : ACGGTCAAATGATATTTTTTTCACAATTTTCATATGTTTTTAAAATATTTTAGACTATTAAGTATTACAAGGTGCAACTTATGATGCTTTTACACT : 1248
Tumaticot  : ACGGTCAAATGATATTTTTTTCACAATTTTCATATGTTTTTAAAATATTTTAGACTATTAAGTATTACAAGGTGCAACTTATGATGCTTTTACACT : 1248
             ACGGTCAAATGATATTTTTTTCACAATTTTCATATGTTTTTAAAATATTTTAGACTATTAAGTATTACAAGGTGCAACTTATGATGCTTTTACACT

               *       1260         *       1280         *       1300         *       1320         *       1340
Perennial  : GTTTTAACTATGTTCATCTTATTTTGAAAAACAAAAAAAACTGATGGTCAAAAGAATAAACGAAATTTAAAAGTTCAAATTCTTAAAAAACAAAAA : 1344
CM334_WUS  : GTTTTAACTATGTTCATCTTATTTTGAAAAACAAAAAAAACTGATGGTCAAAAGAATAAACGAAATTTAAAAGTTCAAATTCTTAAAAAACAAAAA : 1344
Corno      : GTTTTAACTATGTTCATCTTATTTTGAAAAACAAAAAAAACTGATGGTCAAAAGAATAAACGAAATTTAAAAGTTCAAATTCTTAAAAAACAAAAA : 1344
Cuneo      : GTTTTAACTATGTTCATCTTATTTTGAAAAACAAAAAAAACTGATGGTCAAAAGAATAAACGAAATTTAAAAGTTCAAATTCTTAAAAAACAAAAA : 1344
Quadrato   : GTTTTAACTATGTTCATCTTATTTTGAAAAACAAAAAAAACTGATGGTCAAAAGAATAAACGAAATTTAAAAGTTCAAATTCTTAAAAAACAAAAA : 1344
Tumaticot  : GTTTTAACTATGTTCATCTTATTTTGAAAAACAAAAAAAACTGATGGTCAAAAGAATAAACGAAATTTAAAAGTTCAAATTCTTAAAAAACAAAAA : 1344
             GTTTTAACTATGTTCATCTTATTTTGAAAAACAAAAAAAACTGATGGTCAAAAGAATAAACGAAATTTAAAAGTTCAAATTCTTAAAAAACAAAAA

                   *       1360         *       1380         *       1400         *       1420         *       1440
Perennial  : GTATCACATAAATTGGGACAACTTTCAAAATTTGGTAAGACACTCATAATTAAGAATATGACGATACTTGTAAGGTTGAATGACTTCTTTTTATTT : 1440
CM334_WUS  : GTATCACATAAATTGGGACAACTTTCAAAATTTGGTAAGACACTCATAATTAAGAATATGACGATACTTGTAAGGTTGAATGACTTCTTTTTATTT : 1440
Corno      : GTATCACATAAATTGGGACAACTTTCAAAATTTGGTAAGACACTCATAATTAAGAATATGACGATACTTGTAAGGTTGAATGACTTCTTTTTATTT : 1440
Cuneo      : GTATCACATAAATTGGGACAACTTTCAAAATTTGGTAAGACACTCATAATTAAGAATATGACGATACTTGTAAGGTTGAATGACTTCTTTTTATTT : 1440
Quadrato   : GTATCACATAAATTGGGACAACTTTCAAAATTTGGTAAGACACTCATAATTAAGAATATGACGATACTTGTAAGGTTGAATGACTTCTTTTTATTT : 1440
Tumaticot  : GTATCACATAAATTGGGACAACTTTCAAAATTTGGTAAGACACTCATAATTAAGAATATGACGATACTTGTAAGGTTGAATGACTTCTTTTTATTT : 1440
             GTATCACATAAATTGGGACAACTTTCAAAATTTGGTAAGACACTCATAATTAAGAATATGACGATACTTGTAAGGTTGAATGACTTCTTTTTATTT

                       *       1460         *       1480         *       1500         *       1520         *
Perennial  : TTTTTGGCATAACAACGATGATTGGGTTAGTTTATCCGCATTTTAATTATTTTACAGGTTACATTTTGTCTTCCAATGTACGCATGTGTTGTATAA : 1536
CM334_WUS  : TTTTTGGCATAACAACGATGATTGGGTTAGTTTATCCGCATTTTAATTATTTTACAGGTTACATTTTGTCTTCCAATGTACGCATGTGTTGTATAA : 1536
Corno      : TTTTTGGCATAACAACGATGATTGGGTTAGTTTATCCGCATTTTAATTATTTTACAGGTTACATTTTGTCTTCCAATGTACGCATGTGTTGTATAA : 1536
Cuneo      : TTTTTGGCATAACAACGATGATTGGGTTAGTTTATCCGCATTTTAATTATTTTACAGGTTACATTTTGTCTTCCAATGTACGCATGTGTTGTATAA : 1536
Quadrato   : TTTTTGGCATAACAACGATGATTGGGTTAGTTTATCCGCATTTTAATTATTTTACAGGTTACATTTTGTCTTCCAATGTACGCATGTGTTGTATAA : 1536
Tumaticot  : TTTTTGGCATAACAACGATGATTGGGTTAGTTTATCCGCATTTTAATTATTTTACAGGTTACATTTTGTCTTCCAATGTACGCATGTGTTGTATAA : 1536
             TTTTTGGCATAACAACGATGATTGGGTTAGTTTATCCGCATTTTAATTATTTTACAGGTTACATTTTGTCTTCCAATGTACGCATGTGTTGTATAA

             1540         *       1560         *       1580         *       1600         *       1620         *
Perennial  : CTTGGTAGTTTTTGTATAGTACTCCCTTCATTTCGAAATAAATGAATTGTTGGATTTTGACACACAGATTAAGAAAAAAGCATCAAAGACATAAAT : 1632
CM334_WUS  : CTTGGTAGTTTTTGTATAGTACTCCCTTCATTTCGAAATAAATGAATTGTTGGATTTTGACACACAGATTAAGAAAAAAGCATCAAAGACATAAAT : 1632
Corno      : CTTGGTAGTTTTTGTATAGTACTCCCTTCATTTCGAAATAAATGAATTGTTGGATTTTGACACACAGATTAAGAAAAAAGCATCAAAGACATAAAT : 1632
Cuneo      : CTTGGTAGTTTTTGTATAGTACTCCCTTCATTTCGAAATAAATGAATTGTTGGATTTTGACACACAGATTAAGAAAAAAGCATCAAAGACATAAAT : 1632
Quadrato   : CTTGGTAGTTTTTGTATAGTACTCCCTTCATTTCGAAATAAATGAATTGTTGGATTTTGACACACAGATTAAGAAAAAAGCATCAAAGACATAAAT : 1632
Tumaticot  : CTTGGTAGTTTTTGTATAGTACTCCCTTCATTTCGAAATAAATGAATTGTTGGATTTTGACACACAGATTAAGAAAAAAGCATCAAAGACATAAAT : 1632
             CTTGGTAGTTTTTGTATAGTACTCCCTTCATTTCGAAATAAATGAATTGTTGGATTTTGACACACAGATTAAGAAAAAAGCATCAAAGACATAAAT

                 1640         *       1660         *       1680         *       1700         *       1720
Perennial  : TTAACATAAATTCTCATTTTTACCCTTGAAGTCTCTTTTATCCAAAAAAAAGAAAAAGCTTCAGTCTCTTTTACTTGTCAAATCATAAGGGTAATT : 1728
CM334_WUS  : TTAACATAAATTCTCATTTTTACCCTTGAAGTCTCTTTTATCCAAAAAAA-GAAAAAGCTTCAGTCTCTTTTACTTGTCAAATCATAAGGGTAATT : 1727
Corno      : TTAACATAAATTCTCATTTTTACCCTTGAAGTCTCTTTTATCCAAAAAAA-GAAAAAGCTTCAGTCTCTTTTACTTGTCAAATCATAAGGGTAATT : 1727
Cuneo      : TTAACATAAATTCTCATTTTTACCCTTGAAGTYTCTTTTATCCAAAAAAA-GAAAAARCTTMAGTCTCTTTTACTTGTCAAATCATAAGRGTAATT : 1727
Quadrato   : TTAACATAAATTCTCATTTTTACCCTTGAAGTYTCTTTTATCCAAAAAAA-GAAAAARCTTMAGTCTCTTTTACTTGTCAAATCATAAGRGTAATT : 1727
Tumaticot  : TTAACATAAATTCTCATTTTTACCCTTGAAGTCTCTTTTATCCAAAAAAA-GAAAAARCTTMAGTCTCTTTTACTTGTCAAATCATAAGRGTAATT : 1727
             TTAACATAAATTCTCATTTTTACCCTTGAAGT TCTTTTATCCAAAAAAA GAAAAA CTT AGTCTCTTTTACTTGTCAAATCATAAG GTAATT

               *       1740         *       1760         *       1780         *       1800         *       1820
Perennial  : TTGGAAAAAAATTCAACGATTCATTTATTTTGAAACACCAATAAATACTCCAACAATTCATTTATTCCGAAATGGAGGGAGTATTTCTTTTAGCGC : 1824
CM334_WUS  : TTGGAAAAAAATTCAACGATTCATTTATTTTGAAACACCAATAAATACTCCAACAATTCATTTATTCCGAAATGGAGGGAGTATTTCTTTTAGCGC : 1823
Corno      : TTGGAAAAAAATTCAACGATTCATTTATTTTGAAACACCAATAAATACTCCAACAATTCATTTATTCCGAAATGGAGGGAGTATTTCTTTTAGCGC : 1823
Cuneo      : TTRRAAAARAATTCAAYGAWTMAYTTATTTTGAAACAYCAWTAAATACTYYAACAATTYATTTATTYYGAAATGGAGGGAGTATTTCTTTTAGCGC : 1823
Quadrato   : TTRRAAAARAATTCAAYGAWTMAYTTATTTTGAAACAYCAWTAAATACTYYAACAATTYATTTATTYYGAAATGGAGGGAGTATTTCTTTTAGCGC : 1823
Tumaticot  : TTGGAAAAAAATTCAACGATTCATTTATTTTGAAACAYCAWTAAATACTYYAACAATTYATTTATTYYGAAATGGAGGGAGTATTTCTTTTAGCGC : 1823
             TT  AAAA AATTCAA GA T A TTATTTTGAAACA CA TAAATACT  AACAATT ATTTATT  GAAATGGAGGGAGTATTTCTTTTAGCGC

                   *       1840         *       1860         *       1880         *       1900         *       1920
Perennial  : AGTACGTACCGATGGGTAAATGAAGGATAGGGAAGTTTATATAATTTCTTAGAAGATGCAGTACTATAATATACTGTGAATACTTGGTCTCAAAAT : 1920
CM334_WUS  : AGTACGTACCGATGGGTAAATGAAGGATAGGGAAGTTTATATAATTTCTTAGAAGATGCAGTACTATAATATACTGTGAATACTTGGTCTCAAAAT : 1919
Corno      : AGTACGTACCGATGGGTAAATGAAGGATAGGGAAGTTTATATAATTTCTTAGAAGATGCAGTACTATAATATACTGTGAATACTTGGTCTCAAAAT : 1919
Cuneo      : AGTACGTACCGATGGGTAAATGAAGGATAGGGAAGTTTATATAATTTCTTAGAAGATGCAGTACTATAATATACTGTGAATACTTGGTCTCAAAAT : 1919
Quadrato   : AGTACGTACCGATGGGTAAATGAAGGATAGGGAAGTTTATATAATTTCTTAGAAGATGCAGTACTATAATATACTGTGAATACTTGGTCTCAAAAT : 1919
Tumaticot  : AGTACGTACCGATGGGTAAATGAAGGATAGGGAAGTTTATATAATTTCTTAGAAGATGCAGTACTATAATATACTGTGAATACTTGGTCTCAAAAT : 1919
             AGTACGTACCGATGGGTAAATGAAGGATAGGGAAGTTTATATAATTTCTTAGAAGATGCAGTACTATAATATACTGTGAATACTTGGTCTCAAAAT

                       *       1940         *       1960         *       1980         *       2000         *
Perennial  : AGAGAGCAAATGAATGCAATTTTTTATTTTTTTTTTGAAAAAAATGGGGAGATATATAATATGAATGCTATCCTATATATATTCCTGTAGCTGAAGA : 2016
CM334_WUS  : AGAGAGCAAATGAATGCAATTTTTTATTTTTTTTTTGAAAAAAATGGGGAGATATATAATATGAATGCTATCCTATATATATTCCTGTAGCTGAAGA : 2015
Corno      : AGAGAGCAAATGAATGCAATTTTTTATTTTTTTTTTGAAAAAAATGGGGAGATATATAATATGAATGCTATCCTATATATATTCCTGTAGCTGAAGA : 2015
Cuneo      : AGAGAGCAAATGAATGCAATTTTTTATTTTTTTTTTGAAAAAAATGGGGAGATATATAATATGAATGCTATCCTATATATATTCCTGTAGCTGAAGA : 2015
Quadrato   : AGAGAGCAAATGAATGCAATTTTTTATTTTTTTTTTGAAAAAAATGGGGAGATATATAATATGAATGCTATCCTATATATATTCCTGTAGCTGAAGA : 2015
Tumaticot  : AGAGAGCAAATGAATGCAATTTTTTATTTTTTTTTTGAAAAAAATGGGGAGATATATAATATGAATGCTATCCTATATATATTCCTGTAGCTGAAGA : 2015
             AGAGAGCAAATGAATGCAATTTTTTATTTTTTTTTTGAAAAAAATGGGGAGATATATAATATGAATGCTATCCTATATATATTCCTGTAGCTGAAGA

             2020         *       2040         *       2060         *       2080         *       2100         *
Perennial  : AGTTCACTAATGATCTTTTGGATATTTGCCAGTGCTTCAACTAGCAGAAAATTCTGACATTAATGCTACTTCTGAGAATGTGGCTTTAATAATATA : 2112
CM334_WUS  : AGTTCACTAATGATCTTTTGGATATTTGCCAGTGCTTCAACTAGCAGAAAATTCTGACATTAATGCTACTTCTGAGAATGTGGCTTTAATAATATA : 2111
Corno      : AGTTCACTAATGATCTTTTGGATATTTGCCAGTGCTTCAACTAGCAGAAAATTCTGACATTAATGCTACTTCTGAGAATGTGGCTTTAATAATATA : 2111
Cuneo      : AGTTCACTAATGATCTTTTGGATATTTGCCAGTGCTTCAACTAGCAGAAAATTCTGACATTAATGCTACTTCTGAGAATGTGGCTTTAATAATATA : 2111
Quadrato   : AGTTCACTAATGATCTTTTGGATATTTGCCAGTGCTTCAACTAGCAGAAAATTCTGACATTAATGCTACTTCTGAGAATGTGGCTTTAATAATATA : 2111
Tumaticot  : AGTTCACTAATGATCTTTTGGATATTTGCCAGTGCTTCAACTAGCAGAAAATTCTGACATTAATGCTACTTCTGAGAATGTGGCTTTAATAATATA : 2111
             AGTTCACTAATGATCTTTTGGATATTTGCCAGTGCTTCAACTAGCAGAAAATTCTGACATTAATGCTACTTCTGAGAATGTGGCTTTAATAATATA
```

```
                  2120         *       2140         *       2160         *       2180         *       2200
Perennial  : GCCTCCTAATAGAAGCTGCATTTATGTACAAATGACCACACATCATTTGCAAATAAGTAAATTTTTTGAATCTTTTCTGTGTATTTATAAATATAT : 2208
CM334_WUS  : GCCTCCTAATAGAAGCTGCATTTATGTACAAATGACCACACATCATTTGCAAATAAGTAAATTTTTTGAATCTTTTCTGTGTATTTATAAATATAT : 2207
Corno      : GCCTCCTAATAGAAGCTGCATTTATGTACAAATGACCACACATCATTTGCAAATAAGTAAATTTTTTGAATCTTTTCTGTGTATTTATAAATATAT : 2207
Cuneo      : GCCTCCTAATAGAAGCTGCATTTATGTACAAATGACCACACATCATTTGCAAATAAGTAAATTTTTTGAATCTTTTCTGTGTATTTATAAATATAT : 2207
Quadrato   : GCCTCCTAATAGAAGCTGCATTTATGTACAAATGACCACACATCATTTGCAAATAAGTAAATTTTTTGAATCTTTTCTGTGTATTTATAAATATAT : 2207
Tumaticot  : GCCTCCTAATAGAAGCTGCATTTATGTACAAATGACCACACATCATTTGCAAATAAGTAAATTTTTTGAATCTTTTCTGTGTATTTATAAATATAT : 2207
             GCCTCCTAATAGAAGCTGCATTTATGTACAAATGACCACACATCATTTGCAAATAAGTAAATTTTTTGAATCTTTTCTGTGTATTTATAAATATAT

              *       2220         *       2240         *       2260         *       2280         *       2300
Perennial  : ATATATATATATCAGAACTAGTCATTCCTCAGCTTTTAATTACTACTGCATACACATTTACAAGTTTGCCCTAATAACCTTTTTGTAAGTAGCAGC : 2304
CM334_WUS  : ATATATATATATCAGAACTAGTCATTCCTCAGCTTTTAATTACTACTGCATACACATTTACAAGTTTGCCCTAATAACCTTTTTGTAAGTAGCAGC : 2303
Corno      : ATATATATATATCAGAACTAGTCATTCCTCAGCTTTTAATTACTACTGCATACACATTTACAAGTTTGCCCTAATAACCTTTTTGTAAGTAGCAGC : 2303
Cuneo      : ATATATATATATCAGAACTAGTCATTCCTCAGCTTTTAATTACTACTGCATACACATTTACAAGTTTGCCCTAATAACCTTTTTGTAAGTAGCAGC : 2303
Quadrato   : ATATATATATATCAGAACTAGTCATTCCTCAGCTTTTAATTACTACTGCATACACATTTACAAGTTTGCCCTAATAACCTTTTTGTAAGTAGCAGC : 2303
Tumaticot  : ATATATATATATCAGAACTAGTCATTCCTCAGCTTTTAATTACTACTGCATACACATTTACAAGTTTGCCCTAATAACCTTTTTGTAAGTAGCAGC : 2303
             ATATATATATATCAGAACTAGTCATTCCTCAGCTTTTAATTACTACTGCATACACATTTACAAGTTTGCCCTAATAACCTTTTTGTAAGTAGCAGC

                  *       2320         *       2340         *       2360         *       2380         *       2400
Perennial  : TAGAGTACAAAAGAATATTTAAAATTCTGGCATAATTTAATCAGAGACAAAGAGGCCATATATAAAGAAGAATTCCCCTAAATGAAAGACTAAAAT : 2400
CM334_WUS  : TAGAGTACAAAAGAATATTTAAAATTCTGGCATAATTTAATCAGAGACAAAGAGGCCATATATAAAGAAGAATTCCCCTAAATGAAAGACTAAAAT : 2399
Corno      : TAGAGTACAAAAGAATATTTAAAATTCTGGCATAATTTAATCAGAGACAAAGAGGCCATATATAAAGAAGAATTCCCCTAAATGAAAGACTAAAAT : 2399
Cuneo      : TAGAGTACAAAAGAATATTTAAAATTCTGGCATAATTTAATCAGAGACAAAGAGGCCATATATAAAGAAGAATTCCCCTAAATGAAAGACTAAAAT : 2399
Quadrato   : TAGAGTACAAAAGAATATTTAAAATTCTGGCATAATTTAATCAGAGACAAAGAGGCCATATATAAAGAAGAATTCCCCTAAATGAAAGACTAAAAT : 2399
Tumaticot  : TAGAGTACAAAAGAATATTTAAAATTCTGGCATAATTTAATCAGAGACAAAGAGGCCATATATAAAGAAGAATTCCCCTAAATGAAAGACTAAAAT : 2399
             TAGAGTACAAAAGAATATTTAAAATTCTGGCATAATTTAATCAGAGACAAAGAGGCCATATATAAAGAAGAATTCCCCTAAATGAAAGACTAAAAT

                      *       2420         *       2440         *       2460         *       2480         *
Perennial  : CAAAAGAAGAACATATAATTTATACATTCATTCATTCATTTGACAAGAGAAGCTCTAATACTTTTTCTCTTATTCTGTCTTCTCATAAAGCACCCT : 2496
CM334_WUS  : CAAAAGAAGAACATATAATTTATACATTCATTCATTCATTTGACAAGAGAAGCTCTAATACTTTTTCTCTTATTCTGTCTTCTCATAAAGCACCCT : 2495
Corno      : CAAAAGAAGAACATATAATTTATACATTCATTCATTCATTTGACAAGAGAAGCTCTAATACTTTTTCTCTTATTCTGTCTTCTCATAAAGCACCCT : 2495
Cuneo      : CAAAAGAAGAACATATAATTTATACATTCATTCATTCATTTGACAAGAGAAGCTCTAATACTTTTTCTCTTATTCTGTCTTCTCATAAAGCACCCT : 2495
Quadrato   : CAAAAGAAGAACATATAATTTATACATTCATTCATTCATTTGACAAGAGAAGCTCTAATACTTTTTCTCTTATTCTGTCTTCTCATAAAGCACCCT : 2495
Tumaticot  : CAAAAGAAGAACATATAATTTATACATTCATTCATTCATTTGACAAGAGAAGCTCTAATACTTTTTCTCTTATTCTGTCTTCTCATAAAGCACCCT : 2495
             CAAAAGAAGAACATATAATTTATACATTCATTCATTCATTTGACAAGAGAAGCTCTAATACTTTTTCTCTTATTCTGTCTTCTCATAAAGCACCCT

             2500         *       2520         *       2540         *       2560         *       2580         *
Perennial  : TTTTTCTTTCCCATATATATTGTCCCAACTTAATAATCATAACATATTGTCATTGCCTTTTCATCCAAGCAAACTCCTCTGAAATCCAACCTTTTT : 2592
CM334_WUS  : TTTTTCTTTCCCATATATATTGTCCCAACTTAATAATCATAACATATTGTCATTGCCTTTTCATCCAAGCAAACTCCTCTGAAATCCAACCTTTTT : 2591
Corno      : TTTTTCTTTCCCATATATATTGTCCCAACTTAATAATCATAACATATTGTCATTGCCTTTTCATCCAAGCAAACTCCTCTGAAATCCAACCTTTTT : 2591
Cuneo      : TTTTTCTTTCCCATATATATTGTCCCAACTTAATAATCATAACATATTGTCATTGCCTTTTCATCCAAGCAAACTCCTCTGAAATCCAACCTTTTT : 2591
Quadrato   : TTTTTCTTTCCCATATATATTGTCCCAACTTAATAATCATAACATATTGTCATTGCCTTTTCATCCAAGCAAACTCCTCTGAAATCCAACCTTTTT : 2591
Tumaticot  : TTTTTCTTTCCCATATATATTGTCCCAACTTAATAATCATAACATATTGTCATTGCCTTTTCATCCAAGCAAACTCCTCTGAAATCCAACCTTTTT : 2591
             TTTTTCTTTCCCATATATATTGTCCCAACTTAATAATCATAACATATTGTCATTGCCTTTTCATCCAAGCAAACTCCTCTGAAATCCAACCTTTTT

                 2600         *       2620         *       2640         *       2660         *       2680
Perennial  : TTAGTCCCCTTTTGTCCCATACTTTTTCTTTCCTTGTTACCCTTTCCTCTGTCAATTTCAGTCCTTCTCTCCCAGATATACCTCTTGATTTTCTNN : 2688
CM334_WUS  : TTAGTCCCCTTTTGTCCCATACTTTTTCTTTCCTTGTTACCCTTTCCTCTGTCAATTTCAGTCCTTCTCTCCCAGATATACCTCTTGATTTTCTNN : 2687
Corno      : TTAGTCCCCTTTTGTCCCATACTTTTTCTTTCCTTGTTACCCTTTCCTCTGTCAATTTCAGTCCTTCTCTCCCAGATATACCTCTTGATTTTCTNN : 2687
Cuneo      : TTAGTCCCCTTTTGTCCCATACTTTTTCTTTCCTTGTTACCCTTTCCTCTGTCAATTTCAGTCCTTCTCTCCCAGATATACCTCTTGATTTTCTNN : 2687
Quadrato   : TTAGTCCCCTTTTGTCCCATACTTTTTCTTTCCTTGTTACCCTTTCCTCTGTCAATTTCAGTCCTTCTCTCCCAGATATACCTCTTGATTTTCTNN : 2687
Tumaticot  : TTAGTCCCCTTTTGTCCCATACTTTTTCTTTCCTTGTTACCCTTTCCTCTGTCAATTTCAGTCCTTCTCTCCCAGATATACCTCTTGATTTTCTNN : 2687
             TTAGTCCCCTTTTGTCCCATACTTTTTCTTTCCTTGTTACCCTTTCCTCTGTCAATTTCAGTCCTTCTCTCCCAGATATACCTCTTGATTTTCTNN

              *       2700         *       2720         *       2740         *       2760         *       2780
Perennial  : NNNNNNNNNNNNNNNNNNNNNNNACACCCACCCAACAACACTTCCAAAAAAAATCTCTTTATAATTCTCTCATTCTCTTAACTCATTCTTAAGTCT : 2784
CM334_WUS  : NNNNNNNNNNNNNNNNNNNNNNNACACCCACCCAACAACACTTCCAAAAAAAATCTCTTTATAATTCTCTCATTCTCTTAACTCATTCTTAAGTCT : 2783
Corno      : NNNNNNNNNNNNNNNNNNNNNNNACACCCACCCAACAACACTTCCAAAAAAAATCTCTTTATAATTCTCTCATTCTCTTAACTCATTCTTAAGTCT : 2783
Cuneo      : NNNNNNNNNNNNNNNNNNNNNNNACACCCACCCAACAACACTTCCAAAAAAAATCTCTTTATAATTCTCTCATTCTCTTAACTCATTCTTAAGTCT : 2783
Quadrato   : NNNNNNNNNNNNNNNNNNNNNNNACACCCACCCAACAACACTTCCAAAAAAAATCTCTTTATAATTCTCTCATTCTCTTAACTCATTCTTAAGTCT : 2783
Tumaticot  : NNNNNNNNNNNNNNNNNNNNNNNACACCCACCCAACAACACTTCCAAAAAAAATCTCTTTATAATTCTCTCATTCTCTTAACTCATTCTTAAGTCT : 2783
             NNNNNNNNNNNNNNNNNNNNNNNACACCCACCCAACAACACTTCCAAAAAAAATCTCTTTATAATTCTCTCATTCTCTTAACTCATTCTTAAGTCT

                  *       2800         *       2820         *       2840         *       2860         *       2880
Perennial  : TCAGTCTTTTACCAACCTAGCCTAATAGCCACATTCTTCACTTTTTATTATCTGTTTGTTTTTTGTCTACATCCTTTTGTTCTCTTGTTGTTTTAG : 2880
CM334_WUS  : TCAGTCTTTTACCAACCTAGCCTAATAGCCACATTCTTCACTTTTTATTATCTGTTTGTTTTTTGTCTACATCCTTTTGTTCTCTTGTTGTTTTAG : 2879
Corno      : TCAGTCTTTTACCAACCTAGCCTAATAGCCACATTCTTCACTTTTTATTATCTGTTTGTTTTTTGTCTACATCCTTTTGTTCTCTTGTTGTTTTAG : 2879
Cuneo      : TCAGTCTTTTACCAACCTAGCCTAATAGCCACATTCTTCACTTTTTATTATCTGTTTGTTTTTTGTCTACATCCTTTTGTTCTCTTGTTGTTTTAG : 2879
Quadrato   : TCAGTCTTTTACCAACCTAGCCTAATAGCCACATTCTTCACTTTTTATTATCTGTTTGTTTTTTGTCTACATCCTTTTGTTCTCTTGTTGTTTTAG : 2879
Tumaticot  : TCAGTCTTTTACCAACCTAGCCTAATAGCCACATTCTTCACTTTTTATTATCTGTTTGTTTTTTGTCTACATCCTTTTGTTCTCTTGTTGTTTTAG : 2879
             TCAGTCTTTTACCAACCTAGCCTAATAGCCACATTCTTCACTTTTTATTATCTGTTTGTTTTTTGTCTACATCCTTTTGTTCTCTTGTTGTTTTAG

                      *       2900         *       2920         *       2940         *       2960         *
Perennial  : TACTACTAGTAGCTCTATTCTATCTATCTATCATGGAACACCAACAACAAAACCAACAGCACTATCTTCACCAACAACACATAGAAGATGGTGTTG : 2976
CM334_WUS  : TACTACTAGTAGCTCTATTCTATCTATCTATCATGGAACACCAACAACAAAACCAACAGCACTATCTTCACCAACAACACATAGAAGATGGTGTTG : 2975
Corno      : TACTACTAGTAGCTCTATTCTATCTATCTATCATGGAACACCAACAACAAAACCAACAGCACTATCTTCACCAACAACACATAGAAGATGGTGTTG : 2975
Cuneo      : TACTACTAGTAGCTCTATTCTATCTATCTATCATGGAACACCAACAACAAAACCAACAGCACTATCTTCACCAACAACACATAGAAGATGGTGTTG : 2975
Quadrato   : TACTACTAGTAGCTCTATTCTATCTATCTATCATGGAACACCAACAACAAAACCAACAGCACTATCTTCACCAACAACACATAGAAGATGGTGTTG : 2975
Tumaticot  : TACTACTAGTAGCTCTATTCTATCTATCTATCATGGAACACCAACAACAAAACCAACAGCACTATCTTCACCAACAACACATAGAAGATGGTGTTG : 2975
             TACTACTAGTAGCTCTATTCTATCTATCTATCATGGAACACCAACAACAAAACCAACAGCACTATCTTCACCAACAACACATAGAAGATGGTGTTG

             2980         *       3000         *
Perennial  : GCAGCAAAAACAACAACAACAGTAGNGGCAGCAACAGTTTCA : 3018
CM334_WUS  : GCAGCAAAA----------------NGGCAGCAACAGTTTCA : 3001
Corno      : GCAGCAAAAACAACAACAACAGTAGNGGCAGCAACAGTTTCA : 3017
Cuneo      : GCAGCAAAAACAACAACAACAGTAGNGGCAGCAACAGTTTCA : 3017
Quadrato   : GCAGCAAAAACAACAACAACAGTAGNGGCAGCAACAGTTTCA : 3017
Tumaticot  : GCAGCAAAAACAACAACAACAGTAGNGGCAGCAACAGTTTCA : 3017
             GCAGCAAAAacaacaacaacagtagNGGCAGCAACAGTTTCA
```

CLV3 - 3kb upstream

```
                       *        20         *        40         *        60         *        80         *
Corno       : CTCACTTTTGTCTAGAAAGTAAGTAAAAAATATACTTATATGATCCTTAAAGAATCAACAGATTTTACTATTTGCAGTAAAAGAGCCAGTCACAT :   95
Quadrato    : CTCACTTTTGTCTAGAAAGTAAGTAAAAAATATACTTATATGATCCTTAAAGAATCAACAGATTTTACTATTTGCAGTAAAAGAGCCAGTCACAT :   95
Tumaticot   : CTCACTTTTGTCTAGAAAGTAAGTAAAAAATATACTTATATGATCCTTAAAGAATCAACAGATTTTACTATTTGCAGTAAAAGAGCCAGTCACAT :   95
Cuneo       : CTCACTTTTGTCTAGAAAGTAAGTAAAAAATATACTTATATGATCCTTAAAGAATCAACAGATTTTACTATTTGCAGTAAAAGAGCCAGTCACAT :   95
Perennial   : CTCACTTTTGTCTAGAAAGTAAGTAAAAAATATACTTATATGATCCTTAAAGAATCAACAGATTTTACTATTTGCAGTAAAAGAGCCAGTCACAT :   95
CM334_CLV3  : CTCACTTTTGTCTAGAAAGTAAGTAAAAAATATACTTATATGATCCTTAAAGAATCAACAGATTTTACTATTTGCAGTAAAAGAGCCAGTCACAT :   95
              CTCACTTTTGTCTAGAAAGTAAGTAAAAAATATACTTATATGATCCTTAAAGAATCAACAGATTTTACTATTTGCAGTAAAAGAGCCAGTCACAT

                100         *      120         *      140         *      160         *      180         *
Corno       : TTGGCAAATTTAATTAAAGACAAATTCTGTGTAATTTTGACTAGGAGTTAG---GGCCGCGCATTCTTATTAGGGGATTCTTTGTTGGAATATAA :  187
Quadrato    : TTGGCAAATTTAATTAAAGACAAATTCTGTGTAATTTTGACTAGGAGTTAG---GGCCGCGCATTCTTATTAGGGGATTCTTTGTTGGAATATAA :  187
Tumaticot   : TTGGCAAATTTAATTAAAGACAAATTCTGTGTAATTTTGACTAGGAGTTAGTAAGGCCGCGCATTCTTATTAGGGGATTCTTTGTTGGAATATAA :  190
Cuneo       : TTGGCAAATTTAATTAAAGACAAATTCTGTGTAATTTTGACTAGGAGTTAGTAAGGCCGCGCATTCTTATTAGGGGATTCTTTGTTGGAATATAA :  190
Perennial   : TTGGCAAATTTAATTAAAGACAAATTCTGTATAATTTTGACTAGGAGTTAG---GGCCGCGCATTCTTATTAGGGGATTCTTTGTTGGAATATAA :  187
CM334_CLV3  : TTGGCAAATTTAATTAAAGACAAATTCTGTATAATTTTGACTAGGAGTTAG---GGCCGCGCATTCTTATTAGGGGATTCTTTGTTGGAATATAA :  187
              TTGGCAAATTTAATTAAAGACAAATTCTGT TAATTTTGACTAGGAGTTAG   GGCCGCGCATTCTTATTAGGGGATTCTTTGTTGGAATATAA

                     200         *      220         *      240         *      260         *      280
Corno       : CTTACGATACCTGATGGTAGTTTAACCAAAAAATATGTATATATATATATATATATATATTAGGGGATTCAAAGAGGATCAASCTAGAATGTATG :  282
Quadrato    : CTTACGATACCTGATGGTAGTTTAACCAAAAAATATGTATATATATATATATATATATATTAGGGGATTCAAAGAGGATCAASCTAGAATGTATG :  282
Tumaticot   : CTTACGATACCTGATGGTAGTTTAACCAAAAAATATGTATATATATATATATATATATATTAGGGGATTCAAAGAGGATCAAGCTAGAATGTATG :  285
Cuneo       : CTTACGATACCTGATGGTAGTTTAACCAAAAAATATGTATATATATATATATATATATATTAGGGGATTCAAAGAGGATCAAGCTAGAATGTATG :  285
Perennial   : CTTACGATACCTGATGGTAGTTTAACCAAAAAATATGTATATATATATATATATATATATTAGGGGATTCAAAGAGGATCAAGCTAGAATGTATG :  282
CM334_CLV3  : CTTACGATACCTGATGGTAGTTTAACCAAAAAATATGTATATATATATATATATATATATTAGGGGATTCAAAGAGGATCAAGCTAGAATGTATG :  282
              CTTACGATACCTGATGGTAGTTTAACCAAAAAATATGTATATATATATATATATATATATTAGGGGATTCAAAGAGGATCAA CTAGAATGTATG

                  *      300         *      320         *      340         *      360         *      380
Corno       : TGAAATATAATGAGATTCAAATTTGCGATCTACTTCAAATATAAACACAACAATTTATTTATATCATATTTGACACAGTATAATTTTCTGATAAA :  377
Quadrato    : TGAAATATAATGAGATTCAAATTTGCGATCTACTTCAAATATAAACACAACAATTTATTTATATCATATTTGACACAGTATAATTTTCTGATAAA :  377
Tumaticot   : TGAAATATAATGAGATTCAAATTTGCGATCTACTTCAAATATAAACACAACAATTTATTTATATCATATTTGACACAGTATAATTTTCTGATAAA :  380
Cuneo       : TGAAATATAATGAGATTCAAATTTGCGATCTACTTCAAATATAAACACAACAATTTATTTATATCATATTTGACACAGTATAATTTTCTGATAAA :  380
Perennial   : TGAAATATAATGAGATTCAAATTTGCGATCTACTTCAAATATAAACACAACAATTTATTTATATCATATTTGACACAGTATAATTTTCTGATAAA :  377
CM334_CLV3  : TGAAATATAATGAGATTCAAATTTGCGATCTACTTCAAATATAAACACAACAATTTATTTATATCATATTTGACACAGTATAATTTTCTGATAAA :  377
              TGAAATATAATGAGATTCAAATTTGCGATCTACTTCAAATATAAACACAACAATTTATTTATATCATATTTGACACAGTATAATTTTCTGATAAA

                       *       400         *       420         *       440         *       460         *
Corno       : AGAGATCGATCCTGTTAAACATCCTTCATTATTGTAGATCCGCCCCCATTTCCAATTGAGAAAGAGGGAACAAGCAAATGGGGGTGGATTTTACT :  472
Quadrato    : AGAGATCGATCCTGTTAAACATCCTTCATTATTGTAGATCCGCCCCCATTTCCAATTGAGAAAGAGGGAACAAGCAAATGGGGGTGGATTTTACT :  472
Tumaticot   : AGAGATCGATCCTGTTAAACATCCTTCATTATTGTAGATCCGCCCCCATTTCCAATTGAGAAAGAGGGAACAAGCAAATGGGGGTGGATTTTACT :  475
Cuneo       : AGAGATCGATCCTGTTAAACATCCTTCATTATTGTAGATCCGCCCCCATTTCCAATTGAGAAAGAGGGAACAAGCAAATGGGGGTGGATTTTACT :  475
Perennial   : AGAGATCGATCCTGTTAAACATCCTTCATTATTGTAGATCCGCCCCCATTTCCAATTGAGAAAGAGGGAACAAGCAAATGGGGGTGGATTTTACT :  472
CM334_CLV3  : AGAGATCGATCCTGTTAAACATCCTTCATTATTGTAGATCCGCCCCCATTTCCAATTGAGAAAGAGGGAACAAGCAAATGGGGGTGGATTTTACT :  472
              AGAGATCGATCCTGTTAAACATCCTTCATTATTGTAGATCCGCCCCCATTTCCAATTGAGAAAGAGGGAACAAGCAAATGGGGGTGGATTTTACT

                480         *      500         *      520         *      540         *      560         *
Corno       : AAATAATTTAAGCCATGTACTCCATCTCAATTGTGGAAGTATTCCACAAACGAACTTTTCTTGAGTTGATCTCTCAGATAGTCAGTATTTCTAAT :  567
Quadrato    : AAATAATTTAAGCCATGTACTCCATCTCAATTGTGGAAGTATTCCACAAACGAACTTTTCTTGAGTTGATCTCTCAGATAGTCAGTATTTCTAAT :  567
Tumaticot   : AAATAATTTAAGCCATGTACTCCATCTCAATTGTGGAAGTATTCCACAAACGAACTTTTCTTGAGTTGATCTCTCAGATAGTCAGTATTTCTAAT :  570
Cuneo       : AAATAATTTAAGCCATGTACTCCATCTCAATTGTGGAAGTATTCCACAAACGAACTTTTCTTGAGTTGATCTCTCAGATAGTCAGTATTTCTAAT :  570
Perennial   : AAATAATTTAAGCCATGTACTCCATCTCAATTGTGGAAGTATTCCACAAACGAACTTTTCTTGAGTTGATCTCTCAGATAGTCAGTATTTCTAAT :  567
CM334_CLV3  : AAATAATTTAAGCCATGTACTCCATCTCAATTGTGGAAGTATTCCACAAACGAACTTTTCTTGAGTTGATCTCTCAGATAGTCAGTATTTCTAAT :  567
              AAATAATTTAAGCCATGTACTCCATCTCAATTGTGGAAGTATTCCACAAACGAACTTTTCTTGAGTTGATCTCTCAGATAGTCAGTATTTCTAAT

                     580         *      600         *      620         *      640         *      660
Corno       : AGTCGATATTTCAGAAAATCACTTGTTTTGTTGAACAAAAAAATTTGACTTAGAAAAAATGACTTTAGAGATAATAACTAAATCAAACCCTAAATT :  662
Quadrato    : AGTCGATATTTCAGAAAATCACTTGTTTTGTTGAACAAAAAAATTTGACTTAGAAAAAATGACTTTAGAGATAATAACTAAATCAAACCCTAAATT :  662
Tumaticot   : AGTCGATATTTCAGAAAATCACTTGTTTTGTTGAACAAAAAAATTTGACTTAGAAAAAATGACTTTAGAGATAATAACTAAATCAAACCCTAAATT :  665
Cuneo       : AGTCGATATTTCAGAAAATCACTTGTTTTGTTGAACAAAAAAATTTGACTTAGAAAAAATGACTTTAGAGATAATAACTAAATCAAACCCTAAATT :  665
Perennial   : AGTCGATATTTCAGAAAATCACTTGTTTTGTTGAACAAAAAAATTTGACTTAGAAAAAATGACTTTAGAGATAATAACTAAATCAAACCCTAAATT :  662
CM334_CLV3  : AGTCGATATTTCAGAAAATCACTTGTTTTGTTGAACAAAAAAATTTGACTTAGAAAAAATGACTTTAGAGATAATAACTAAATCAAACCCTAAATT :  662
              AGTCGATATTTCAGAAAATCACTTGTTTTGTTGAACAAAAAAATTTGACTTAGAAAAAATGACTTTAGAGATAATAACTAAATCAAACCCTAAATT

                  *      680         *      700         *      720         *      740         *      760
Corno       : TTTCTCTAAGGACCCCAAATTACCTATGACATTGTGATTTGTGATGGAAACGTTGGTGCGGAATTCTTGGATTCCTAGTATTTTGTTTTGTTAGG :  757
Quadrato    : TTTCTCTAAGGACCCCAAATTACCTATGACATTGTGATTTGTGATGGAAACGTTGGTGCGGAATTCTTGGATTCCTAGTATTTTGTTTTGTTAGG :  757
Tumaticot   : TTTCTCTAAGGACCCCAAATTACCTATGACATTGTGATTTGTGATGGAAACGTTGGTGCGGAATTCTTGGATTCCTAGTATTTTGTTTTGTTAGG :  760
Cuneo       : TTTCTCTAAGGACCCCAAATTACCTATGACATTGTGATTTGTGATGGAAACGTTGGTGCGGAATTCTTGGATTCCTAGTATTTTGTTTTGTTAGG :  760
Perennial   : TTTCTCTAAGGACCCCAAATTACCTATGACATTGTGATTTGTGATGGAAACGTTGGTGCGGAATTCTTGGATTCCTAGTATTTTGTTTTGTTAGG :  757
CM334_CLV3  : TTTCTCTAAGGACCCCAAATTACCTATGACATTGTGATTTGTGATGGAAACGTTGGTGCGGAATTCTTGGATTCCTAGTATTTTGTTTTGTTAGG :  757
              TTTCTCTAAGGACCCCAAATTACCTATGACATTGTGATTTGTGATGGAAACGTTGGTGCGGAATTCTTGGATTCCTAGTATTTTGTTTTGTTAGG

                       *       780         *       800         *       820         *       840         *
Corno       : GTAACATTACAACAATAACAACATATTCAGTGTATGGTAAAGTGTACGCAGTCCATCCCGACCACTACCTCAGATGAAGTAGAGAGGTTGTTTCC :  852
Quadrato    : GTAACATTACAACAATAACAACATATTCAGTGTATGGTAAAGTGTACGCAGTCCATCCCGACCACTACCTCAGATGAAGTAGAGAGGTTGTTTCC :  852
Tumaticot   : GTAACATTACAACAATAACAACATATTCAGTGTATGGTAAAGTGTACGCAGTCCATCCCGACCACTACCTCAGATGAAGTAGAGAGGTTGTTTCC :  855
Cuneo       : GTAACATTACAACAATAACAACATATTCAGTGTATGGTAAAGTGTACGCAGTCCATCCCGACCACTACCTCAGATGAAGTAGAGAGGTTGTTTCC :  855
Perennial   : GTAACATTACAACAATAACAACATATTCAGTGTATGGTAAAGTGTACGCAGTCCATCCCGACCACTAACTCAGATGAAGTAGAGAGGTTGTTTCC :  852
CM334_CLV3  : GTAACATTACAACAATAACAACATATTCAGTGTATGGTAAAGTGTACGCAGTCCATCCCGACCACTAACTCAGATGAAGTAGAGAGGTTGTTTCC :  852
              GTAACATTACAACAATAACAACATATTCAGTGTATGGTAAAGTGTACGCAGTCCATCCCGACCACTA CTCAGATGAAGTAGAGAGGTTGTTTCC

                860         *      880         *      900         *      920         *      940         *
Corno       : GATAGACCCCTCGGCTCATGTTWGTGTAACATTGACAATAGAAATTCCTCTTTCCCTTCATGGTATTTTCTCCTCCTTTTCTTCTCTACTAGCAT :  947
Quadrato    : GATAGACCCCTCGGCTCATGTTWGTGTAACATTGACAATAGAAATTCCTCTTTCCCTTCATGGTATTTTCTCCTCCTTTTCTTCTCTACTAGCAT :  947
Tumaticot   : GATAGACCCCTCGGCTCATGTTAGTGTAACATTGACAATAGAAATTCCTCTTTCCCTTCATGGTATTTTCTCCTCCTTTTCTTCTCTACTAGCAT :  950
Cuneo       : GATAGACCCCTCGGCTCATGTTAGTGTAACATTGACAATAGAAATTCCTCTTTCCCTTCATGGTATTTTCTCCTCCTTTTCTTCTCTACTAGCAT :  950
Perennial   : GATAGACCCCTCGGCTCATGTTAGTGTAACATTGACAATAGAAATTCCTCTTTCCCTTCATGGTATTTTCTCCTCCTTTTCTTCTCTACTAGCAT :  947
CM334_CLV3  : GATAGACCCCTCGGCTCATGTTAGTGTAACATTGACAATAGAAATTCCTCTTTCCCTTCATGGTATTTTCTCCTCCTTTTCTTCTCTACTAGCAT :  947
              GATAGACCCCTCGGCTCATGTT GTGTAACATTGACAATAGAAATTCCTCTTTCCCTTCATGGTATTTTCTCCTCCTTTTCTTCTCTACTAGCAT

                     960         *      980         *     1000         *     1020         *     1040
Corno       : TATCACTAGTAATAAAAAGGCTATGAAGAGAGACTAGAATTCCTTTTCATGGGTAATAACTAACATAAGGAGTAAAGTAGCCTCTTGGACTCATC : 1042
Quadrato    : TATCACTAGTAATAAAAAGGCTATGAAGAGAGACTAGAATTCCTTTTCATGGGTAATAACTAACATAAGGAGTAAAGTAGCCTCTTGGACTCATC : 1042
Tumaticot   : TATCACTAGTAATAAAAAGGCTATGAAGAGAGACTAGAATTCCTTTTCATGGGTAATAACTAACATAAGGAGTAAAGTAGCCTCTTGGACTCATC : 1045
Cuneo       : TATCACTAGTAATAAAAAGGCTATGAAGAGAGACTAGAATTCCTTTTCATGGGTAATAACTAACATAAGGAGTAAAGTAGCCTCTTGGACTCATC : 1045
Perennial   : TATCACTAGTAATAAAAAGGCTATGAAGAGAGACTAGAATTCCTGTTCATGGGTAATAACTAACATAAGGAGTAAAGTAGCCTCTTGGACTCATC : 1042
CM334_CLV3  : TATCACTAGTAATAAAAAGGCTATGAAGAGAGACTAGAATTCCTGTTCATGGGTAATAACTAACATAAGGAGTAAAGTAGCCTCTTGGACTCATC : 1042
              TATCACTAGTAATAAAAAGGCTATGAAGAGAGACTAGAATTCCT TTCATGGGTAATAACTAACATAAGGAGTAAAGTAGCCTCTTGGACTCATC
```

```
                      *      1060         *      1080         *      1100         *      1120         *      1140
Corno       : TCTTTTATTTTATTTCTTTTGCCTTTCGGTGCTCTATCTTCGACTTTTTCTGACCATTGTTCAGTCACACAGAAAAGTCAGTAAACTAAGAAAA : 1137
Quadrato    : TCTTTTATTTTATTTCTTTTGCCTTTCGGTGCTCTATCTTCGACTTTTTCTGACCATTGTTCAGTCACACAGAAAAGTCAGTAAACTAAGAAAA : 1137
Tumaticot   : TCTTTTATTTTATTTCTTTTGCCTTTCGGTGCTCTATCTTCGACTTTTTCTGACCATTGTTCAGTCACACAGAAAAGTCAGTAAACTAAGAAAA : 1140
Cuneo       : TCTTTTATTTTATTTCTTTTGCCTTTCGGTGCTCTATCTTCGACTTTTTCTGACCATTGTTCAGTCACACAGAAAAGTCAGTAAACTAAGAAAA : 1140
Perennial   : TCTTTTATTTTATTTCTTTTGCCTTTCGGTGCTCTATCTTCGACTTTTTCTGACCATTGTTCAGTCACACAGAAAAGTCAGTAAACTAAGAAAA : 1137
CM334_CLV3  : TCTTTTATTTTATTTCTTTTGCCTTTCGGTGCTCTATCTTCGACTTTTTCTGACCATTGTTCAGTCACACAGAAAAGTCAGTAAACTAAGAAAA : 1137
              TCTTTTATTTTATTTCTTTTGCCTTTCGGTGCTCTATCTTCGACTTTTTCTGACCATTGTTCAGTCACACAGAAAAGTCAGTAAACTAAGAAAA

                      *      1160         *      1180         *      1200         *      1220         *
Corno       : TGACATGACCACAACTAAAATCATGAAGTACATAATATGAGAGTAAGCAAATTCAAATATCTTTTAAAAAAAAAAAAAAATCAAGAGAAAAAACAA : 1232
Quadrato    : TGACATGACCACAACTAAAATCATGAAGTACATAATATGAGAGTAAGCAAATTCAAATATCTTTTAAAAAAAAAAAAAAATCAAGAGAAAAAACAA : 1232
Tumaticot   : TGACATGACCACAACTAAAATCATGAAGTACATAATATGAGAGTAAGCAAATTCAAATATCTTTTAAAAAAAAAAAAAAATCAAGAGAAAAAACAA : 1235
Cuneo       : TGACATGACCACAACTAAAATCATGAAGTACATAATATGAGAGTAAGCAAATTCAAATATCTTTTAAAAAAAAAAAAAAATCAAGAGAAAAAACAA : 1235
Perennial   : TGACATGACCACAACTAAAATCATGAAGTACATAATATGAGAGTAAGCAAATTCAAATATCTTTTAAAAAAAAAAAAAAATCAAGAGAAAAA-CAA : 1231
CM334_CLV3  : TGACATGACCACAACTAAAATCATGAAGTACATAATATGAGAGTAAGCAAATTCAAATATCTTTTAAAAAAAAAAAAAAATCAAGAGAAAAA-CAA : 1231
              TGACATGACCACAACTAAAATCATGAAGTACATAATATGAGAGTAAGCAAATTCAAATATCTTTTAAAAAAAAAAAAAAATCAAGAGAAAAA CAA

                 1240         *      1260         *      1280         *      1300         *      1320         *
Corno       : ATTCAACGTGTGATATCCAAAGTAAATTTTCTGATGTAATCACTCTAGCATCATCATTTTATTTTGTCTCTTCAAATCAAATAACGTAACTACAT : 1327
Quadrato    : ATTCAACGTGTGATATCCAAAGTAAATTTTCTGATGTAATCACTCTAGCATCATCATTTTATTTTGTCTCTTCAAATCAAATAACGTAACTACAT : 1327
Tumaticot   : ATTCAACGTGTGATATCCAAAGTAAATTTTCTGATGTAATCACTCTAGCATCATCATTTTATTTTGTCTCTTCAAATCAAATAACGTAACTACAT : 1330
Cuneo       : ATTCAACGTGTGATATCCAAAGTAAATTTTCTGATGTAATCACTCTAGCATCATCATTTTATTTTGTCTCTTCAAATCAAATAACGTAACTACAT : 1330
Perennial   : ATTCAACGTGTGATATCCAAAGTAAATTTTCTGATGTAATCACTCTAGCATCATCATTTTATTTTGTCTCTTCAAATCAAATAACGTAACTACAT : 1326
CM334_CLV3  : ATTCAACGTGTGATATCCAAAGTAAATTTTCTGATGTAATCACTCTAGCATCATCATTTTATTTTGTCTCTTCAAATCAAATAACGTAACTACAT : 1326
              ATTCAACGTGTGATATCCAAAGTAAATTTTCTGATGTAATCACTCTAGCATCATCATTTTATTTTGTCTCTTCAAATCAAATAACGTAACTACAT

                      1340         *      1360         *      1380         *      1400         *      1420
Corno       : ACATTATATTATATAAATGGAGGAAAAAGATTGATAAAAGAAAAATAATTTATTAGTACACACTAATAACAATGAAGAGGAAAAAATTAAAGCAA : 1422
Quadrato    : ACATTATATTATATAAATGGAGGAAAAAGATTGATAAAAGAAAAATAATTTATTAGTACACACTAATAACAATGAAGAGGAAAAAATTAAAGCAA : 1422
Tumaticot   : ACATTATATTATATAAATGGAGGAAAAAGATTGATAAAAGAAAAATAATTTATTAGTACACACTAATAACAATGAAGAGGAAAAAATTAAAGCAA : 1425
Cuneo       : ACATTATATTATATAAATGGAGGAAAAAGATTGATAAAAGAAAAATAATTTATTAGTACACACTAATAACAATGAAGAGGAAAAAATTAAAGCAA : 1425
Perennial   : ACATTATATTATATAAATGGAGGAAAAAGATTGATAAAAGAAAAATAATTTATTAGTACACACTAATAACAATGAAGAGGAAAAAATTAAAGCAA : 1421
CM334_CLV3  : ACATTATATTATATAAATGGAGGAAAAAGATTGATAAAAGAAAAATAATTTATTAGTACACACTAATAACAATGAAGAGGAAAAAATTAAAGCAA : 1421
              ACATTATATTATATAAATGGAGGAAAAAGATTGATAAAAGAAAAATAATTTATTAGTACACACTAATAACAATGAAGAGGAAAAAATTAAAGCAA

                      *      1440         *      1460         *      1480         *      1500         *      1520
Corno       : AATATATATGTTGTGACTTGTGAGAGGATTATGTCAATTATCCAGTGGACAAATGCATACTCCTATAACCTACTTATTTTATTTTCAGAAAAAAG : 1517
Quadrato    : AATATATATGTTGTGACTTGTGAGAGGATTATGTCAATTATCCAGTGGACAAATGCATACTCCTATAACCTACTTATTTTATTTTCAGAAAAAAG : 1517
Tumaticot   : AATATATATGTTGTGACTTGTGAGAGGATTATGTCAATTATCCAGTGGACAAATGCATACTCCTATAACCTACTTATTTTATTTTCAGAAAAAAG : 1520
Cuneo       : AATATATATGTTGTGACTTGTGAGAGGATTATGTCAATTATCCAGTGGACAAATGCATACTCCTATAACCTACTTATTTTATTTTCAGAAAAAAG : 1520
Perennial   : AATATATATGTTGTGACTTGTGAGAGGATTACGTCAATTATCCAGTGGACAAATGCATACTCCTATAACCTACTTATTTTATTTTCAGAAAAAAG : 1516
CM334_CLV3  : AATATATATGTTGTGACTTGTGAGAGGATTACGTCAATTATCCAGTGGACAAATGCATACTCCTATAACCTACTTATTTTATTTTCAGAAAAAAG : 1516
              AATATATATGTTGTGACTTGTGAGAGGATTA GTCAATTATCCAGTGGACAAATGCATACTCCTATAACCTACTTATTTTATTTTCAGAAAAAAG

                      *      1540         *      1560         *      1580         *      1600         *
Corno       : AATGTGATGGTTTCAAGATTAGTACAATTATTTATGATCAGTGCAGTGCAGTAGTA-GAACCTGATGAGGATCGACCAATAAATTGGAAGAGGAA : 1611
Quadrato    : AATGTGATGGTTTCAAGATTAGTACAATTATTTATGATCAGTGCAGTGCAGTAGTA-GAACCTGATGAGGATCGACCAATAAATTGGAAGAGGAA : 1611
Tumaticot   : AATGTGATGGTTTCAAGATTAGTACAATTATTTATGATCAGTGCAGTGCAGTAGTA-GAACCTGATGAGGATCGACCAATAAATTGGAAGAGGAA : 1614
Cuneo       : AATGTGATGGTTTCAAGATTAGTACAATTATTTATGATCAGTGCAGTGCAGTAGTA-GAACCTGATGAGGATCGACCAATAAATTGGAAGAGGAA : 1614
Perennial   : AATGTGATGGTTTCAAGATTAGTACAATTATTTATGATCAGTGCAGTGCAGTAGTACGAACCTGATTAGGATCGACCAATAAATTGGAAGAGGAA : 1611
CM334_CLV3  : AATGTGATGGTTTCAAGATTAGTACAATTATTTATGATCAGTGCAGTGCAGTAGTACGAACCTGATTAGGATCGACCAATAAATTGGAAGAGGAA : 1611
              AATGTGATGGTTTCAAGATTAGTACAATTATTTATGATCAGTGCAGTGCAGTAGTA GAACCTGAT AGGATCGACCAATAAATTGGAAGAGGAA

                 1620         *      1640         *      1660         *      1680         *      1700         *
Corno       : ATAAAAGGAAAATGATAACCAAATTATGAGGAATAATTAACGAACTTGTTAAATGGTGGACTAATAATTATTGGAATCCTTAAAAAAATATTATA : 1706
Quadrato    : ATAAAAGGAAAATGATAACCAAATTATGAGGAATAATTAACGAACTTGTTAAATGGTGGACTAATAATTATTGGAATCCTTAAAAAAATATTATA : 1706
Tumaticot   : ATAAAAGGAAAATGATAACCAAATTATGAGGAATAATTAACGAACTTGTTAAATGGTGGACTAATAATTATTGGAATCCTTAAAAAAATATTATA : 1709
Cuneo       : ATAAAAGGAAAATGATAACCAAATTATGAGGAATAATTAACGAACTTGTTAAATGGTGGACTAATAATTATTGGAATCCTTAAAAAAATATTATA : 1709
Perennial   : ATAAAAGGAAAATGATCACCAAATTATGAGGAATAATTAACGAACTTGTTAAATGGTGGACTAATAATTAGTTGAATCCTTAAAAAAATATTATA : 1706
CM334_CLV3  : ATAAAAGGAAAATGATCACCAAATTATGAGGAATAATTAACGAACTTGTTAAATGGTGGACTAATAATTAGTTGAATCCTTAAAAAAATATTATA : 1706
              ATAAAAGGAAAATGAT ACCAAATTATGAGGAATAATTAACGAACTTGTTAAATGGTGGACTAATAATTA T GAATCCTTAAAAAAATATTATA

                      1720         *      1740         *      1760         *      1780         *      1800
Corno       : TTCACAAGAAACCCTAGTCCTATCCTATGTATAGAGATCTAGTTCTATAAATATTAAATTAAATATTTAATTTAAAGCGTGAGTATTATTTAATT : 1801
Quadrato    : TTCACAAGAAACCCTAGTCCTATCCTATGTATAGAGATCTAGTTCTATAAATATTAAATTAAATATTTAATTTAAAGCGTGAGTATTATTTAATT : 1801
Tumaticot   : TTCACAAGAAACCCTAGTCCTATCCTATGTATAGAGATCTAGTTCTATAAATATTAAATTAAATATTTAATTTAAAGCGTGAGTATTATTTAATT : 1804
Cuneo       : TTCACAAGAAACCCTAGTCCTATCCTATGTATAGAGATCTAGTTCTATAAATATTAAATTAAATATTTAATTTAAAGCGTGAGTATTATTTAATT : 1804
Perennial   : TTCACAAGAAACCCTAGTCCTATCCTATGTATAGAGATCTAGTTCTATAAATATTAAATTAAATATTTAATTTAAAGCGTGAGTATTATTTAATT : 1801
CM334_CLV3  : TTCACAAGAAACCCTAGTCCTATCCTATGTATAGAGATCTAGTTCTATAAATATTAAATTAAATATTTAATTTAAAGCGTGAGTATTATTTAATT : 1801
              TTCACAAGAAACCCTAGTCCTATCCTATGTATAGAGATCTAGTTCTATAAATATTAAATTAAATATTTAATTTAAAGCGTGAGTATTATTTAATT

                      *      1820         *      1840         *      1860         *      1880         *      1900
Corno       : TCTGCCTAGGTACTTGTTGGATAGGGTTTGAACAAAATTAACTGGATCTGAACTAAAATCATATTTACTTCCATTAACCTTCAAAAATCAATCAA : 1896
Quadrato    : TCTGCCTAGGTACTTGTTGGATAGGGTTTGAACAAAATTAACTGGATCTGAACTAAAATCATATTTACTTCCATTAACCTTCAAAAATCAATCAA : 1896
Tumaticot   : TCTGCCTAGGTACTTGTTGGATAGGGTTTGAACAAAATTAACTGGATCTGAACTAAAATCATATTTACTTCCATTAACCTTCAAAAATCAATCAA : 1899
Cuneo       : TCTGCCTAGGTACTTGTTGGATAGGGTTTGAACAAAATTAACTGGATCTGAACTAAAATCATATTTACTTCCATTAACCTTCAAAAATCAATCAA : 1899
Perennial   : TCTGCCTAGGTACTTGTTGGATAGGGTTTGAACAAAATTAACTGGATCTGAACTAAAATCATATTTACTTCCATTAACCTTCAAAAATCAATCAA : 1896
CM334_CLV3  : TCTGCCTAGGTACTTGTTGGATAGGGTTTGAACAAAATTAACTGGATCTGAACTAAAATCATATTTACTTCCATTAACCTTCAAAAATCAATCAA : 1896
              TCTGCCTAGGTACTTGTTGGATAGGGTTTGAACAAAATTAACTGGATCTGAACTAAAATCATATTTACTTCCATTAACCTTCAAAAATCAATCAA

                      *      1920         *      1940         *      1960         *      1980         *
Corno       : GGAAAAAAGAGAAAACAGTGAAACACTTTGTTTTAAATTATTTGAAAATTAGCATAAAATATGACAATTTTCTAACACTTTGAAGCAAAATTTCT : 1991
Quadrato    : GGAAAAAAGAGAAAACAGTGAAACACTTTGTTTTAAATTATTTGAAAATTAGCATAAAATATGACAATTTTCTAACACTTTGAAGCAAAATTTCT : 1991
Tumaticot   : GGAAAAAAGAGAAAACAGTGAAACACTTTGTTTTAAATTATTTGAAAATTAGCATAAAATATGACAATTTTCTAACACTTTGAAGCAAAATTTCT : 1994
Cuneo       : GGAAAAAAGAGAAAACAGTGAAACACTTTGTTTTAAATTATTTGAAAATTAGCATAAAATATGACAATTTTCTAACACTTTGAAGCAAAATTTCT : 1994
Perennial   : GGAAAAAAGAGAAAACAGTAAAACACTTTGTTTTAAATTATTTGAAAATTAGCATAAAATATGACAATTTTCTAACACTTTGAAGCAAAATTTCT : 1991
CM334_CLV3  : GGAAAAAAGAGAAAACAGTAAAACACTTTGTTTTAAATTATTTGAAAATTAGCATAAAATATGACAATTTTCTAACACTTTGAAGCAAAATTTCT : 1991
              GGAAAAAAGAGAAAACAGT AAACACTTTGTTTTAAATTATTTGAAAATTAGCATAAAATATGACAATTTTCTAACACTTTGAAGCAAAATTTCT

                 2000         *      2020         *      2040         *      2060         *      2080         *
Corno       : AGAAACATCACATCTTTACGGTTGATTTATCAAATGGTCACTCAAGTAATATATAGTTATTATCTCAATTTGTTATCTTTCTTTTTTATTACAAT : 2086
Quadrato    : AGAAACATCACATCTTTACGGTTGATTTATCAAATGGTCACTCAAGTAATATATAGTTATTATCTCAATTTGTTATCTTTCTTTTTTATTACAAT : 2086
Tumaticot   : AGAAACATCACATCTTTACGGTTGATTTATCAAATGGTCACTCAAGTAATATATAGTTATTATCTCAATTTGTTATCTTTCTTTTTTATTACAAT : 2089
Cuneo       : AGAAACATCACATCTTTACGGTTGATTTATCAAATGGTCACTCAAGTAATATATAGTTATTATCTCAATTTGTTATCTTTCTTTTTTATTACAAT : 2089
Perennial   : ACAAACATCACATCTTTACGATTGATTTATCAAATGGTCACTCAAGTAATATATAGTTATTATCTCAATTTGTTATCTTTCTTTTTTATTACAAT : 2086
CM334_CLV3  : ACAAACATCACATCTTTACGATTGATTTATCAAATGGTCACTCAAGTAATATATAGTTATTATCTCAATTTGTTATCTTTCTTTTTTATTACAAT : 2086
              A AAACATCACATCTTTACG TTGATTTATCAAATGGTCACTCAAGTAATATATAGTTATTATCTCAATTTGTTATCTTTCTTTTTTATTACAAT
```

```
                     2100         *       2120         *       2140         *       2160         *       2180
Corno        : CTTTTCCAACAAAGAAAACAATTTTCCGAGAAAATAACTACTATTAATTGAATGACCATCTGAAAAATCATCTCGGCATCTTTCTAACCCTATCT : 2181
Quadrato     : CTTTTCCAACAAAGAAAACAATTTTCCGAGAAAATAACTACTATTAATTGAATGACCATCTGAAAAATCATCTCGGCATCTTTCTAACCCTATCT : 2181
Tumaticot    : CTTTTCCAACAAAGAAAACAATTTTCCGAGAAAATAACTACTATTAATTGAATGACCATCTGAAAAATCATCTCGGCATCTTTCTAACCCTATCT : 2184
Cuneo        : CTTTTCCAACAAAGAAAACAATTTTCCGAGAAAATAACTACTATTAATTGAATGACCATCTGAAAAATCATCTCGGCATCTTTCTAACCCTATCT : 2184
Perennial    : CTTTTCCAACAAAGAAAACAATTTTCCGAGAAAATAACTACTATTAGTTGAATGACCATCTGAAAAATCATCTCGGCATCTTTCTAACCCTATCT : 2181
CM334_CLV3   : CTTTTCCAACAAAGAAAACAATTTTCCGAGAAAATAACTACTATTAGTTGAATGACCATCTGAAAAATCATCTCGGCATCTTTCTAACCCTATCT : 2181
               CTTTTCCAACAAAGAAAACAATTTTCCGAGAAAATAACTACTATTA TTGAATGACCATCTGAAAAATCATCTCGGCATCTTTCTAACCCTATCT

                *       2200         *       2220         *       2240         *       2260         *       2280
Corno        : TATGGCTTCTACTAAAAAGAAAAAGAAGACTTCGGTTTAATTTCACAAGATTTGCTTTCAAAAAATCTAGAAAAAAGTGGAACAAAATTAAACTT : 2276
Quadrato     : TATGGCTTCTACTAAAAAGAAAAAGAAGACTTCGGTTTAATTTCACAAGATTTGCTTTCAAAAAATCTAGAAAAAAGTGGAACAAAATTAAACTT : 2276
Tumaticot    : TATGGCTTCTACTAAAAAGAAAAAGAAGACTTCGGTTTAATTTCACAAGATTTGCTTTCAAAAAATCTAGAAAAAAGTGGAACAAAATTAAACTT : 2279
Cuneo        : TATGGCTTCTACTAAAAAGAAAAAGAAGACTTCGGTTTAATTTCACAAGATTTGCTTTCAAAAAATCTAGAAAAAAGTGGAACAAAATTAAACTT : 2279
Perennial    : TATGGCTTGTACTAAAAAGAAAAAGAAGACTTCGGTTTAATTTCACAAGATTTGCTTTCAAAAAATCTAGAAAAAAGTGGAACAAAATTAAACTT : 2276
CM334_CLV3   : TATGGCTTGTACTAAAAAGAAAAAGAAGACTTCGGTTTAATTTCACAAGATTTGCTTTCAAAAAATCTAGAAAAAAGTGGAACAAAATTAAACTT : 2276
               TATGGCTT TACTAAAAAGAAAAAGAAGACTTCGGTTTAATTTCACAAGATTTGCTTTCAAAAAATCTAGAAAAAAGTGGAACAAAATTAAACTT

                        *       2300         *       2320         *       2340         *       2360         *
Corno        : TGAACTTGTATTCATCATCATAGACAAAATGAGAGCTCAGAAAAATGGCTCTTAATTTCTTCAAACGATTCGATCATGCATTTGAAAACCAGCTT : 2371
Quadrato     : TGAACTTGTATTCATCATCATAGACAAAATGAGAGCTCAGAAAAATGGCTCTTAATTTCTTCAAACGATTCGATCATGCATTTGAAAACCAGCTT : 2371
Tumaticot    : TGAACTTGTATTCATCATCATAGACAAAATGAGAGCTCAGAAAAATGGCTCTTAATTTCTTCAAACGATTCGATCATGCATTTGAAAACCAGCTT : 2374
Cuneo        : TGAACTTGTATTCATCATCATAGACAAAATGAGAGCTCAGAAAAATGGCTCTTAATTTCTTCAAACGATTCGATCATGCATTTGAAAACCAGCTT : 2374
Perennial    : TGAACTTGTATTCATCATCATAGACAAAATGAGAGCTCAGAAAAATGGCTCTTAATTTCTTCAAACGATTCGATCATGCATTTGAAAACCAGCTT : 2371
CM334_CLV3   : TGAACTTGTATTCATCATCATAGACAAAATGAGAGCTCAGAAAAATGGCTCTTAATTTCTTCAAACGATTCGATCATGCATTTGAAAACCAGCTT : 2371
               TGAACTTGTATTCATCATCATAGACAAAATGAGAGCTCAGAAAAATGGCTCTTAATTTCTTCAAACGATTCGATCATGCATTTGAAAACCAGCTT

                2380         *       2400         *       2420         *       2440         *       2460         *
Corno        : AGCATATATAAAGGGTTTTAAAATTTTTGAAGGAGATGGATGAAAAAATTAAAATAAAAGAAGTGTACCGCAAGAATTGTGTTGCAGAAGTTGCA : 2466
Quadrato     : AGCATATATAAAGGGTTTTAAAATTTTTGAAGGAGATGGATGAAAAAATTAAAATAAAAGAAGTGTACCGCAAGAATTGTGTTGCAGAAGTTGCA : 2466
Tumaticot    : AGCATATATAAAGGGTTTTAAAATTTTTGAAGGAGATGGATGAAAAAATTAAAATAAAAGAAGTGTACCGCAAGAATTGTGTTGCAGAAGTTGCA : 2469
Cuneo        : AGCATATATAAAGGGTTTTAAAATTTTTGAAGGAGATGGATGAAAAAATTAAAATAAAAGAAGTGTACCGCAAGAATTGTGTTGCAGAAGTTGCA : 2469
Perennial    : AGCATATATAAAGGGTTTTAAAATTTTTGAAGGAGATGGATGAAAAAATTAAAATAAAAGAAGTGTACCGCAAGAATTGTGTTGCAGAAGTTGCA : 2466
CM334_CLV3   : AGCATATATAAAGGGTTTTAAAATTTTTGAAGGAGATGGATGAAAAAATTAAAATAAAAGAAGTGTACCGCAAGAATTGTGTTGCAGAAGTTGCA : 2466
               AGCATATATAAAGGGTTTTAAAATTTTTGAAGGAGATGGATGAAAAAATTAAAATAAAAGAAGTGTACCGCAAGAATTGTGTTGCAGAAGTTGCA

                     2480         *       2500         *       2520         *       2540         *       2560
Corno        : ATGAACATAGCAAACACGTTCCAAGGAAGATGAATATGTCATGTCTAGTGACATTTTTCTTAAAGGTGTTTTTCTTTTCTTTTTCTTTTTTTAGC : 2561
Quadrato     : ATGAACATAGCAAACACGTTCCAAGGAAGATGAATATGTCATGTCTAGTGACATTTTTCTTAAAGGTGTTTTTCTTTTCTTTTTCTTTTTTTAGC : 2561
Tumaticot    : ATGAACATAGCAAACACGTTCCAAGGAAGATGAATATGTCATGTCTAGTGACATTTTTCTTAAAGGTGTTTTTCTTTTCTTTTTCTTTTTTTAGC : 2564
Cuneo        : ATGAACATAGCAAACACGTTCCAAGGAAGATGAATATGTCATGTCTAGTGACATTTTTCTTAAAGGTGTTTTTCTTTTCTTTTTCTTTTTTTAGC : 2564
Perennial    : ATGAACATAGCAAACACGTTCCAAGGAAGATGAATATGTCATGTCTAGTGACATTTTTCTTAAAGGTGTTTTTCTTTTCTTTTTCTTTTTTTAGC : 2561
CM334_CLV3   : ATGAACATAGCAAACACGTTCCAAGGAAGATGAATATGTCATGTCTAGTGACATTTTTCTTAAAGGTGTTTTTCTTTTCTTTTTCTTTTTTTAGC : 2561
               ATGAACATAGCAAACACGTTCCAAGGAAGATGAATATGTCATGTCTAGTGACATTTTTCTTAAAGGTGTTTTTCTTTTCTTTTTCTTTTTTTAGC

                *       2580         *       2600         *       2620         *       2640         *       2660
Corno        : CCTGGAATCACGGATCGAAGAAGAGACGGATCAACAAGGAGCTGTGAACTGANNNNNNNNNNNNNNNNNNNNNNNNNNNNNNNNNNNNNNNNNNN : 2656
Quadrato     : CCTGGAATCACGGATCGAAGAAGAGACGGATCAACAAGGAGCTGTGAACTGANNNNNNNNNNNNNNNNNNNNNNNNNNNNNNNNNNNNNNNNNNN : 2656
Tumaticot    : CCTGGAATCACGGATCGAAGAAGAGACGGATCAACAAGGAGCTGTGAACTGANNNNNNNNNNNNNNNNNNNNNNNNNNNNNNNNNNNNNNNNNNN : 2659
Cuneo        : CCTGGAATCACGGATCGAAGAAGAGACGGATCAACAAGGAGCTGTGAACTGANNNNNNNNNNNNNNNNNNNNNNNNNNNNNNNNNNNNNNNNNNN : 2659
Perennial    : CCTGGAATCACGGATCGAAGAAGAGACGGATCAACAAGGAGCTGTGAACTGANNNNNNNNNNNNNNNNNNNNNNNNNNNNNNNNNNNNNNNNNNN : 2656
CM334_CLV3   : CCTGGAATCACGGATCGAAGAAGAGACGGATCAACAAGGAGCTGTGAACTGANNNNNNNNNNNNNNNNNNNNNNNNNNNNNNNNNNNNNNNNNNN : 2656
               CCTGGAATCACGGATCGAAGAAGAGACGGATCAACAAGGAGCTGTGAACTGANNNNNNNNNNNNNNNNNNNNNNNNNNNNNNNNNNNNNNNNNNN

                        *       2680         *       2700         *       2720         *       2740         *
Corno        : NNNNNNNNNNNNNNNNNNNNNNNNNNNNNNNNNNNNNNNNNNNNNNNNNNNNNNNNNNNNNNNNNNNNNNNNNNNNNNNNNNNNNNNNNNNNNNN : 2751
Quadrato     : NNNNNNNNNNNNNNNNNNNNNNNNNNNNNNNNNNNNNNNNNNNNNNNNNNNNNNNNNNNNNNNNNNNNNNNNNNNNNNNNNNNNNNNNNNNNNNN : 2751
Tumaticot    : NNNNNNNNNNNNNNNNNNNNNNNNNNNNNNNNNNNNNNNNNNNNNNNNNNNNNNNNNNNNNNNNNNNNNNNNNNNNNNNNNNNNNNNNNNNNNNN : 2754
Cuneo        : NNNNNNNNNNNNNNNNNNNNNNNNNNNNNNNNNNNNNNNNNNNNNNNNNNNNNNNNNNNNNNNNNNNNNNNNNNNNNNNNNNNNNNNNNNNNNNN : 2754
Perennial    : NNNNNNNNNNNNNNNNNNNNNNNNNNNNNNNNNNNNNNNNNNNNNNNNNNNNNNNNNNNNNNNNNNNNNNNNNNNNNNNNNNNNNNNNNNNNNNN : 2751
CM334_CLV3   : NNNNNNNNNNNNNNNNNNNNNNNNNNNNNNNNNNNNNNNNNNNNNNNNNNNNNNNNNNNNNNNNNNNNNNNNNNNNNNNNNNNNNNNNNNNNNNN : 2751
               NNNNNNNNNNNNNNNNNNNNNNNNNNNNNNNNNNNNNNNNNNNNNNNNNNNNNNNNNNNNNNNNNNNNNNNNNNNNNNNNNNNNNNNNNNNNNNN

                2760         *       2780         *       2800         *       2820         *       2840         *
Corno        : NNNNNNNNNNNNNNNNNNNNNNNNNNNNNNNNNNNNNNNNNNNNNNNNNNNNNNNNNNNNNNNNNNNNNNNNNNNNNNNNNNNNNNNNNNNNNNN : 2846
Quadrato     : NNNNNNNNNNNNNNNNNNNNNNNNNNNNNNNNNNNNNNNNNNNNNNNNNNNNNNNNNNNNNNNNNNNNNNNNNNNNNNNNNNNNNNNNNNNNNNN : 2846
Tumaticot    : NNNNNNNNNNNNNNNNNNNNNNNNNNNNNNNNNNNNNNNNNNNNNNNNNNNNNNNNNNNNNNNNNNNNNNNNNNNNNNNNNNNNNNNNNNNNNNN : 2849
Cuneo        : NNNNNNNNNNNNNNNNNNNNNNNNNNNNNNNNNNNNNNNNNNNNNNNNNNNNNNNNNNNNNNNNNNNNNNNNNNNNNNNNNNNNNNNNNNNNNNN : 2849
Perennial    : NNNNNNNNNNNNNNNNNNNNNNNNNNNNNNNNNNNNNNNNNNNNNNNNNNNNNNNNNNNNNNNNNNNNNNNNNNNNNNNNNNNNNNNNNNNNNNN : 2846
CM334_CLV3   : NNNNNNNNNNNNNNNNNNNNNNNNNNNNNNNNNNNNNNNNNNNNNNNNNNNNNNNNNNNNNNNNNNNNNNNNNNNNNNNNNNNNNNNNNNNNNNN : 2846
               NNNNNNNNNNNNNNNNNNNNNNNNNNNNNNNNNNNNNNNNNNNNNNNNNNNNNNNNNNNNNNNNNNNNNNNNNNNNNNNNNNNNNNNNNNNNNNN

                     2860         *       2880         *       2900         *       2920         *       2940
Corno        : NNNNNNNNNNNNNNNNNNNNNNNNNNNNNNNNNNNNNNNNNNNNNNNNNNNNNNNNNNNNNNNNNNNNNNNNNNNNNNNNNNNNNNNNTGGGGTG : 2941
Quadrato     : NNNNNNNNNNNNNNNNNNNNNNNNNNNNNNNNNNNNNNNNNNNNNNNNNNNNNNNNNNNNNNNNNNNNNNNNNNNNNNNNNNNNNNNNTGGGGTG : 2941
Tumaticot    : NNNNNNNNNNNNNNNNNNNNNNNNNNNNNNNNNNNNNNNNNNNNNNNNNNNNNNNNNNNNNNNNNNNNNNNNNNNNNNNNNNNNNNNNTGGGGTG : 2944
Cuneo        : NNNNNNNNNNNNNNNNNNNNNNNNNNNNNNNNNNNNNNNNNNNNNNNNNNNNNNNNNNNNNNNNNNNNNNNNNNNNNNNNNNNNNNNNTGGGGTG : 2944
Perennial    : NNNNNNNNNNNNNNNNNNNNNNNNNNNNNNNNNNNNNNNNNNNNNNNNNNNNNNNNNNNNNNNNNNNNNNNNNNNNNNNNNNNNNNNNTGGGGTG : 2941
CM334_CLV3   : NNNNNNNNNNNNNNNNNNNNNNNNNNNNNNNNNNNNNNNNNNNNNNNNNNNNNNNNNNNNNNNNNNNNNNNNNNNNNNNNNNNNNNNNTGGGGTG : 2941
               NNNNNNNNNNNNNNNNNNNNNNNNNNNNNNNNNNNNNNNNNNNNNNNNNNNNNNNNNNNNNNNNNNNNNNNNNNNNNNNNNNNNNNNNTGGGGTG

                *       2960         *       2980         *       3000
Corno        : GTAGGGTAGGGTTGAAGGATGAGAAGTGAGTGAAGGAAGTAGTGGGAGTACGGGATCATC : 3001
Quadrato     : GTAGGGTAGGGTTGAAGGATGAGAAGTGAGTGAAGGAAGTAGTGGGAGTACGGGATCATC : 3001
Tumaticot    : GTAGGGTAGGGTTGAAGGATGAGAAGTGAGTGAAGGAAGTAGTGGGAGTACGGGATCATC : 3004
Cuneo        : GTAGGGTAGGGTTGAAGGATGAGAAGTGAGTGAAGGAAGTAGTGGGAGTACGGGATCATC : 3004
Perennial    : GTAGGGTAGGGTTGAAGGATGAGAAGTGAGTGAAGGAAGTAGTGGGAGTACGGGATCATC : 3001
CM334_CLV3   : GTAGGGTAGGGTTGAAGGATGAGAAGTGAGTGAAGGAAGTAGTGGGAGTACGGGATCATC : 3001
               GTAGGGTAGGGTTGAAGGATGAGAAGTGAGTGAAGGAAGTAGTGGGAGTACGGGATCATC
```

OVATE 3kb upstream

```
                      *        20         *        40         *        60         *        80         *
Perennial  : TTCTTTTTTGTCTCTGTGTTTGTTTTTTGGCCCTTTGATAAAGTGTGAAGGTGAGAGTTGTTTATATTGGGGACAAGGGGTGATGGAGAGCGTGA :   95
CM334_OVAT : TTCTTTTTTGTCTCTGTGTTTGTTTTTTGGCCCTTTGATAAAGTGTGAAGGTGAGAGTTGTTTATATTGGGGACAAGGGGTGATGGAGAGCGTGA :   95
Cuneo      : TTCTTTTTTGTCTCTGTGTTTGTTTTTTGGCCCTTTGATAAAGTGTGAAGGTGAGAGTTGTTTATATTGGGGACAAGGGGTGATGGAGAGCGTGA :   95
Corno      : TTCTTTTTTGTCTCTGTGTTTGTTTTTTGGCCCTTTGATAAAGTGTGAAGGTGAGAGTTGTTTATATTGGGGACAAGGGGTGATGGAGAGCGTGA :   95
Quadrato   : TTCTTTTTTGTCTCTGTGTTTGTTTTTTGGCCCTTTGATAAAGTGTGAAGGTGAGAGTTGTTTATATTGGGGACAAGGGGTGATGGAGAGCGTGA :   95
Tumaticot  : TTCTTTTTTGTCTCTGTGTTTGTTTTTTGGCCCTTTGATAAAGTGTGAAGGTGAGAGTTGTTTATATTGGGGACAAGGGGTGATGGAGAGCGTGA :   95
             TTCTTTTTTGTCTCTGTGTTTGTTTTTTGGCCCTTTGATAAAGTGTGAAGGTGAGAGTTGTTTATATTGGGGACAAGGGGTGATGGAGAGCGTGA

                 100         *       120         *       140         *       160         *       180         *
Perennial  : CCCAGAGTCTCTAAAATGTTTTGAGTTATTAGTCAAAGTTAGTAGTAATTGGTTTTACTGATAACGGTTTAATTTCTCCTTTTCACGCTTATTGA :  190
CM334_OVAT : CCCAGAGTCTCTAAAATGTTTTGAGTTATTAGTCAAAGTTAGTAGTAATTGGTTTTACTGATAACGGTTTAATTTCTCCTTTTCACGCTTATTGA :  190
Cuneo      : CCCAGAGTCTCTAAAATGTTTTGAGTTATTAGTCAAAGTTAGTAGTAATTGGTTTTACTGATAACGGTTTAATTTCTCCTTTTCACGCTTATTGA :  190
Corno      : CCCAGAGTCTCTAAAATGTTTTGAGTTATTAGTCAAAGTTAGTAGTAATTGGTTTTACTGATAACGGTTTAATTTCTCCTTTTCACGCTTATTGA :  190
Quadrato   : CCCAGAGTCTCTAAAATGTTTTGAGTTATTAGTCAAAGTTAGTAGTAATTGGTTTTACTGATAACGGTTTAATTTCTCCTTTTCACGCTTATTGA :  190
Tumaticot  : CCCAGAGTCTCTAAAATGTTTTGAGTTATTAGTCAAAGTTAGTAGTAATTGGTTTTACTGATAACGGTTTAATTTCTCCTTTTCACGCTTATTGA :  190
             CCCAGAGTCTCTAAAATGTTTTGAGTTATTAGTCAAAGTTAGTAGTAATTGGTTTTACTGATAACGGTTTAATTTCTCCTTTTCACGCTTATTGA

                   200         *       220         *       240         *       260         *       280
Perennial  : GATAAATCATTGTAATTTTTGAAAGAATTTTAATTTTTTCACATTAGACATTGTCATGTTTCTTGCATTATATAGTTGTTGATAATCGTTAGTAA :  285
CM334_OVAT : GATAAATCATTGTAATTTTTGAAAGAATTTTAATTTTTTCACATTAGACATTGTCATGTTTCTTGCATTATATAGTTGTTGATAATCGTTAGTAA :  285
Cuneo      : GATAAATCATTGTAATTTTTGAAAGAATTTTAATTTTTTCACATTAGACATTGTCATGTTTCTTGCATTATATAGTTGTTGATAATCGTTAGTAA :  285
Corno      : GATAAATCATTGTAATTTTTGAAAGAATTTTAATTTTTTCACATTAGACATTGTCATGTTTCTTGCATTATATAGTTGTTGATAATCGTTAGTAA :  285
Quadrato   : GATAAATCATTGTAATTTTTGAAAGAATTTTAATTTTTTCACATTAGACATTGTCATGTTTCTTGCATTATATAGTTGTTGATAATCGTTAGTAA :  285
Tumaticot  : GATAAATCATTGTAATTTTTGAAAGAATTTTAATTTTTTCACATTAGACATTGTCATGTTTCTTGCATTATATAGTTGTTGATAATCGTTAGTAA :  285
             GATAAATCATTGTAATTTTTGAAAGAATTTTAATTTTTTCACATTAGACATTGTCATGTTTCTTGCATTATATAGTTGTTGATAATCGTTAGTAA

                 *       300         *       320         *       340         *       360         *       380
Perennial  : AGACTAAATTCAAAAAATAAGACAATTTACCTACTACTTAAAATACAATAACATTCTGTAATTATGTGTATAAAGAAAGAATTTTTATTATATTG :  380
CM334_OVAT : AGACTAAATTCAAAAAATAAGACAATTTACCTACTACTTAAAATACAATAACATTCTGTAATTATGTGTATAAAGAAAGAATTTTTATTATATTG :  380
Cuneo      : AGACTAAATTCAAAAAATAAGACAATTTACCTACTACTTAAAATACAATAACATTCTGTAATTATGTGTATAAAGAAAGAATTTTTATTATATTG :  380
Corno      : AGACTAAATTCAAAAAATAAGACAATTTACCTACTACTTAAAATACAATAACATTCTGTAATTATGTGTATAAAGAAAGAATTTTTATTATATTG :  380
Quadrato   : AGACTAAATTCAAAAAATAAGACAATTTACCTACTACTTAAAATACAATAACATTCTGTAATTATGTGTATAAAGAAAGAATTTTTATTATATTG :  380
Tumaticot  : AGACTAAATTCAAAAAATAAGACAATTTACCTACTACTTAAAATACAATAACATTCTGTAATTATGTGTATAAAGAAAGAATTTTTATTATATTG :  380
             AGACTAAATTCAAAAAATAAGACAATTTACCTACTACTTAAAATACAATAACATTCTGTAATTATGTGTATAAAGAAAGAATTTTTATTATATTG

                      *       400         *       420         *       440         *       460         *
Perennial  : GGGATAAAAGAAAGAAAAGAAAAAAGAAGACAGTATAGATACTACTATTAGACTAATGAGCTTTGCTTATTTTTGCATATTAACACTCATACATA :  475
CM334_OVAT : GGGATAAAAGAAAGAAAAGAAAAAAGAAGACAGTATAGATACTACTATTAGACTAATGAGCTTTGCTTATTTTTGCATATTAACACTCATACATA :  475
Cuneo      : GGGATAAAAGAAAGAAAAGAAAAAAGAAGACAGTATAGATACTACTATTAGACTAATGAGCTTTGCTTATTTTTGCATATTAACACTCATACATA :  475
Corno      : GGGATAAAAGAAAGAAAAGAAAAAAGAAGACAGTATAGATACTACTATTAGACTAATGAGCTTTGCTTATTTTTGCATATTAACACTCATACATA :  475
Quadrato   : GGGATAAAAGAAAGAAAAGAAAAAAGAAGACAGTATAGATACTACTATTAGACTAATGAGCTTTGCTTATTTTTGCATATTAACACTCATACATA :  475
Tumaticot  : GGGATAAAAGAAAGAAAAGAAAAAAGAAGACAGTATAGATACTACTATTAGACTAATGAGCTTTGCTTATTTTTGCATATTAACACTCATACATA :  475
             GGGATAAAAGAAAGAAAAGAAAAAAGAAGACAGTATAGATACTACTATTAGACTAATGAGCTTTGCTTATTTTTGCATATTAACACTCATACATA

                 480         *       500         *       520         *       540         *       560         *
Perennial  : TAGTCGATAGTCTATCAAAAACATTTCTATCCTAATTCCGGACAAGGTAAGGTAGTATTGCGTATATATCACTCCTTTCAAAAATCTTTCTTGTA :  570
CM334_OVAT : TAGTCGATAGTCTATCAAAAACATTTCTATCCTAATTCCGGACAAGGTAAGGTAGTATTGCGTATATATCACTCCTTTCAAAAATCTTTCTTGTA :  570
Cuneo      : TAGTCGATAGTCTATCAAAAACATTTCTATCCTAATTCCGGACAAGGTAAGGTAGTATTGCGTATATATCACTCCTTTCAAAAATCTTTCTTGTA :  570
Corno      : TAGTCGATAGTCTATCAAAAACATTTCTATCCTAATTCCGGACAAGGTAAGGTAGTATTGCGTATATATCACTCCTTTCAAAAATCTTTCTTGTA :  570
Quadrato   : TAGTCGATAGTCTATCAAAAACATTTCTATCCTAATTCCGGACAAGGTAAGGTAGTATTGCGTATATATCACTCCTTTCAAAAATCTTTCTTGTA :  570
Tumaticot  : TAGTCGATAGTCTATCAAAAACATTTCTATCCTAATTCCGGACAAGGTAAGGTAGTATTGCGTATATATCACTCCTTTCAAAAATCTTTCTTGTA :  570
             TAGTCGATAGTCTATCAAAAACATTTCTATCCTAATTCCGGACAAGGTAAGGTAGTATTGCGTATATATCACTCCTTTCAAAAATCTTTCTTGTA

                   580         *       600         *       620         *       640         *       660
Perennial  : GGACTATATGTTGTTATTATGTCTAGCTTTTCACATGCGTTTACACTTTGCACAGATCGAGGAGAAGAGAATAGGACAAACTTCAAATTTGGAGT :  665
CM334_OVAT : GGACTATATGTTGTTATTATGTCTAGCTTTTCACATGCGTTTACACTTTGCACAGATCGAGGAGAAGAGAATAGGACAAACTTCAAATTTGGAGT :  665
Cuneo      : GGACTATATGTTGTTATTATGTCTAGCTTTTCACATGCGTTTACACTTTGCACAGATCGAGGAGAAGAGAATAGGACAAACTTCAAATTTGGAGT :  665
Corno      : GGACTATATGTTGTTATTATGTCTAGCTTTTCACATGCGTTTACACTTTGCACAGATCGAGGAGAAGAGAATAGGACAAACTTCAAATTTGGAGT :  665
Quadrato   : GGACTATATGTTGTTATTATGTCTAGCTTTTCACATGCGTTTACACTTTGCACAGATCGAGGAGAAGAGAATAGGACAAACTTCAAATTTGGAGT :  665
Tumaticot  : GGACTATATGTTGTTATTATGTCTAGCTTTTCACATGCGTTTACACTTTGCACAGATCGAGGAGAAGAGAATAGGACAAACTTCAAATTTGGAGT :  665
             GGACTATATGTTGTTATTATGTCTAGCTTTTCACATGCGTTTACACTTTGCACAGATCGAGGAGAAGAGAATAGGACAAACTTCAAATTTGGAGT

                 *       680         *       700         *       720         *       740         *       760
Perennial  : AATAGTCCTCTATTAAATTAGAATGTTGATAGATGGGGATAAAGTTGTAGGCAGTGAGAAACAGGAAGCTGCAGATGCAGTTTCATCTGTTTACT :  760
CM334_OVAT : AATAGTCCTCTATTAAATTAGAATGTTGATAGATGGGGATAAAGTTGTAGGCAGTGAGAAACAGGAAGCTGCAGATGCAGTTTCATCTGTTTACT :  760
Cuneo      : AATAGTCCTCTATTAAATTAGAATGTTGATAGATGGGGATAAAGTTGTAGGCAGTGAGAAACAGGAAGCTGCAGATGCAGTTTCATCTGTTTACT :  760
Corno      : AATAGTCCTCTATTAAATTAGAATGTTGATAGATGGGGATAAAGTTGTAGGCAGTGAGAAACAGGAAGCTGCAGATGCAGTTTCATCTGTTTACT :  760
Quadrato   : AATAGTCCTCTATTAAATTAGAATGTTGATAGATGGGGATAAAGTTGTAGGCAGTGAGAAACAGGAAGCTGCAGATGCAGTTTCATCTGTTTACT :  760
Tumaticot  : AATAGTCCTCTATTAAATTAGAATGTTGATAGATGGGGATAAAGTTGTAGGCAGTGAGAAACAGGAAGCTGCAGATGCAGTTTCATCTGTTTACT :  760
             AATAGTCCTCTATTAAATTAGAATGTTGATAGATGGGGATAAAGTTGTAGGCAGTGAGAAACAGGAAGCTGCAGATGCAGTTTCATCTGTTTACT

                      *       780         *       800         *       820         *       840         *
Perennial  : CTTTACTTGTAGAGAAAACATTTCAATGAAAGGTCTGAAATTAGTTGGCGTCTAGCAGGCAAGAGAATTTAATAAAGTGATTCTATGTATGTATG :  855
CM334_OVAT : CTTTACTTGTAGAGAAAACATTTCAATGAAAGGTCTGAAATTAGTTGGCGTCTAGCAGGCAAGAGAATTTAATAAAGTGATTCTATGTATGTATG :  855
Cuneo      : CTTTACTTGTAGAGAAAACATTTCAATGAAAGGTCTGAAATTAGTTGGCGTCTAGCAGGCAAGAGAATTTAATAAAGTGATTCTATGTATGTATG :  855
Corno      : CTTTACTTGTAGAGAAAACATTTCAATGAAAGGTCTGAAATTAGTTGGCGTCTAGCAGGCAAGAGAATTTAATAAAGTGATTCTATGTATGTATG :  855
Quadrato   : CTTTACTTGTAGAGAAAACATTTCAATGAAAGGTCTGAAATTAGTTGGCGTCTAGCAGGCAAGAGAATTTAATAAAGTGATTCTATGTATGTATG :  855
Tumaticot  : CTTTACTTGTAGAGAAAACATTTCAATGAAAGGTCTGAAATTAGTTGGCGTCTAGCAGGCAAGAGAATTTAATAAAGTGATTCTATGTATGTATG :  855
             CTTTACTTGTAGAGAAAACATTTCAATGAAAGGTCTGAAATTAGTTGGCGTCTAGCAGGCAAGAGAATTTAATAAAGTGATTCTATGTATGTATG

                 860         *       880         *       900         *       920         *       940         *
Perennial  : CATTGTAATGAATTCACTATCCAGGGAAACCAACCGTACCGTACTCTGTTGGATTCCCATTTTTCTTTCCAACTTCTTCATTCCACCATCATGTT :  950
CM334_OVAT : CATTGTAATGAATTCACTATCCAGGGAAACCAACCGTACCGTACTCTGTTGGATTCCCATTTTTCTTTCCAACTTCTTCATTCCACCATCATGTT :  950
Cuneo      : CATTGTAATGAATTCACTATCCAGGGAAACCAACCGTACCGTACTCTGTTGGATTCCCATTTTTCTTTCCAACTTCTTCATTCCACCATCATGTT :  950
Corno      : CATTGTAATGAATTCACTATCCAGGGAAACCAACCGTACCGTACTCTGTTGGATTCCCATTTTTCTTTCCAACTTCTTCATTCCACCATCATGTT :  950
Quadrato   : CATTGTAATGAATTCACTATCCAGGGAAACCAACCGTACCGTACTCTGTTGGATTCCCATTTTTCTTTCCAACTTCTTCATTCCACCATCATGTT :  950
Tumaticot  : CATTGTAATGAATTCACTATCCAGGGAAACCAACCGTACCGTACTCTGTTGGATTCCCATTTTTCTTTCCAACTTCTTCATTCCACCATCATGTT :  950
             CATTGTAATGAATTCACTATCCAGGGAAACCAACCGTACCGTACTCTGTTGGATTCCCATTTTTCTTTCCAACTTCTTCATTCCACCATCATGTT

                   960         *       980         *      1000         *      1020         *      1040
Perennial  : ATAAATCCATCTTTTCCTCTAGTTACTACTTTGGCTTGGTTTGATACCAAGCAAAACGTTTTTTGGAGATAAATGTTTTCCGGGATGTTGATTCA : 1045
CM334_OVAT : ATAAATCCATCTTTTCCTCTAGTTACTACTTTGGCTTGGTTTGATACCAAGCAAAACGTTTTTTGGAGATAAATGTTTTCCGGGATGTTGATTCA : 1045
Cuneo      : ATAAATCCATCTTTTCCTCTAGTTACTACTTTGGCTTGGTTTGATACCAAGCAAAACGTTTTTTGGAGATAAATGTTTTCCGGGATGTTGATTCA : 1045
Corno      : ATAAATCCATCTTTTCCTCTAGTTACTACTTTGGCTTGGTTTGATACCAAGCAAAACGTTTTTTGGAGATAAATGTTTTCCGGGATGTTGATTCA : 1045
Quadrato   : ATAAATCCATCTTTTCCTCTAGTTACTACTTTGGCTTGGTTTGATACCAAGCAAAACGTTTTTTGGAGATAAATGTTTTCCGGGATGTTGATTCA : 1045
Tumaticot  : ATAAATCCATCTTTTCCTCTAGTTACTACTTTGGCTTGGTTTGATACCAAGCAAAACGTTTTTTGGAGATAAATGTTTTCCGGGATGTTGATTCA : 1045
             ATAAATCCATCTTTTCCTCTAGTTACTACTTTGGCTTGGTTTGATACCAAGCAAAACGTTTTTTGGAGATAAATGTTTTCCGGGATGTTGATTCA
```

```
                    *      1060         *      1080         *      1100         *      1120         *      1140
Perennial  : ATCAGAATATCTGTAGGAACGACCGATGAACACTTTTCCAATGGCTAATTCCATGGGAAAATAGTGATTTTTAGTAGTGATATATCCGCAAGTCA : 1140
CM334_OVAT : ATCAGAATATCTGTAGGAACGACCGATGAACACTTTTCCAATGGCTAATTCCATGGGAAAATAGTGATTTTTAGTAGTGATATATCCGCAAGTCA : 1140
Cuneo      : ATCAGAATATCTGTAGGAACGACCGATGAACACTTTTCCAATGGCTAATTCCATGGGAAAATAGTGATTTTTAGTAGTGATATATCCGCAAGTCA : 1140
Corno      : ATCAGAATATCTGTAGGAACGACCGATGAACACTTTTCCAATGGCTAATTCCATGGGAAAATAGTGATTTTTAGTAGTGATATATCCGCAAGTCA : 1140
Quadrato   : ATCAGAATATCTGTAGGAACGACCGATGAACACTTTTCCAATGGCTAATTCCATGGGAAAATAGTGATTTTTAGTAGTGATATATCCGCAAGTCA : 1140
Tumaticot  : ATCAGAATATCTGTAGGAACGACCGATGAACACTTTTCCAATGGCTAATTCCATGGGAAAATAGTGATTTTTAGTAGTGATATATCCGCAAGTCA : 1140
             ATCAGAATATCTGTAGGAACGACCGATGAACACTTTTCCAATGGCTAATTCCATGGGAAAATAGTGATTTTTAGTAGTGATATATCCGCAAGTCA

                        *      1160         *      1180         *      1200         *      1220         *
Perennial  : AATTGTGATGATATGTCGAAACTCCTTAATGAAACTCATTTTATGTAATACTGTTTGACTTGTCACGTTGTTTATGTTAAAAAAAATAAAATTGA : 1235
CM334_OVAT : AATTGTGATGATATGTCGAAACTCCTTAATGAAACTCATTTTATGTAATACTGTTTGACTTGTCACGTTGTTTATGTTAAAAAAAATAAAATTGA : 1235
Cuneo      : AATTGTGATGATATGTCGAAACTCCTTAATGAAACTCATTTTATGTAATACTGTTTGACTTGTCACGTTGTTTATGTTAAAAAAAATAAAATTGA : 1235
Corno      : AATTGTGATGATATGTCGAAACTCCTTAATGAAACTCATTTTATGTAATACTGTTTGACTTGTCACGTTGTTTATGTTAAAAAAAATAAAATTGA : 1235
Quadrato   : AATTGTGATGATATGTCGAAACTCCTTAATGAAACTCATTTTATGTAATACTGTTTGACTTGTCACGTTGTTTATGTTAAAAAAAATAAAATTGA : 1235
Tumaticot  : AATTGTGATGATATGTCGAAACTCCTTAATGAAACTCATTTTATGTAATACTGTTTGACTTGTCACGTTGTTTATGTTAAAAAAAATAAAATTGA : 1235
             AATTGTGATGATATGTCGAAACTCCTTAATGAAACTCATTTTATGTAATACTGTTTGACTTGTCACGTTGTTTATGTTAAAAAAAATAAAATTGA

              1240         *      1260         *      1280         *      1300         *      1320         *
Perennial  : AACTTGTGATCTAAATCAATCCTTGTATATTTATGTGACTGTAAATCATTTCATCAAGTCTAAAAAAGAAATTTTTGAAGTTAAATTGTTTCTAA : 1330
CM334_OVAT : AACTTGTGATCTAAATCAATCCTTGTATATTTATGTGACTGTAAATCATTTCATCAAGTCTAAAAAAGAAATTTTTGAAGTTAAATTGTTTCTAA : 1330
Cuneo      : AACTTGTGATCTAAATCAATCCTTGTATATTTATGTGACTGTAAATCATTTCATCAAGTCTAAAAAAGAAATTTTTGAAGTTAAATTGTTTCTAA : 1330
Corno      : AACTTGTGATCTAAATCAATCCTTGTATATTTATGTGACTGTAAATCATTTCATCAAGTCTAAAAAAGAAATTTTTGAAGTTAAATTGTTTCTAA : 1330
Quadrato   : AACTTGTGATCTAAATCAATCCTTGTATATTTATGTGACTGTAAATCATTTCATCAAGTCTAAAAAAGAAATTTTTGAAGTTAAATTGTTTCTAA : 1330
Tumaticot  : AACTTGTGATCTAAATCAATCCTTGTATATTTATGTGACTGTAAATCATTTCATCAAGTCTAAAAAAGAAATTTTTGAAGTTAAATTGTTTCTAA : 1330
             AACTTGTGATCTAAATCAATCCTTGTATATTTATGTGACTGTAAATCATTTCATCAAGTCTAAAAAAGAAATTTTTGAAGTTAAATTGTTTCTAA

                   1340         *      1360         *      1380         *      1400         *      1420
Perennial  : TTATAATAAGGTGACAATTTTTGGGACGGACGGAAAAGGATATGGTGAAACATAAAATGTGACTGGCTGGAGTATAAAATTCTACATGTACAACT : 1425
CM334_OVAT : TTATAATAAGGTGACAATTTTTGGGACGGACGGAAAAGGATATGGTGAAACATAAAATGTGACTGGCTGGAGTATAAAATTCTACATGTACAACT : 1425
Cuneo      : TTATAATAAGGTGACAATTTTTGGGACGGACGGAAAAGGATATGGTGAAACATAAAATGTGACTGGCTGGAGTATAAAATTCTACATGTACAACT : 1425
Corno      : TTATAATAAGGTGACAATTTTTGGGACGGACGGAAAAGGATATGGTGAAACATAAAATGTGACTGGCTGGAGTATAAAATTCTACATGTACAACT : 1425
Quadrato   : TTATAATAAGGTGACAATTTTTGGGACGGACGGAAAAGGATATGGTGAAACATAAAATGTGACTGGCTGGAGTATAAAATTCTACATGTACAACT : 1425
Tumaticot  : TTATAATAAGGTGACAATTTTTGGGACGGACGGAAAAGGATATGGTGAAACATAAAATGTGACTGGCTGGAGTATAAAATTCTACATGTACAACT : 1425
             TTATAATAAGGTGACAATTTTTGGGACGGACGGAAAAGGATATGGTGAAACATAAAATGTGACTGGCTGGAGTATAAAATTCTACATGTACAACT

                    *      1440         *      1460         *      1480         *      1500         *      1520
Perennial  : AGGGCAAAAATTTACACGCACCCGATGTATACATCAAGCTATTGTTCTTTATCATGATTACGTAATTAAATTAAATAAAATGCATGTTAATTAAT : 1520
CM334_OVAT : AGGGCAAAAATTTACACGCACCCGATGTATACATCAAGCTATTGTTCTTTATCATGATTACGTAATTAAATTAAATAAAATGCATGTTAATTAAT : 1520
Cuneo      : AGGGCAAAAATTTACACGCACCCGATGTATACATCAAGCTATTGTTCTTTATCATGATTACGTAATTAAATTAAATAAAATGCATGTTAATTAAT : 1520
Corno      : AGGGCAAAAATTTACACGCACCCGATGTATACATCAAGCTATTGTTCTTTATCATGATTACGTAATTAAATTAAATAAAATGCATGTTAATTAAT : 1520
Quadrato   : AGGGCAAAAATTTACACGCACCCGATGTATACATCAAGCTATTGTTCTTTATCATGATTACGTAATTAAATTAAATAAAATGCATGTTAATTAAT : 1520
Tumaticot  : AGGGCAAAAATTTACACGCACCCGATGTATACATCAAGCTATTGTTCTTTATCATGATTACGTAATTAAATTAAATAAAATGCATGTTAATTAAT : 1520
             AGGGCAAAAATTTACACGCACCCGATGTATACATCAAGCTATTGTTCTTTATCATGATTACGTAATTAAATTAAATAAAATGCATGTTAATTAAT

                        *      1540         *      1560         *      1580         *      1600         *
Perennial  : CATGATTAAGTAACATGAACTCTAAATTCAAACACATGGCATGTATGTATCGGCTTGATGTATAGATCAGGTGCATGTAAGTTTTAACCTACAAC : 1615
CM334_OVAT : CATGATTAAGTAACATGAACTCTAAATTCAAACACATGGCATGTATGTATCGGCTTGATGTATAGATCAGGTGCATGTAAGTTTTAACCTACAAC : 1615
Cuneo      : CATGATTAAGTAACATGAACTCTAAATTCAAACACATGGCATGTATGTATCGGCTTGATGTATAGATCAGGTGCATGTAAGTTTTAACCTACAAC : 1615
Corno      : CATGATTAAGTAACATGAACTCTAAATTCAAACACATGGCATGTATGTATCGGCTTGATGTATAGATCAGGTGCATGTAAGTTTTAACCTACAAC : 1615
Quadrato   : CATGATTAAGTAACATGAACTCTAAATTCAAACACATGGCATGTATGTATCGGCTTGATGTATAGATCAGGTGCATGTAAGTTTTAACCTACAAC : 1615
Tumaticot  : CATGATTAAGTAACATGAACTCTAAATTCAAACACATGGCATGTATGTATCGGCTTGATGTATAGATCAGGTGCATGTAAGTTTTAACCTACAAC : 1615
             CATGATTAAGTAACATGAACTCTAAATTCAAACACATGGCATGTATGTATCGGCTTGATGTATAGATCAGGTGCATGTAAGTTTTAACCTACAAC

              1620         *      1640         *      1660         *      1680         *      1700         *
Perennial  : TAGTTAGACTTACCCTAGTTAGATGCTTGGTTGAAATCTCGCTAAATATTCTTTTTCTTCTTCTCCCTTTCCCTACTCTTACTAATATATGGATC : 1710
CM334_OVAT : TAGTTAGACTTACCCTAGTTAGATGCTTGGTTGAAATCTCGCTAAATATTCTTTTTCTTCTTCTCCCTTTCCCTACTCTTACTAATATATGGATC : 1710
Cuneo      : TAGTTAGACTTACCCTAGTTAGATGCTTGGTTGAAATCTCGCTAAATATTCTTTTTCTTCTTCTCCCTTTCCCTACTCTTACTAATATATGGATC : 1710
Corno      : TAGTTAGACTTACCCTAGTTAGATGCTTGGTTGAAATCTCGCTAAATATTCTTTTTCTTCTTCTCCCTTTCCCTACTCTTACTAATATATGGATC : 1710
Quadrato   : TAGTTAGACTTACCCTAGTTAGATGCTTGGTTGAAATCTCGCTAAATATTCTTTTTCTTCTTCTCCCTTTCCCTACTCTTACTAATATATGGATC : 1710
Tumaticot  : TAGTTAGACTTACCCTAGTTAGATGCTTGGTTGAAATCTCGCTAAATATTCTTTTTCTTCTTCTCCCTTTCCCTACTCTTACTAATATATGGATC : 1710
             TAGTTAGACTTACCCTAGTTAGATGCTTGGTTGAAATCTCGCTAAATATTCTTTTTCTTCTTCTCCCTTTCCCTACTCTTACTAATATATGGATC

                   1720         *      1740         *      1760         *      1780         *      1800
Perennial  : CACCACAAAATGCAATTAAATGAACAAAGAACTACAAATAGAACCAGAAACATAGACTAATTCCCAAATCAAACATATTAATCCCTATCACTATA : 1805
CM334_OVAT : CACCACAAAATGCAATTAAATGAACAAAGAACTACAAATAGAACCAGAAACATAGACTAATTCCCAAATCAAACATATTAATCCCTATCACTATA : 1805
Cuneo      : CACCACAAAATGCAATTAAATGAACAAAGAACTACAAATAGAACCAGAAACATAGACTAATTCCCAAATCAAACATATTAATCCCTATCACTATA : 1805
Corno      : CACCACAAAATGCAATTAAATGAACAAAGAACTACAAATAGAACCAGAAACATAGACTAATTCCCAAATCAAACATATTAATCCCTATCACTATA : 1805
Quadrato   : CACCACAAAATGCAATTAAATGAACAAAGAACTACAAATAGAACCAGAAACATAGACTAATTCCCAAATCAAACATATTAATCCCTATCACTATA : 1805
Tumaticot  : CACCACAAAATGCAATTAAATGAACAAAGAACTACAAATAGAACCAGAAACATAGACTAATTCCCAAATCAAACATATTAATCCCTATCACTATA : 1805
             CACCACAAAATGCAATTAAATGAACAAAGAACTACAAATAGAACCAGAAACATAGACTAATTCCCAAATCAAACATATTAATCCCTATCACTATA

                    *      1820         *      1840         *      1860         *      1880         *      1900
Perennial  : CAAATAACTTTTTTAATGTTATAAAGCTTTCCGATATTATCTATTATATATATAGGCAAATATAGTGAGACAGACGAGCATTGGTGGCAAAAAAGTA : 1900
CM334_OVAT : CAAATAACTTTTTTAATGTTATAAAGCTTTCCGATATTATCTATTATATATATAGGCAAATATAGTGAGACAGACGAGCATTGGTGGCAAAAAAGTA : 1900
Cuneo      : CAAATAACTTTTTTAATGTTATAAAGCTTTCCGATATTATCTATTATATATATAGGCAAATATAGTGAGACAGACGAGCATTGGTGGCAAAAAAGTA : 1900
Corno      : CAAATAACTTTTTTAATGTTATAAAGCTTTCCGATATTATCTATTATATATATAGGCAAATATAGTGAGACAGACGAGCATTGGTGGCAAAAAAGTA : 1900
Quadrato   : CAAATAACTTTTTTAATGTTATAAAGCTTTCCGATATTATCTATTATATATATAGGCAAATATAGTGAGACAGACGAGCATTGGTGGCAAAAAAGTA : 1900
Tumaticot  : CAAATAACTTTTTTAATGTTATAAAGCTTTCCGATATTATCTATTATATATATAGGCAAATATAGTGAGACAGACGAGCATTGGTGGCAAAAAAGTA : 1900
             CAAATAACTTTTTTAATGTTATAAAGCTTTCCGATATTATCTATTATATATATAGGCAAATATAGTGAGACAGACGAGCATTGGTGGCAAAAAAGTA

                        *      1920         *      1940         *      1960         *      1980         *
Perennial  : CTTTACAAAGAAAAGTAATCTTTTGTCTTGGATCATAATGAAACCATTTGCATATTAGGCCACTTGATAGAAATGAAAAAGAAGGTACTAATCTT : 1995
CM334_OVAT : CTTTACAAAGAAAAGTAATCTTTTGTCTTGGATCATAATGAAACCATTTGCATATTAGGCCACTTGATAGAAATGAAAAAGAAGGTACTAATCTT : 1995
Cuneo      : CTTTACAAAGAAAAGTAATCTTTTGTCTTGGATCATAATGAAACCATTTGCATATTAGGCCACTTGATAGAAATGAAAAAGAAGGTACTAATCTT : 1995
Corno      : CTTTACAAAGAAAAGTAATCTTTTGTCTTGGATCATAATGAAACCATTTGCATATTAGGCCACTTGATAGAAATGAAAAAGAAGGTACTAATCTT : 1995
Quadrato   : CTTTACAAAGAAAAGTAATCTTTTGTCTTGGATCATAATGAAACCATTTGCATATTAGGCCACTTGATAGAAATGAAAAAGAAGGTACTAATCTT : 1995
Tumaticot  : CTTTACAAAGAAAAGTAATCTTTTGTCTTGGATCATAATGAAACCATTTGCATATTAGGCCACTTGATAGAAATGAAAAAGAAGGTACTAATCTT : 1995
             CTTTACAAAGAAAAGTAATCTTTTGTCTTGGATCATAATGAAACCATTTGCATATTAGGCCACTTGATAGAAATGAAAAAGAAGGTACTAATCTT

              2000         *      2020         *      2040         *      2060         *      2080         *
Perennial  : CTTTGTCTTTGGACGACAGCATTTTGAATGATGTGCCTTGTGAAAATTAAAGCCAGTACAAATAAGAAACCTCTCAAAAGATTATAAGGGAATCC : 2090
CM334_OVAT : CTTTGTCTTTGGACGACAGCATTTTGAATGATGTGCCTTGTGAAAATTAAAGCCAGTACAAATAAGAAACCTCTCAAAAGATTATAAGGGAATCC : 2090
Cuneo      : CTTTGTCTTTGGACGACAGCATTTTGAATGATGTGCCTTGTGAAAATTAAAGCCAGTACAAATAAGAAACCTCTCAAAAGATTATAAGGGAATCC : 2090
Corno      : CTTTGTCTTTGGACGACAGCATTTTGAATGATGTGCCTTGTGAAAATTAAAGCCAGTACAAATAAGAAACCTCTCAAAAGATTATAAGGGAATCC : 2090
Quadrato   : CTTTGTCTTTGGACGACAGCATTTTGAATGATGTGCCTTGTGAAAATTAAAGCCAGTACAAATAAGAAACCTCTCAAAAGATTATAAGGGAATCC : 2090
Tumaticot  : CTTTGTCTTTGGACGACAGCATTTTGAATGATGTGCCTTGTGAAAATTAAAGCCAGTACAAATAAGAAACCTCTCAAAAGATTATAAGGGAATCC : 2090
             CTTTGTCTTTGGACGACAGCATTTTGAATGATGTGCCTTGTGAAAATTAAAGCCAGTACAAATAAGAAACCTCTCAAAAGATTATAAGGGAATCC
```

```
                   2100         *       2120         *       2140         *       2160         *       2180
Perennial  : AAGAAAACAAAATCATCCATCCATGGACTTGTCCTTTCTTTTCTCTGTTTTCTCGTTACAAGGACACTTGCTAGCCTATCCGTGTGACGAAGCAA : 2185
CM334_OVAT : AAGAAAACAAAATCATCCATCCATGGACTTGTCCTTTCTTTTCTCTGTTTTCTCGTTACAAGGACACTTGCTAGCCTATCCGTGTGACGAAGCAA : 2185
Cuneo      : AAGAAAACAAAATCATCCATCCATGGACTTGTCCTTTCTTTTCTCTGTTTTCTCGTTACAAGGACACTTGCTAGCCTATCCGTGTGACGAAGCAA : 2185
Corno      : AAGAAAACAAAATCATCCATCCATGGACTTGTCCTTTCTTTTCTCTGTTTTCTCGTTACAAGGACACTTGCTAGCCTATCCGTGTGACGAAGCAA : 2185
Quadrato   : AAGAAAACAAAATCATCCATCCATGGACTTGTCCTTTCTTTTCTCTGTTTTCTCGTTACAAGGACACTTGCTAGCCTATCCGTGTGACGAAGCAA : 2185
Tumaticot  : AAGAAAACAAAATCATCCATCCATGGACTTGTCCTTTCTTTTCTCTGTTTTCTCGTTACAAGGACACTTGCTAGCCTATCCGTGTGACGAAGCAA : 2185
             AAGAAAACAAAATCATCCATCCATGGACTTGTCCTTTCTTTTCTCTGTTTTCTCGTTACAAGGACACTTGCTAGCCTATCCGTGTGACGAAGCAA

                *       2200         *       2220         *       2240         *       2260         *       2280
Perennial  : GGATTTTTTTCATTAAAAATTAATTACAAGCTCAAATCAAAGAAATTGAAATATAGTATGTATACGGAAAATTTAATTTTTTATATGTCTATCTA : 2280
CM334_OVAT : GGATTTTTTTCATTAAAAATTAATTACAAGCTCAAATCAAAGAAATTGAAATATAGTATGTATACGGAAAATTTAATTTTTTATATGTCTATCTA : 2280
Cuneo      : GGATTTTTTTCATTAAAAATTAATTACAAGCTCAAATCAAAGAAATTGAAATATAGTATGTATACGGAAAATTTAATTTTTTATATGTCTATCTA : 2280
Corno      : GGATTTTTTTCATTAAAAATTAATTACAAGCTCAAATCAAAGAAATTGAAATATAGTATGTATACGGAAAATTTAATTTTTTATATGTCTATCTA : 2280
Quadrato   : GGATTTTTTTCATTAAAAATTAATTACAAGCTCAAATCAAAGAAATTGAAATATAGTATGTATACGGAAAATTTAATTTTTTATATGTCTATCTA : 2280
Tumaticot  : GGATTTTTTTCATTAAAAATTAATTACAAGCTCAAATCAAAGAAATTGAAATATAGTATGTATACGGAAAATTTAATTTTTTATATGTCTATCTA : 2280
             GGATTTTTTTCATTAAAAATTAATTACAAGCTCAAATCAAAGAAATTGAAATATAGTATGTATACGGAAAATTTAATTTTTTATATGTCTATCTA

                     *       2300         *       2320         *       2340         *       2360         *
Perennial  : CACAATATAAATTTTGGGAATGTCAATATTAAGCTCTACCGTTGCAGGTGACAAAGTATGAGGGTGTTGTGAAGTACTTGCGTTAGTAAGGAGCA : 2375
CM334_OVAT : CACAATATAAATTTTGGGAATGTCAATATTAAGCTCTACCGTTGCAGGTGACAAAGTATGAGGGTGTTGTGAAGTACTTGCGTTAGTAAGGAGCA : 2375
Cuneo      : CACAATATAAATTTTGGGAATGTCAATATTAAGCTCTACCGTTGCAGGTGACAAAGTATGAGGGTGTTGTGAAGTACTTGCGTTAGTAAGGAGCA : 2375
Corno      : CACAATATAAATTTTGGGAATGTCAATATTAAGCTCTACCGTTGCAGGTGACAAAGTATGAGGGTGTTGTGAAGTACTTGCGTTAGTAAGGAGCA : 2375
Quadrato   : CACAATATAAATTTTGGGAATGTCAATATTAAGCTCTACCGTTGCAGGTGACAAAGTATGAGGGTGTTGTGAAGTACTTGCGTTAGTAAGGAGCA : 2375
Tumaticot  : CACAATATAAATTTTGGGAATGTCAATATTAAGCTCTACCGTTGCAGGTGACAAAGTATGAGGGTGTTGTGAAGTACTTGCGTTAGTAAGGAGCA : 2375
             CACAATATAAATTTTGGGAATGTCAATATTAAGCTCTACCGTTGCAGGTGACAAAGTATGAGGGTGTTGTGAAGTACTTGCGTTAGTAAGGAGCA

              2380         *       2400         *       2420         *       2440         *       2460         *
Perennial  : TAAGTGTGCACAAGTTTATTTACATGAATTTAACTTATATACGTTGATGCTATAAATATCTTTGCATACTTTCAGTTAAATTTAATCTGATGTAT : 2470
CM334_OVAT : TAAGTGTGCACAAGTTTATTTACATGAATTTAACTTATATACGTTGATGCTATAAATATCTTTGCATACTTTCAGTTAAATTTAATCTGATGTAT : 2470
Cuneo      : TAAGTGTGCACAAGTTTATTTACATGAATTTAACTTATATACGTTGATGCTATAAATATCTTTGCATACTTTCAGTTAAATTTAATCTGATGTAT : 2470
Corno      : TAAGTGTGCACAAGTTTATTTACATGAATTTAACTTATATACGTTGATGCTATAAATATCTTTGCATACTTTCAGTTAAATTTAATCTGATGTAT : 2470
Quadrato   : TAAGTGTGCACAAGTTTATTTACATGAATTTAACTTATATACGTTGATGCTATAAATATCTTTGCATACTTTCAGTTAAATTTAATCTGATGTAT : 2470
Tumaticot  : TAAGTGTGCACAAGTTTATTTACATGAATTTAACTTATATACGTTGATGCTATAAATATCTTTGCATACTTTCAGTTAAATTTAATCTGATGTAT : 2470
             TAAGTGTGCACAAGTTTATTTACATGAATTTAACTTATATACGTTGATGCTATAAATATCTTTGCATACTTTCAGTTAAATTTAATCTGATGTAT

                   2480         *       2500         *       2520         *       2540         *       2560
Perennial  : CAAATTAATCCATTTTACTTATTCGAAAACAGATTAAAACAAAGGATGAACTTGTACAGTAGATTCCGAAAACTCTCTCTGTGATATTAAATACA : 2565
CM334_OVAT : CAAATTAATCCATTTTACTTATTCGAAAACAGATTAAAACAAAGGATGAACTTGTACAGTAGATTCCGAAAACTCTCTCTGTGATATTAAATACA : 2565
Cuneo      : CAAATTAATCCATTTTACTTATTCGAAAACAGATTAAAACAAAGGATGAACTTGTACAGTAGATTCCGAAAACTCTCTCTGTGATATTAAATACA : 2565
Corno      : CAAATTAATCCATTTTACTTATTCGAAAACAGATTAAAACAAAGGATGAACTTGTACAGTAGATTCCGAAAACTCTCTCTGTGATATTAAATACA : 2565
Quadrato   : CAAATTAATCCATTTTACTTATTCGAAAACAGATTAAAACAAAGGATGAACTTGTACAGTAGATTCCGAAAACTCTCTCTGTGATATTAAATACA : 2565
Tumaticot  : CAAATTAATCCATTTTACTTATTCGAAAACAGATTAAAACAAAGGATGAACTTGTACAGTAGATTCCGAAAACTCTCTCTGTGATATTAAATACA : 2565
             CAAATTAATCCATTTTACTTATTCGAAAACAGATTAAAACAAAGGATGAACTTGTACAGTAGATTCCGAAAACTCTCTCTGTGATATTAAATACA

                *       2580         *       2600         *       2620         *       2640         *       2660
Perennial  : CTTACTTTTATCAGTTAGGTGAAGTGGACTACTTTTTGGAAACAAAACAAAACAAAAAAGATTTGGAGCCAAAGACAAACTAAAAAACTCTTTCT : 2660
CM334_OVAT : CTTACTTTTATCAGTTAGGTGAAGTGGACTACTTTTTGGAAACAAAACAAAACAAAAAAGATTTGGAGCCAAAGACAAACTAAAAAACTCTTTCT : 2660
Cuneo      : CTTACTTTTATCAGTTAGGTGAAGTGGACTACTTTTTGGAAACAAAACAAAACAAAAAAGATTTGGAGCCAAAGACAAACTAAAAAACTCTTTCT : 2660
Corno      : CTTACTTTTATCAGTTAGGTGAAGTGGACTACTTTTTGGAAACAAAACAAAACAAAAAAGATTTGGAGCCAAAGACAAACTAAAAAACTCTTTCT : 2660
Quadrato   : CTTACTTTTATCAGTTAGGTGAAGTGGACTACTTTTTGGAAACAAAACAAAACAAAAAAGATTTGGAGCCAAAGACAAACTAAAAAACTCTTTCT : 2660
Tumaticot  : CTTACTTTTATCAGTTAGGTGAAGTGGACTACTTTTTGGAAACAAAACAAAACAAAAAAGATTTGGAGCCAAAGACAAACTAAAAAACTCTTTCT : 2660
             CTTACTTTTATCAGTTAGGTGAAGTGGACTACTTTTTGGAAACAAAACAAAACAAAAAAGATTTGGAGCCAAAGACAAACTAAAAAACTCTTTCT

                     *       2680         *       2700         *       2720         *       2740         *
Perennial  : TTTTATTCGTGTTTGATATTTATATTAAAACTTTGATTAGTATACTTAGATTTGCATCGTGTAAGATCAGACTACATACCTACTACGTAGAGTAG : 2755
CM334_OVAT : TTTTATTCGTGTTTGATATTTATATTAAAACTTTGATTAGTATACTTAGATTTGCATCGTGTAAGATCAGACTACATACCTACTACGTAGAGTAG : 2755
Cuneo      : TTTTATTCGTGTTTGATATTTATATTAAAACTTTGATTAGTATACTTAGATTTGCATCGTGTAAGATCAGACTACATACCTACTACGTAGAGTAG : 2755
Corno      : TTTTATTCGTGTTTGATATTTATATTAAAACTTTGATTAGTATACTTAGATTTGCATCGTGTAAGATCAGACTACATACCTACTACGTAGAGTAG : 2755
Quadrato   : TTTTATTCGTGTTTGATATTTATATTAAAACTTTGATTAGTATACTTAGATTTGCATCGTGTAAGATCAGACTACATACCTACTACGTAGAGTAG : 2755
Tumaticot  : TTTTATTCGTGTTTGATATTTATATTAAAACTTTGATTAGTATACTTAGATTTGCATCGTGTAAGATCAGACTACATACCTACTACGTAGAGTAG : 2755
             TTTTATTCGTGTTTGATATTTATATTAAAACTTTGATTAGTATACTTAGATTTGCATCGTGTAAGATCAGACTACATACCTACTACGTAGAGTAG

              2760         *       2780         *       2800         *       2820         *       2840         *
Perennial  : AAATGAGTAGTTAAAATCAACTCCAGTGATCCAATTCATGGGTATGTTATTATGTGTTGGTCCAGATTTCTGAAAGTTTGTGGGAATTGAAATAA : 2850
CM334_OVAT : AAATGAGTAGTTAAAATCAACTCCAGTGATCCAATTCATGGGTATGTTATTATGTGTTGGTCCAGATTTCTGAAAGTTTGTGGGAATTGAAATAA : 2850
Cuneo      : AAATGAGTAGTTAAAATCAACTCCAGTGATCCAATTCATGGGTATGTTATTATGTGTTGGTCCAGATTTCTGAAAGTTTGTGGGAATTGAAATAA : 2850
Corno      : AAATGAGTAGTTAAAATCAACTCCAGTGATCCAATTCATGGGTATGTTATTATGTGTTGGTCCAGATTTCTGAAAGTTTGTGGGAATTGAAATAA : 2850
Quadrato   : AAATGAGTAGTTAAAATCAACTCCAGTGATCCAATTCATGGGTATGTTATTATGTGTTGGTCCAGATTTCTGAAAGTTTGTGGGAATTGAAATAA : 2850
Tumaticot  : AAATGAGTAGTTAAAATCAACTCCAGTGATCCAATTCATGGGTATGTTATTATGTGTTGGTCCAGATTTCTGAAAGTTTGTGGGAATTGAAATAA : 2850
             AAATGAGTAGTTAAAATCAACTCCAGTGATCCAATTCATGGGTATGTTATTATGTGTTGGTCCAGATTTCTGAAAGTTTGTGGGAATTGAAATAA

                   2860         *       2880         *       2900         *       2920         *       2940
Perennial  : AAGGACACTTGCTGAGTATGACATTTTGCCAAACTTTCTTTACATTTCATGGCCATCTAAATATATTTTTTCGTACATCTAGTCTTCTAGATTAC : 2945
CM334_OVAT : AAGGACACTTGCTGAGTATGACATTTTGCCAAACTTTCTTTACATTTCATGGCCATCTAAATATATTTTTTCGTACATCTAGTCTTCTAGATTAC : 2945
Cuneo      : AAGGACACTTGCTGAGTATGACATTTTGCCAAACTTTCTTTACATTTCATGGCCATCTAAATATATTTTTTCGTACATCTAGTCTTCTAGATTAC : 2945
Corno      : AAGGACACTTGCTGAGTATGACATTTTGCCAAACTTTCTTTACATTTCATGGCCATCTAAATATATTTTTTCGTACATCTAGTCTTCTAGATTAC : 2945
Quadrato   : AAGGACACTTGCTGAGTATGACATTTTGCCAAACTTTCTTTACATTTCATGGCCATCTAAATATATTTTTTCGTACATCTAGTCTTCTAGATTAC : 2945
Tumaticot  : AAGGACACTTGCTGAGTATGACATTTTGCCAAACTTTCTTTACATTTCATGGCCATCTAAATATATTTTTTCGTACATCTAGTCTTCTAGATTAC : 2945
             AAGGACACTTGCTGAGTATGACATTTTGCCAAACTTTCTTTACATTTCATGGCCATCTAAATATATTTTTTCGTACATCTAGTCTTCTAGATTAC

                *       2960         *       2980         *       3000
Perennial  : AGAGTACCTCACTCACACTAGGATGAATTATTGGCCAGGAAATTGTTGAAGAGATA : 3001
CM334_OVAT : AGAGTACCTCACTCACACTAGGATGAATTATTGGCCAGGAAATTGTTGAAGAGATA : 3001
Cuneo      : AGAGTACCTCACTCACACTAGGATGAATTATTGGCCAGGAAATTGTTGAAGAGATA : 3001
Corno      : AGAGTACCTCACTCACACTAGGATGAATTATTGGCCAGGAAATTGTTGAAGAGATA : 3001
Quadrato   : AGAGTACCTCACTCACACTAGGATGAATTATTGGCCAGGAAATTGTTGAAGAGATA : 3001
Tumaticot  : AGAGTACCTCACTCACACTAGGATGAATTATTGGCCAGGAAATTGTTGAAGAGATA : 3001
             AGAGTACCTCACTCACACTAGGATGAATTATTGGCCAGGAAATTGTTGAAGAGATA
```

CNR 3kb upstream

```
                     *        20         *        40         *        60         *        80         *
Quadrato  : TAGGCACGCGTTTTGTATTTCCATTCAGAGCTACTACTATTCAAGTGCAGCAGTTGGATCAGTCAAAATAAAGTACACTGGATTTACATCAGATGC :   96
Tumaticot : TAGGCACGCGTTTTGTATTTCCATTCAGAGCTACTACTATTCAAGTGCAGCAGTTGGATCAGTCAAAATAAAGTACACTGGATTTACATCAGATGC :   96
Corno     : TAGGCACGCGTTTTGTATTTCCATTCAGAGCTACTACTATTCAAGTGCAGCAGTTGGATCAGTCAAAATAAAGTACACTGGATTTACATCAGATGC :   96
Cuneo     : TAGGCACGCGTTTTGTATTTCCATTCAGAGCTACTACTATTCAAGTGCAGCAGTTGGATCAGTCAAAATAAAGTACACTGGATTTACATCAGATGC :   96
CM334_CNR : TAGGCACGCGTTTTGTATTTCCATTCAGAGCTACTACTATTCAAGTGCAGCAGTTGGATCAGTCAAAATAAAGTACACTGGATTTACATCAGATGC :   96
Perennial : TAGGCACGCGTTTYGTATTTCCATTCAGAGCTACTACTATTCAAGTGCAGCAGTTGGATCAGTCAAAATAAAGTACACTGGATTTACATCAGATGC :   96
            TAGGCACGCGTTTtGTATTTCCATTCAGAGCTACTACTATTCAAGTGCAGCAGTTGGATCAGTCAAAATAAAGTACACTGGATTTACATCAGATGC

             100         *       120         *       140         *       160         *       180         *
Quadrato  : TGTTCACTATCCTCCCTTACTTTATTTACTGCAACATGCCAAATTGCCAGCAACTTTAGACTTCATTTAATTAGTTCACTTAACCTGTTATATGCT :  192
Tumaticot : TGTTCACTATCCTCCCTTACTTTATTTACTGCAACATGCCAAATTGCCAGCAACTTTAGACTTCATTTAATTAGTTCACTTAACCTGTTATATGCT :  192
Corno     : TGTTCACTATCCTCCCTTACTTTATTTACTGCAACATGCCAAATTGCCAGCAACTTTAGACTTCATTTAATTAGTTCACTTAACCTGTTATATGCT :  192
Cuneo     : TGTTCACTATCCTCCCTTACTTTATTTACTGCAACATGCCAAATTGCCAGCAACTTTAGACTTCATTTAATTAGTTCACTTAACCTGTTATATGCT :  192
CM334_CNR : TGTTCACTATCCTCCCTTACTTTATTTACTGCAACATGCCAAATTGCCAGCAACTTTAGACTTCATTTAATTAGTTCACTTAACCTGTTATATGCT :  192
Perennial : TGTTCACTATCCTCCCTTACTTTATTTACTGCAACATGCCAAATTGCCAGCAACTTTAGACTTCATTTAATTAGTTCACTTAACCTGTTATATGCT :  192
            TGTTCACTATCCTCCCTTACTTTATTTACTGCAACATGCCAAATTGCCAGCAACTTTAGACTTCATTTAATTAGTTCACTTAACCTGTTATATGCT

                 200         *       220         *       240         *       260         *       280
Quadrato  : CGAATTATAGGAGTAGTGATTGTTGATGCGATAGATTTATGATACAAAGGATTGTTACTTGATGATTATATATAAATTTCTGTATAACTTAATATA :  288
Tumaticot : CGAATTATAGGAGTAGTGATTGTTGATGCGATAGATTTATGATACAAAGGATTGTTACTTGATGATTATATATAAATTTCTGTATAACTTAATATA :  288
Corno     : CGAATTATAGGAGTAGTGATTGTTGATGCGATAGATTTATGATACAAAGGATTGTTACTTGATGATTATATATAAATTTCTGTATAACTTAATATA :  288
Cuneo     : CGAATTATAGGAGTAGTGATTGTTGATGCGATAGATTTATGATACAAAGGATTGTTACTTGATGATTATATATAAATTTCTGTATAACTTAATATA :  288
CM334_CNR : CGAATTATAGGAGTAGTGATTGTTGATGCGATAGATTTATGATACAAAGGATTGTTACTTGATGATTATATATAAATTTCTGTATAACTTAATATA :  288
Perennial : CGAATTATAGGAGTAGTGATTGTTGATGCGATAGATTTATGATACAAAGGATTGTTACTTGATGATTATATATAAATTTCTGTATAACTTAATATA :  288
            CGAATTATAGGAGTAGTGATTGTTGATGCGATAGATTTATGATACAAAGGATTGTTACTTGATGATTATATATAAATTTCTGTATAACTTAATATA

             *       300         *       320         *       340         *       360         *       380
Quadrato  : TAGGTATTATTTAATACCGTTAAGCAAACTTTTTTATAAAAAAAAAA-TATAGTAATAGGGGAAAGGAAAATGTGGAAGGGTATTACGAGGTGAGA :  383
Tumaticot : TAGGTATTATTTAATACCGTTAAGCAAACTTTTTTATAAAAAAAAAA-TATAGTAATAGGGGAAAGGAAAATGTGGAAGGGTATTACGAGGTGAGA :  383
Corno     : TAGGTATTATTTAATACCGTTAAGCAAACTTTTTTATAAAAAAAAAA-TATAGTAATAGGGGAAAGGAAAATGTGGAAGGGTATTACGAGGTGAGA :  383
Cuneo     : TAGGTATTATTTAATACCGTTAAGCAAACTTTTTTATAAAAAAAAAA-TATAGTAATAGGGGAAAGGAAAATGTGGAAGGGTATTACGAGGTGAGA :  383
CM334_CNR : TAGGTATTATTTAATACCGTTAAGCAAACTTTTTTATAAAAAAAAAAATATAGTAATAGGGGAAAGGAAAATGTGGAAGGGTATTACGAGGTGAGA :  384
Perennial : TAGGTATTATTTAATACCGTTAAGCAAACTTTTTTATAAAAAAAAAA-TATAGTAATAGGGGAAAGGAAAATGTGGAAGGGTATTACGAGGTGAGA :  383
            TAGGTATTATTTAATACCGTTAAGCAAACTTTTTTATAAAAAAAAAA TATAGTAATAGGGGAAAGGAAAATGTGGAAGGGTATTACGAGGTGAGA

                 *       400         *       420         *       440         *       460         *       480
Quadrato  : AATTGAACCCAACGTTGTTTGTCATGCAACAATTTTTTTTTATGCAGTCAATCAAACATTATACTATTACCTTATGCTGAGTTTTGTTGTAGGATT :  479
Tumaticot : AATTGAACCCAACGTTGTTTGTCATGCAACAATTTTTTTTTATGCAGTCAATCAAACATTATACTATTACCTTATGCTGAGTTTTGTTGTAGGATT :  479
Corno     : AATTGAACCCAACGTTGTTTGTCATGCAACAATTTTTTTTTATGCAGTCAATCAAACATTATACTATTACCTTATGCTGAGTTTTGTTGTAGGATT :  479
Cuneo     : AATTGAACCCAACGTTGTTTGTCATGCAACAATTTTTTTTTATGCAGTCAATCAAACATTATACTATTACCTTATGCTGAGTTTTGTTGTAGGATT :  479
CM334_CNR : AATTGAACCCAACGTTGTTTGTCATGCAACAATTTTTTTTTATGCAGTCAATCAAACATTATACTATTACCTTATGCTGAGTTTTGTTGTAGGATT :  480
Perennial : AATTGAACCCAACGTTGTTTGTCATGCAACAATTTTTTTTTATGCAGTCAATCAAACATTATACTATTACCTTATGCTGAGTTTTGTTGTAGGATT :  479
            AATTGAACCCAACGTTGTTTGTCATGCAACAATTTTTTTTTATGCAGTCAATCAAACATTATACTATTACCTTATGCTGAGTTTTGTTGTAGGATT

                     *       500         *       520         *       540         *       560         *
Quadrato  : AGCTGATCCAATACAAAACCAAACGATCATGCTAAACTAGGGTAATTTTCACTAATGATCATTGAATTTAAAACCTATACTCCCTCTGTCTAAACA :  575
Tumaticot : AGCTGATCCAATACAAAACCAAACGATCATGCTAAACTAGGGTAATTTTCACTAATGATCATTGAATTTAAAACCTATACTCCCTCTGTCTAAACA :  575
Corno     : AGCTGATCCAATACAAAACCAAACGATCATGCTAAACTAGGGTAATTTTCACTAATGATCATTGAATTTAAAACCTATACTCCCTCTGTCTAAACA :  575
Cuneo     : AGCTGATCCAATACAAAACCAAACGATCATGCTAAACTAGGGTAATTTTCACTAATGATCATTGAATTTAAAACCTATACTCCCTCTGTCTAAACA :  575
CM334_CNR : AGCTGATCCAATACAAAACCAAACGATCATGCTAAACTAGGGTAATTTTCACTAATGATCATTGAATTTAAAACCTATACTCCCTCTGTCTAAACA :  576
Perennial : AGCTGATCCAATACAAAACCAAACGATCATGCTAAACTAGGGTAATTTTCACTAATGATCATTGAATTTAAAACCTATACTCCCTCTGTCTAAACA :  575
            AGCTGATCCAATACAAAACCAAACGATCATGCTAAACTAGGGTAATTTTCACTAATGATCATTGAATTTAAAACCTATACTCCCTCTGTCTAAACA

             580         *       600         *       620         *       640         *       660         *
Quadrato  : ACGCGCATAGCGGGAGTTTTAGTGCACAAAGATGCCCTTTTACTTCCATTGTTGGAGTCCCACATCGGTGGGTTAAAGGGTCCTTGGTCTACTTAT :  671
Tumaticot : ACGCGCATAGCGGGAGTTTTAGTGCACAAAGATGCCCTTTTACTTCCATTGTTGGAGTCCCACATCGGTGGGTTAAAGGGTCCTTGGTCTACTTAT :  671
Corno     : ACGCGCATAGCGGGAGTTTTAGTGCACAAAGATGCCCTTTTACTTCCATTGTTGGAGTCCCACATCGGTGGGTTAAAGGGTCCTTGGTCTACTTAT :  671
Cuneo     : ACGCGCATAGCGGGAGTTTTAGTGCACAAAGATGCCCTTTTACTTCCATTGTTGGAGTCCCACATCGGTGGGTTAAAGGGTCCTTGGTCTACTTAT :  671
CM334_CNR : ACGCGCATAGCGGGAGTTTTAGTGCACAAAGATGCCCTTTTACTTCCATTGTTGGAGTCCCACATCGGTGGGTTAAAGGGTCCTTGGTCTACTTAT :  672
Perennial : ACGCGCATAGCGGGAGTTTTAGTGCACAAAGATGCCCTTTTACTTCCATTGTTGGAGTCCCACATCGGTGGGTTAAAGGGTCCTTGGTCTACTTAT :  671
            ACGCGCATAGCGGGAGTTTTAGTGCACAAAGATGCCCTTTTACTTCCATTGTTGGAGTCCCACATCGGTGGGTTAAAGGGTCCTTGGTCTACTTAT

                 680         *       700         *       720         *       740         *       760
Quadrato  : ATGGTCTTGGGCAATCCTCCCCTCATGAGCTAGCTTTTGAGGTTGAGTTAGGCCTAAGGTTCATTTCTTTATCATGATATCAGAGTCAGGCCCACC :  767
Tumaticot : ATGGTCTTGGGCAATCCTCCCCTCATGAGCTAGCTTTTGAGGTTGAGTTAGGCCTAAGGTTCATTTCTTTATCATGATATCAGAGTCAGGCCCACC :  767
Corno     : ATGGTCTTGGGCAATCCTCCCCTCATGAGCTAGCTTTTGAGGTTGAGTTAGGCCTAAGGTTCATTTCTTTATCATGATATCAGAGTCAGGCCCACC :  767
Cuneo     : ATGGTCTTGGGCAATCCTCCCCTCATGAGCTAGCTTTTGAGGTTGAGTTAGGCCTAAGGTTCATTTCTTTATCATGATATCAGAGTCAGGCCCACC :  767
CM334_CNR : ATGGTCTTGGGCAATCCTCCCCTCATGAGCTAGCTTTTGAGGTTGAGTTAGGCCTAAGGTTCATTTCTTTATCATGATATCAGAGTCAGGCCCACC :  768
Perennial : ATGGTCTTGGGCAATCCTCCCCTCATGAGCTAGCTTTTGAGGTTGAGTTAGGCCTAAGGTTCATTTCTTTATCATGATATCAGAGTCAGGCCCACC :  767
            ATGGTCTTGGGCAATCCTCCCCTCATGAGCTAGCTTTTGAGGTTGAGTTAGGCCTAAGGTTCATTTCTTTATCATGATATCAGAGTCAGGCCCACC

             *       780         *       800         *       820         *       840         *       860
Quadrato  : CAGTTCATTGTTTCTCATCTTATATTGTCCACGCTTCAGATACCCAACCCTGGGTGTGCGTGGGGTGTTGTAGTCCCACATCGGTGGGTTTAAAGG :  863
Tumaticot : CAGTTCATTGTTTCTCATCTTATATTGTCCACGCTTCAGATACCCAACCCTGGGTGTGCGTGGGGTGTTGTAGTCCCACATCGGTGGGTTTAAAGG :  863
Corno     : CAGTTCATTGTTTCTCATCTTATATTGTCCACGCTTCAGATACCCAACCCTGGGTGTGCGTGGGGTGTTGTAGTCCCACATCGGTGGGTTTAAAGG :  863
Cuneo     : CAGTTCATTGTTTCTCATCTTATATTGTCCACGCTTCAGATACCCAACCCTGGGTGTGCGTGGGGTGTTGTAGTCCCACATCGGTGGGTTTAAAGG :  863
CM334_CNR : CAGTTCATTGTTTCTCATCTTATATTGTCCACGCTTCAGATACCCAACCCTGGGTGTGCGTGGGGTGTTGTAGTCCCACATCGGTGGGTTTAAAGG :  864
Perennial : CAGTTCATTGTTTCTCATCTTATATTGTCCACGCTTCAGATACCCAACCCTGGGTGTGCGTGGGGTGTTGTAGTCCCACATCGGTGGGTTTAAAGG :  863
            CAGTTCATTGTTTCTCATCTTATATTGTCCACGCTTCAGATACCCAACCCTGGGTGTGCGTGGGGTGTTGTAGTCCCACATCGGTGGGTTTAAAGG

                 *       880         *       900         *       920         *       940         *       960
Quadrato  : TCCTTGATCTCCTTATATTATATGGTCTTGGGCAATAGTTTTTGAGGTTGAGTTAGGGCCAAGGTTATTTCTTTATCATCCTCTATCTCAATTTAT :  959
Tumaticot : TCCTTGATCTCCTTATATTATATGGTCTTGGGCAATAGTTTTTGAGGTTGAGTTAGGGCCAAGGTTATTTCTTTATCATCCTCTATCTCAATTTAT :  959
Corno     : TCCTTGATCTCCTTATATTATATGGTCTTGGGCAATAGTTTTTGAGGTTGAGTTAGGGCCAAGGTTATTTCTTTATCATCCTCTATCTCAATTTAT :  959
Cuneo     : TCCTTGATCTCCTTATATTATATGGTCTTGGGCAATAGTTTTTGAGGTTGAGTTAGGGCCAAGGTTATTTCTTTATCATCCTCTATCTCAATTTAT :  959
CM334_CNR : TCCTTGATCTCCTTATATTATATGGTCTTGGGCAATAGTTTTTGAGGTTGAGTTAGGGCCAAGGTTATTTCTTTATCATCCTCTATCTCAATTTAT :  960
Perennial : TCCTTGATCTCCTTATATTATATGGTCTTGGGCAATAGTTTTTGAGGTTGAGTTAGGGCCAAGGTTATTTCTTTATCATCCTCTATCTCAATTTAT :  959
            TCCTTGATCTCCTTATATTATATGGTCTTGGGCAATAGTTTTTGAGGTTGAGTTAGGGCCAAGGTTATTTCTTTATCATCCTCTATCTCAATTTAT

                     *       980         *      1000         *      1020         *      1040         *
Quadrato  : TTGACACTTTTCTACAATTATAAATTTCTCTTTTACCGGAGTAACACAAAAGTCACAAATTGAAAACAAACTCTAATGTAAATATATATATTTAAC : 1055
Tumaticot : TTGACACTTTTCTACAATTATAAATTTCTCTTTTACCGGAGTAACACAAAAGTCACAAATTGAAAACAAACTCTAATGTAAATATATATATTTAAC : 1055
Corno     : TTGACACTTTTCTACAATTATAAATTTCTCTTTTACCGGAGTAACACAAAAGTCACAAATTGAAAACAAACTCTAATGTAAATATATATATTTAAC : 1055
Cuneo     : TTGACACTTTTCTACAATTATAAATTTCTCTTTTACCGGAGTAACACAAAAGTCACAAATTGAAAACAAACTCTAATGTAAATATATATATTTAAC : 1055
CM334_CNR : TTGACACTTTTCTACAATTATAAATTTCTCTTTTACCGGAGTAACACAAAAGTCACAAATTGAAAACAAACTCTAATGTAAATATATATATTTAAC : 1056
Perennial : TTGACACTTTTCTACAATTATAAATTTCTCTTTTACCGGAGTAACACAAAAGTCACAAATTGAAAACAAACTCTAATGTAAATATATATATTTAAC : 1055
            TTGACACTTTTCTACAATTATAAATTTCTCTTTTACCGGAGTAACACAAAAGTCACAAATTGAAAACAAACTCTAATGTAAATATATATATTTAAC
```

```
                 1060         *       1080         *       1100         *       1120         *       1140         *
Quadrato   : GATCTAGATGAGCTACCTATGAGATCATGAAAAATAATTCCCTGGAAAGAAGCTGTGATTTGCAACATGCCAAGGATTTTGACTTAGTTGCCCATT : 1151
Tumaticot  : GATCTAGATGAGCTACCTATGAGATCATGAAAAATAATTCCCTGGAAAGAAGCTGTGATTTGCAACATGCCAAGGATTTTGACTTAGTTGCCCATT : 1151
Corno      : GATCTAGATGAGCTACCTATGAGATCATGAAAAATAATTCCCTGGAAAGAAGCTGTGATTTGCAACATGCCAAGGATTTTGACTTAGTTGCCCATT : 1151
Cuneo      : GATCTAGATGAGCTACCTATGAGATCATGAAAAATAATTCCCTGGAAAGAAGCTGTGATTTGCAACATGCCAAGGATTTTGACTTAGTTGCCCATT : 1151
CM334_CNR  : GATCTAGATGAGCTACCTATGAGATCATGAAAAATAATTCCCTGGAAAGAAGCTGTGATTTGCAACATGCCAAGGATTTTGACTTAGTTGCCCATT : 1152
Perennial  : GATCTAGATGAGCTACCTATGAGATCATGAAAAATAATTCCCTGGAAAGAAGCTGTGATTTGCAACATGCCAAGGATTTTGACTTAGTTGCCCATT : 1151
             GATCTAGATGAGCTACCTATGAGATCATGAAAAATAATTCCCTGGAAAGAAGCTGTGATTTGCAACATGCCAAGGATTTTGACTTAGTTGCCCATT

                1160         *       1180         *       1200         *       1220         *       1240
Quadrato   : TCCAAGAGAGTAGGCCAGTCAAGTTGTTCCAATCTATAGAAGCTTGTGGACAAGACCTCTCATGAAAACATTAGGCAAATGTTATTTTCCTAAGGA : 1247
Tumaticot  : TCCAAGAGAGTAGGCCAGTCAAGTTGTTCCAATCTATAGAAGCTTGTGGACAAGACCTCTCATGAAAACATTAGGCAAATGTTATTTTCCTAAGGA : 1247
Corno      : TCCAAGAGAGTAGGCCAGTCAAGTTGTTCCAATCTATAGAAGCTTGTGGACAAGACCTCTCATGAAAACATTAGGCAAATGTTATTTTCCTAAGGA : 1247
Cuneo      : TCCAAGAGAGTAGGCCAGTCAAGTTGTTCCAATCTATAGAAGCTTGTGGACAAGACCTCTCATGAAAACATTAGGCAAATGTTATTTTCCTAAGGA : 1247
CM334_CNR  : TCCAAGAGAGTAGGCCAGTCAAGTTGTTCCAATCTATAGAAGCTTGTGGACAAGACCTCTCATGAAAACATTAGGCAAATGTTATTTTCCTAAGGA : 1248
Perennial  : TCCAAGAGAGTAGGCCAGTCAAGTTGTTCCAATCTATAGAAGCTTGTGGACAAGACCTCTCATGAAAACATTAGGCAAATGTTATTTTCCTAAGGA : 1247
             TCCAAGAGAGTAGGCCAGTCAAGTTGTTCCAATCTATAGAAGCTTGTGGACAAGACCTCTCATGAAAACATTAGGCAAATGTTATTTTCCTAAGGA

              *       1260         *       1280         *       1300         *       1320         *       1340
Quadrato   : AAAAATGCTCTTACCTACTCGTTCATGGAACGTTATAAATTGCTCAAATCTATTCATATTTAAAATATTGATGTATGTTAATAAGTTGGAAGAATC : 1343
Tumaticot  : AAAAATGCTCTTACCTACTCGTTCATGGAACGTTATAAATTGCTCAAATCTATTCATATTTAAAATATTGATGTATGTTAATAAGTTGGAAGAATC : 1343
Corno      : AAAAATGCTCTTACCTACTCGTTCATGGAACGTTATAAATTGCTCAAATCTATTCATATTTAAAATATTGATGTATGTTAATAAGTTGGAAGAATC : 1343
Cuneo      : AAAAATGCTCTTACCTACTCGTTCATGGAACGTTATAAATTGCTCAAATCTATTCATATTTAAAATATTGATGTATGTTAATAAGTTGGAAGAATC : 1343
CM334_CNR  : AAAAATGCTCTTACCTACTCGTTCATGGAACGTTATAAATTGCTCAAATCTATTCATATTTAAAATATTGATGTATGTTAATAAGTTGGAAGAATC : 1344
Perennial  : AAAAATGCTCTTACCTACTCGTTCATGGAACGTTATAAATTGCTCAAATCTATTCATATTTAAAATATTGATGTATGTTAATAAGTTGGAAGAATC : 1343
             AAAAATGCTCTTACCTACTCGTTCATGGAACGTTATAAATTGCTCAAATCTATTCATATTTAAAATATTGATGTATGTTAATAAGTTGGAAGAATC

                  *       1360         *       1380         *       1400         *       1420         *       1440
Quadrato   : AAGTGGTAATTACAGTAACCCCCTACTACTACAATTGAATTGGCGAAAGCAATAATAGCACATTACAGATTCCAAATGTAAGTGATCTAGTGATAC : 1439
Tumaticot  : AAGTGGTAATTACAGTAACCCCCTACTACTACAATTGAATTGGCGAAAGCAATAATAGCACATTACAGATTCCAAATGTAAGTGATCTAGTGATAC : 1439
Corno      : AAGTGGTAATTACAGTAACCCCCTACTACTACAATTGAATTGGCGAAAGCAATAATAGCACATTACAGATTCCAAATGTAAGTGATCTAGTGATAC : 1439
Cuneo      : AAGTGGTAATTACAGTAACCCCCTACTACTACAATTGAATTGGCGAAAGCAATAATAGCACATTACAGATTCCAAATGTAAGTGATCTAGTGATAC : 1439
CM334_CNR  : AAGTGGTAATTACAGTAACCCCCTACTACTACAATTGAATTGGCGAAAGCAATAATAGCACATTACAGATTCCAAATGTAAGTGATCTAGTGATAC : 1440
Perennial  : AAGTGGTAATTACAGTAACCCCCTACTACTACAATTGAATTGGCGAAAGCAATAATAGCACATTACAGATTCCAAATGTAAGTGATCTAGTGATAC : 1439
             AAGTGGTAATTACAGTAACCCCCTACTACTACAATTGAATTGGCGAAAGCAATAATAGCACATTACAGATTCCAAATGTAAGTGATCTAGTGATAC

                      *       1460         *       1480         *       1500         *       1520         *
Quadrato   : TAGCTAGCCAGCAGACATTATCATTGAGTACTAATTTGTTTGCGTACCTAAACATGAGCTGATTTTTTTTTACTTAGAAAAGAAAAAATGGATAGC : 1535
Tumaticot  : TAGCTAGCCAGCAGACATTATCATTGAGTACTAATTTGTTTGCGTACCTAAACATGAGCTGATTTTTTTTTACTTAGAAAAGAAAAAATGGATAGC : 1535
Corno      : TAGCTAGCCAGCAGACATTATCATTGAGTACTAATTTGTTTGCGTACCTAAACATGAGCTGATTTTTTTTTACTTAGAAAAGAAAAAATGGATAGC : 1535
Cuneo      : TAGCTAGCCAGCAGACATTATCATTGAGTACTAATTTGTTTGCGTACCTAAACATGAGCTGATTTTTTTTTACTTAGAAAAGAAAAAATGGATAGC : 1535
CM334_CNR  : TAGCTAGCCAGCAGACATTATCATTGAGTACTAATTTGTTTGCGTACCTAAACATGAGCTGATTTTTTTTTACTTAGAAAAGAAAAAATGGATAGC : 1536
Perennial  : TAGCTAGCCAGCAGACATTATCATTGAGTACTAATTTGTTTGCGTACCTAAACATGAGCTGATTTTTTTTTACTTAGAAAAGAAAAAATGGATAGC : 1535
             TAGCTAGCCAGCAGACATTATCATTGAGTACTAATTTGTTTGCGTACCTAAACATGAGCTGATTTTTTTTTACTTAGAAAAGAAAAAATGGATAGC

             1540         *       1560         *       1580         *       1600         *       1620         *
Quadrato   : TATTATTTTCCCACAAATTTCCCTCCACTTAGATATAACTGTAAATTGAAAAACCTAACACCTAATCTTTATTGGAGTTAACTTTCTCATGTGCAA : 1631
Tumaticot  : TATTATTTTCCCACAAATTTCCCTCCACTTAGATATAACTGTAAATTGAAAAACCTAACACCTAATCTTTATTGGAGTTAACTTTCTCATGTGCAA : 1631
Corno      : TATTATTTTCCCACAAATTTCCCTCCACTTAGATATAACTGTAAATTGAAAAACCTAACACCTAATCTTTATTGGAGTTAACTTTCTCATGTGCAA : 1631
Cuneo      : TATTATTTTCCCACAAATTTCCCTCCACTTAGATATAACTGTAAATTGAAAAACCTAACACCTAATCTTTATTGGAGTTAACTTTCTCATGTGCAA : 1631
CM334_CNR  : TATTATTTTCCCACAAATTTCCCTCCACTTAGATATAACTGTAAATTGAAAAACCTAACACCTAATCTTTATTGGAGTTAACTTTCTCATGTGCAA : 1632
Perennial  : TATTATTTTCCCACAAATTTCCCTCCACTTAGATATAACTGTAAATTGAAAAACCTAACACCTAATCTTTATTGGAGTTAACTTTCTCATGTGCAA : 1631
             TATTATTTTCCCACAAATTTCCCTCCACTTAGATATAACTGTAAATTGAAAAACCTAACACCTAATCTTTATTGGAGTTAACTTTCTCATGTGCAA

                1640         *       1660         *       1680         *       1700         *       1720
Quadrato   : ACGTTACGTTATTGGGCGTAGCCCACCATTTTATCCATTTCATTCTTGTCCAGAATTAGCAGCTTTCATGCATTGATGGGCCTCCCACGAGCTGAA : 1727
Tumaticot  : ACGTTACGTTATTGGGCGTAGCCCACCATTTTATCCATTTCATTCTTGTCCAGAATTAGCAGCTTTCATGCATTGATGGGCCTCCCACGAGCTGAA : 1727
Corno      : ACGTTACGTTATTGGGCGTAGCCCACCATTTTATCCATTTCATTCTTGTCCAGAATTAGCAGCTTTCATGCATTGATGGGCCTCCCACGAGCTGAA : 1727
Cuneo      : ACGTTACGTTATTGGGCGTAGCCCACCATTTTATCCATTTCATTCTTGTCCAGAATTAGCAGCTTTCATGCATTGATGGGCCTCCCACGAGCTGAA : 1727
CM334_CNR  : ACGTTACGTTATTGGGCGTAGCCCACCATTTTATCCATTTCATTCTTGTCCAGAATTAGCAGCTTTCATGCATTGATGGGCCTCCCACGAGCTGAA : 1728
Perennial  : ACGTTACGTTATTGGGCGTAGCCCACCATTTTATCCATTTCATTCTTGTCCAGAATTAGCAGCTTTCATGCATTGATGGGCCTCCCACGAGCTGAA : 1727
             ACGTTACGTTATTGGGCGTAGCCCACCATTTTATCCATTTCATTCTTGTCCAGAATTAGCAGCTTTCATGCATTGATGGGCCTCCCACGAGCTGAA

              *       1740         *       1760         *       1780         *       1800         *       1820
Quadrato   : CCATATGTGCAGGCCCTGCAATGTCTCTATGTACAGGAGCCGGGAGCGGAGCTACCCTCGTCCATTTGTGTACCGCTCTATAGACAGATATTTTTA : 1823
Tumaticot  : CCATATGTGCAGGCCCTGCAATGTCTCTATGTACAGGAGCCGGGAGCGGAGCTACCCTCGTCCATTTGTGTACCGCTCTATAGACAGATATTTTTA : 1823
Corno      : CCATATGTGCAGGCCCTGCAATGTCTCTATGTACAGGAGCCGGGAGCGGAGCTACCCTCGTCCATTTGTGTACCGCTCTATAGACAGATATTTTTA : 1823
Cuneo      : CCATATGTGCAGGCCCTGCAATGTCTCTATGTACAGGAGCCGGGAGCGGAGCTACCCTCGTCCATTTGTGTACCGCTCTATAGACAGATATTTTTA : 1823
CM334_CNR  : CCATATGTGCAGGCCCTGCAATGTCTCTATGTACAGGAGCCGGGAGCGGAGCTACCCTCGTCCATTTGTGTACCGCTCTATAGACAGATATTTTTA : 1824
Perennial  : CCATATGTGCAGGCCCTGCAATGTCTCTATGTACAGGAGCCGGGAGCGGAGCTACCCTCGTCCATTTGTGTACCGCTCTATAGACAGATATTTTTA : 1823
             CCATATGTGCAGGCCCTGCAATGTCTCTATGTACAGGAGCCGGGAGCGGAGCTACCCTCGTCCATTTGTGTACCGCTCTATAGACAGATATTTTTA

                  *       1840         *       1860         *       1880         *       1900         *       1920
Quadrato   : TTTATAATGTAAATGTTGAATCCTTCGACTTTTTCGTGGATTTGCATTTTAAAGATACATGCTGTGAAGGGTTATTCTGAAGGAAGTGATGAGGAA : 1919
Tumaticot  : TTTATAATGTAAATGTTGAATCCTTCGACTTTTTCGTGGATTTGCATTTTAAAGATACATGCTGTGAAGGGTTATTCTGAAGGAAGTGATGAGGAA : 1919
Corno      : TTTATAATGTAAATGTTGAATCCTTCGACTTTTTCGTGGATTTGCATTTTAAAGATACATGCTGTGAAGGGTTATTCTGAAGGAAGTGATGAGGAA : 1919
Cuneo      : TTTATAATGTAAATGTTGAATCCTTCGACTTTTTCGTGGATTTGCATTTTAAAGATACATGCTGTGAAGGGTTATTCTGAAGGAAGTGATGAGGAA : 1919
CM334_CNR  : TTTATAATGTAAATGTTGAATCCTTCGACTTTTTCGTGGATTTGCATTTTAAAGATACATGCTGTGAAGGGTTATTCTGAAGGAAGTGATGAGGAA : 1920
Perennial  : TTTATAATGTAAATGTTGAATCCTTCGACTTTTTCGTGGATTTGCATTTTAAAGATACATGCTGTGAAGGGTTATTCTGAAGGAAGTGATGAGGAA : 1919
             TTTATAATGTAAATGTTGAATCCTTCGACTTTTTCGTGGATTTGCATTTTAAAGATACATGCTGTGAAGGGTTATTCTGAAGGAAGTGATGAGGAA

                      *       1940         *       1960         *       1980         *       2000         *
Quadrato   : ATTTCTAAGATGGTACAGTTTCTTTTGCCTCTTGGAAGGTGCGTATTGCTTTGTAATGACTTCGTGTACACCCTTTTATGCTTGGACTAGGTAATT : 2015
Tumaticot  : ATTTCTAAGATGGTACAGTTTCTTTTGCCTCTTGGAAGGTGCGTATTGCTTTGTAATGACTTCGTGTACACCCTTTTATGCTTGGACTAGGTAATT : 2015
Corno      : ATTTCTAAGATGGTACAGTTTCTTTTGCCTCTTGGAAGGTGCGTATTGCTTTGTAATGACTTCGTGTACACCCTTTTATGCTTGGACTAGGTAATT : 2015
Cuneo      : ATTTCTAAGATGGTACAGTTTCTTTTGCCTCTTGGAAGGTGCGTATTGCTTTGTAATGACTTCGTGTACACCCTTTTATGCTTGGACTAGGTAATT : 2015
CM334_CNR  : ATTTCTAAGATGGTACAGTTTCTTTTGCCTCTTGGAAGGTGCGTATTGCTTTGTAATGACTTCGTGTACACCCTTTTATGCTTGGACTAGGTAATT : 2016
Perennial  : ATTTCTAAGATGGTACAGTTTCTTTTGCCTCTTGGAAGGTGCGTATTGCTTTGTAATGACTTCGTGTACACCCTTTTATGCTTGGACTAGGTAATT : 2015
             ATTTCTAAGATGGTACAGTTTCTTTTGCCTCTTGGAAGGTGCGTATTGCTTTGTAATGACTTCGTGTACACCCTTTTATGCTTGGACTAGGTAATT

             2020         *       2040         *       2060         *       2080         *       2100         *
Quadrato   : TTTAAAGCTTGTGAGGGGACTTTTTTTTTGTTATGATAAGATTAAGAAAGAGTAGTTTAAATGATTTAGCAGAAACCCAACGTAAACCCTGTTTCT : 2111
Tumaticot  : TTTAAAGCTTGTGAGGGGACTTTTTTTTTGTTATGATAAGATTAAGAAAGAGTAGTTTAAATGATTTAGCAGAAACCCAACGTAAACCCTGTTTCT : 2111
Corno      : TTTAAAGCTTGTGAGGGGACTTTTTTTTTGTTATGATAAGATTAAGAAAGAGTAGTTTAAATGATTTAGCAGAAACCCAACGTAAACCCTGTTTCT : 2111
Cuneo      : TTTAAAGCTTGTGAGGGGACTTTTTTTTTGTTATGATAAGATTAAGAAAGAGTAGTTTAAATGATTTAGCAGAAACCCAACGTAAACCCTGTTTCT : 2111
CM334_CNR  : TTTAAAGCTTGTGAGGGGACTTTTTTTTTGTTATGATAAGATTAAGAAAGAGTAGTTTAAATGATTTAGCAGAAACCCAACGTAAACCCTGTTTCT : 2112
Perennial  : TTTAAAGCTTGTGAGGGGACTTTTTTTTTGTTATGATAAGATTAAGAAAGAGTAGTTTAAATGATTTAGCAGAAACCCAACGTAAACCCTGTTTCT : 2111
             TTTAAAGCTTGTGAGGGGACTTTTTTTTTGTTATGATAAGATTAAGAAAGAGTAGTTTAAATGATTTAGCAGAAACCCAACGTAAACCCTGTTTCT
```

```
                  2120         *       2140         *       2160         *       2180         *       2200
Quadrato  : CATTTCACGTGGACACCACACCAACTTATATACTTAATTGAGAAGTATCCTTTTAATTTGCTGTATTGAAAGAATAAGGTGCTCAAAATATTTTAC : 2207
Tumaticot : CATTTCACGTGGACACCACACCAACTTATATACTTAATTGAGAAGTATCCTTTTAATTTGCTGTATTGAAAGAATAAGGTGCTCAAAATATTTTAC : 2207
Corno     : CATTTCACGTGGACACCACACCAACTTATATACTTAATTGAGAAGTATCCTTTTAATTTGCTGTATTGAAAGAATAAGGTGCTCAAAATATTTTAC : 2207
Cuneo     : CATTTCACGTGGACACCACACCAACTTATATACTTAATTGAGAAGTATCCTTTTAATTTGCTGTATTGAAAGAATAAGGTGCTCAAAATATTTTAC : 2207
CM334_CNR : CATTTCACGTGGACACCACACCAACTTATATACTTAATTGAGAAGTATCCTTTTAATTTGCTGTATTGAAAGAATAAGGTGCTCAAAATATTTTAC : 2208
Perennial : CATTTCACGTGGACACCACACCAACTTATATACTTAATTGAGAAGTATCCTTTTAATTTGCTGTATTGAAAGAATAAGGTGCTCAAAATATTTTAC : 2207
            CATTTCACGTGGACACCACACCAACTTATATACTTAATTGAGAAGTATCCTTTTAATTTGCTGTATTGAAAGAATAAGGTGCTCAAAATATTTTAC

             *       2220         *       2240         *       2260         *       2280         *       2300
Quadrato  : TGAATTATTGAGCTTGGATTGCGCATTGAAATGCTCAATTCGAACTCACATAAATGTTTATAAATTTAATTTATTTGAAGACTAACCCTTGATAAT : 2303
Tumaticot : TGAATTATTGAGCTTGGATTGCGCATTGAAATGCTCAATTCGAACTCACATAAATGTTTATAAATTTAATTTATTTGAAGACTAACCCTTGATAAT : 2303
Corno     : TGAATTATTGAGCTTGGATTGCGCATTGAAATGCTCAATTCGAACTCACATAAATGTTTATAAATTTAATTTATTTGAAGACTAACCCTTGATAAT : 2303
Cuneo     : TGAATTATTGAGCTTGGATTGCGCATTGAAATGCTCAATTCGAACTCACATAAATGTTTATAAATTTAATTTATTTGAAGACTAACCCTTGATAAT : 2303
CM334_CNR : TGAATTATTGAGCTTGGATTGCGCATTGAAATGCTCAATTCGAACTCACATAAATGTTTATAAATTTAATTTATTTGAAGACTAACCCTTGATAAT : 2304
Perennial : TGAATTATTGAGCTTGGATTGCGCATTGAAATGCTCAATTCGAACTCACATAAATGTTTATAAATTTAATTTATTTGAAGACTAACCCTTGATAAT : 2303
            TGAATTATTGAGCTTGGATTGCGCATTGAAATGCTCAATTCGAACTCACATAAATGTTTATAAATTTAATTTATTTGAAGACTAACCCTTGATAAT

                 *       2320         *       2340         *       2360         *       2380         *       2400
Quadrato  : ACCACCAGAATATTATTGCACGCATTATTTTGTTCAAATTAGCATGCTTAATTTTTACACGGACCGACGCCTTTTATTTCGCCGTTATTTTCCAGA : 2399
Tumaticot : ACCACCAGAATATTATTGCACGCATTATTTTGTTCAAATTAGCATGCTTAATTTTTACACGGACCGACGCCTTTTATTTCGCCGTTATTTTCCAGA : 2399
Corno     : ACCACCAGAATATTATTGCACGCATTATTTTGTTCAAATTAGCATGCTTAATTTTTACACGGACCGACGCCTTTTATTTCGCCGTTATTTTCCAGA : 2399
Cuneo     : ACCACCAGAATATTATTGCACGCATTATTTTGTTCAAATTAGCATGCTTAATTTTTACACGGACCGACGCCTTTTATTTCGCCGTTATTTTCCAGA : 2399
CM334_CNR : ACCACCAGAATATTATTGCACGCATTATTTTGTTCAAATTAGCATGCTTAATTTTTACACGGACCGACGCCTTTTATTTCGCCGTTATTTTCCAGA : 2400
Perennial : ACCACCAGAATATTATTGCACGCATTATTTTGTTCAAATTAGCATGCTTAATTTTTACACGGACCGACGCCTTTTATTTCGCCGTTATTTTCCAGA : 2399
            ACCACCAGAATATTATTGCACGCATTATTTTGTTCAAATTAGCATGCTTAATTTTTACACGGACCGACGCCTTTTATTTCGCCGTTATTTTCCAGA

                     *       2420         *       2440         *       2460         *       2480         *
Quadrato  : AAAGTTGACATTATTTCACACCAGTTGATCCATTTTCTTTTATATTAGAGTAAAAATCCATTTGAAGCATAATATAGCATCTCATTTATCAATTTA : 2495
Tumaticot : AAAGTTGACATTATTTCACACCAGTTGATCCATTTTCTTTTATATTAGAGTAAAAATCCATTTGAAGCATAATATAGCATCTCATTTATCAATTTA : 2495
Corno     : AAAGTTGACATTATTTCACACCAGTTGATCCATTTTCTTTTATATTAGAGTAAAAATCCATTTGAAGCATAATATAGCATCTCATTTATCAATTTA : 2495
Cuneo     : AAAGTTGACATTATTTCACACCAGTTGATCCATTTTCTTTTATATTAGAGTAAAAATCCATTTGAAGCATAATATAGCATCTCATTTATCAATTTA : 2495
CM334_CNR : AAAGTTGACATTATTTCACACCAGTTGATCCATTTTCTTTTATATTAGAGTAAAAATCCATTTGAAGCATAATATAGCATCTCATTTATCAATTTA : 2496
Perennial : AAAGTTGACATTATTTCACACCAGTTGATCCATTTTCTTTTATATTAGAGTAAAAATCCATTTGAAGCATAATATAGCATCTCATTTATCAATTTA : 2495
            AAAGTTGACATTATTTCACACCAGTTGATCCATTTTCTTTTATATTAGAGTAAAAATCCATTTGAAGCATAATATAGCATCTCATTTATCAATTTA

            2500         *       2520         *       2540         *       2560         *       2580         *
Quadrato  : AGAACTTTTTTCTAAGAGAAATTTCGAGCCAAAGTAGAATTATAATAGCAAAAACATGAATGACTCAACTTTTAACTGAGGTGACGGTAGATCATT : 2591
Tumaticot : AGAACTTTTTTCTAAGAGAAATTTCGAGCCAAAGTAGAATTATAATAGCAAAAACATGAATGACTCAACTTTTAACTGAGGTGACGGTAGATCATT : 2591
Corno     : AGAACTTTTTTCTAAGAGAAATTTCGAGCCAAAGTAGAATTATAATAGCAAAAACATGAATGACTCAACTTTTAACTGAGGTGACGGTAGATCATT : 2591
Cuneo     : AGAACTTTTTTCTAAGAGAAATTTCGAGCCAAAGTAGAATTATAATAGCAAAAACATGAATGACTCAACTTTTAACTGAGGTGACGGTAGATCATT : 2591
CM334_CNR : AGAACTTTTTTCTAAGAGAAATTTCGAGCCAAAGTAGAATTATAATAGCAAAAACATGAATGACTCAACTTTTAACTGAGGTGACGGTAGATCATT : 2592
Perennial : AGAACTTTTTTCTAAGAGAAATTTCGAGCCAAAGTAGAATTATAATAGCAAAAACATGAATGACTCAACTTTTAACTGAGGTGACGGTAGATCATT : 2591
            AGAACTTTTTTCTAAGAGAAATTTCGAGCCAAAGTAGAATTATAATAGCAAAAACATGAATGACTCAACTTTTAACTGAGGTGACGGTAGATCATT

                2600         *       2620         *       2640         *       2660         *       2680
Quadrato  : TCACATAATTCAGGGAAATAGTTGACCTTTTGCCATTAAAAAATCATGGTAGATGTGTTCAATTCAAAAAGGATGATCAAGAATCTAATGCATGAA : 2687
Tumaticot : TCACATAATTCAGGGAAATAGTTGACCTTTTGCCATTAAAAAATCATGGTAGATGTGTTCAATTCAAAAAGGATGATCAAGAATCTAATGCATGAA : 2687
Corno     : TCACATAATTCAGGGAAATAGTTGACCTTTTGCCATTAAAAAATCATGGTAGATGTGTTCAATTCAAAAAGGATGATCAAGAATCTAATGCATGAA : 2687
Cuneo     : TCACATAATTCAGGGAAATAGTTGACCTTTTGCCATTAAAAAATCATGGTAGATGTGTTCAATTCAAAAAGGATGATCAAGAATCTAATGCATGAA : 2687
CM334_CNR : TCACATAATTCAGGGAAATAGTTGACCTTTTGCCATTAAAAAATCATGGTAGATGTGTTCAATTCAAAAAGGATGATCAAGAATCTAATGCATGAA : 2688
Perennial : TCACATAATTCAGGGAAATAGTTGACCTTTTGCCATTAAAAAATCATGGTAGATGTGTTCAATTCAAAAAGGATGATCAAGAATCTAATGCATGAA : 2687
            TCACATAATTCAGGGAAATAGTTGACCTTTTGCCATTAAAAAATCATGGTAGATGTGTTCAATTCAAAAAGGATGATCAAGAATCTAATGCATGAA

             *       2700         *       2720         *       2740         *       2760         *       2780
Quadrato  : ACTTTAACTAGCTACTAAAATGCACTTTGCAAAAAAATAAGACAATTGCAAAAAAAAAAAAATTCATATTGATCGACGTACAACGTATAAATTCACA : 2783
Tumaticot : ACTTTAACTAGCTACTAAAATGCACTTTGCAAAAAAATAAGACAATTGCAAAAAAAAAAAAATTCATATTGATCGACGTACAACGTATAAATTCACA : 2783
Corno     : ACTTTAACTAGCTACTAAAATGCACTTTGCAAAAAAATAAGACAATTGCAAAAAAAAAAAAATTCATATTGATCGACGTACAACGTATAAATTCACA : 2783
Cuneo     : ACTTTAACTAGCTACTAAAATGCACTTTGCAAAAAAATAAGACAATTGCAAAAAAAAAAAAATTCATATTGATCGACGTACAACGTATAAATTCACA : 2783
CM334_CNR : ACTTTAACTAGCTACTAAAATGCACTTTGCAAAAAAATAAGACAATTGCAAAAAAAAAAAAATTCATATTGATCGACGTACAACGTATAAATTCACA : 2784
Perennial : ACTTTAACTAGCTACTAAAATGCACTTTGCAAAAAAATAAGACAATTGCAAAAAAAAAAAAATTCATATTGATCGACGTACAACGTATAAATTCACA : 2783
            ACTTTAACTAGCTACTAAAATGCACTTTGCAAAAAAATAAGACAATTGCAAAAAAAAAAAAATTCATATTGATCGACGTACAACGTATAAATTCACA

                 *       2800         *       2820         *       2840         *       2860         *       2880
Quadrato  : TTAAAATTATTTTATTTTTCATCGTAGCGCAAGTTAGTCAAATAAAATTTCATCAACTTACGAGATTTCTTGGAAATGATGTTTGAGATACGTTTT : 2879
Tumaticot : TTAAAATTATTTTATTTTTCATCGTAGCGCAAGTTAGTCAAATAAAATTTCATCAACTTACGAGATTTCTTGGAAATGATGTTTGAGATACGTTTT : 2879
Corno     : TTAAAATTATTTTATTTTTCATCGTAGCGCAAGTTAGTCAAATAAAATTTCATCAACTTACGAGATTTCTTGGAAATGATGTTTGAGATACGTTTT : 2879
Cuneo     : TTAAAATTATTTTATTTTTCATCGTAGCGCAAGTTAGTCAAATAAAATTTCATCAACTTACGAGATTTCTTGGAAATGATGTTTGAGATACGTTTT : 2879
CM334_CNR : TTAAAATTATTTTATTTTTCATCGTAGCGCAAGTTAGTCAAATAAAATTTCATCAACTTACGAGATTTCTTGGAAATGATGTTTGAGATACGTTTT : 2880
Perennial : TTAAAATTATTTTATTTTTCATCGTAGCGCAAGTTAGTCAAATAAAATTTCATCAACTTACGAGATTTCTTGGAAATGATGTTTGAGATACGTTTT : 2879
            TTAAAATTATTTTATTTTTCATCGTAGCGCAAGTTAGTCAAATAAAATTTCATCAACTTACGAGATTTCTTGGAAATGATGTTTGAGATACGTTTT

                     *       2900         *       2920         *       2940         *       2960         *
Quadrato  : CGAAACTTCCACATTTCTTGAAACTGATTTTTTTTCTACCCGTTAAATTAGGACATAGTACCCTTTACCAAAAGTTAACTTCATCAACTCATTTGC : 2975
Tumaticot : CGAAACTTCCACATTTCTTGAAACTGATTTTTTTTCTACCCGTTAAATTAGGACATAGTACCCTTTACCAAAAGTTAACTTCATCAACTCATTTGC : 2975
Corno     : CGAAACTTCCACATTTCTTGAAACTGATTTTTTTTCTACCCGTTAAATTAGGACATAGTACCCTTTACCAAAAGTTAACTTCATCAACTCATTTGC : 2975
Cuneo     : CGAAACTTCCACATTTCTTGAAACTGATTTTTTTTCTACCCGTTAAATTAGGACATAGTACCCTTTACCAAAAGTTAACTTCATCAACTCATTTGC : 2975
CM334_CNR : CGAAACTTCCACATTTCTTGAAACTGATTTTTTTTCTACCCGTTAAATTAGGACATAGTACCCTTTACCAAAAGTTAACTTCATCAACTCATTTGC : 2976
Perennial : CGAAACTTCCACATTTCTTGAAACTGATTTTTTTTCTACCCGTTAAATTAGGACATAGTACCCTTTACCAAAAGTTAACTTCATCAACTCATTTGC : 2975
            CGAAACTTCCACATTTCTTGAAACTGATTTTTTTTCTACCCGTTAAATTAGGACATAGTACCCTTTACCAAAAGTTAACTTCATCAACTCATTTGC

            2980         *       3000
Quadrato  : TTCAAAACTTGTCTAAAAAAGAAAAA : 3000
Tumaticot : TTCAAAACTTGTCTAAAAAAGAAAAA : 3000
Corno     : TTCAAAACTTGTCTAAAAAAGAAAAA : 3000
Cuneo     : TTCAAAACTTGTCTAAAAAAGAAAAA : 3000
CM334_CNR : TTCAAAACTTGTCTAAAAAAGAAAAA : 3001
Perennial : TTCAAAACTTGTCTAAAAAAGAARA : 3000
            TTCAAAACTTGTCTAAAAAAGAAAaA
```

Figure S5

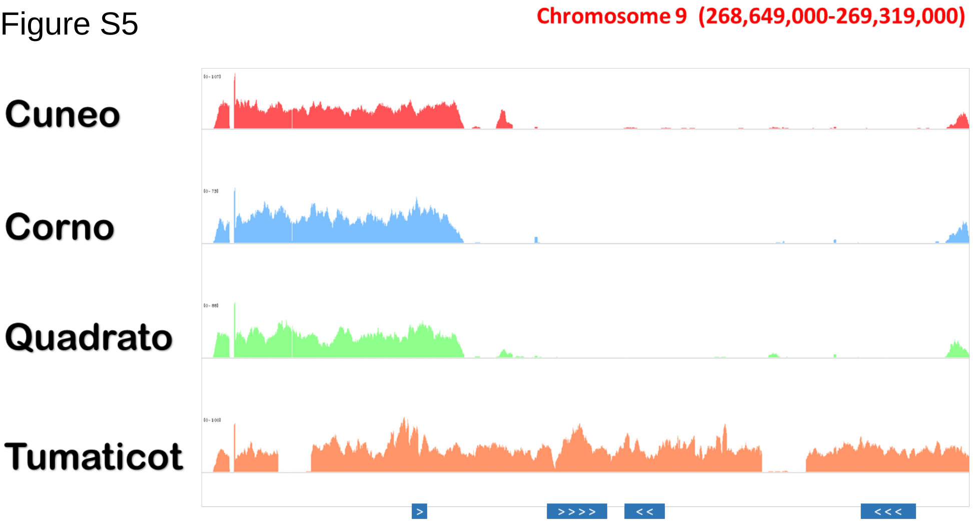
Chromosome 9 (268,649,000-269,319,000)
Cuneo
[0 - 107]
Corno
[0 - 73]
Quadrato
[0 - 88]
Tumaticot
[0 - 100]
>
> > > >
< <
< < <
